# Synthesis of Complex Tetracyclic Fused Scaffolds Enabled by (3 + 2) Cycloaddition

**DOI:** 10.1021/acs.orglett.4c01269

**Published:** 2024-05-31

**Authors:** Vincent Porte, Branca C. van Veen, Haoqi Zhang, Paolo Piacentini, Sergio Armentia Matheu, Sophie Woolford, Kevin R. Sokol, Saad Shaaban, Harald Weinstabl, Nuno Maulide

**Affiliations:** † Christian Doppler Laboratory for Entropy-Oriented Drug Design, Institute of Organic Chemistry, University of Vienna, 1090 Vienna, Austria; ‡ Boehringer Ingelheim RCV GmbH&CoKG, 1120 Vienna, Austria

## Abstract

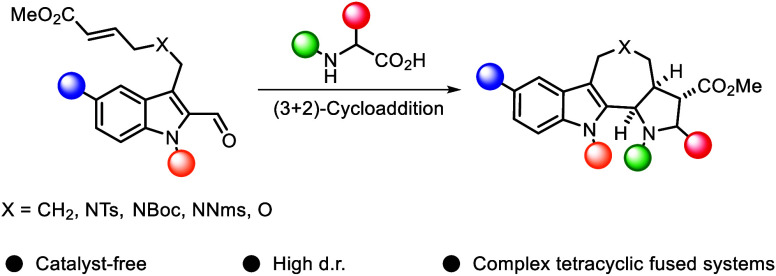

We describe the single-step formation of complex tetracyclic fused scaffolds enabled by (3 + 2) cycloaddition of azomethine ylides. Various indoles, N-protecting groups, and amino acids are well tolerated. The products are obtained in a catalyst-free manner with moderate to excellent yield and high diastereoselectivity. Representing a new scaffold that is not yet found in nature, the construction of pyrrolidine-fused cyclohepta-, azepino-, or oxepinoindoles could be found valuable in the synthesis of new pseudo-natural products.

Drug discovery is focused on rapidly finding drug candidates that are both active and synthetically easily accessible. In this regard, it often draws inspiration from the biological relevance and chemical diversity of natural products.^1^ The need to explore new chemical space has been facilitated by strategies such as fragment-based compound design.^2−4^ For example, formal fragmentation of natural products and subsequent recombination, in various arrangements, can deliver novel analogues.^5−7^ Such analogues, per se inaccessible through biosynthetic pathways, may inherit the biological and chemical properties of the original natural products and, thus, are called pseudo-natural products, a principle introduced by Waldmann et al. that has been shown effective for identifying new biologically active compounds.^8−11^

Azepino/cycloheptaindoles and pyrrolidines are common structural motifs in natural products (Scheme 1a).^12−15^ While the specific combination of those two moieties is rarely found in nature,^16,17^ it could embody a new scaffold for pseudonatural products (Scheme 1b). In this regard, 1,3-dipolar cycloadditions,^18−20^ and in particular azomethine ylide cycloadditions, have been widely applied to the construction of pyrrolidine-fused rings through cycloaddition.^21−25^ In the context of a drug discovery program, we were interested in the combination of the aforementioned natural product fragments in an efficient manner. Herein, we report the successful deployment of a (3 + 2) cycloaddition using azomethine ylides to combine pyrrolidine and azepinoindole or cycloheptaindole fragments (Scheme 1c).

**Scheme 1:**
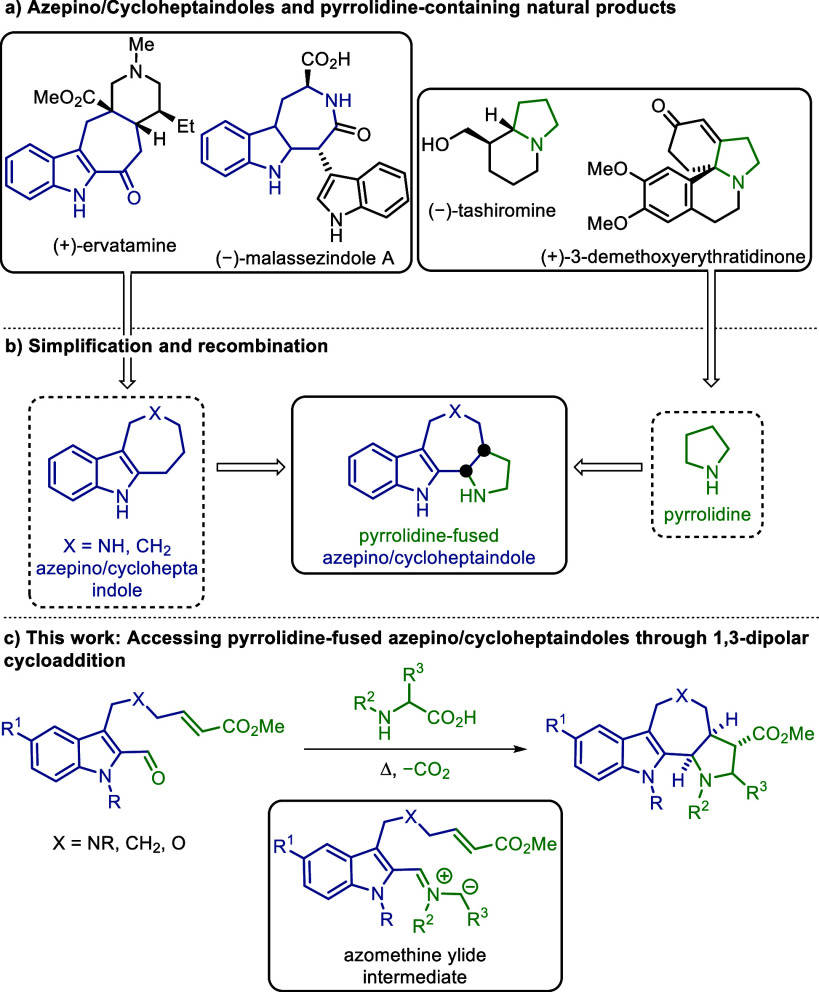
Design of a Complex Tetracyclic Scaffold on the Basis of Fragments Derived from Azepino/Cycloheptaindoles and Pyrrolidine-Containing Natural Products

This method is characterized by its high diastereoselectivity and moderate to excellent yields, which gives access to complex tetracyclic fused systems in a single step without the need for catalyst mediation.

We started our investigations by examining the (3 + 2) cycloaddition of **1** with sarcosine (**2a**) (Table 1). Using DMF as the solvent and a temperature of 80 °C, product **3** was obtained in a yield of 18% (entry 1). No conversion of **1** was observed when acetonitrile or toluene was used instead of DMF (entries 2 and 3). Increasing the reaction temperature led to improved yields of product **3** (entries 4 and 5), while the addition of 3 Å MS did not prove beneficial (entry 6). Upon isolation, product **3** was afforded in a maximum yield of only 21%. In addition, similar low isolated yields were obtained when having an electron-donating or electron-withdrawing substituent on the indole moiety (Supporting Information, section V).

**Table 1:**
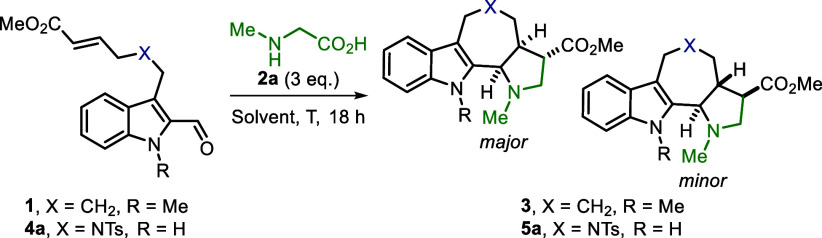
Optimization of the Reaction Conditions^a^

entry	solvent	T (°C)	X	yield (%)^b^	d.r.
1	DMF	80	CH_2_	18	>20:1
2	MeCN	80	CH_2_	0	n.d.
3	PhMe	80	CH_2_	0	n.d.
4	DMF	100	CH_2_	32	>20:1
5	DMF	120	CH_2_	40	>20:1
6	DMF^c^	120	CH_2_	30	>20:1
7	DMF	120	NTs	95	13:1
8	DMF	100	NTs	97	13:1
9	DMF	80	NTs	94	10:1
10	DMF	60	NTs	75	9:1
11^d^	DMF	100	NTs	73	13:1

a All reactions were performed with 0.1 mmol (1 equiv) of **1** or **4a** and 0.3 mmol of sarcosine **2a** (3 equiv) in 1 mL of solvent (0.1 M).

b Yields were determined by ^1^H NMR using CH_2_Br_2_ as internal standard. Isolated yields are shown in parentheses.

c To the reaction was added 3 Å MS.

d Using 1.5 equiv of sarcosine **2a**. n.d., not detected.

In an attempt to improve yields, we considered whether the Thorpe–Ingold effect could be used to our advantage in the (3 + 2) cycloaddition to promote ring closure and in turn increase molecular complexity. Encouragingly, the reaction of **4a** with sarcosine in DMF at 120 °C resulted in the formation of product **5a** in an excellent yield of 95% (Table 1, entry 7). Similar yields were observed when the reaction temperature was lowered to 100 and 80 °C, yet a decreased diastereoselectivity was observed in case of the latter (entries 8 and 9). Changing the solvent to acetonitrile, toluene, or dichloroethane (DCE) with a reaction temperature of 80 °C led to decreased yields of **5a** (see the Supporting Information, section II). A significant decrease in the yield was also observed when the reaction was performed at 60 °C in DMF (Table 1, entry 10). Finally, reducing the amounts of sarcosine afforded product **5a** in lower yield but with the same diastereoselectivity (entry 11).

With the optimized conditions in hand (Table 1, entry 8), we investigated the generality of this cycloaddition (Scheme 2). Notably, the scalability of the method was demonstrated by executing the reaction of **4a** at a 1 mmol scale, which generated **5a** without erosion in yield and diastereoselectivity. Product **5b** bearing an *N*-Me indole was afforded in a good yield. This demonstrated that *N*-alkylated indoles are suitable substrates for the (3 + 2) cycloaddition when the Thorpe–Ingold effect is in play. Different substituents, both electron-donating and -withdrawing, on the indole-2-carbaldehyde were also well tolerated, and products **5c**, **5d**, and **5e** were afforded in good yields and high diastereoselectivity. Furthermore, another heterocycle, oxepinoindole **5f**, could be constructed in moderate yield.

**Scheme 2:**
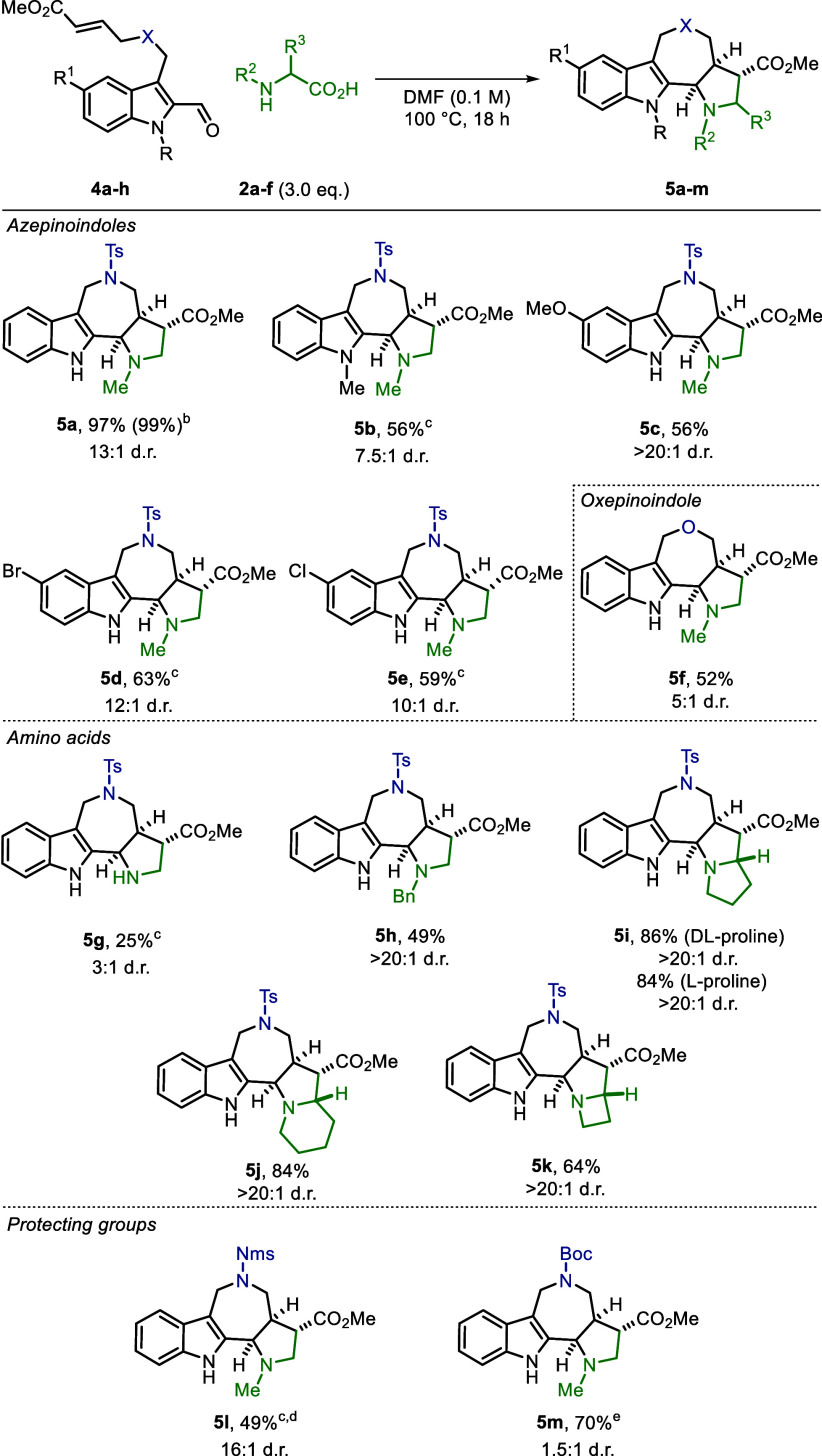
Scope of the (3 + 2) Cycloaddition of Indole-2-carbaldehydes Reactions were carried out on 0.03–0.3 mmol scale, and combined isolated yields were reported unless otherwise stated. Scale-up: 1 mmol. Minor diastereomer was not isolated. Reaction was performed at 60 °C. Unseparable mixture of diastereomers.

Next, we investigated the use of different amino acids and observed tolerance with glycine and *N*-benzyl glycine, which gave the corresponding products (**5g** and **5h**) in moderate to good yields (Scheme 2). Of particular note is the deployment of proline, pipecolic acid, and azetidine-2-carboxylic acid as amino acids in this process, which led to products where an additional stereocenter is incorporated (**5i**–**k**) without affecting the excellent diastereoselectivity observed before.

These products open up the possibility of further postfunctionalization and increase in the structural complexity. Additionally, the scope of the (3 + 2) cycloaddition was examined using various protected amines to allow for milder deprotection conditions. Good results were obtained with both Nms^26^ and Boc protecting groups, which provided products **5l** and **5m**, respectively.

To explore whether alternative dipoles would be compatible with this (3 + 2) cycloaddition, nitrone-containing compounds were prepared and submitted to the standard reaction conditions (Scheme 3). However, when α,β-unsaturated ester **6a** was employed, no desired product **7a** was observed. Instead, the (3 + 2) cycloaddition of **6b** bearing a terminal alkene afforded **7b** as a single diastereomer in low yield, which featured an oxazolidine-fused azepinoindole analogue. Interestingly, the eight-membered bridged cycloadduct **8b** of which the structure was confirmed by X-ray crystallography was isolated as the major product. With a more sterically hindered R′ group (Ph instead of H), only eight-membered bridged cycloadduct **8c** was formed.

**Scheme 3:**
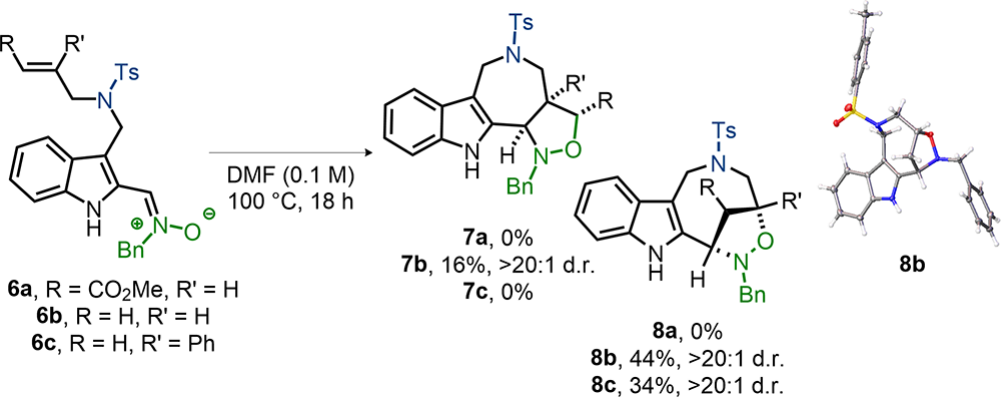
(3 + 2) Cycloaddition of Nitrone-Containing Substrates

Furthermore, oxime-containing substrates **9a**,**b** were submitted to oxidative conditions, which led to (3 + 2) cycloaddition forming isoxazoline products **10a**,**b** that resemble a scaffold with antimicrobial activity (Scheme 4).^27^ In contrast to the nitrone-containing compounds, no eight-membered bridged cycloadducts were observed. It is noteworthy that methylation of the indole proved to be crucial as only traces of product were observed with free indole motifs.

**Scheme 4:**
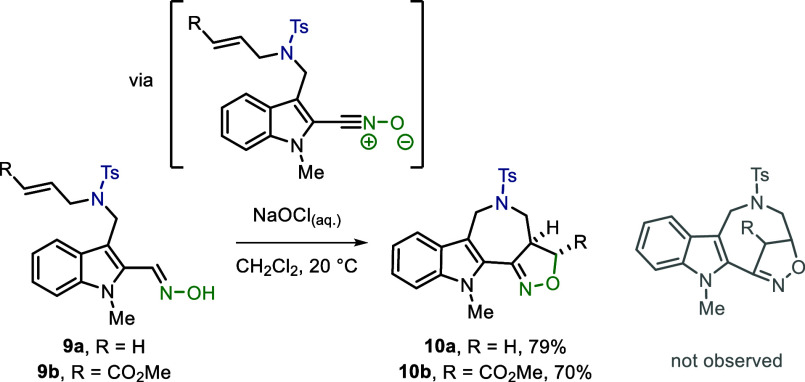
(3 + 2) Cycloaddition of In Situ Generated Nitrile Oxide-Containing Substrates

In conclusion, we have developed the synthesis of complex tetracyclic fused scaffolds in a single step by (3 + 2) cycloaddition of azomethine ylides. Good to excellent yields were achieved, and the reaction shows tolerance of several amino acids, N-protecting groups, and variously substituted indoles while delivering highly complex products in excellent diastereoselectivities. We believe this scaffold could potentially represent a new pseudo-natural product and display interesting bioactivities.

## Data Availability

The data underlying this study are available in the published article and its Supporting Information.

## Supporting Information Available

Experimental procedures, spectroscopic characterization data, and X-ray analysis (PDF)
