# Supplementary material for: Synthesis of Complex Tetracyclic Fused Scaffolds Enabled by (3 + 2) Cycloaddition
Source: Org Lett. 2024 May 31;26(23):4873–6. doi: 10.1021/acs.orglett.4c01269 (PMC11187634; doi:10.1021/acs.orglett.4c01269)
Supplement: Supplementary file 1 — ol4c01269_si_001.pdf [file ol4c01269_si_001.pdf]

Supporting Information

Synthesis of Complex Tetracyclic Fused Scaffolds Enabled by (3+2)-Cycloaddition

Vincent Porte,[a,+] Branca C. van Veen,[a,+] Haoqi Zhang,[a,+] Paolo Piacentini,[a,+] Sergio Armentia Matheu,[a,+] Sophie Woolford,[a] Kevin Sokol,[b] Saad Shaaban,[a] Harald Weinstabl[b] and Nuno Maulide*

[a] Christian Doppler Laboratory for Entropy-Oriented Drug Design, Institute of Organic Chemistry, University of Vienna, 1090 Vienna, Austria

[b] Boehringer Ingelheim RCV GmbH&CoKG, 1120 Vienna, Austria

E-mail: *Correspondence: nuno.maulide@univie.ac.at

I. GENERAL INFORMATION 4

II. OPTIMIZATION 5

III. UNSUCCESSFUL SUBSTRATES 6

IV. SYNTHESIS AND CHARACTERIZATION OF STARTING MATERIALS 7

A. SYNTHESIS OF METHYL (*E*)-6-(2-FORMYL-1-METHYL-1*H*-INDOL-3-YL)HEX-2-ENOATE (1) 7

B. SYNTHESIS OF METHYL (*E*)-6-(5-BROMO-2-FORMYL-1-METHYL-1*H*-INDOL-3-YL)HEX-2-ENOATE (1^{BR}) 15

C. SYNTHESIS OF METHYL (*E*)-6-(2-FORMYL-5-METHOXY-1-METHYL-1*H*-INDOL-3-YL)HEX-2-ENOATE (1^{OME}) 22

D. SYNTHESIS OF METHYL (*E*)-4-((N-((2-FORMYL-1*H*-INDOL-3-YL)METHYL)-4-METHYLPHENYL)SULFONAMIDO)BUT-2-ENOATE (4A) 30

E. SYNTHESIS OF METHYL (*E*)-4-((*N*-((2-FORMYL-1-METHYL-1*H*-INDOL-3-YL)METHYL)-4-METHYLPHENYL)SULFONAMIDO)BUT-2-ENOATE (4B) 37

F. SYNTHESIS OF METHYL (*E*)-4-((*N*-((2-FORMYL-5-METHOXY-1*H*-INDOL-3-YL)METHYL)-4-METHYLPHENYL)SULFONAMIDO)BUT-2-ENOATE (4C) 41

G. SYNTHESIS OF METHYL (*E*)-4-((*N*-((5-BROMO-2-FORMYL-1*H*-INDOL-3-YL)METHYL)-4-METHYLPHENYL)SULFONAMIDO)BUT-2-ENOATE (4D) 48

H. SYNTHESIS OF METHYL (*E*)-4-((*N*-((5-CHLORO-2-FORMYL-1*H*-INDOL-3-YL)METHYL)-4-METHYLPHENYL)SULFONAMIDO)BUT-2-ENOATE (4E) 55

I. SYNTHESIS OF METHYL (*E*)-4-((2-FORMYL-1*H*-INDOL-3-YL)METHOXY)BUT-2-ENOATE (4F) 63

J. SYNTHESIS OF METHYL (*E*)-4-((*N*-((2-FORMYL-1H-INDOL-3-YL)METHYL)-2,4,6-TRIS(TRIFLUOROMETHYL)PHENYL)SULFONAMIDO)BUT-2-ENOATE (4G) 72

K. SYNTHESIS OF METHYL (*E*)-4-((TERT-BUTOXYCARBONYL)((2-FORMYL-1*H*-INDOL-3-YL)METHYL)AMINO)BUT-2-ENOATE (4H) 81

SYNTHESIS OF (*Z*)-1-(3-(((*N*-ALLYL-4-METHYLPHENYL)SULFONAMIDO)METHYL)-1*H*-INDOL-2-YL)-N-BENZYLMETHANIMINE OXIDE (6B) 87

SYNTHESIS OF METHYL (*E*)-4-((TERT-BUTOXYCARBONYL)((2-FORMYL-1*H*-INDOL-3-YL)METHYL)AMINO)BUT-2-ENOATE (6C) 89

V. SYNTHESIS AND CHARACTERIZATION OF PRODUCTS 98

VI. X-RAY ANALYSIS 163

VII. REFERENCES 165

I. General information

Unless otherwise stated, all glassware was flame-dried with a stirring bar before use and all reactions were performed under an atmosphere of argon. All solvents were used as received, if anhydrous, or distilled from appropriate drying agents before use. When heating was required an oil bath was used. All reagents were used as received from commercial suppliers unless otherwise stated. Reaction progress was monitored by thin layer chromatography (TLC) performed on aluminum plates coated with silica gel F254 with 0.2 mm thickness. Chromatograms were visualized by fluorescence quenching with UV light at 254 nm or by staining using potassium permanganate or phosphomolybdic acid. Flash column chromatography was performed using silica gel 60 (230-400 mesh, Merck and co.) or prepacked columns (Chromabond silica) using a Biotage Selekt Flash Purification System. Neat infrared spectra were recorded using a Perkin-Elmer Spectrum 100 FT-IR spectrometer. Wavenumbers (ν_{max}) are reported in cm^{-1}. Mass spectra were obtained using a Finnigan MAT 8200 or (70 eV) or an Agilent 5973 (70 eV) spectrometer, using electrospray ionization (ESI). All ^{1}H NMR and ^{13}C NMR spectra were recorded using a Bruker AV-400, AV-600 and AV-700 spectrometer at 300K. Chemical shifts (δ) were given in "parts per million" (ppm), referenced to using the solvent as internal standard according to Fulmer *et al*.[1]. Coupling constants are quoted in Hz (*J*). Spectroscopy splitting patterns were designated as singlet (s), doublet (d), triplet (t), quartet (q), quintuplet (qp), multiplet (m), br (broad) or combinations of that as the observed pattern. DEPTQ ^{13}C spectra are phased such that CH_2/C signals are up, and CH/CH_3 signals are down, or vice versa. Structural assignments were made with additional information from gCOSY, gNOESY, gHSQC, and gHMBC experiments. DMA stands for the solvent combination of CH_2Cl_2/methanol/ammonium hydroxide aqueous solution (~ 25% ammonia content) in a ratio of 90:9:1.

II. Optimization

Table 1: Additional Optimization of the Reaction Conditions

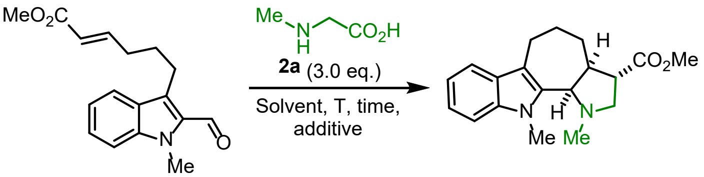

Entry	Time (h)	T (°C)	Solvent (M)	**2a** (eq.)	Additive (eq.)	Yield (%)[a]
1	18 h	80 °C	DMF (0.1)	3	-	18
2	67 h	80 °C	DMF (0.1)	3	-	18
3	18 h	100 °C	DMF (0.1)	3	-	32
4	18 h	80 °C	DMA (0.1)	3	-	20
5	18 h	80 °C	PhMe (0.1)	3	-	0
6	18 h	80 °C	MeCN (0.1)	3	-	0
7	18 h	120 °C	DMF (0.1)	3	-	40
8	18 h	150 °C	DMF (0.1)	3	-	31
9	18 h	120 °C	DMF (0.1)	10	-	32
10	18 h	120 °C	DMF (0.5)	3	-	20
11	18 h	120 °C	DMF (0.1)	1.2	-	11
12	18 h	120 °C	DMF (0.1)	3	TEA (3.0)	32
13	18 h	120 °C	DMF (0.1)	3	3 Å MS-	21
14	18 h	110 °C	PhMe (0.1)	3	-	0
15	18 h	110 °C	PhMe (0.1)	3	$Sc(OTf)_3$ (0.2)	0
16	18 h	58 °C	HFIP (0.1)	3	-	0
17	18 h	100 °C	$MeNO_2$ (0.1)	3	-	0
18	18 h	100 °C	$MeNO_2$ (0.1)	3	$Sc(OTf)_3$ (0.2)<br>3 Å MS	0

[a]Yields were determined by ^1H NMR using CH_2Br_2 as an internal standard.

Table 2: Additional Optimization of the Reaction Conditions[a]

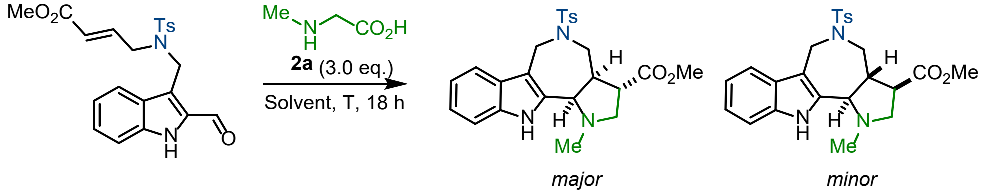

Entry	Solvent	T (°C)	Yield (%)[b]	d.r.
1	MeCN	80	76	9:1
2	toluene	80	47	>20:1
3	DCE	80	28	n.d.

[a]All reactions were performed with 0.1 mmol (1.0 eq.) of **4a** and 0.3 mmol of sarcosine **2a** (3.0 eq.) in 1 mL of solvent (0.1 M). [b]Yields were determined by ^{1}H NMR analysis using CH_2Br_2 as an internal standard.

III. Unsuccessful substrates

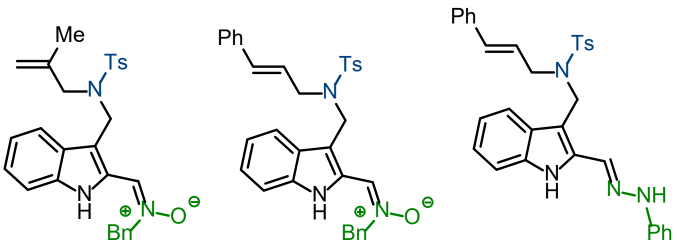

IV. Synthesis and characterization of starting materials

a. Synthesis of methyl (*E*)-6-(2-formyl-1-methyl-1*H*-indol-3-yl)hex-2-enoate (1)

3-(pent-4-en-1-yl)-1*H*-indole (SM-1-1)

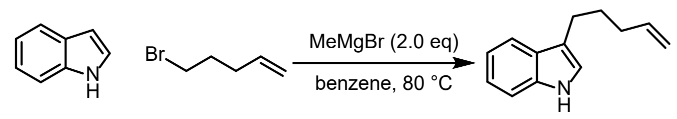

(1.5 eq) (1.0 eq) **SM-1-1**: 49%

Benzene was dried over 3Å molecular sieves before use. A 100 mL round-bottomed flask was charged with indole (576 mg, 4.92 mmol, 1.5 eq.) and 10 mL benzene. Methylmagnesium bromide (3 M in Et_2O, 2.19 mL, 6.56 mmol, 2.0 eq.) was added dropwise and the mixture was stirred for 10 min at 25 °C. 5-Bromo-1-pentene (0.38 mL, 3.28 mmol, 1.0 eq.) was added and the reaction was refluxed for 22 h. After returning to ambient temperature, the work up was performed by addition of a sat. aq. solution of NH_4Cl (30 mL) and extraction with EtOAc (30 mL). The organic layer was washed with brine (30 mL), dried over $MgSO_4$, filtered and concentrated under reduced pressure. Purification was performed by flash chromatography using a gradient of heptanes/EtOAc to yield **SM-1-1** as a yellow oil (298 mg, 1.61 mmol, 49% yield).

The analytical data is in accordance to previous report.[2]

^{1}H NMR (400 MHz, $CDCl_3$) δ 7.83 (br s, 1H), 7.54 (d, *J* = 7.9 Hz, 1H), 7.29 (d, *J* = 8.1 Hz, 1H), 7.15 – 7.08 (m, 1H), 7.07 – 7.00 (m, 1H), 6.91 (d, *J* = 2.1 Hz, 1H), 5.80 (ddt, *J* = 16.9, 10.2, 6.6 Hz, 1H), 5.03 – 4.88 (m, 2H), 2.71 (t, *J* = 7.6 Hz, 2H), 2.09 (dd, *J* = 14.2, 7.3 Hz, 2H), 1.81 – 1.69 (m, 2H).

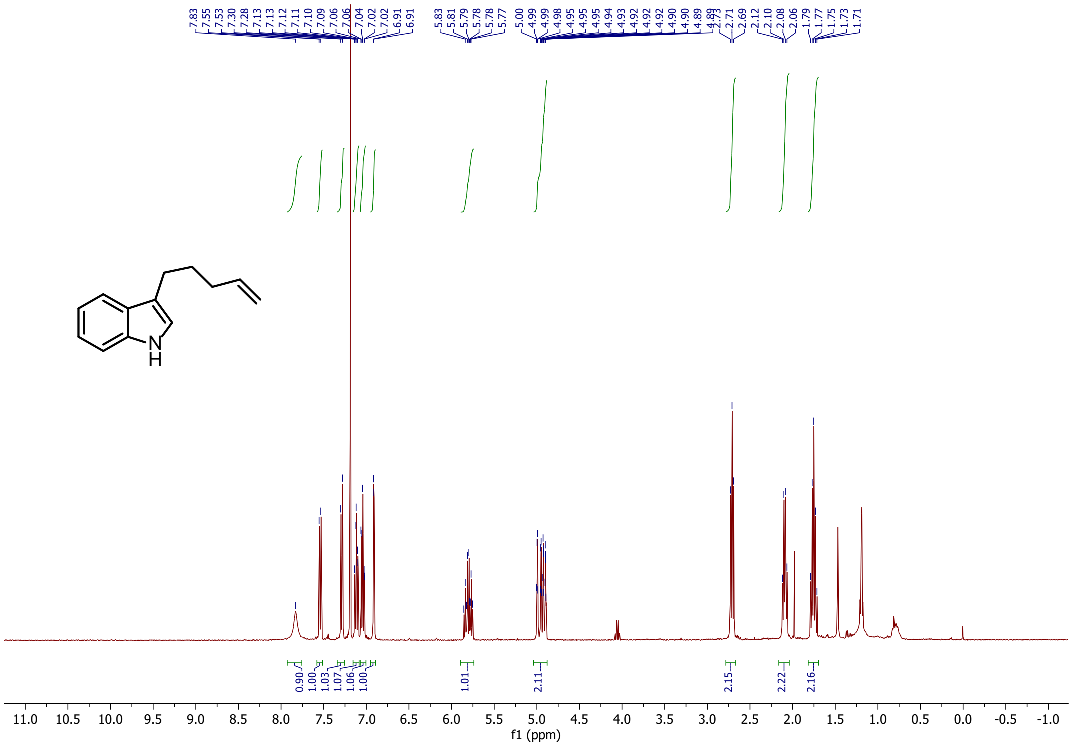

Figure 1: ***^{1}H NMR (400 MHz, CDCl₃) of SM-1-1.***

1-methyl-3-(pent-4-en-1-yl)-1*H*-indole (SM-1-2)

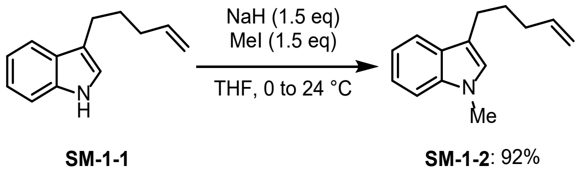

A 50-mL Schlenk flask was charged with NaH (60% in paraffine, 94 mg, 2.35 mmol, 1.5 eq.) and THF (6 mL), and cooled to 0 °C. **SM-1-1** (290 mg, 1.57 mmol, 1.0 eq.) was added dropwise as a solution in THF (2 mL). The resulting mixture was stirred for 30 min at 0 °C, before the addition of iodomethane (146 µL, 2.35 mmol, 1.5 eq.) at the same temperature. The resulting mixture was stirred for 3 h at 24 °C. Work-up was performed by dilution with EtOAc (30 mL) and sat. aq. solution of NH_4Cl (30 mL). The phases were separated and the aqueous phase was extracted twice with EtOAc (15 mL). The organic phases were combined, dried over Na_2SO_4 and filtered. The solution was concentrated under reduced pressure and the crude mixture was purified by flash chromatography using a gradient of heptanes/EtOAc to yield **SM-1-2** as a yellow oil (289 mg, 1.45 mmol, 92% yield).

^{1}H NMR (600 MHz, $CDCl_3$) δ 7.59 (d, J = 7.9 Hz, 1H), 7.29 (d, J = 8.2 Hz, 1H), 7.24 – 7.19 (m, 1H), 7.13 – 7.06 (m, 1H), 6.83 (s, 1H), 5.87 (ddt, J = 16.9, 10.2, 6.6 Hz, 1H), 5.09 – 4.94 (m, 2H), 3.75 (s, 3H), 2.76 (t, J = 7.5 Hz, 2H), 2.19 – 2.12 (m, 2H), 1.84 – 1.77 (m, 2H).

^{13}C NMR (151 MHz, $CDCl_3$) δ 139.1, 137.2, 128.1, 126.2, 121.5, 119.2, 118.6, 115.3, 114.7, 109.2, 33.8, 32.7, 29.7, 24.6 ppm. *One quaternary sp^2 carbon could not be found under these conditions.*

HRMS (ESI-TOF) m/z: $[M + H]^+$ Calcd for $C_{14}H_{18}N$ 200.1434; Found 200.1425.

IR (neat) vmax: 3074, 3055, 2974, 2930, 2855, 1711, 1640, 1614, 1471, 910, 739.

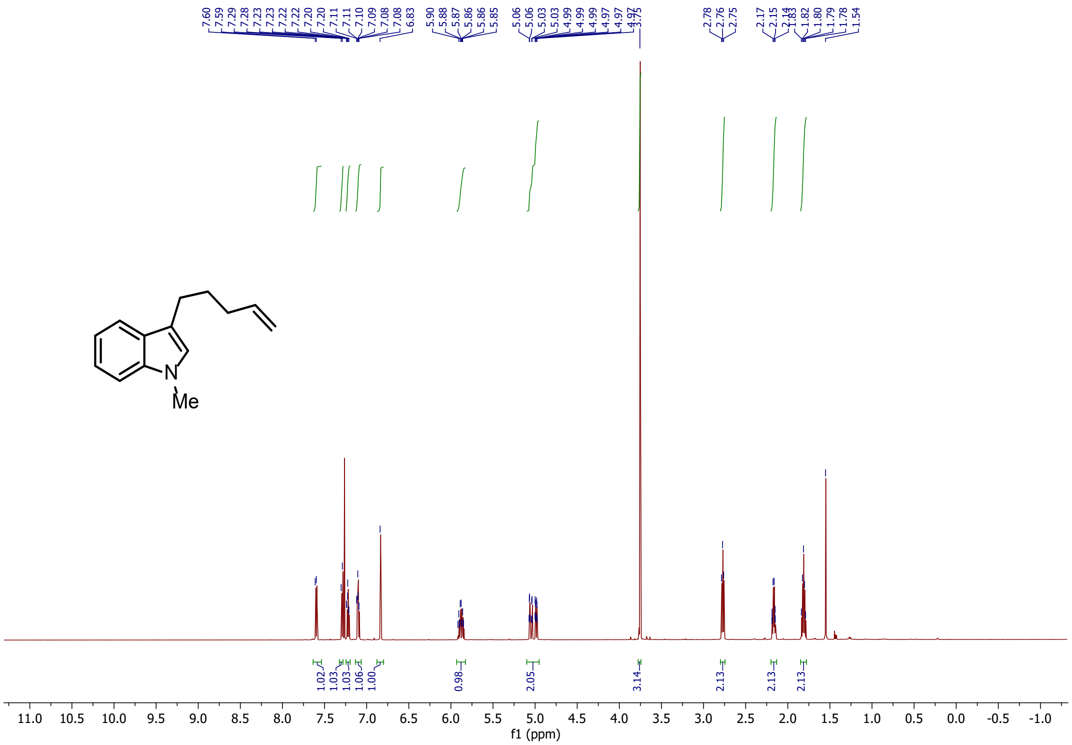

Figure 2: ***^{1}H NMR (600 MHz, $CDCl_3$) of SM-1-2.***

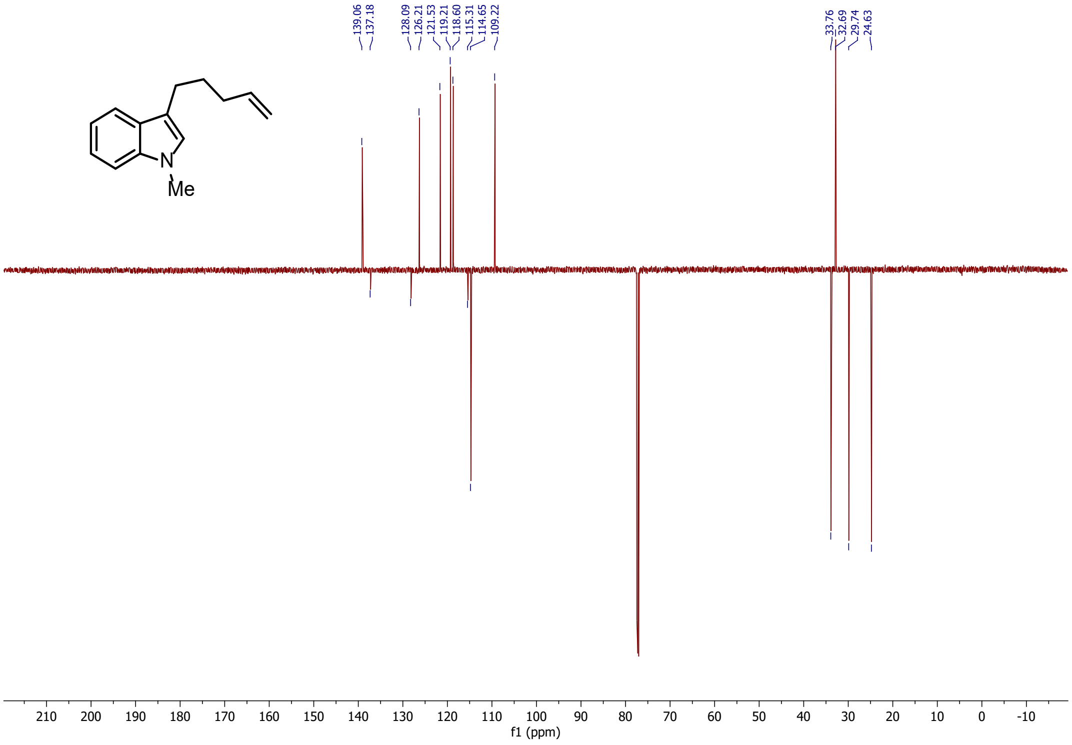

Figure 3: ***^{13}C NMR (151 MHz, $CDCl_3$) of SM-1-2.***

1-methyl-3-(pent-4-en-1-yl)-1*H*-indole-2-carbaldehyde (SM-1-3)

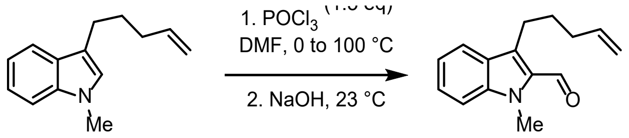

SM-1-2 **SM-1-3**: 77%

A 25 mL Schlenk flask was charged with **SM-1-2** (152 mg, 0.76 mmol, 1.0 eq.) and DMF (3.8 mL), before cooling to 0 °C. Phosphorus(V) oxychloride (0.11 mL, 1.14 mmol, 1.5 eq.) was added dropwise and the resulting mixture was stirred at 100 °C for 1 h. Afterwards, the reaction was cooled to 0 °C, before addition of an aq. solution of NaOH (2 M, 2 mL) and stirring for 15 min at 23 °C. Work-up was performed by dilution with EtOAc (10 mL) and H_2O (10 mL). The phases were separated and the aqueous phase was extracted twice with EtOAc (10 mL). The organic phases were combined, dried over Na_2SO_4 and filtered. The solution was concentrated under reduced pressure and the crude mixture was purified by flash chromatography using a gradient of heptanes/EtOAc to yield **SM-1-3** as a yellow oil (134 mg, 0.59 mmol, 77% yield).

^{1}H NMR (600 MHz, $CDCl_3$) δ 10.13 (s, 1H), 7.71 (dt, *J* = 8.1, 0.9 Hz, 1H), 7.46 – 7.39 (m, 1H), 7.35 (d, *J* = 8.5 Hz, 1H), 7.15 (ddd, *J* = 7.9, 6.9, 0.9 Hz, 1H), 5.84 (ddt, *J* = 16.9, 10.2, 6.6 Hz, 1H), 5.10 – 4.94 (m, 2H), 4.06 (s, 3H), 3.16 – 3.03 (m, 2H), 2.21 – 2.09 (m, 2H), 1.91 – 1.77 (m, 2H).

^{13}C NMR (151 MHz, $CDCl_3$) δ 181.7, 140.0, 138.2, 131.4, 127.4, 126.4, 121.6, 120.3, 115.4, 110.4, 33.5, 31.8, 31.5, 23.2. *One quaternary sp^2 carbon could not be found under these conditions.*

HRMS (ESI-TOF) m/z: $[M + H]^+$ Calcd for $C_{15}H_{18}NO$ 228.1383; Found 228.1378.

IR (neat) vmax: 3060, 2974, 2931, 2861, 1660, 1613, 1489, 1382, 743.

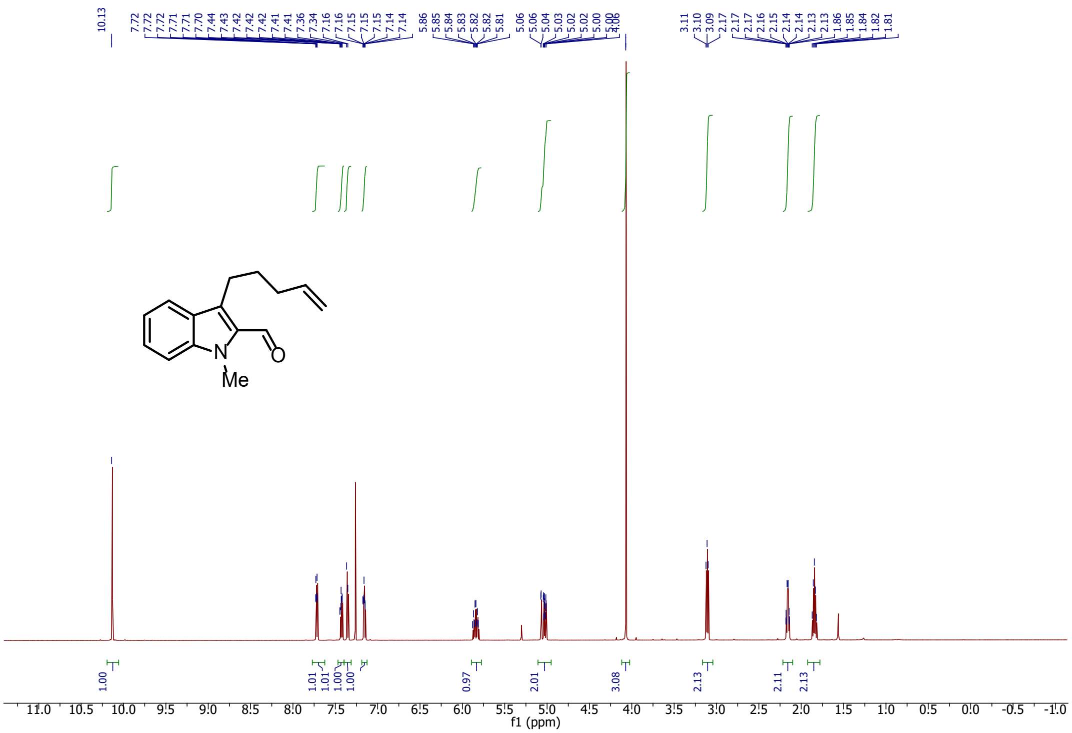

Figure 4: ***^{1}H NMR (600 MHz, CDCl3) of SM-1-3.***

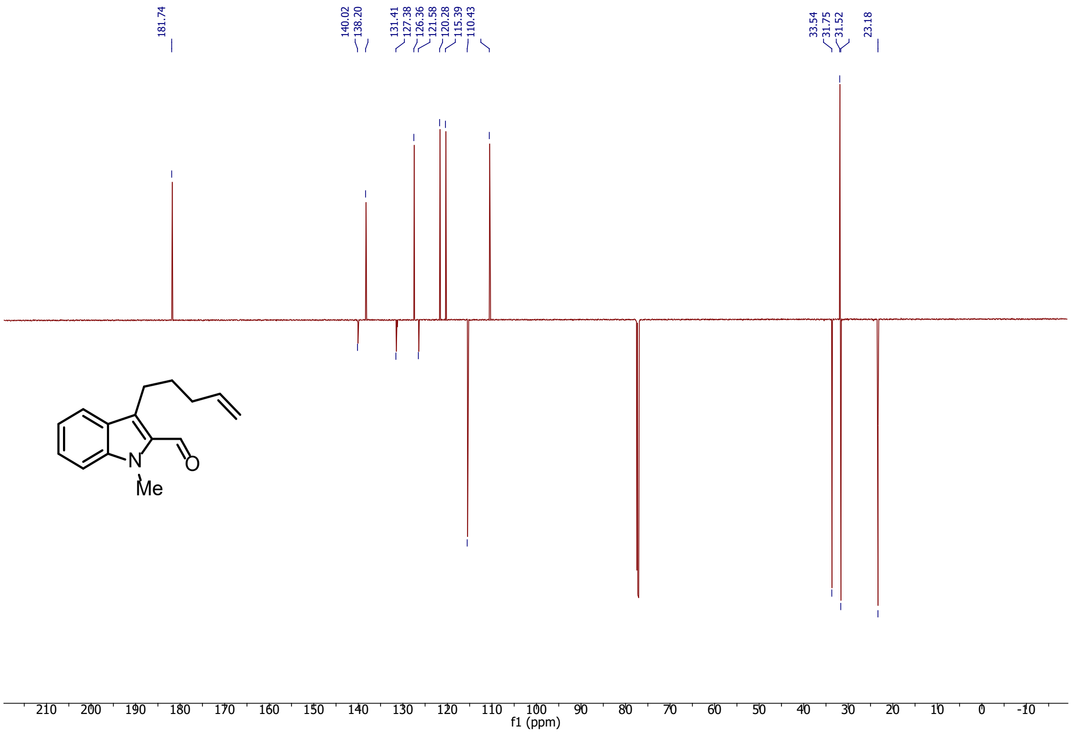

Figure 5: ***^{13}C NMR (151 MHz, CDCl3) of SM-1-3.***

Methyl (*E*)-6-(2-formyl-1-methyl-1*H*-indol-3-yl)hex-2-enoate (1)

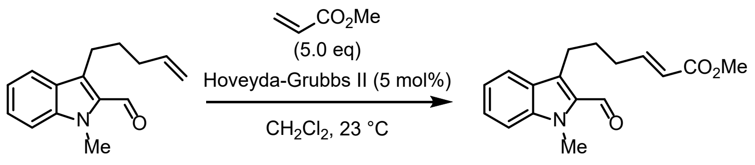

A Schlenk flask was charged with **SM-1-3** (150 mg, 0.66 mmol, 1.0 eq.), CH_2Cl_2 (6.6 mL), methyl acrylate (0.27 mL, 3.30 mmol, 5.0 eq.) and Hoveyda-Grubbs 2nd generation catalyst (21 mg, 0.033 mmol, 5 mol%). The mixture was stirred for 3 h at 23 °C, before being concentrated under reduced pressure. The crude mixture was purified twice by flash chromatography using a gradient of heptanes/EtOAc to yield **1** as a brown oil (174 mg, 0.61 mmol, 92% yield).

^{1}H NMR (600 MHz, CDCl$_3$) δ 10.13 (s, 1H), 7.69 (d, *J* = 8.1 Hz, 1H), 7.43 (ddd, *J* = 8.1, 6.9, 1.0 Hz, 1H), 7.36 (d, *J* = 8.5 Hz, 1H), 7.16 (ddd, *J* = 7.9, 6.9, 0.8 Hz, 1H), 6.97 (dt, *J* = 15.6, 6.9 Hz, 1H), 5.84 (dt, *J* = 15.6, 1.5 Hz, 1H), 4.06 (s, 3H), 3.72 (s, 3H), 3.13 (t, *J* = 7.6 Hz, 2H), 2.33 – 2.25 (m, 2H), 1.95 – 1.87 (m, 2H).

^{13}C NMR (151 MHz, CDCl$_3$) δ 181.5, 167.1, 148.6, 140.0, 130.3, 127.5, 126.3, 121.7, 121.4, 120.5, 110.5, 51.6, 32.0, 31.7, 30.6, 23.3 ppm. *One quaternary sp^2 carbon could not be found under these conditions.*

HRMS (ESI-TOF) m/z: $[M + H]^+$ Calcd for $C_{17}H_{20}NO_3$ 286.1438; Found 286.1433.

IR (neat) νmax:. 2946, 2862, 2734, 1720, 1657, 1204, 746.

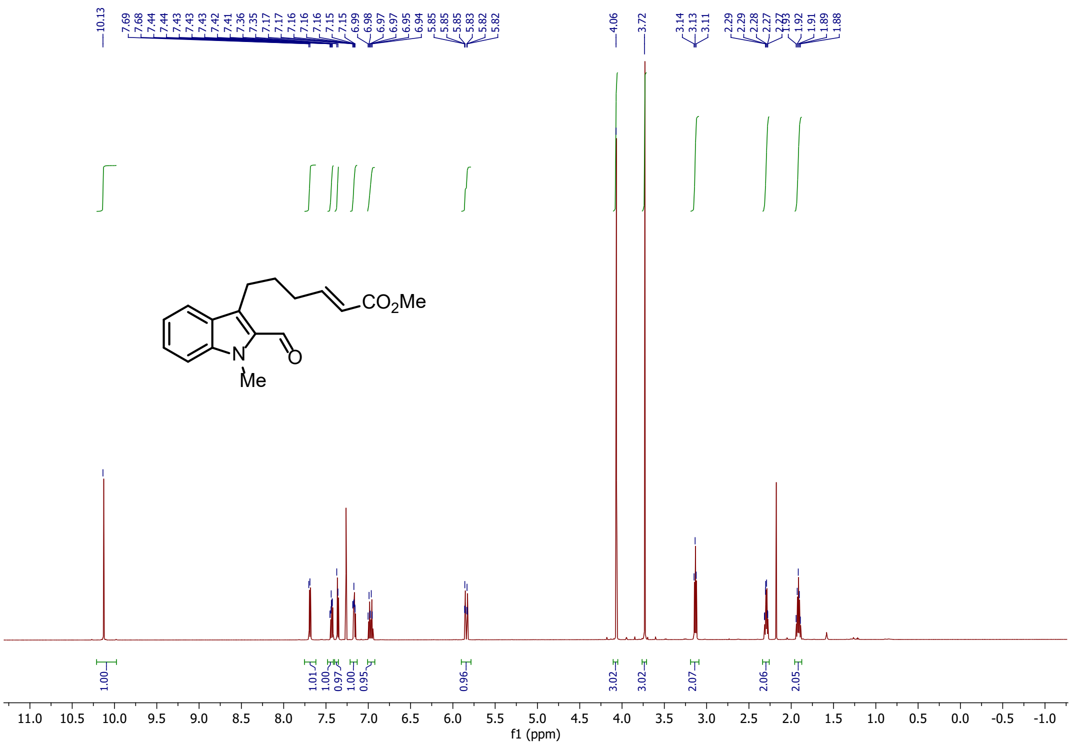

*Figure 6: **^{1}H NMR (600 MHz, CDCl3) of 1.***

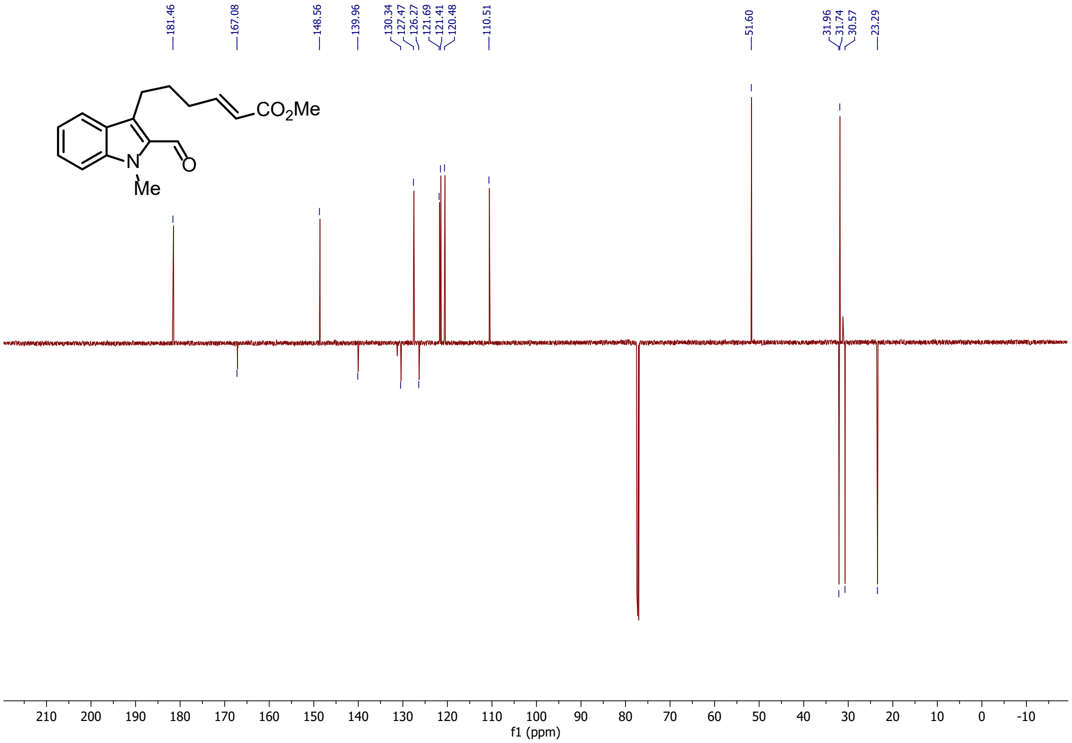

*Figure 7: **^{13}C NMR (151 MHz, CDCl3) of 1.***

b. Synthesis of methyl (*E*)-6-(5-bromo-2-formyl-1-methyl-1*H*-indol-3-yl)hex-2-enoate (1^{Br})

5-bromo-1-methyl-3-(pent-4-en-1-yl)-1*H*-indole-2-carbaldehyde (SM-1^{Br}-3)

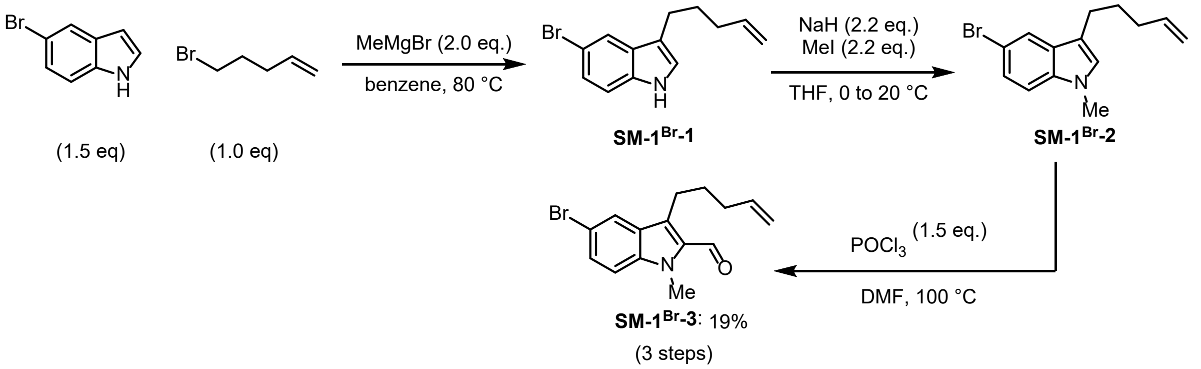

Benzene was dried over 3Å molecular sieves before use. A 25-mL Schlenk flask was charged with 5-bromoindole (996 mg, 5.80 mmol, 1.50 eq.) and dried benzene (10 mL). Methylmagnesium bromide (3 M in Et_2O, 2.19 ml, 6.56 mmol, 2.0 eq.) was added dropwise and the mixture was stirred for 10 min at 25 °C. 5-Bromo-1-pentene (0.39 ml, 3.28 mmol, 1.0 eq.) was added and the reaction was refluxed for 22 h. After returning to ambient temperature, the work up was performed by addition of a sat. aq. solution of NH_4Cl (30 mL) and extraction with EtOAc (30 mL). The organic layer was washed with brine (30 mL), dried over $MgSO_4$, filtered and concentrated under reduced pressure. Purification was attempted by flash chromatography using a gradient of heptanes/EtOAc to yield a co-eluting mixture consisting of **SM-1^{Br}-1** and 30% of starting material. The resulting mixture was used assumed pure and without any further purification.

A 25-mL Schlenk flask equipped with a stirring bar was charged with NaH (60% in paraffine, 144 mg, 3.61 mmol, 2.20 eq.) and THF (7 mL), and cooled to 0 °C. **SM-1^{Br}-1** (441 mg, 1.67 mmol, 1.00 eq.) was added dropwise as a solution in THF (2 mL). The resulting mixture was stirred for 30 min at 0 °C, before the addition of iodomethane (225 µL, 3.60 mmol, 2.20 eq.) at the same temperature. The resulting mixture was stirred for 3 h at 20 °C. Work-up was performed by dilution with EtOAc (50 mL) and a sat. aq. solution of NH_4Cl (40 mL). The phases were separated and the aqueous phase was extracted twice with EtOAc (50 mL). The organic phases were combined, dried over $MgSO_4$ and filtered. The solution was concentrated under reduced pressure. Several attempts of purification were performed, but it was not possible to separate the co-eluting methylated **SM-1^{Br}-1** and **SM-1^{Br}-2**. Only a small amount of **SM-1^{Br}-2** was obtained pure as a pale-yellow solid via flash chromatography using a gradient of heptanes/EtOAc (see characterization below). The rest was assumed pure and used without further purification.

SM-1[Br]-2

^{1}H NMR (600 MHz, CDCl₃) δ 7.70 (d, *J* = 1.8 Hz, 1H), 7.28 (dd, *J* = 8.6, 1.9 Hz, 1H), 7.14 (d, *J* = 8.6 Hz, 1H), 6.82 (s, 1H), 5.86 (ddt, *J* = 16.9, 10.2, 6.6 Hz, 1H), 5.05 (ddd, *J* = 17.1, 3.5, 1.6 Hz, 1H), 4.99 (ddd, *J* = 10.2, 2.0, 1.1 Hz, 1H), 3.72 (s, 3H), 2.70 (t, *J* = 7.6 Hz, 2H), 2.14 (dd, *J* = 14.5, 7.1 Hz, 2H), 1.80 – 1.74 (m, 2H).

^{13}C NMR (151 MHz, CDCl₃) δ 138.8, 135.8, 129.8, 127.4, 124.3, 121.8, 115.0, 114.9, 112.1, 110.7, 33.7, 32.9, 29.6, 24.4.

HRMS (ESI-TOF) m/z: $[M + H]^+$ Calcd for $C_{14}H_{17}{}^{79}BrN$ 278.0539; Found 278.0539.

IR (neat) vmax: 2928, 1639, 1461, 1342, 1267, 1225, 1134, 1094, 1054.

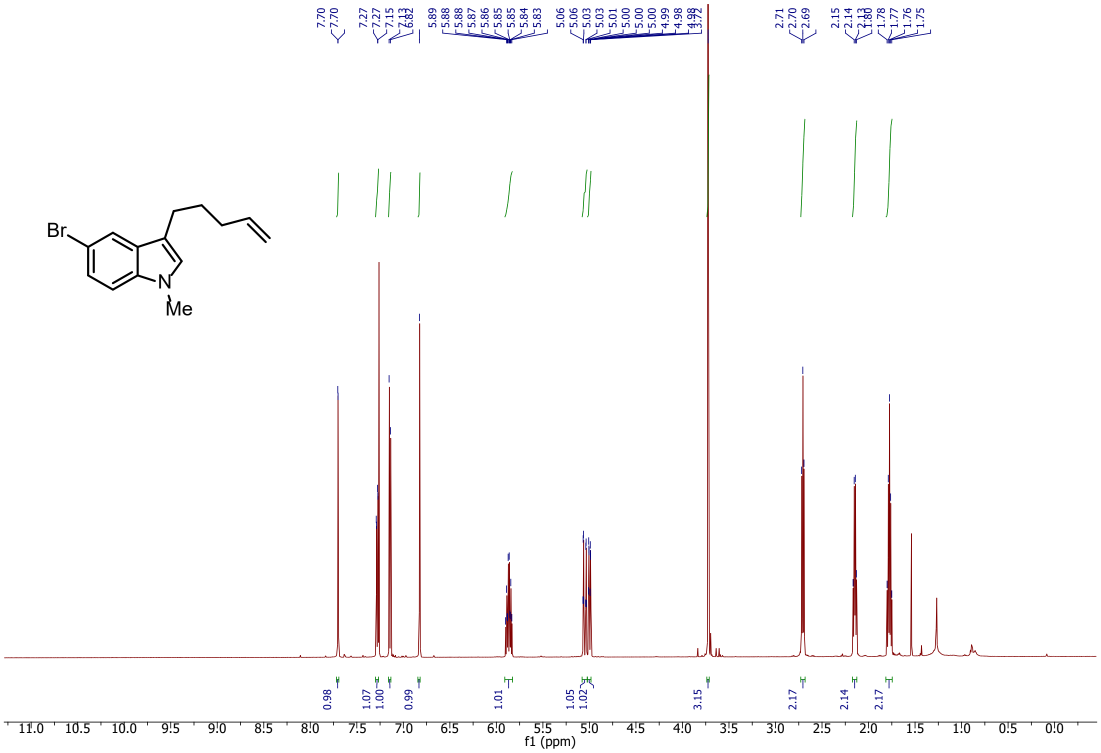

Figure 8: ^{1}H NMR (600 MHz, CDCl₃) of SM-1^{Br}-2

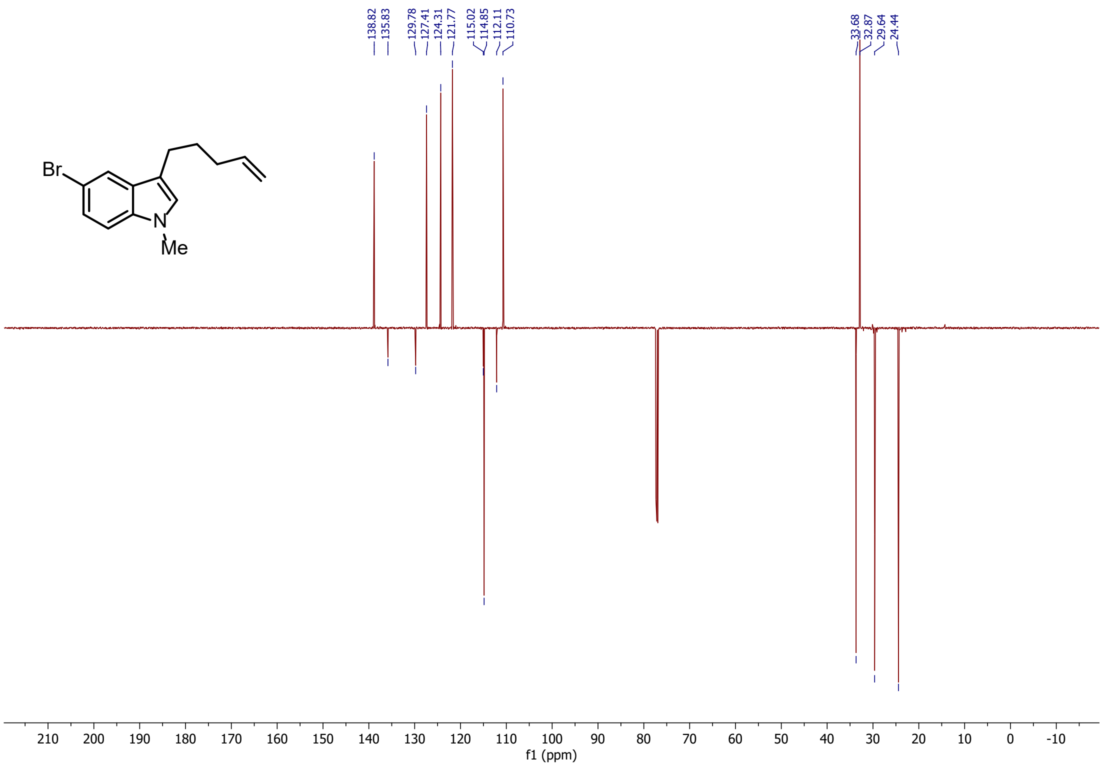

Figure 9: ***^{13}C NMR (151 MHz, $CDCl_3$) of SM-1^{Br}-2.***

A 25 mL flame-dried Schlenk flask equipped with a stirring bar was charged with **SM-1^{Br}-2** (258 mg, 0.93 mmol, 1.00 eq.) and DMF (6.0 mL), before cooling to 0 °C. Phosphorus(V) oxychloride (0.13 mL, 1.39 mmol, 1.50 eq.) was added dropwise and the resulting mixture heated to 100 °C and stirred at the same temperature for 2 h. The crude was then cooled to 0 °C before the addition of an aq. solution of NaOH (2 M, 2 mL) and stirring for 15 min at 23 °C. Work up was performed by dilution in EtOAc (10 mL) and H_2O (10 mL). The phases were separated and the aqueous phase was extracted twice with EtOAc (20 mL). The organic phases were combined, dried over $MgSO_4$ and filtered . The solution was concentrated under reduced pressure and the crude mixture was purified by flash chromatography using a gradient of heptanes and a mixture of heptane:toluene:MTBE (85:10:5) to yield **SM-1^{Br}-3** as an off-white semi-solid (55 mg, 0.18 mmol, 19% yield).

^{1}H NMR (600 MHz, $CDCl_3$) δ 10.12 (s, 1H), 7.83 (d, *J* = 1.5 Hz, 1H), 7.48 (dd, *J* = 8.9, 1.9 Hz, 1H), 7.23 (d, *J* = 8.8 Hz, 1H), 5.83 (ddt, *J* = 16.9, 10.2, 6.6 Hz, 1H), 5.08 – 5.01 (m, 2H), 4.03 (s, 3H), 3.06 – 3.02 (m, 2H), 2.14 (dd, *J* = 14.4, 6.9 Hz, 2H), 1.81 (dd, *J* = 15.1, 7.6 Hz, 2H).

^{13}C NMR (151 MHz, $CDCl_3$) δ 181.8, 138.5, 138.0, 130.2 (2C), 127.9, 123.9, 115.6 (2C), 113.5, 112.1, 33.5, 32.0, 31.4, 23.1.

HRMS (ESI-TOF) m/z: $[M + Na]^+$ Calcd for $C_{15}H_{16}{}^{79}BrNO^+Na$ 328.0307; Found 328.0305.

IR (neat) νmax: 2931, 1711, 1661, 1474, 1412, 1366, 1269, 1220, 1109, 1056.

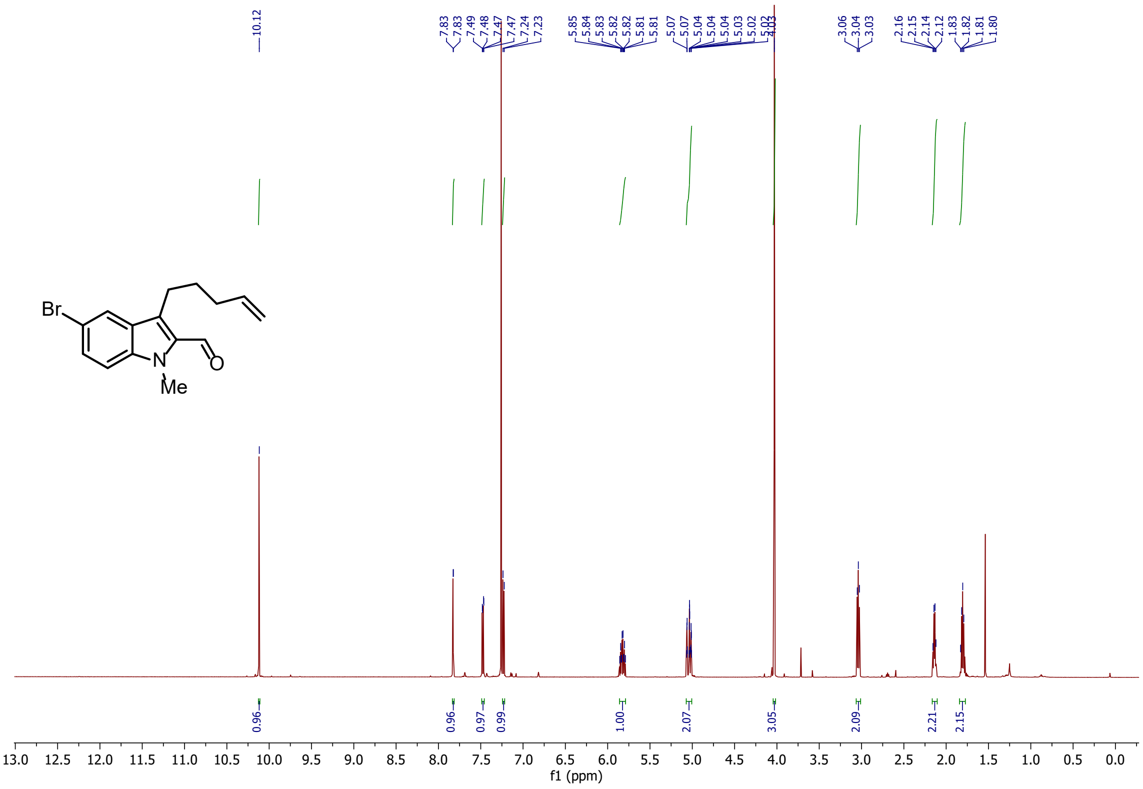

Figure 10: ***^{1}H NMR (600 MHz, CDCl3) of SM-1^{Br}-3***

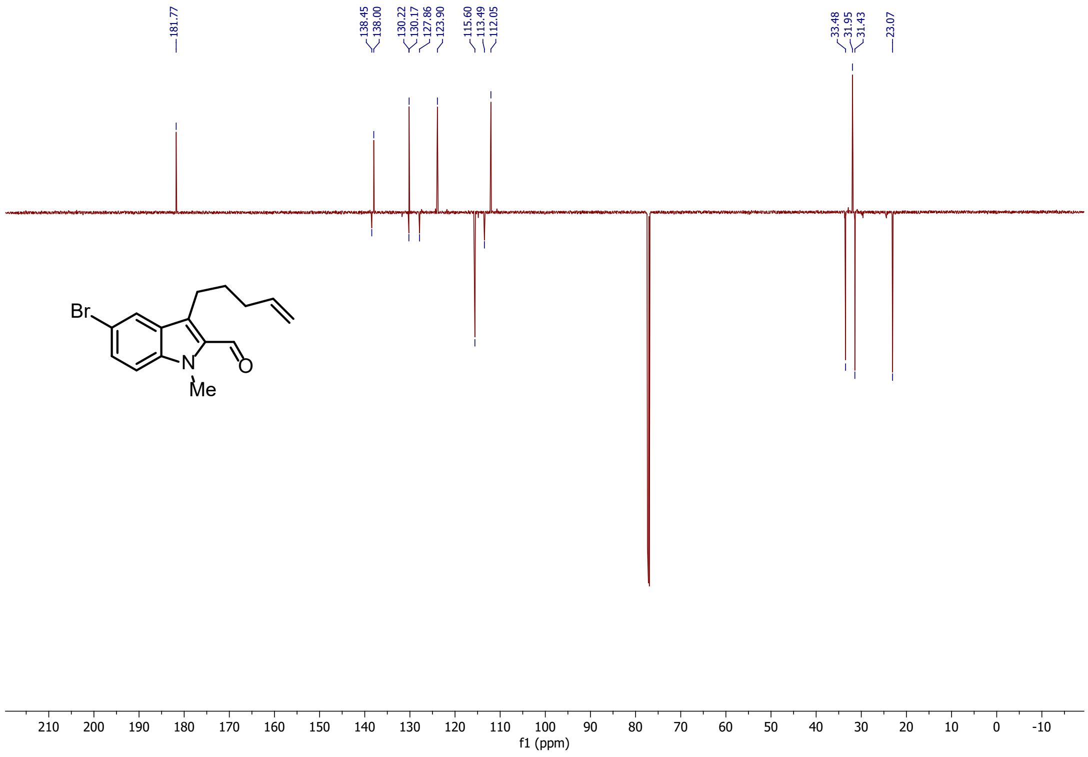

Figure 11: ***^{13}C NMR (150 MHz, CDCl3) of SM-1^{Br}-3***

Methyl (*E*)-6-(5-bromo-2-formyl-1-methyl-1*H*-indol-3-yl)hex-2-enoate (1^{Br})

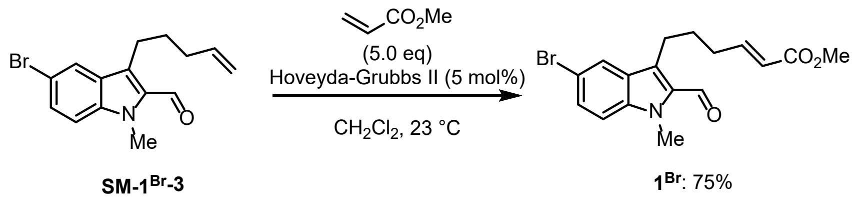

A Schlenk flask was charged with **SM-1^{Br}-3** (55 mg, 0.18 mmol, 1.0 eq.), CH_2Cl_2 (1.0 mL), methyl acrylate (81 µL, 0.90 mmol, 5.0 eq.) and Hoveyda-Grubbs 2nd generation catalyst (5.6 mg, 0.0090 mmol, 5 mol%). The mixture was stirred for 3 h at 23 °C, before being concentrated under reduced pressure. The crude mixture was purified by flash chromatography using a gradient of heptanes/EtOAc to yield **1^{Br}** as a light-yellow solid (49 mg, 0.14 mmol, 75% yield).

^{1}H NMR (700 MHz, $CDCl_3$) δ 10.11 (s, 1H), 7.81 – 7.79 (m, 1H), 7.48 (dd, *J* = 8.9, 1.9 Hz, 1H), 7.24 (d, *J* = 8.8 Hz, 1H), 6.95 (dt, *J* = 15.6, 6.9 Hz, 1H), 5.83 (dt, *J* = 15.6, 1.5 Hz, 1H), 4.03 (s, 3H), 3.73 (s, 3H), 3.06 (t, *J* = 7.6 Hz, 2H), 2.30 – 2.26 (m, 2H), 1.90 – 1.86 (m, 2H).

^{13}C NMR (176 MHz, $CDCl_3$) δ 181.5, 167.0, 148.3, 138.4, 131.8, 130.3, 129.1, 127.7, 123.7, 121.8, 113.7, 112.1 (2C), 51.6, 31.9, 30.5, 23.2.

HRMS (ESI-TOF) m/z: $[M + Na]^+$ Calcd for $C_{17}H_{18}{}^{79}BrNO_3Na$ 386.0362; Found 386.0362.

IR (neat) νmax: 2946, 1717, 1656, 1526, 1472, 1382, 1326, 1268, 1202, 1147, 1095, 1029.

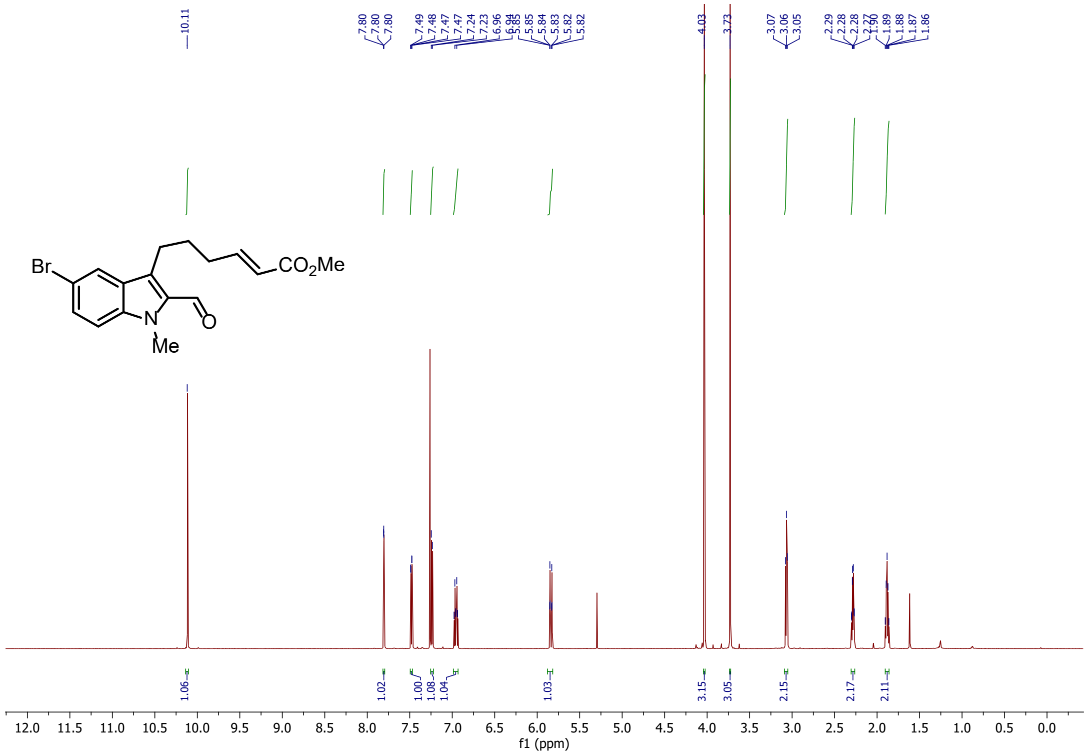

Figure 12: ***^{1}H NMR (700 MHz, CDCl$_3$) of 1^{Br}.***

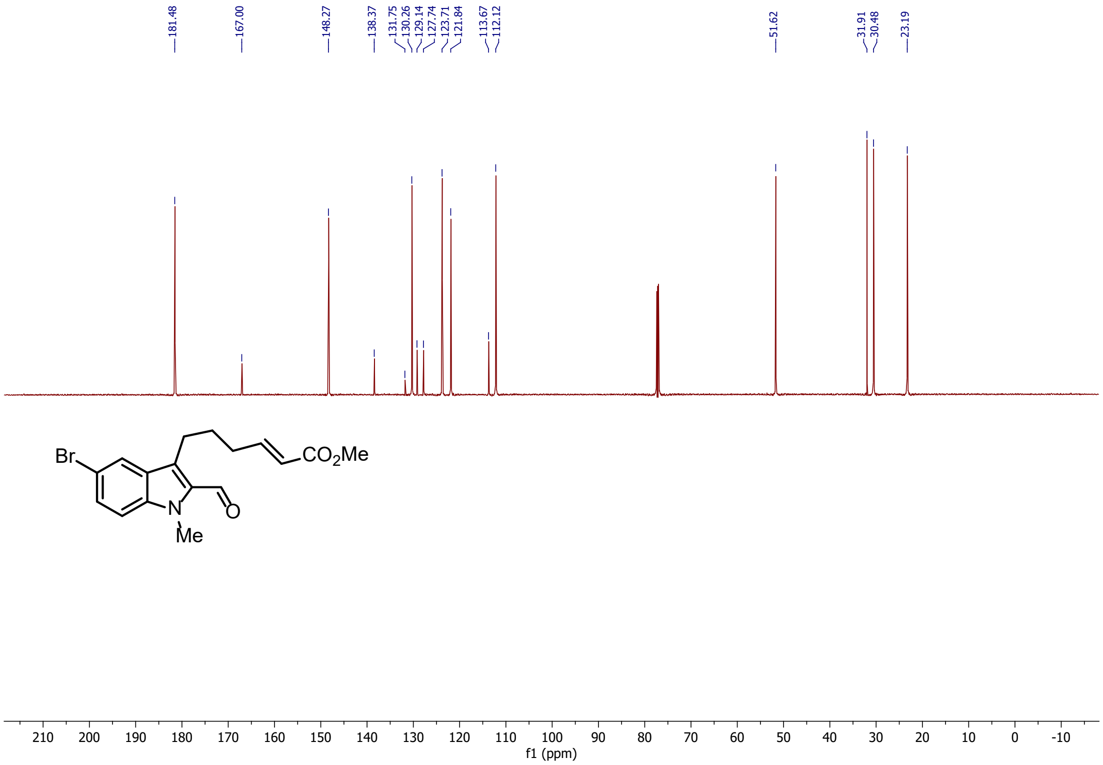

Figure 13: ***^{13}C NMR (176 MHz, CDCl$_3$) of 1^{Br}.***

c. Synthesis of methyl (*E*)-6-(2-formyl-5-methoxy-1-methyl-1*H*-indol-3-yl)hex-2-enoate (1^{OMe})

5-methoxy-3-(pent-4-en-1-yl)-1*H*-indole (SM-1^{OMe}-1)

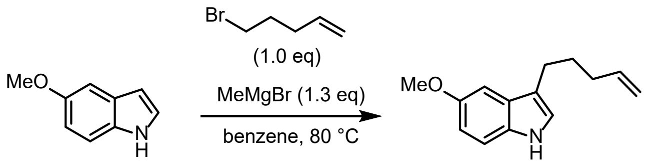

SM-1^{OMe}-1: 49%

Benzene was dried over 3Å molsieves before use. A 50 mL Schlenk flask was charged with 5-methoxyindole (1.00 g, 6.73 mmol, 1.50 eq.) and dried benzene (15 mL). Methylmagnesium bromide (3.0 M in Et_2O, 3.09 mL, 8.97 mmol, 2.00 eq.) was added dropwise and the mixture was stirred for 10 min at 25 °C. 5-Bromo-1-pentene (800 µL, 6.73 mmol, 1.50 eq.) was added and the reaction was refluxed for 20 h. After returning to ambient temperature, the work-up was performed by addition of a sat. aq. solution of NH_4Cl (20 mL) and extraction with EtOAc (50 mL). The organic layer was washed with brine (50 mL), dried over $MgSO_4$, filtered and concentrated under reduced pressure. Purification was attempted by flash chromatography using a gradient of heptanes/EtOAc to yield **SM-1^{OMe}-1** as a dark-yellow solid (710 mg, 3.30 mmol, 49% yield). The analytical data is in accordance to previous report.[3]

^{1}H NMR (400 MHz, $CDCl_3$) δ 7.82 (br s, 1H), 7.28 (d, *J* = 1.9 Hz, 1H), 7.08 (d, *J* = 2.3 Hz, 1H), 6.99 (d, *J* = 2.0 Hz, 1H), 6.89 (dd, *J* = 8.8, 2.4 Hz, 1H), 5.92 (ddt, *J* = 16.9, 10.2, 6.6 Hz, 1H), 5.09 (dd, *J* = 17.2, 1.6 Hz, 2H), 5.03 (dd, *J* = 10.2, 0.9 Hz, 1H), 3.91 (s, 3H), 2.78 (t, *J* = 7.6 Hz, 2H), 2.20 (dd, *J* = 14.4, 7.1 Hz, 2H), 1.89 – 1.81 (m, 2H).

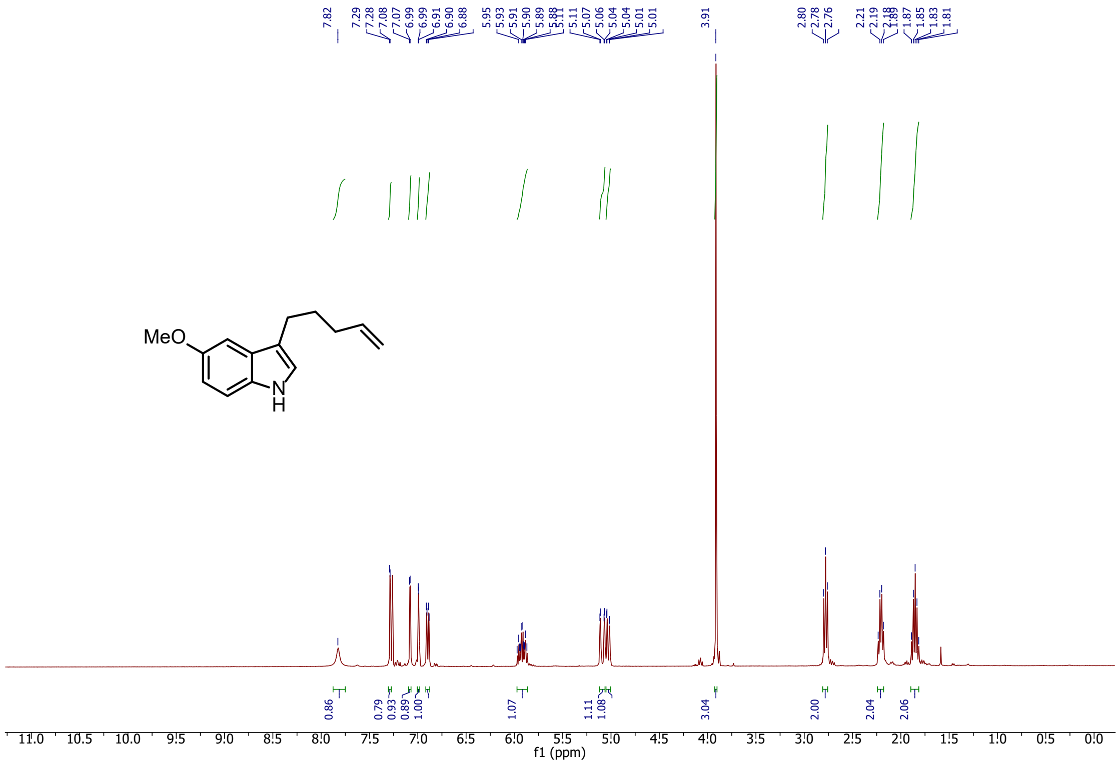

Figure 14: ***^{1}H NMR (400 MHz, $CDCl_3$) of SM-1^{OMe}-1.***

5-methoxy-1-methyl-3-(pent-4-en-1-yl)-1*H*-indole (SM-1^{OMe}-2)

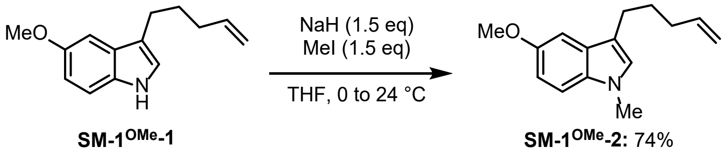

A 25-mL Schlenk flask was charged with NaH (60% in paraffine, 189 mg, 4.70 mmol, 1.50 eq.), THF (9 mL), and cooled to 0 °C. **SM-1^{OMe}-1** (677 mg, 3.14 mmol, 1.00 eq.) was added dropwise as a solution in THF (3 mL). The resulting mixture was stirred for 30 min at 0 °C, before the addition of iodomethane (294 µL, 4.70 mmol, 1.50 eq.) at the same temperature. The resulting mixture was stirred for 3 h at 24 °C. Work up was performed by dilution with EtOAc (10 mL) and a sat. aq. solution of NH_4Cl (10 mL). The phases were separated and the aqueous phase was extracted twice with EtOAc (10 mL). The organic phases were combined, dried over $MgSO_4$ and filtered through a cotton pad. The solution was concentrated under reduced pressure and the crude mixture was purified by flash chromatography using a gradient of heptanes/EtOAc to yield **SM-1^{OMe}-2** as a light-yellow solid (534 mg, 2.33 mmol, 74% yield).

^{1}H NMR (700 MHz, $CDCl_3$) δ 7.18 (d, *J* = 8.8 Hz, 1H), 7.03 (d, *J* = 2.4 Hz, 1H), 6.89 (dd, *J* = 8.8, 2.4 Hz, 1H), 6.81 (s, 1H), 5.88 (ddt, *J* = 16.9, 10.2, 6.6 Hz, 1H), 5.07 – 5.04 (m, 1H), 4.99 (ddt, *J* = 10.2, 2.2, 1.2 Hz, 1H), 3.88 (s, 3H), 3.72 (s, 3H), 2.73 (t, *J* = 7.5 Hz, 2H), 2.19 – 2.15 (m, 2H), 1.82 – 1.78 (m, 2H).

^{13}C NMR (176 MHz, $CDCl_3$) δ 153.7, 139.1, 132.7, 128.3, 126.9, 114.7(2C), 111.7, 109.9, 101.3, 56.2, 33.7, 32.9, 29.6, 24.6.

HRMS (ESI-TOF) m/z: $[M + Na]^+$ Calcd for $C_{15}H_{19}NONa$ 252.1359; Found 252.1354.

IR (neat) vmax: 2928, 1639, 1578, 1490, 1454, 1355, 1277, 1250, 1174, 1150, 1121.

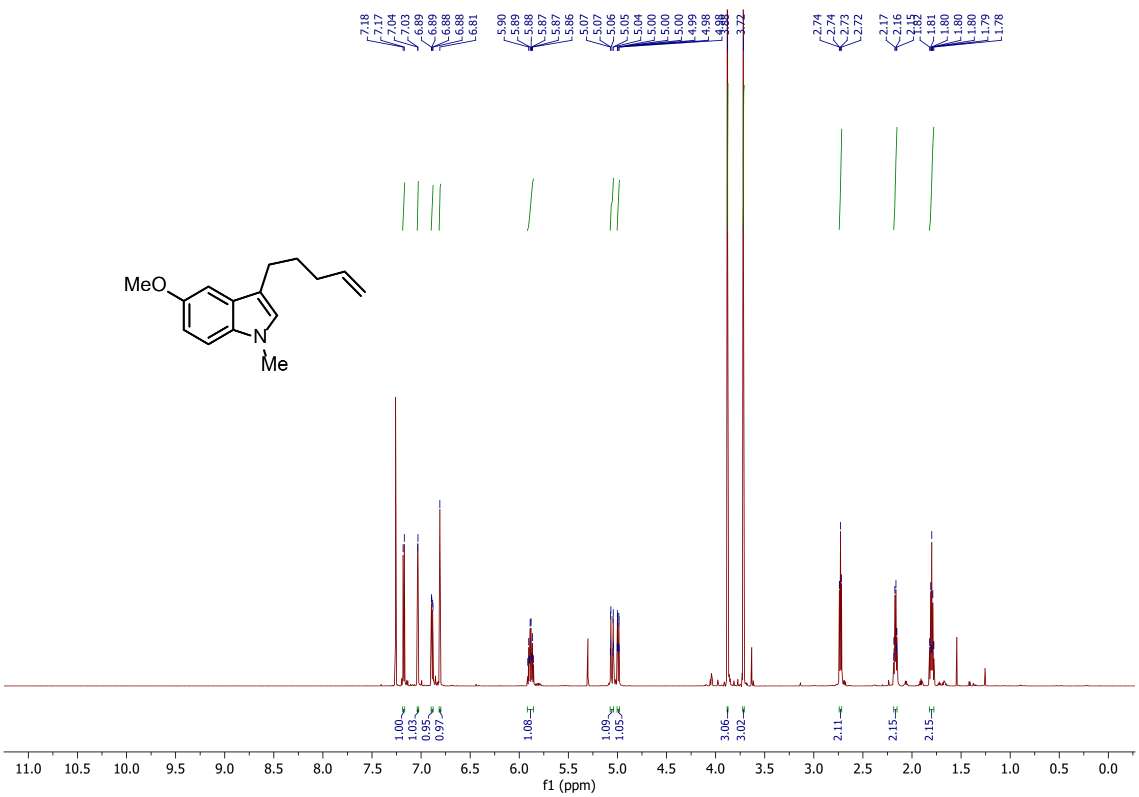

Figure 15: ¹H NMR (700 MHz, CDCl₃) of SM-1ᴼᴹᵉ-2.

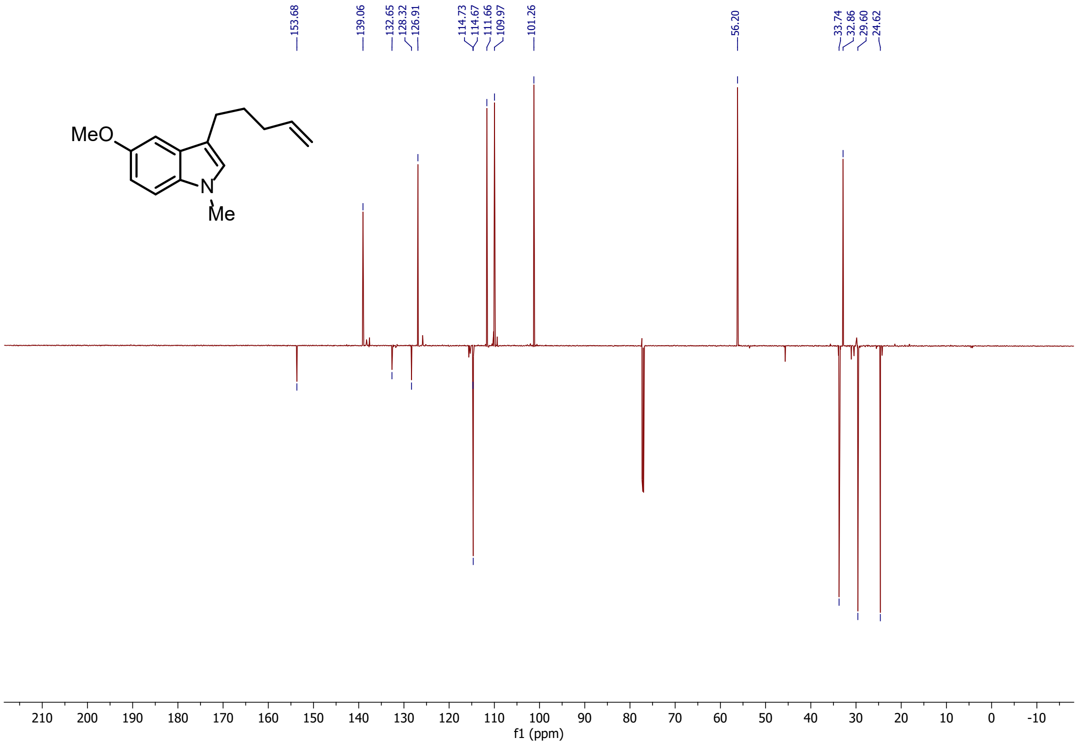

Figure 16: ¹³C NMR (176 MHz, CDCl₃) of SM-1ᴼᴹᵉ-2.

5-methoxy-1-methyl-3-(pent-4-en-1-yl)-1*H*-indole-2-carbaldehyde (SM-1^{OMe}-3)

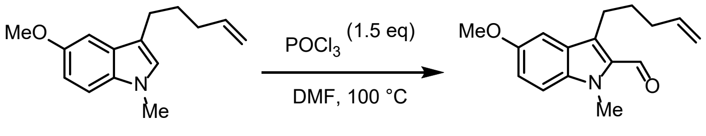

A 50 mL Schlenk flask was charged with **SM-1^{OMe}-2** (524 mg, 2.28 mmol, 1.00 eq.) and DMF (13 mL), before cooling to 0 °C. Phosphorus(V) oxychloride (139 µL, 3.30 mmol, 1.50 eq.) was added dropwise and the resulting mixture heated to 100 °C and stirred at the same temperature for 2 h. The crude was then cooled to 0 °C before the addition of an aq. solution of NaOH (2 M, 2 mL) and stirring for 15 min at 23 °C. Work-up was performed by dilution in EtOAc (20 mL) and H_2O (20 mL). The phases were separated and the aqueous phase was extracted twice with EtOAc (20 mL). The organic phases were combined, dried over $MgSO_4$ and filtered. The solution was concentrated under reduced pressure and the crude mixture was purified by flash chromatography using a gradient of heptanes/EtOAc to yield **SM-1^{OMe}-3** as a light-yellow solid (308 mg, 1.20 mmol, 52% yield).

^{1}H NMR (700 MHz, $CDCl_3$) δ 10.10 (s, 1H), 7.27 (s, 1H), 7.12 (dd, *J* = 9.0, 2.4 Hz, 1H), 7.04 (d, *J* = 2.4 Hz, 1H), 5.86 (ddt, *J* = 16.9, 10.2, 6.6 Hz, 1H), 5.07 (ddd, *J* = 17.1, 3.5, 1.6 Hz, 1H), 5.04 – 5.02 (m, 1H), 4.04 (s, 3H), 3.89 (s, 3H), 3.08 – 3.06 (m, 2H), 2.18 – 2.15 (m, 2H), 1.86 – 1.81 (m, 2H).

^{13}C NMR (176 MHz, $CDCl_3$) δ 181.5, 154.5, 138.3, 135.8, 130.3, 126.4, 119.3, 115.4, 111.5, 101.1, 56.0, 33.5, 31.9, 31.3, 23.2. *Two quaternary carbons overlap.*

HRMS (ESI-TOF) m/z: $[M + Na]^+$ Calcd for $C_{16}H_{19}NO_2Na$ 280.1308; Found 280.1302.

IR (neat) νmax: 2935, 1654, 1525, 1492, 1457, 1386, 1307, 1195, 1166, 1117, 1040.

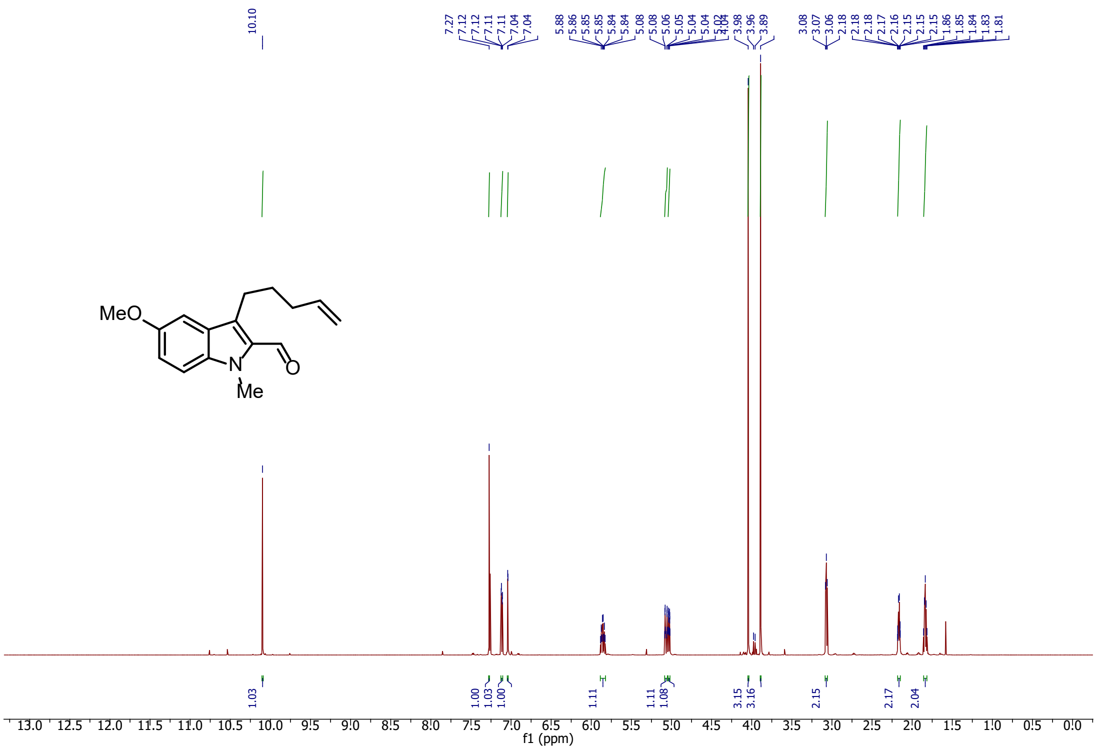

Figure 17: ***^{1}H NMR (700 MHz, $CDCl_3$) of SM-1^{OMe}-3.***

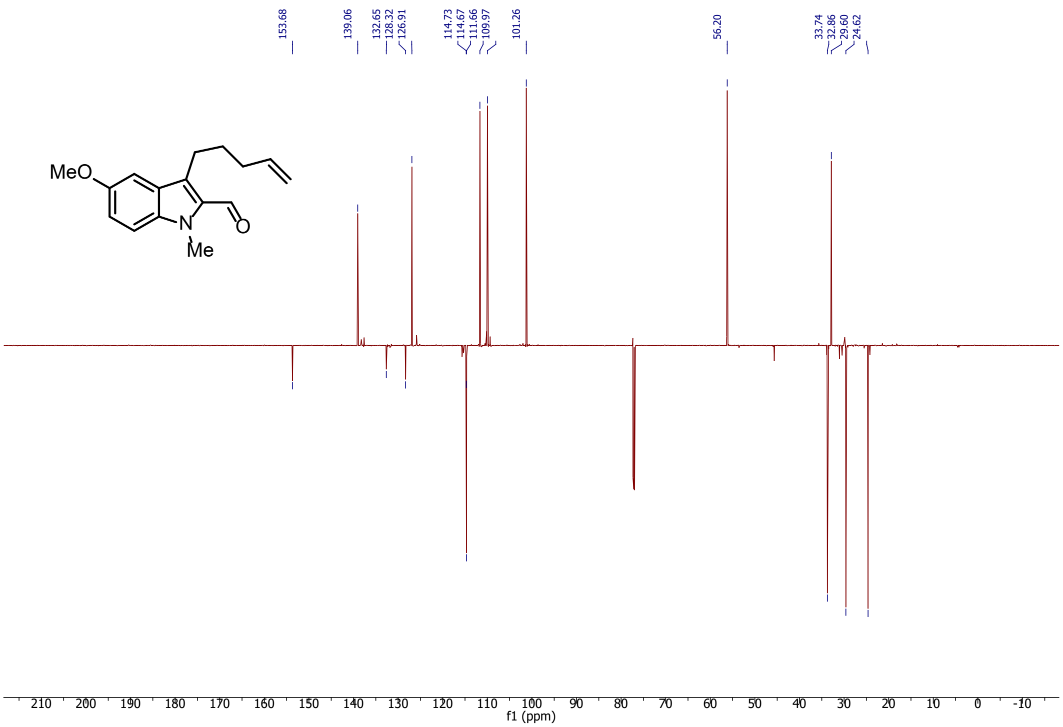

Figure 18: ***^{13}C NMR (176 MHz, $CDCl_3$) of SM-1^{OMe}-3.***

Methyl (*E*)-6-(2-formyl-5-methoxy-1-methyl-1*H*-indol-3-yl)hex-2-enoate (1^{OMe})

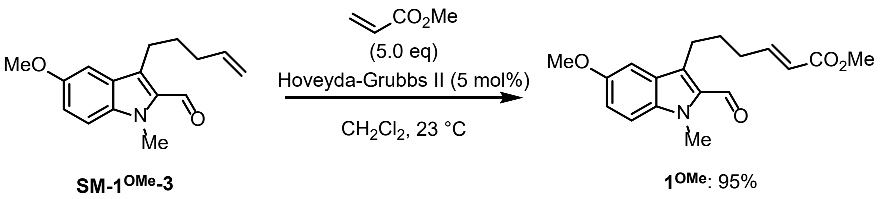

A Schlenk flask was charged with **SM-1^{OMe}-3** (290 mg, 1.13 mmol, 1.00 eq.), CH_2Cl_2 (7.0 mL), methyl acrylate (507 µL, 5.63 mmol, 5.0 eq.) and Hoveyda-Grubbs 2nd generation catalyst (35 mg, 0.056 mmol, 5 mol%). The mixture was stirred for 3 h at 23 °C, before being concentrated under reduced pressure. The crude mixture was purified by flash chromatography using a gradient of heptanes/EtOAc to yield **1^{OMe}** as a light-yellow solid (339 mg, 1.07 mmol, 95% yield).

^{1}H NMR (600 MHz, $CDCl_3$) δ 10.08 (s, 1H), 7.27 – 7.25 (m, 1H), 7.10 (dd, *J* = 9.1, 2.4 Hz, 1H), 4.03 (s, 3H), 3.87 (s, 3H), 3.72 (s, 3H), 3.08 (t, *J* = 7.5 Hz, 2H), 2.31 – 2.26 (m, 2H), 1.92 – 1.87 (m, 2H). *One aromatic proton overlaps with the solvent peak.*

^{13}C NMR (151 MHz, $CDCl_3$) δ 181.3, 167.1, 154.6, 148.7, 135.7, 131.5, 129.2, 126.3, 121.7, 119.4, 111.6, 100.8, 55.9, 51.6, 31.8, 31.8, 30.4, 23.2.

HRMS (ESI-TOF) m/z: $[M + Na]^+$ Calcd for $C_{18}H_{21}NO_4Na$ 338.1363; Found 338.1363.

IR (neat) νmax: 2945, 1719, 1654, 1524, 1493, 1437, 1417, 1387, 1310, 1271, 1196, 1149, 1117.

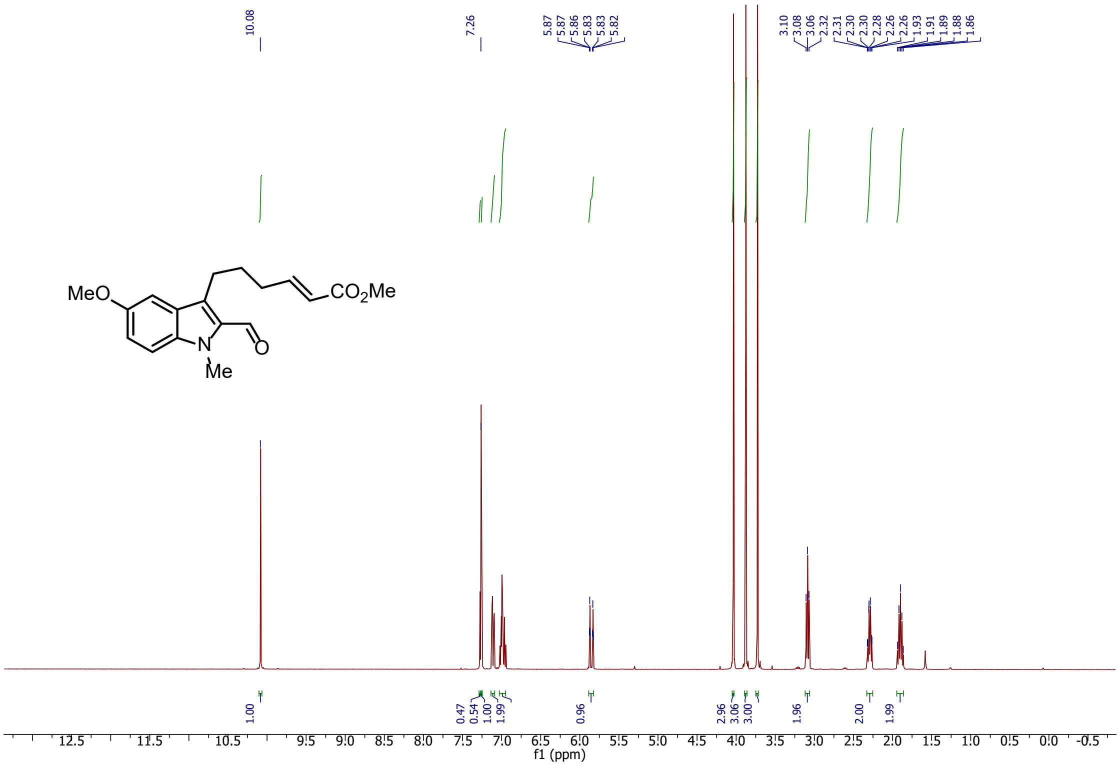

Figure 19: ^{1}H NMR (600 MHz, CDCl3) of 1[OMe].

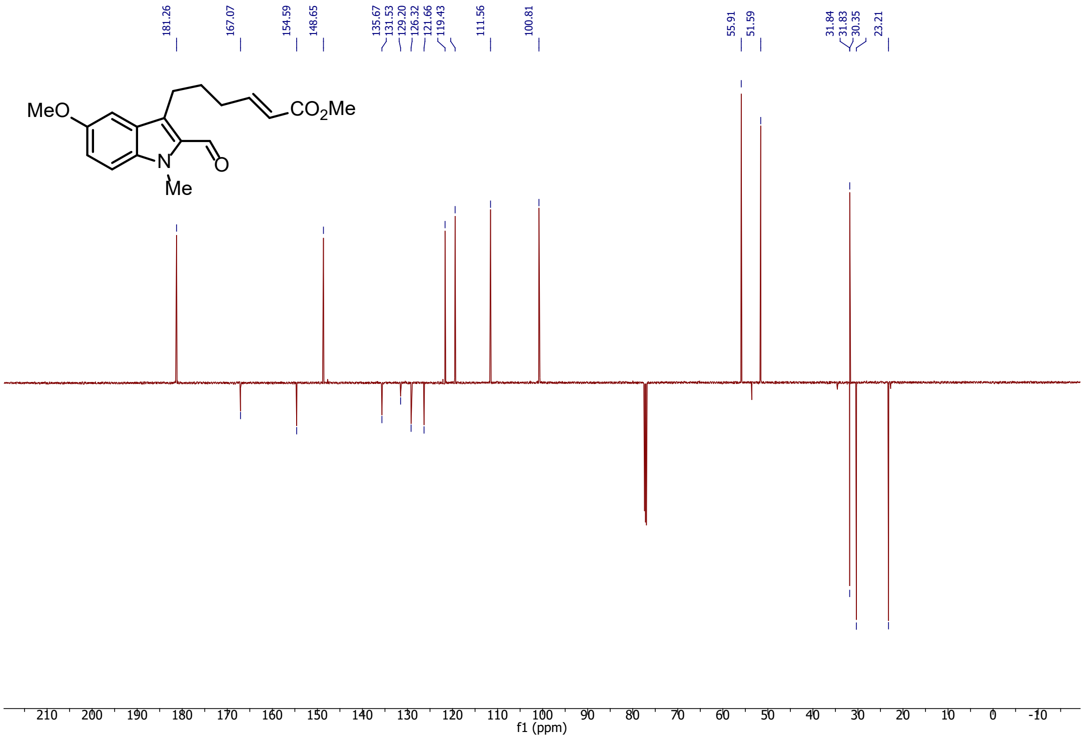

Figure 20: ^{13}C NMR (151 MHz, CDCl3) of 1[OMe].

d. Synthesis of methyl (*E*)-4-((N-((2-formyl-1*H*-indol-3-yl)methyl)-4-methylphenyl)sulfonamido)but-2-enoate (4a)

Ethyl 3-formyl-1*H*-indole-2-carboxylate (SM-4a-1)

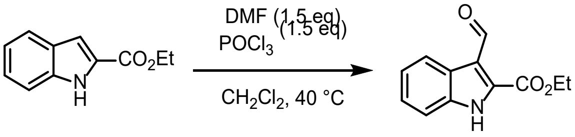

SM-4a-1

SM-4a-1 was synthesized according to a reported procedure.[4]

^{1}H NMR (400 MHz, CDCl$_3$) δ 10.79 (s, 1H), 8.51 (d, *J* = 8.1 Hz, 1H), 7.50 (d, *J* = 8.2 Hz, 1H), 7.47 – 7.41 (m, 1H), 7.38 (ddd, *J* = 8.1, 6.9, 1.3 Hz, 1H), 4.56 (q, *J* = 7.1 Hz, 2H), 1.51 (t, *J* = 7.1 Hz, 3H).

The analytical data is in accordance to previous report.[4]

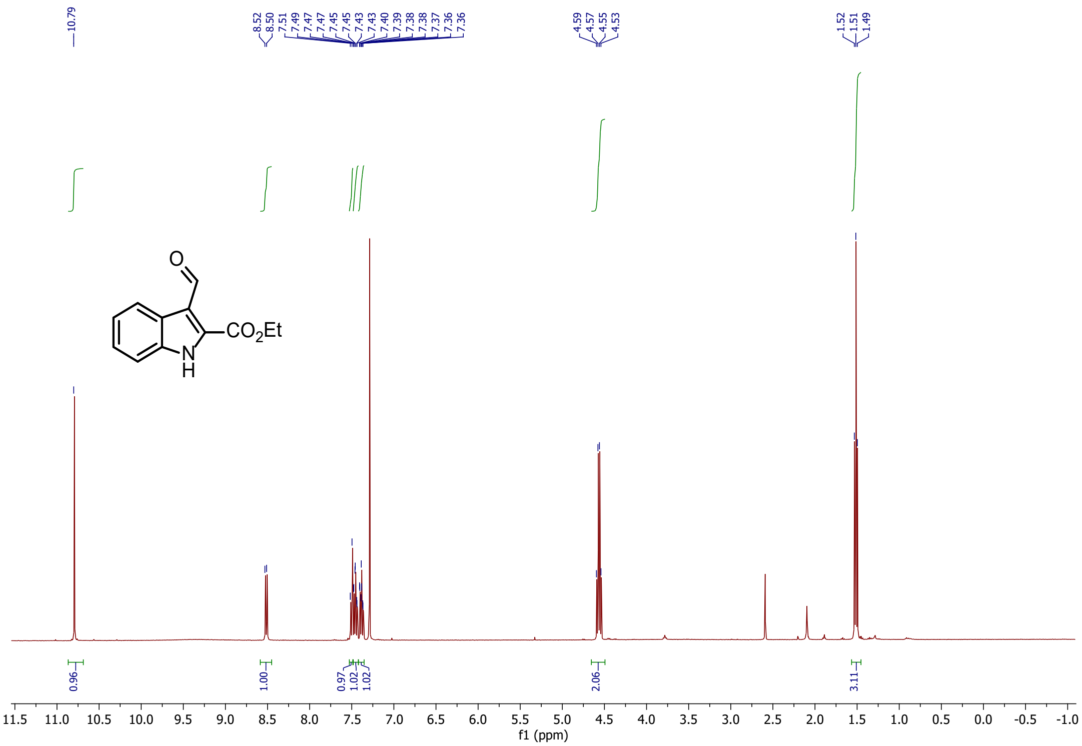

*Figure 21: ^{1}**H NMR (400 MHz, CDCl$_3$) of SM-4a-1.***

Ethyl 3-(((*N*-allyl-4-methylphenyl)sulfonamido)methyl)-1*H*-indole-2-carboxylate (SM-4a-3)

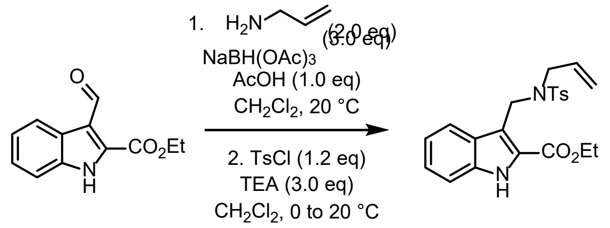

SM-4a-1 **SM-4a-3**: 94% (2 steps)

In a 250 mL round-bottomed flask, **SM-4a-1** (2.17 g, 10.0 mmol, 1.00 eq.) was dissolved in CH_2Cl_2 (50 mL) followed by the addition of allylamine (1.53 mL, 20.0 mmol, 2.00 eq.), $Na(OAc)_3BH$ (6.36 g, 20.0 mmol, 3.00 eq.) and AcOH (0.57 mL, 10 mmol, 1.0 eq.). The mixture was stirred for 20 h at 20 °C, before being poured into a sat. aq. solution of Na_2CO_3 (50 mL) and diluted with CH_2Cl_2 (50 mL). The phases were separated and the aqueous phase was extracted twice more with CH_2Cl_2 (20 mL). The organic phases were combined, dried over Na_2SO_4 and filtered. The solution was concentrated under reduced pressure to yield **SM-4a-2** as a yellow oil, which was used as such in the next step.

In a 250 mL round-bottomed flask, **SM-4a-2** (assumed pure; 2.58 g, 10.0 mmol, 1.00 eq.) was dissolved in CH_2Cl_2 (60 mL). The solution was cooled to 0 °C, followed by the addition of Et_3N (4.18 mL, 30.0 mmol, 3.00 eq.) and *p*-TsCl (2.29 g, 12.0 mmol, 1.20 eq.). The mixture was stirred for 1 h at 20 °C, before being diluted with CH_2Cl_2 (50 mL) and a sat. aq. solution of NH_4Cl (50 mL). The phases were separated and the aqueous phase was extracted twice more with CH_2Cl_2 (25 mL). The organic phases were combined, dried over Na_2SO_4 and filtered . The solution was concentrated under reduced pressure and the crude mixture was purified by flash chromatography using a gradient of heptanes/EtOAc to yield **SM-4a-3** as a yellow solid (3.86 g, 9.36 mmol, 94% yield over 2 steps).

^{1}H NMR (700 MHz, $CDCl_3$) δ 8.75 (br s, 1H), 8.11 (dd, *J* = 8.1, 0.8 Hz, 1H), 7.81 – 7.73 (m, 2H), 7.38 – 7.34 (m, 2H), 7.33 (d, *J* = 7.9 Hz, 2H), 7.19 (ddd, *J* = 8.0, 6.4, 1.5 Hz, 1H), 5.39 (ddd, *J* = 12.5, 10.2, 5.1 Hz, 1H), 4.97 (s, 2H), 4.87-4.72 (m, 2H), 4.35 (q, *J* = 7.1 Hz, 2H), 3.72 – 3.64 (m, 2H), 2.45 (s, 3H), 1.34 (t, *J* = 7.1 Hz, 3H).

^{13}C NMR (176 MHz, $CDCl_3$) δ 161.8, 143.4, 136.8, 135.8, 133.3, 129.8 (2C), 127.9, 127.6 (2C), 126.2, 124.9, 122.5, 121.2, 117.6, 117.3, 111.6, 61.2, 50.2, 42.6, 21.7, 14.5.

HRMS (ESI-TOF) m/z: $[M + Na]^+$ Calcd for $C_{22}H_{24}N_2O_4SNa$ 435.1349; Found 435.1347.

IR (neat) vmax: 3351, 3062, 2981, 2925, 1707, 1377, 1326, 1245, 1196, 1158, 1134, 1091, 747, 706, 664, 614, 548.

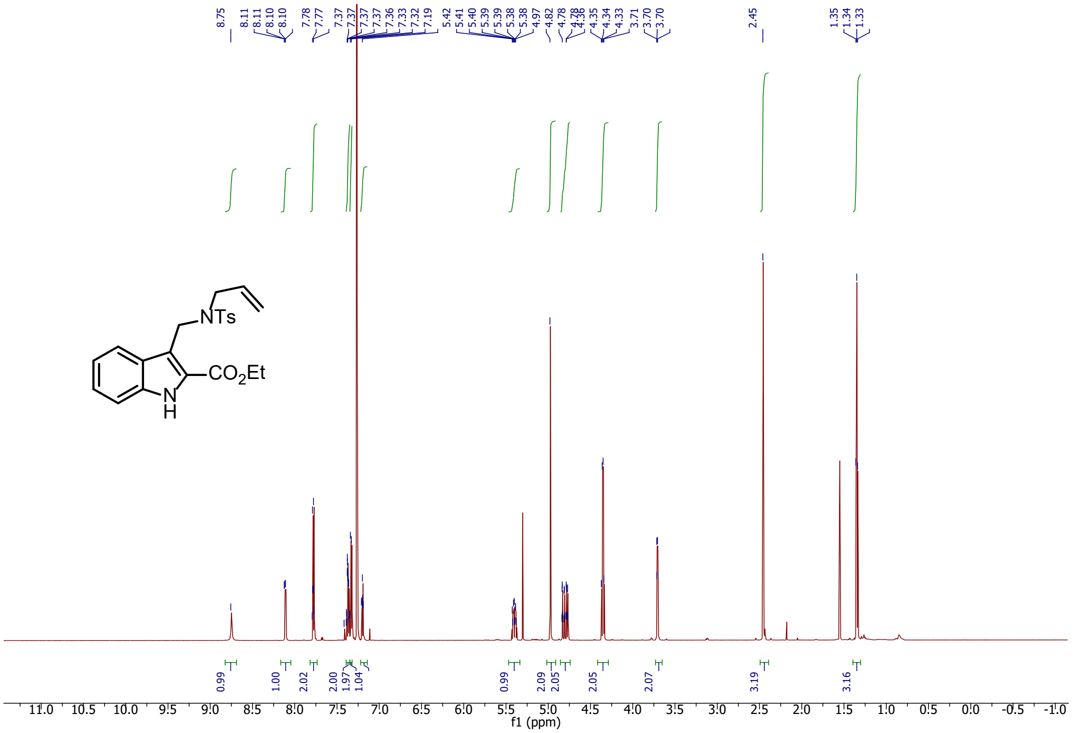

Figure 22: ***^{1}H NMR (700 MHz, $CDCl_3$) of SM-4a-3.***

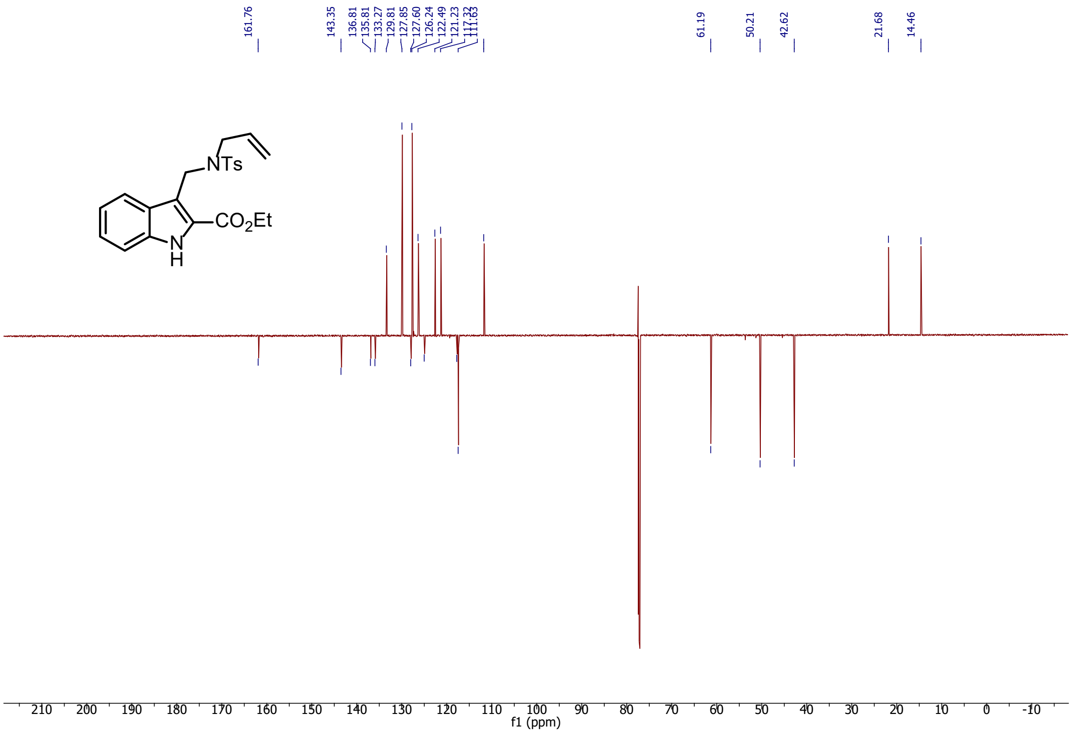

Figure 23: ***^{13}C NMR (176 MHz, $CDCl_3$) of SM-4a-3.***

N-allyl-*N*-((2-formyl-1H-indol-3-yl)methyl)-4-methylbenzenesulfonamide (SM-4a-5)

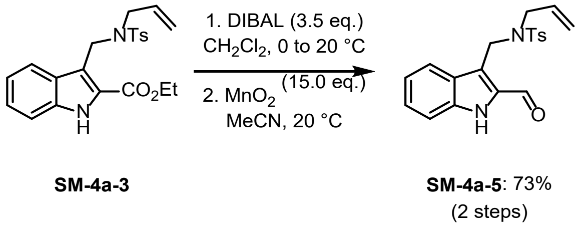

A 500 mL Schlenk flask was charged with **SM-4a-3** (3.42 g, 8.29 mmol, 1.00 eq.), CH_2Cl_2 (137 mL) and the mixture was cooled to −78 °C. DIBAL (1 mol/L in PhMe, 29 mL, 29 mmol, 3.5 eq.) was added dropwise before being stirred for 1 h at −78 °C. After this time, the mixture was allowed to stirred at 0 °C for 10 min followed by the dropwise addition of H_2O (1.2 mL), 1 M NaOH (2.9 mL) and H_2O (1.2 mL). The mixture was allowed to return to ambient temperature and was stirred for 15 min. $MgSO_4$ was added, stirred for 15 min followed by filtration over Celite using CH_2Cl_2. The filtrate was concentrated under reduced pressure to yield **SM-4a-4** as a yellow solid, which was used as such in the next step.

In a 250 mL round-bottomed flask was charged **SM-4a-4** (assumed pure, 3.10 g, 8.29 mmol, 1.00 eq.), acetonitrile (83 mL) followed by MnO_2 (10.8 g, 124 mmol, 15.0 eq.) and $MgSO_4$ (2.0 g). The mixture was stirred at 23 °C for 20 h, before being filtered over Celite, eluted with EtOAc and concentrated under reduced pressure. The crude mixture was purified by flash chromatography using a gradient of heptanes/EtOAc to yield **SM-4a-5** as a yellow solid (2.22 g, 6.02 mmol, 73% over 2 steps).

^{1}H NMR (400 MHz, $CDCl_3$) δ 10.03 (s, 1H), 9.30 (br s, 1H), 7.89 (d, *J* = 8.2 Hz, 1H), 7.82 – 7.70 (m, 2H), 7.46 – 7.36 (m, 2H), 7.33 (d, *J* = 8.3 Hz, 2H), 7.19 – 7.09 (m, 1H), 5.42 (ddt, *J* = 16.7, 10.2, 6.4 Hz, 1H), 4.95 (ddd, *J* = 18.4, 13.6, 1.2 Hz, 2H), 4.85 (s, 2H), 3.78 (d, *J* = 6.4 Hz, 2H), 2.46 (s, 3H).

^{13}C NMR (101 MHz, $CDCl_3$) δ 181.1, 143.8, 137.3, 137.0, 133.3, 132.4, 130.0 (2C), 127.8, 127.4 (2C), 122.3, 121.5, 120.6, 119.5 (2C), 112.5, 49.7, 40.4, 21.7.

HRMS (ESI-TOF) m/z: $[M + Na]^+$ Calcd for $C_{20}H_{20}N_2O_3SNa$ 391.1087; Found 391.1075.

IR (neat) vmax: 3314, 3063, 2923, 2864, 1651, 1328, 1157, 747, 663, 548.

Mp.: 155-157 °C.

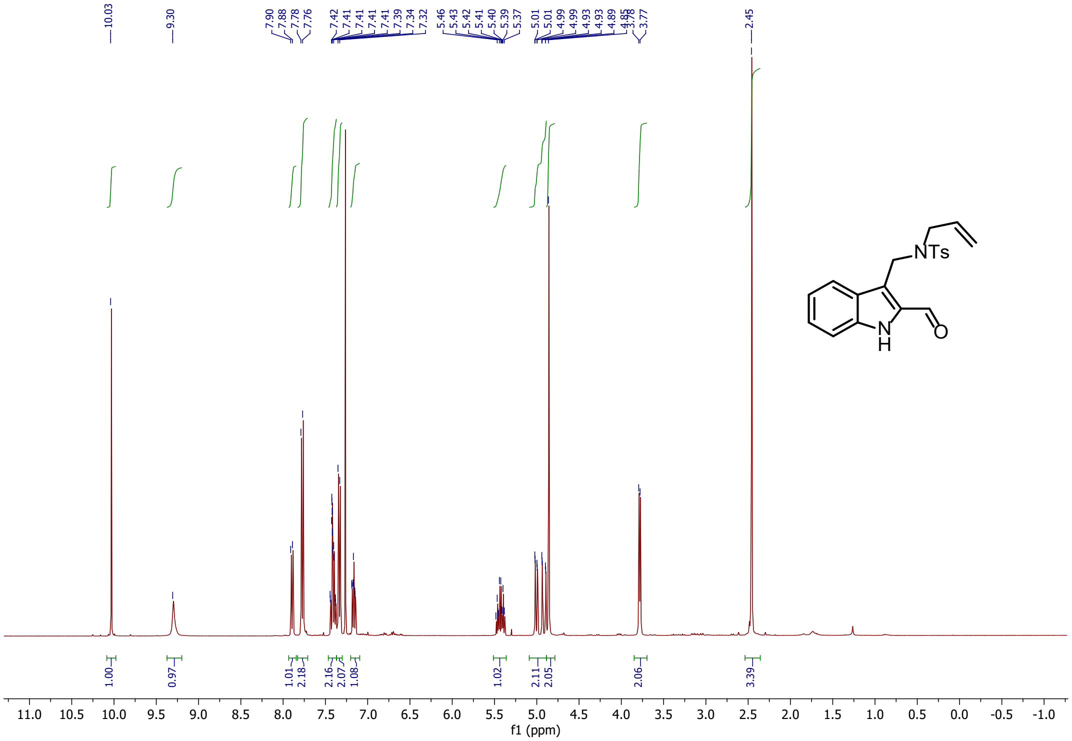

*Figure 24: [1]**H NMR (400 MHz, CDCl₃) of SM-4a-5.***

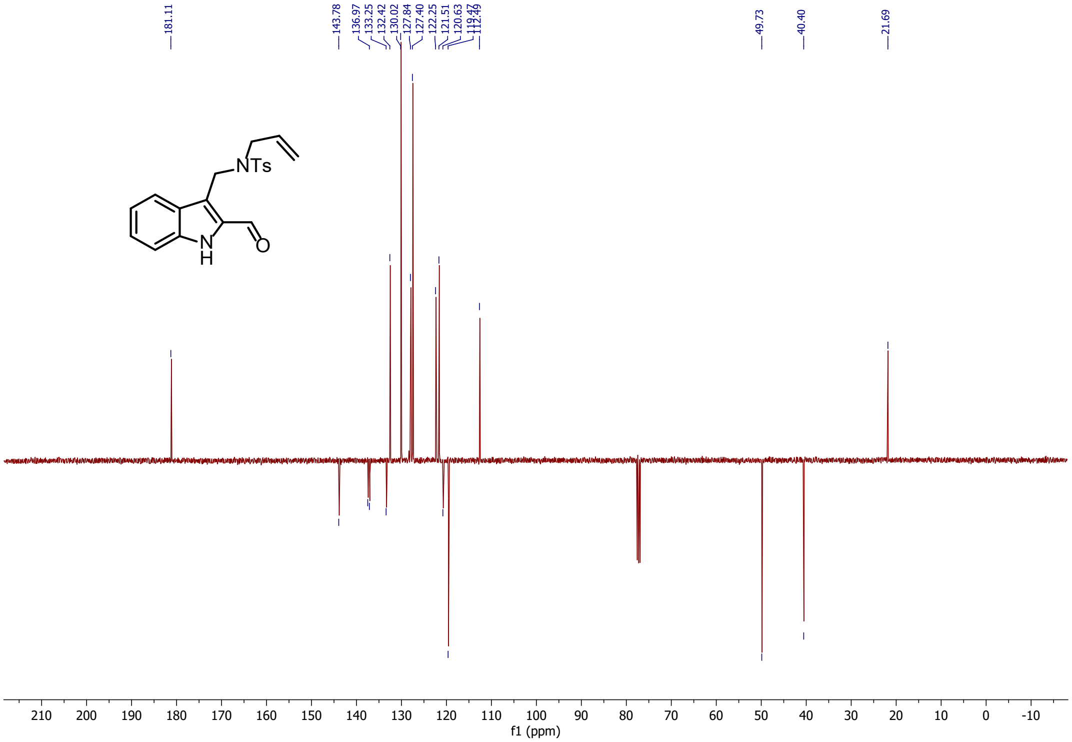

*Figure 25: [13]**C NMR (101 MHz, CDCl₃) of SM-4a-5.***

Methyl (*E*)-4-((*N*-((2-formyl-1H-indol-3-yl)methyl)-4-methylphenyl)sulfonamido)but-2-enoate (4a)

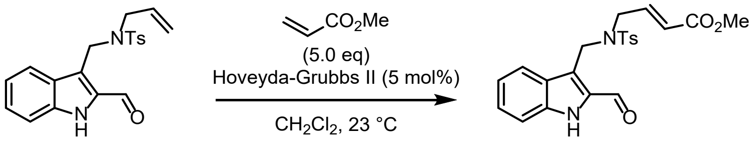

SM-4a-5 **4a**: 63%

A Schlenk flask was charged with **SM-4a-5** (1.62 g, 4.40 mmol, 1.00 eq.), CH_2Cl_2 (44 mL), methyl acrylate (1.80 mL, 22.0 mmol, 5.0 eq.) and Hoveyda-Grubbs 2nd generation catalyst (135 mg, 0.220 mmol, 5 mol%). The mixture was stirred for 20 h at 23 °C, before being concentrated under reduced pressure. The crude mixture was purified twice by flash chromatography using a gradient of heptanes/EtOAc to yield **4a** as a light purple solid (1.17 g, 2.75 mmol, 63% yield).

^{1}H NMR (700 MHz, CDCl$_3$) δ 10.00 (s, 1H), 8.91 (br s, 1H), 7.82 (dd, *J* = 8.2, 0.7 Hz, 1H), 7.79 – 7.69 (m, 2H), 7.42 – 7.37 (m, 2H), 7.35 (d, *J* = 7.9 Hz, 2H), 7.19 (ddd, *J* = 8.0, 6.4, 1.5 Hz, 1H), 6.48 (dt, *J* = 15.7, 6.0 Hz, 1H), 5.59 (dt, *J* = 15.7, 1.6 Hz, 1H), 4.84 (s, 2H), 3.88 (dd, *J* = 6.0, 1.6 Hz, 2H), 3.63 (s, 3H), 2.46 (s, 3H).

^{13}C NMR (176 MHz, CDCl$_3$) δ 180.6, 165.8, 144.3, 142.2, 137.0, 136.2, 133.4, 130.2 (2C), 128.0, 127.5 (2C), 123.5, 121.9, 121.9, 119.7, 112.5, 51.8, 48.1, 41.3, 21.7. *One quaternary carbon could not be found under these conditions.*

HRMS (ESI-TOF) m/z: $[M + Na]^+$ Calcd for $C_{22}H_{22}N_2O_5SNa$ 449.1142; Found 449.1141.

IR (neat) νmax: 3361, 3306, 1724, 1660, 1332, 1159, 748, 549.

Mp: 157-158 °C.

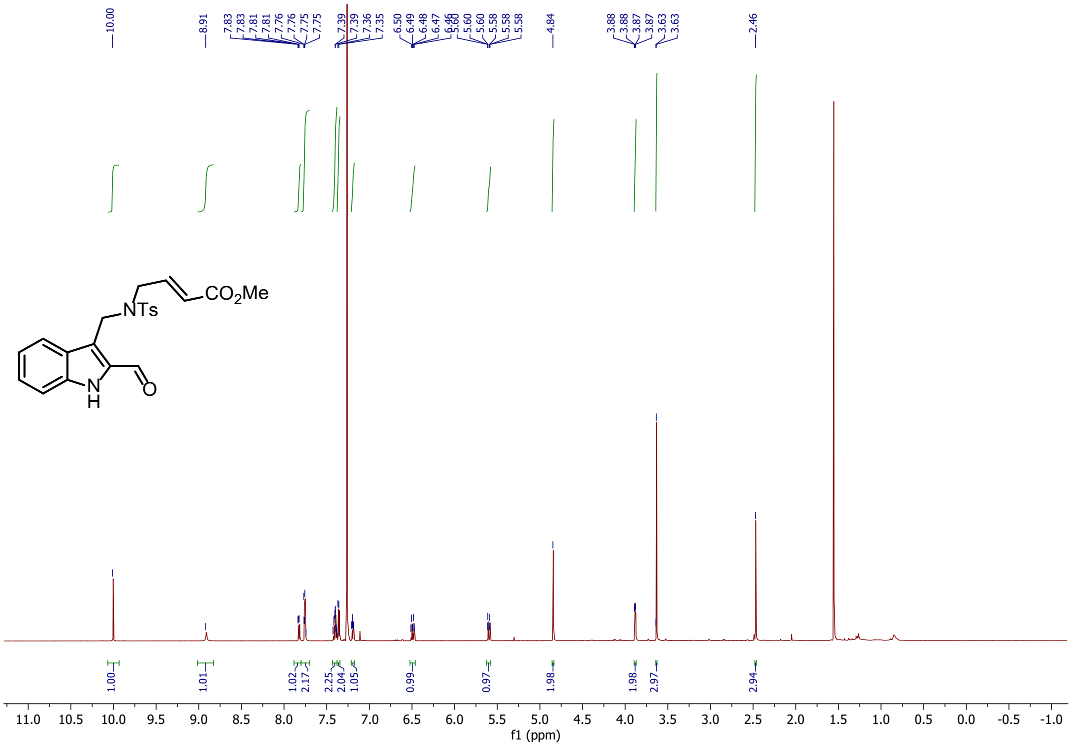

*Figure 26: **^{1}H NMR (700 MHz, CDCl3) of 4a.***

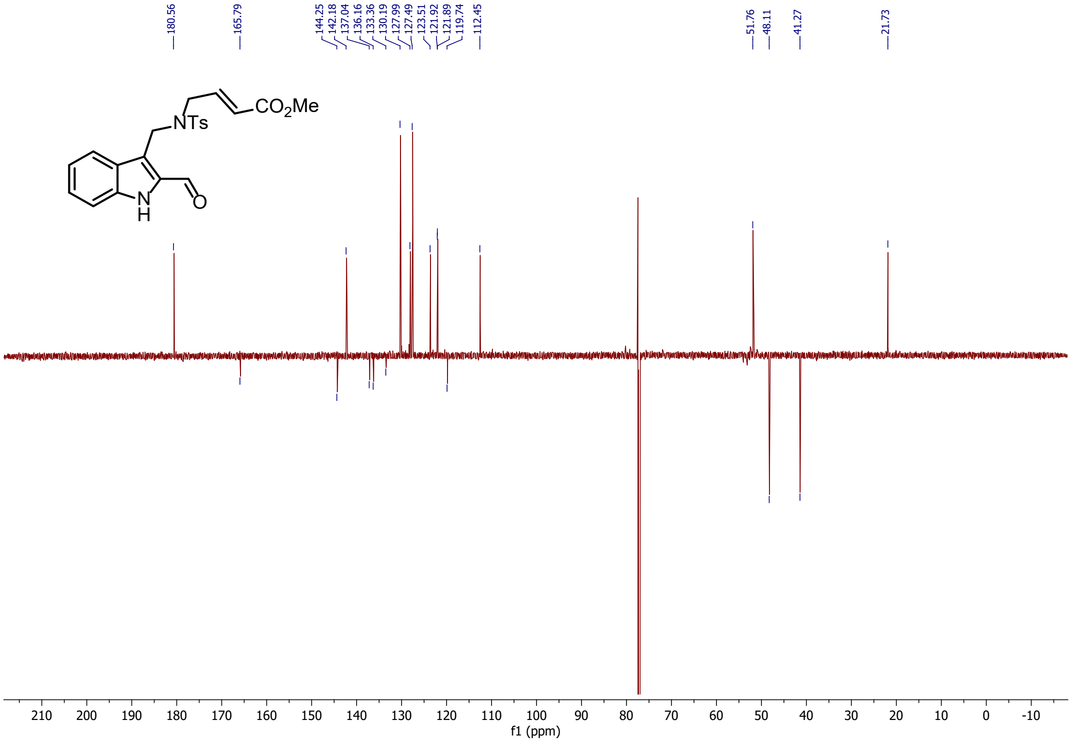

*Figure 27: **^{13}C NMR (176 MHz, CDCl3) of 4a.***

e. Synthesis of methyl (*E*)-4-((*N*-((2-formyl-1-methyl-1*H*-indol-3-yl)methyl)-4-methylphenyl)sulfonamido)but-2-enoate (4b)

N-allyl-*N*-((2-formyl-1-methyl-1H-indol-3-yl)methyl)-4-methylbenzenesulfonamide (SM-4b-1)

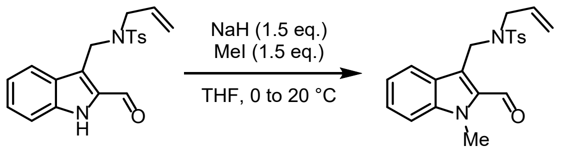

SM-4a-5 **SM-4b-1**: 33%

A 25-mL Schlenk flask was charged with NaH (60% in paraffine, 18 mg, 0.45 mmol, 1.5 eq.) and THF (3 mL), followed by cooling to 0 °C. **SM-4a-5** (111 mg, 0.30 mmol, 1.00 eq.) was added dropwise as a solution in THF (3 mL). The resulting mixture was stirred for 10 min at 0 °C, before the addition of iodomethane (28 µL, 0.45 mmol, 1.5 eq.) at the same temperature. The resulting mixture was stirred for 3 h at 20 °C. Work-up was performed by dilution with EtOAc (10 mL) and a sat. aq. solution of NH_4Cl (10 mL). The phases were separated and the aqueous phase was extracted twice with EtOAc (10 mL). The organic phases were combined, dried over Na_2SO_4 and filtered. The solution was concentrated under reduced pressure and the crude mixture was purified by flash chromatography using a gradient of heptanes/EtOAc to yield **SM-4b-1** as a colorless oil (38 mg, 0.10 mmol, 33% yield).

^{1}H NMR (600 MHz, $CDCl_3$) δ 10.13 (s, 1H), 7.93 (d, *J* = 8.2 Hz, 1H), 7.76 (d, *J* = 8.1 Hz, 3H), 7.46-7.42 (m, 1H), 7.40-7.34 (m, 2H), 7.33 (d, *J* = 8.0 Hz, 2H), 7.18 (app. t, *J* = 7.5 Hz, 1H), 5.42 (ddt, *J* = 16.6, 10.2, 6.3 Hz, 1H), 4.94 (dd, *J* = 48.8, 13.7 Hz, 2H), 4.86 (s, 2H), 4.06 (s, 3H), 3.75 (d, *J* = 6.2 Hz, 2H), 2.46 (s, 3H), 1.53 (s, 3H).

^{13}C NMR (151 MHz, $CDCl_3$) δ 182.1, 143.7, 139.7, 137.0, 132.6, 130.0 (2C), 128.4, 127.5 (2C), 126.3, 122.3, 121.9, 121.4, 119.3, 110.4, 49.6, 40.5, 31.9, 21.7. *One quaternary carbon could not be found under these conditions.*

HRMS (ESI-TOF) m/z: $[M + Na]^+$ Calcd for $C_{21}H_{22}N_2O_3SNa$ 405.1243; Found 405.1229.

IR (neat) νmax: 3309, 2977, 2951, 1653, 1455, 1436, 1269, 1248, 1154, 730.

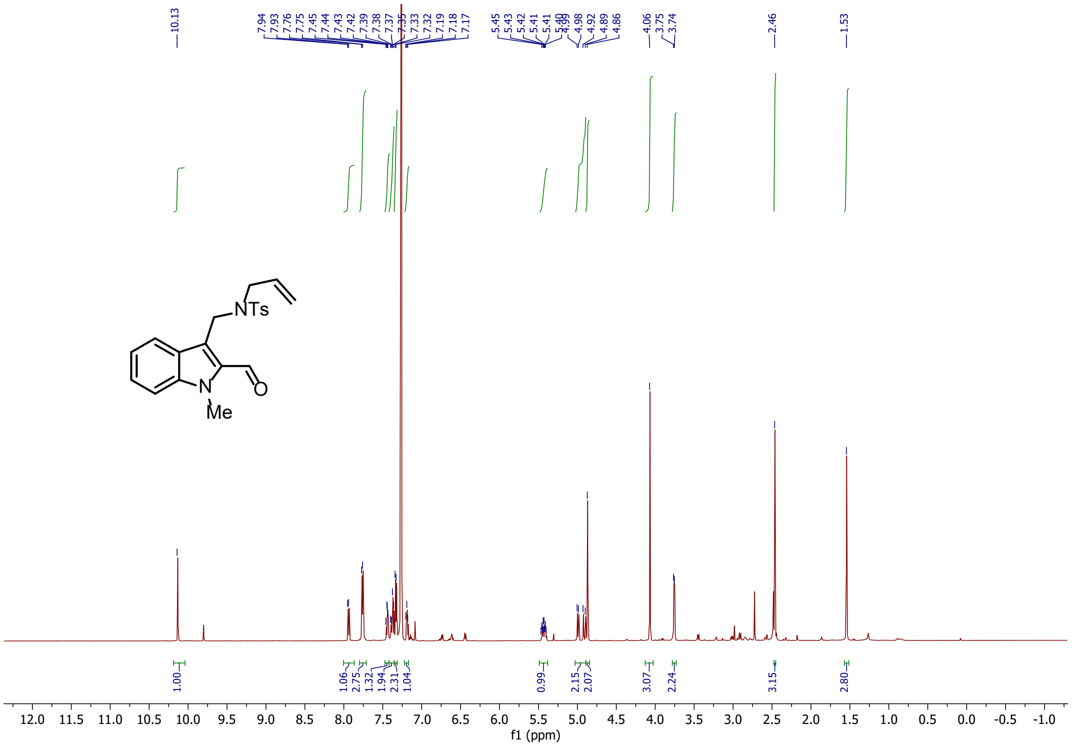

Figure 28: ***¹H NMR (600 MHz, CDCl₃) of SM-4b-1.***

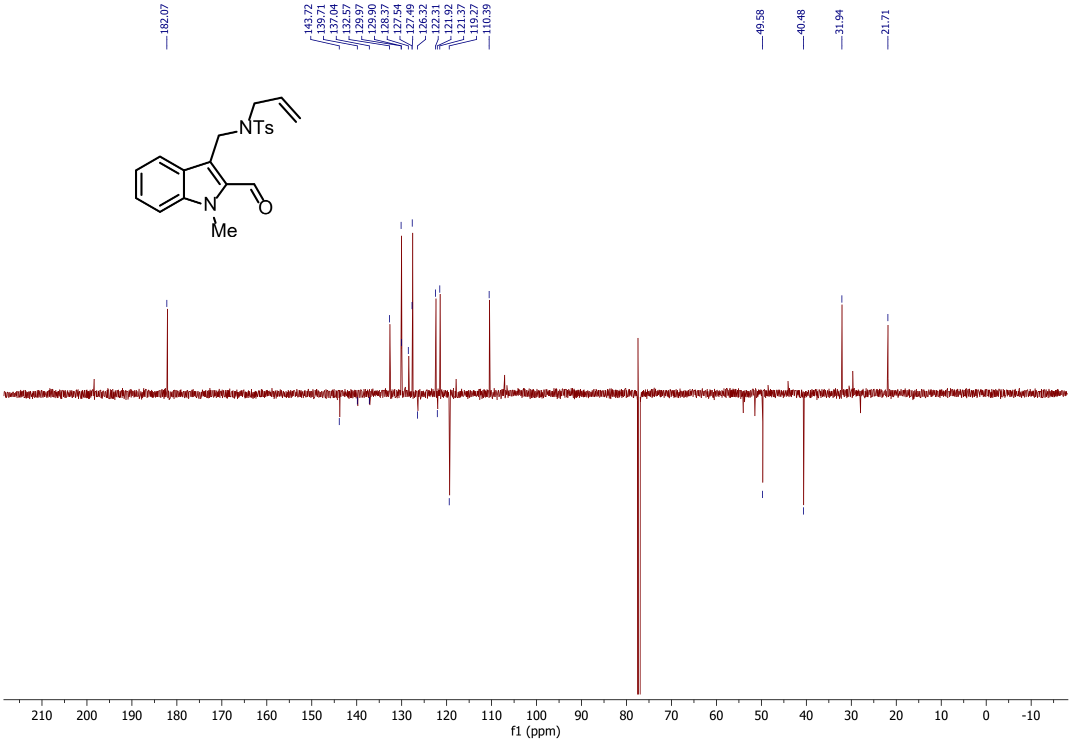

Figure 29: ***¹³C NMR (151 MHz, CDCl₃) of SM-4b-1.***

Methyl (*E*)-4-((*N*-((2-formyl-1-methyl-1*H*-indol-3-yl)methyl)-4-methylphenyl)sulfonamido)but-2-enoate (4b)

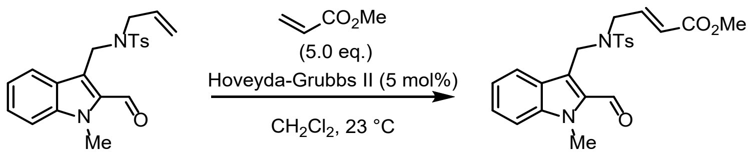

SM-4b-1 **4b**: 53%

A Schlenk flask was charged with **SM-4b-1** (62 mg, 0.162 mmol, 1.00 eq.), CH_2Cl_2 (2.0 mL), methyl acrylate (70 µL, 0.81 mmol, 5.0 eq.) and Hoveyda-Grubbs 2nd generation catalyst (5 mg, 0.001 mmol, 5 mol%). The mixture was stirred for 5 h at 23 °C, before being concentrated under reduced pressure. The crude mixture was purified twice by flash chromatography using a gradient of heptanes/EtOAc to yield **4b** as a light purple semi-solid (38 mg, 0.086 mmol, 53% yield).

^{1}H NMR (600 MHz, $CDCl_3$) δ 10.13 (s, 1H), 7.85 (d, *J* = 8.2 Hz, 1H), 7.75 (d, *J* = 8.2 Hz, 2H), 7.44 (dd, *J* = 9.2, 6.0 Hz, 1H), 7.35 (d, *J* = 7.5 Hz, 3H), 7.19 (app. t, *J* = 7.5 Hz, 1H), 6.43 (dt, *J* = 15.7, 6.0 Hz, 1H), 5.51 (d, *J* = 15.7 Hz, 1H), 4.85 (s, 2H), 4.04 (s, 3H), 3.83 (d, *J* = 5.0 Hz, 2H), 3.62 (s, 3H), 2.47 (s, 3H).

^{13}C NMR (151 MHz, $CDCl_3$) δ 181.8, 165.8, 144.2, 142.5, 139.5, 136.0, 130.1 (2C), 127.7, 127.5 (2C), 126.4, 123.0, 121.8, 121.7, 121.0, 110.5, 51.7, 48.1, 41.5, 31.8, 21.7

HRMS (ESI-TOF) m/z: $[M + Na]^+$ Calcd for $C_{23}H_{24}N_2O_5SNa$ 463.1298; Found 463.1297.

IR (neat) vmax: 3057, 2951, 2924, 2853, 1720, 1662, 1158, 1093, 747, 514.

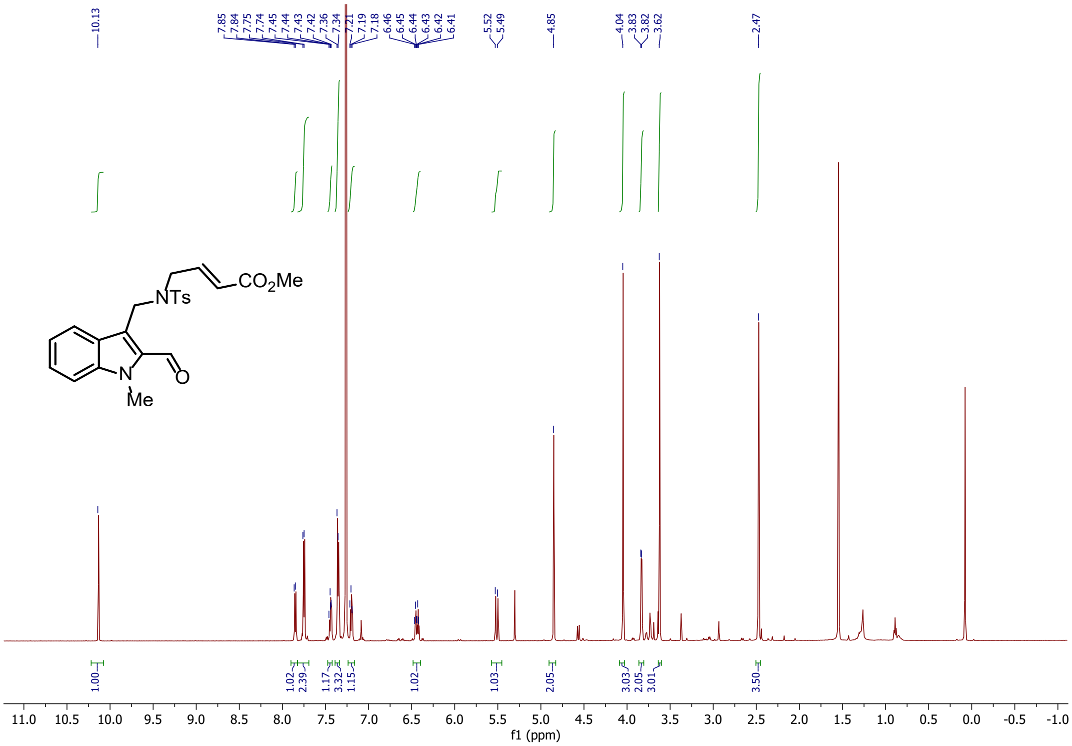

*Figure 30: **^{1}H NMR (600 MHz, $CDCl_3$) of 4b.***

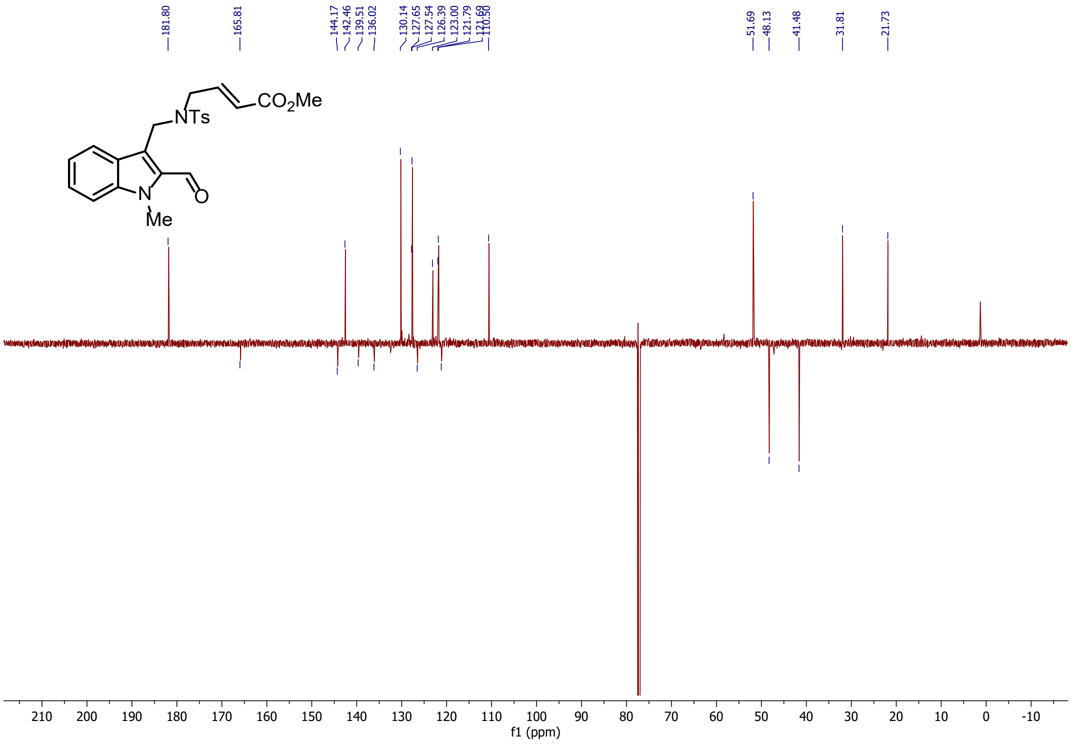

*Figure 31: **^{13}C NMR (151 MHz, $CDCl_3$) of 4b.***

f. Synthesis of methyl (*E*)-4-((*N*-((2-formyl-5-methoxy-1*H*-indol-3-yl)methyl)-4-methylphenyl)sulfonamido)but-2-enoate (4c)

Methyl 3-(((*N*-allyl-4-methylphenyl)sulfonamido)methyl)-5-methoxy-1*H*-indole-2-carboxylate (SM-4c-4)

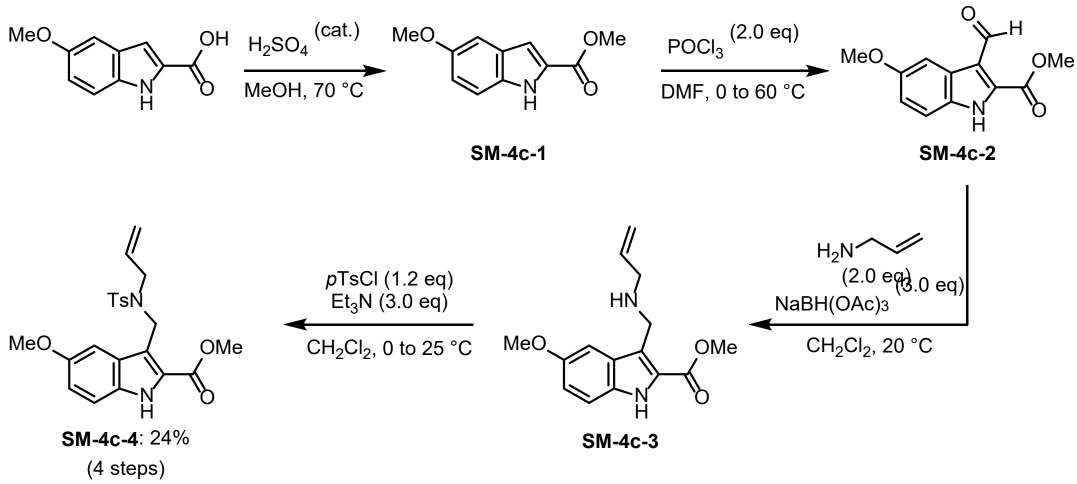

A 50 mL round-bottomed flask fitted with a condenser was charged 5-methoxyindole-2-carboxylic acid (1.91 g, 10.0 mmol, 1.00 eq.) followed by MeOH (20 mL) and H_2SO_4 (100 µL, 1.80 mmol, 18 mol%). The mixture was heated at 70 °C for 18 h, before being allowed to return to ambient temperature. The orange solid was filtered and washed with heptanes. The solid was dissolved using Et_2O and the solution was concentrated under reduced pressure to yield **SM-4c-1**, which was used as such in the next step.

DMF (1.25 mL, 16.2 mmol, 4.00 eq.) was charged in a 50 mL two-necked round-bottomed flask and cooled to 0 °C. $POCl_3$ (0.75 mL, 8.09 mmol, 2.00 eq.) was added dropwise over 5 min followed by the addition of a solution of **SM-4c-1** (0.83 g, 4.04 mmol, 1.00 eq.) in DMF (10 mL). The mixture was heated at 60 °C for 3 h, before adding dropwise H_2O (20 mL) – temperature reached 85 °C – and was then allowed to return to ambient temperature. The suspension was filtered over sintered glass and washed abundantly with H_2O. The solid was dissolved in MeOH and the filtrate was concentrated under reduced pressure to yield **SM-4c-2**, which was used as such in the next step.

In 250 mL round-bottomed flask, **SM-4c-2** (assumed pure, 580 mg, 2.49 mmol, 1.00 eq.) was dissolved in CH_2Cl_2 (25 mL) followed by the addition of allylamine (0.38 mL, 4.97 mmol, 2.00 eq.), $NaBH(OAc)_3$ (1.58 g, 7.46 mmol, 3.00 eq.) and AcOH (0.14 mL, 2.49 mmol, 1.00 eq.). The mixture was stirred for 20 h at 20 °C, before being poured into a sat. aq. solution of Na_2CO_3 (25 mL) and diluted with CH_2Cl_2 (25 mL). The phases were separated and the aqueous phase was extracted twice more with CH_2Cl_2 (25 mL). The organic phases were combined, dried over

Na_2SO_4 and filtered. The solution was concentrated under reduced pressure to yield **SM-4c-3**, which was used as such in the next step.

In a 50 mL round-bottomed flask, **SM-4c-3** (assumed pure, 683 mg, 2.49 mmol, 1.00 eq.) was dissolved in CH_2Cl_2 (25 mL), cooled to 0 °C followed by the addition of Et_3N (1.04 mL, 7.46 mmol, 3.00 eq.) and *p*-TsCl (0.57 g, 2.99 mmol, 1.20 eq.). The mixture was stirred for 30 min at 20 °C, before being diluted with CH_2Cl_2 (25 mL) and a sat. aq. solution of NH_4Cl (25 mL). The phases were separated and the aqueous phase was extracted twice more with CH_2Cl_2 (25 mL). The organic phases were combined, dried over Na_2SO_4 and filtered through a cotton pad. The solution was concentrated under reduced pressure and the crude mixture was purified by flash chromatography using a gradient of heptanes/EtOAc to yield **SM-4c-4** as a yellow solid (1.03 g, 2.40 mmol, 24% yield over 4 steps).

^{1}H NMR (400 MHz, $CDCl_3$) δ 8.67 (br s, 1H), 7.77 (d, *J* = 8.3 Hz, 2H), 7.64 (d, *J* = 2.4 Hz, 1H), 7.32 (d, *J* = 8.1 Hz, 2H), 7.03 (dd, *J* = 9.0, 2.4 Hz, 1H), 5.42 (ddt, *J* = 16.5, 10.2, 6.2 Hz, 1H), 4.94 (s, 2H), 4.88 – 4.76 (m, 2H), 3.89 (s, 3H), 3.87 (s, 3H), 3.72 (d, *J* = 6.2 Hz, 2H), 2.45 (s, 3H). *One aromatic proton is missing due to signal overlap with the solvent peak.*

^{13}C NMR (101 MHz, $CDCl_3$) δ 162.1, 155.0, 143.3, 137.2, 133.2, 131.1, 129.8 (2C), 128.3, 127.5 (2C), 124.9, 118.4, 117.4, 117.2, 112.6, 101.9, 55.8, 51.9, 50.1, 42.5, 21.7.

HRMS (ESI-TOF) m/z: $[M + H]^+$ Calcd for $C_{22}H_{25}N_2O_5S$ 429.1479; Found 429.1477.

IR (neat) νmax: 3347, 2994, 2950, 2833, 2362, 1708, 1627, 1597, 1551, 1493, 1460, 1438, 1380, 1335, 1304.

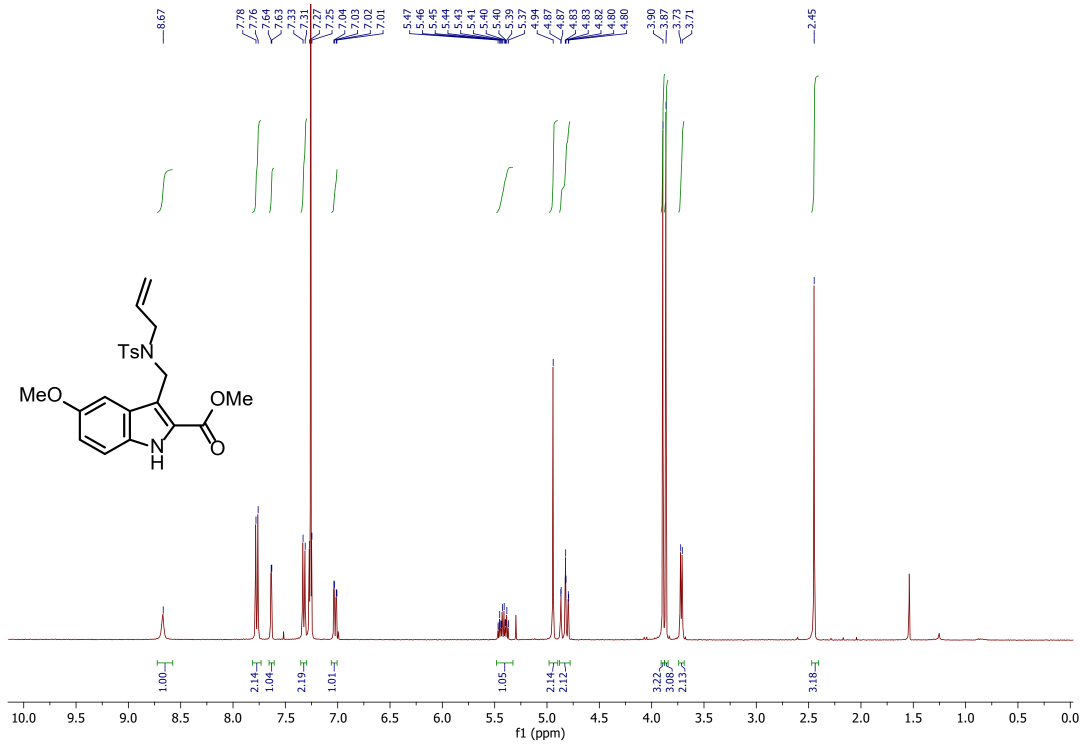

*Figure 32: **^{1}H NMR (400 MHz, CDCl$_3$) of SM-4c-4.***

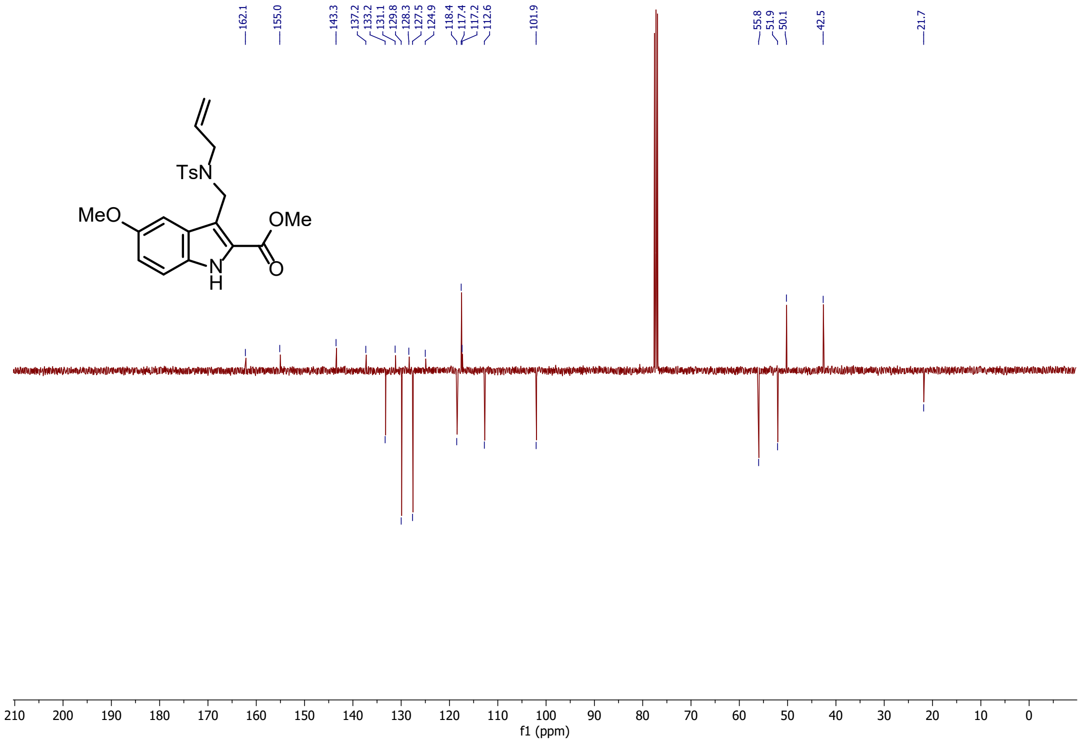

*Figure 33: **^{13}C NMR (101 MHz, CDCl$_3$) of SM-4c-4.***

N-allyl-*N*-((2-formyl-5-methoxy-1*H*-indol-3-yl)methyl)-4-methylbenzenesulfonamide (SM-4c-6)

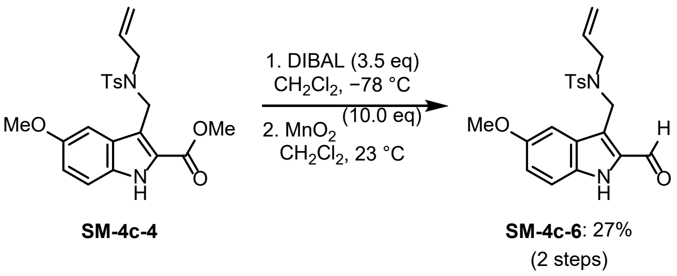

A 100 mL two-necked round-bottomed flask was charged with **SM-4c-4** (1.03 g, 2.40 mmol, 1.00 eq.), CH_2Cl_2 (50 mL) and the mixture was cooled to −78 °C. DIBAL (1 mol/L in PhMe, 8.4 mL, 8.4 mmol, 3.5 eq.) was added dropwise before being stirred for 1 h at −78 °C. After this time, the mixture was allowed to stirred at 0 °C for 10 min followed by the dropwise addition of H_2O (340 µL), 1 M NaOH (840 µL) and H_2O (340 µL). The mixture was allowed to return to ambient temperature and was stirred for 15 min. $MgSO_4$ was added, stirred for 15 min followed by filtration over Celite using CH_2Cl_2. The filtrate was concentrated under reduced pressure to yield **SM-4c-5**, which was used as such in the next step.

In a 50 mL round-bottomed flask was charged **SM-4c-5** (assumed pure, 921 mg, 2.30 mmol, 1.00 eq.), CH_2Cl_2 (25 mL) followed by MnO_2 (2.00 g, 23.0 mmol, 10.0 eq.) and $MgSO_4$ (2.0 g). The mixture was stirred at 23 °C for 20 h, before being filtered over Celite, eluted with CH_2Cl_2 and concentrated under reduced pressure. The crude mixture was purified by flash chromatography using a gradient of heptanes/EtOAc to yield **SM-4c-6** as a yellow solid (260 mg, 0.65 mmol, 27% over 2 steps).

^{1}H NMR (700 MHz, $CDCl_3$) δ 9.93 (s, 1H), 8.86 (br s, 1H), 7.81 – 7.75 (m, 2H), 7.41 (d, *J* = 2.4 Hz, 1H), 7.34 (d, *J* = 7.9 Hz, 2H), 7.30 (d, *J* = 8.9 Hz, 1H), 7.08 (dd, *J* = 9.0, 2.4 Hz, 1H), 5.42 (ddt, *J* = 16.6, 10.2, 6.4 Hz, 1H), 5.03 – 5.01 (m, 1H), 4.95 – 4.90 (m, 1H), 4.83 (s, 2H), 3.86 (s, 3H), 3.78 (d, *J* = 6.4 Hz, 2H), 2.46 (s, 3H).

^{13}C NMR (176 MHz, $CDCl_3$) δ 180.5, 155.2, 143.8, 137.2, 133.7, 132.7, 132.5, 130.1 (2C), 127.9, 127.4 (2C), 120.2, 119.8, 119.5, 113.3, 101.7, 55.8, 49.6, 40.4, 21.7.

HRMS (ESI-TOF) m/z: $[M + H]^+$ Calcd for $C_{21}H_{23}N_2O_4S$ 399.1373; Found 399.1369.

IR (neat) νmax: 3327, 2920, 2849, 2362, 1653, 1597, 1539, 1493, 1469, 1436, 1336, 1215, 1160, 1089, 1026, 1011.

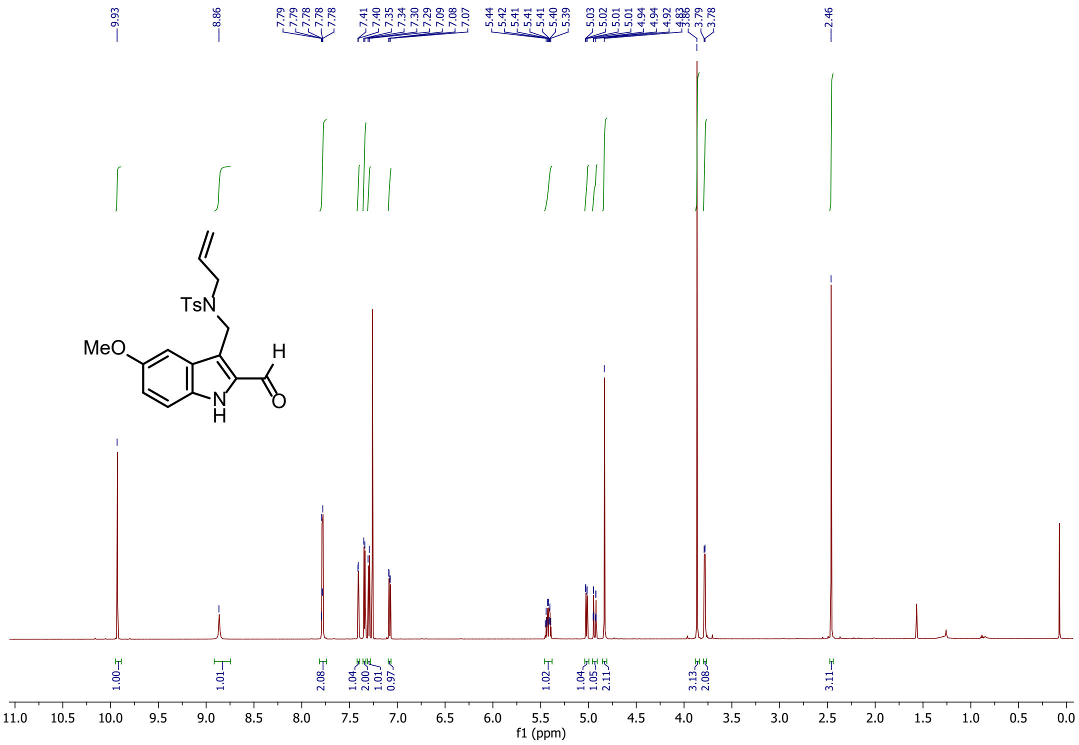

Figure 34: ^{1}H NMR (700 MHz, CDCl3) of SM-4c-6.

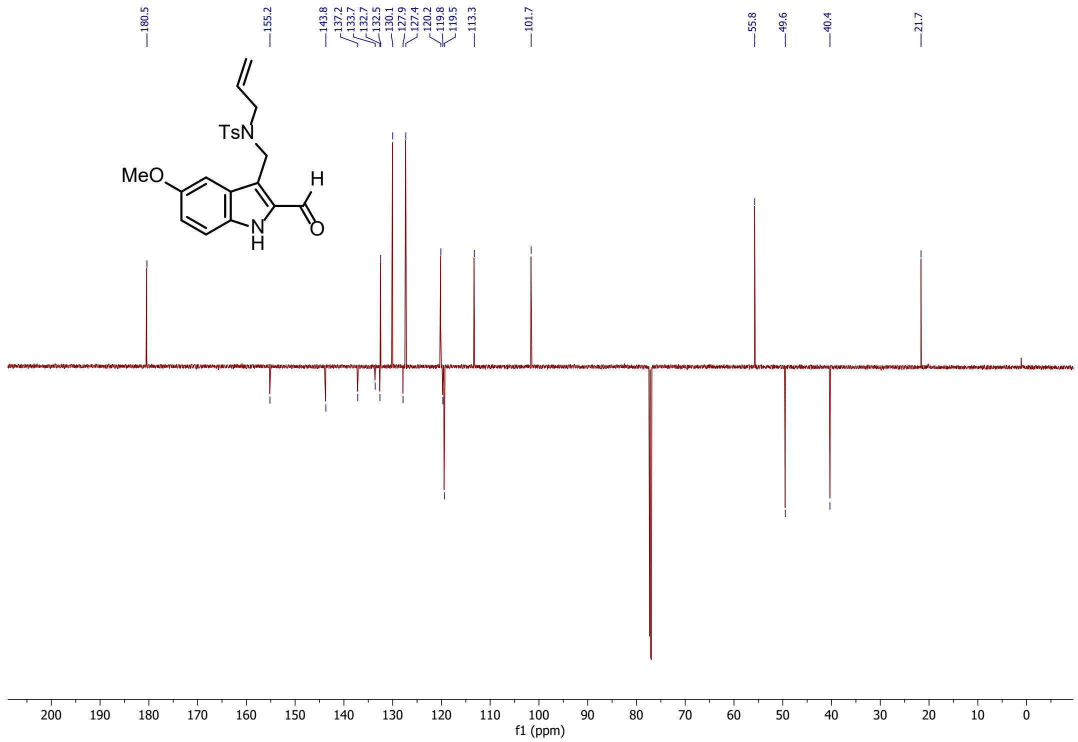

Figure 35: ^{13}C NMR (176 MHz, CDCl3) of SM-4c-6.

Methyl (*E*)-4-((*N*-((2-formyl-5-methoxy-1*H*-indol-3-yl)methyl)-4-methylphenyl)sulfonamido)but-2-enoate (4c)

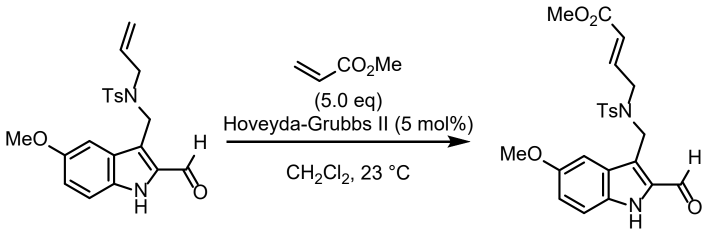

A Schlenk flask was charged with **SM-4c-6** (260 mg, 0.65 mmol, 1.00 eq.), CH_2Cl_2 (35.0 mL), methyl acrylate (270 µL, 3.25 mmol, 5.00 eq.) and Hoveyda-Grubbs 2nd generation catalyst (20 mg, 0.03 mmol, 5 mol%). The mixture was stirred for 18 h at 23 °C, before being concentrated under reduced pressure. The crude mixture was purified twice by flash chromatography using a gradient of heptanes/EtOAc/EtOH to yield **4c** as a purple solid (9 mg, 0.02 mmol, 3% yield, 17% BRSM).

^{1}H NMR (400 MHz, $CDCl_3$) δ 9.83 (s, 1H), 8.85 (br s, 1H), 7.70 (d, *J* = 8.3 Hz, 2H), 7.29 (d, *J* = 8.0 Hz, 2H), 7.24 – 7.20 (m, 2H), 7.01 (dd, *J* = 9.2, 2.3 Hz, 1H), 6.42 (dt, *J* = 15.7, 6.0 Hz, 1H), 5.55 (dt, *J* = 15.7, 1.5 Hz, 1H), 4.74 (s, 2H), 3.81 (dd, *J* = 6.0, 1.5 Hz, 2H), 3.79 (s, 3H), 3.56 (s, 3H), 2.39 (s, 3H).

^{13}C NMR (151 MHz, $CDCl_3$) δ 180.1, 165.8, 155.5, 144.2, 142.2, 136.3, 133.7, 132.5, 130.2 (2C), 127.9, 127.4 (2C), 123.5, 120.4, 119.0, 113.5, 101.1, 55.8, 51.8, 47.9, 41.3, 21.7.

HRMS (ESI-TOF) m/z: $[M + H]^+$ Calcd for $C_{23}H_{25}N_2O_6S$ 457.1428; Found 457.1429.

IR (neat) νmax: 3326, 2950, 2851, 2362, 1722, 1653, 1597, 1540, 1494, 1467, 1437, 1376, 1335, 1305, 1285.

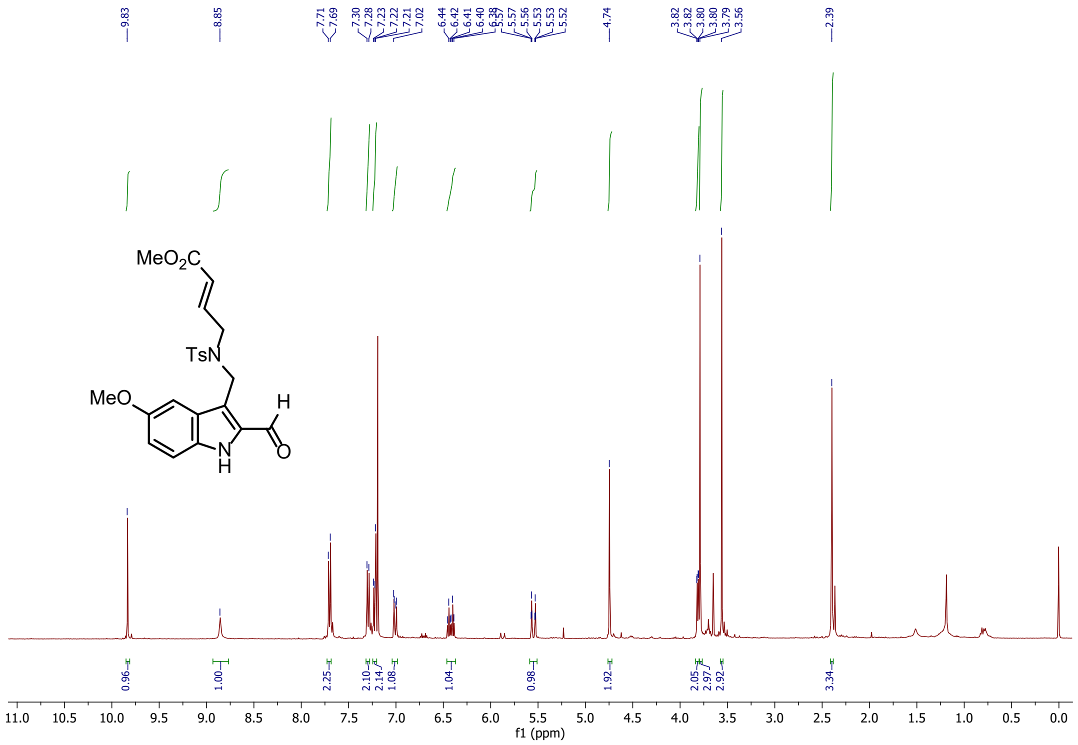

Figure 36: ***^{1}H NMR (400 MHz, $CDCl_3$) of 4c.***

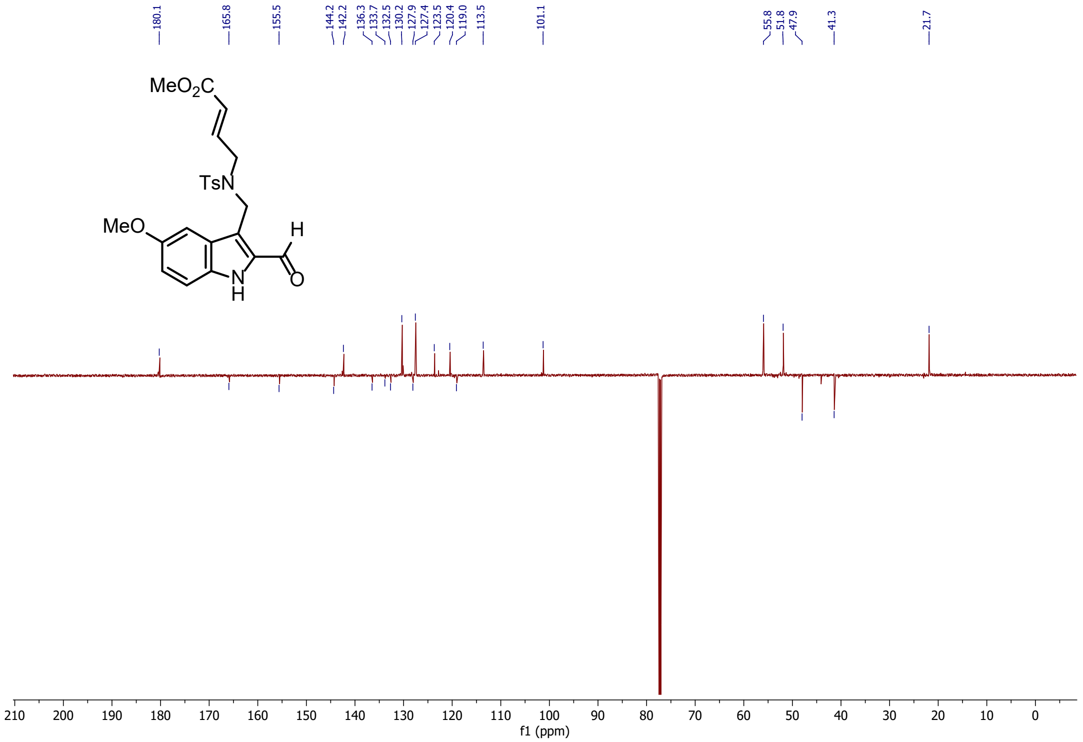

Figure 37: ***^{13}C NMR (151 MHz, $CDCl_3$) of 4c.***

g. Synthesis of methyl (*E*)-4-((*N*-((5-bromo-2-formyl-1*H*-indol-3-yl)methyl)-4-methylphenyl)sulfonamido)but-2-enoate (4d)

Ethyl 3-(((*N*-allyl-4-methylphenyl)sulfonamido)methyl)-5-bromo-1*H*-indole-2-carboxylate (SM-4d-3)

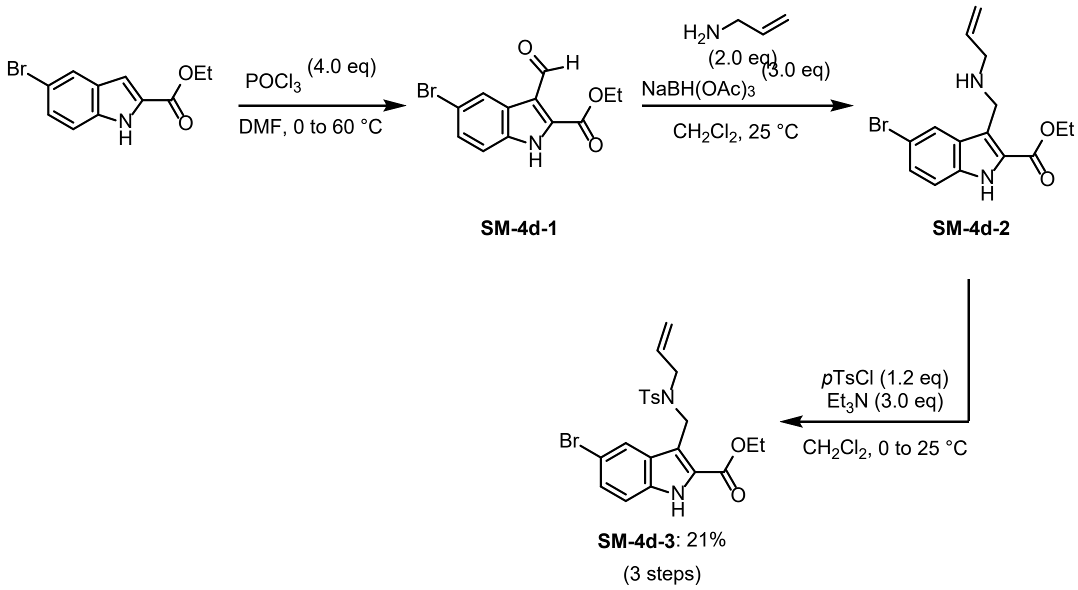

DMF (3.09 mL, 40.0 mmol, 4.00 eq.) was charged in a 25 mL two-necked round-bottomed flask fitted with a condenser and cooled to 0 °C. $POCl_3$ (3.09 mL, 40.0 mmol, 4.00 eq.) was added dropwise over 20 min followed by the addition of a solution of 2-(ethoxycarbonyl)-5-bromo-indole (2.68 g, 10.0 mmol, 1.00 eq) in DMF (10 mL). The mixture was heated at 60 °C for 4 h, before being allowed to return to ambient temperature (~25 °C) and aged for 18 h. The reaction mixture was poured into ice-water (100 mL), filtered over sintered glass and washed abundantly with water. The solid was dissolved with MeOH and the filtrate was concentrated under reduced pressure to yield **SM-4d-1**, which was used as such in the next step.

In 50 mL round-bottomed flask, **SM-4d-1** (assumed pure, 2.96 g, 10.0 mmol, 1.00 eq.) was dissolved in CH_2Cl_2 (25 mL) followed by the addition of allylamine (1.53 mL, 20.0 mmol, 2.0 eq.), $NaBH(OAc)_3$ (6.35 g, 30.0 mmol, 3.0 eq.) and AcOH (0.57 mL, 10 mmol, 1.0 eq.). The mixture was stirred for 2 h at 25 °C, before being poured into a sat. aq. solution of Na_2CO_3 (50 mL) and diluted with CH_2Cl_2 (50 mL). The phases were separated and the aqueous phase was extracted twice more with CH_2Cl_2 (20 mL). The organic phases were combined, dried over Na_2SO_4 and filtered. The solution was concentrated under reduced pressure to yield **SM-4d-2**, which was used as such in the next step.

In a 250 mL round-bottomed flask, **SM-4d-2** (assumed pure, 3.37 g, 10.0 mmol, 1.00 eq.) was dissolved in CH_2Cl_2 (60 mL), cooled to 0 °C followed by the addition of Et_3N (4.18 mL, 30.0 mmol, 3.00 eq.) and *p*-TsCl (2.29 g, 12.0

mmol, 1.20 eq.). The mixture was stirred for 30 min at 20 °C, before being diluted with CH_2Cl_2 (50 mL) and a sat. aq. solution of NH_4Cl (50 mL). The phases were separated and the aqueous phase was extracted twice more with CH_2Cl_2 (25 mL). The organic phases were combined, dried over Na_2SO_4 and filtered through a cotton pad. The solution was concentrated under reduced pressure and the crude mixture was purified by flash chromatography using a gradient of heptanes/EtOAc to yield **SM-4d-3** as a yellow solid (1.04 g, 2.12 mmol, 21% yield over 3 steps).

^{1}H NMR (700 MHz, $CDCl_3$) δ 8.86 (br s, 1H), 8.05 (d, *J* = 1.8 Hz, 1H), 7.82 – 7.70 (m, 2H), 7.40 (dd, *J* = 8.7, 1.9 Hz, 1H), 7.33 (d, *J* = 7.9 Hz, 2H), 7.24 (d, *J* = 8.8 Hz, 1H), 5.45 (ddt, *J* = 16.5, 10.2, 6.2 Hz, 1H), 4.89 (s, 2H), 4.86 – 4.79 (m, 2H), 4.36 (q, *J* = 7.1 Hz, 2H), 3.71 (d, *J* = 6.2 Hz, 2H), 2.45 (s, 3H), 1.35 (t, *J* = 7.1 Hz, 3H).

^{13}C NMR (176 MHz, $CDCl_3$) δ 161.4, 143.6, 136.6, 134.3, 133.4, 129.9 (2C), 129.34, 129.27, 127.6 (2C), 126.0, 124.6, 117.5, 116.8, 114.5, 113.2, 61.5, 50.3, 42.3, 21.7, 14.4.

HRMS (ESI-TOF) m/z: $[M + Na]^+$ Calcd for $C_{22}H_{23}{}^{79}BrN_2O_4SNa$ 513.0454; Found 513.0451.

IR (neat) νmax: 3328, 3080, 2981, 2928, 2361, 1685, 1597, 1545, 1493, 1444, 1395, 1376, 1327, 1290, 1242.

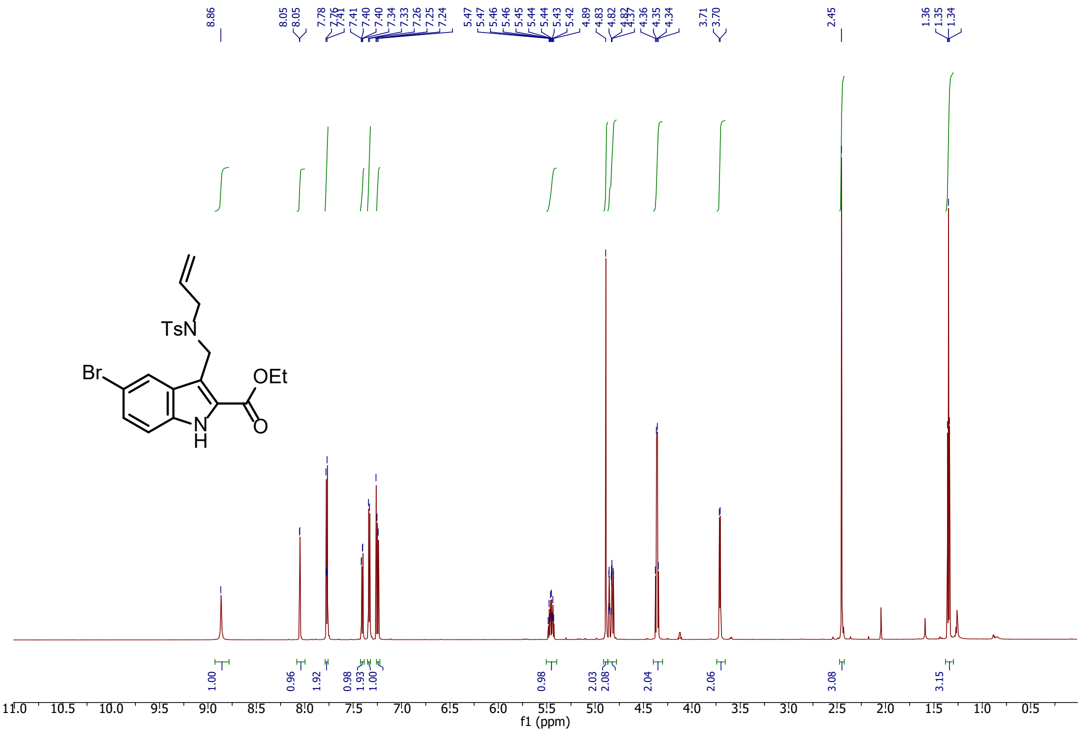

Figure 38: ***^{1}H NMR (700 MHz, $CDCl_3$) of SM-4d-3.***

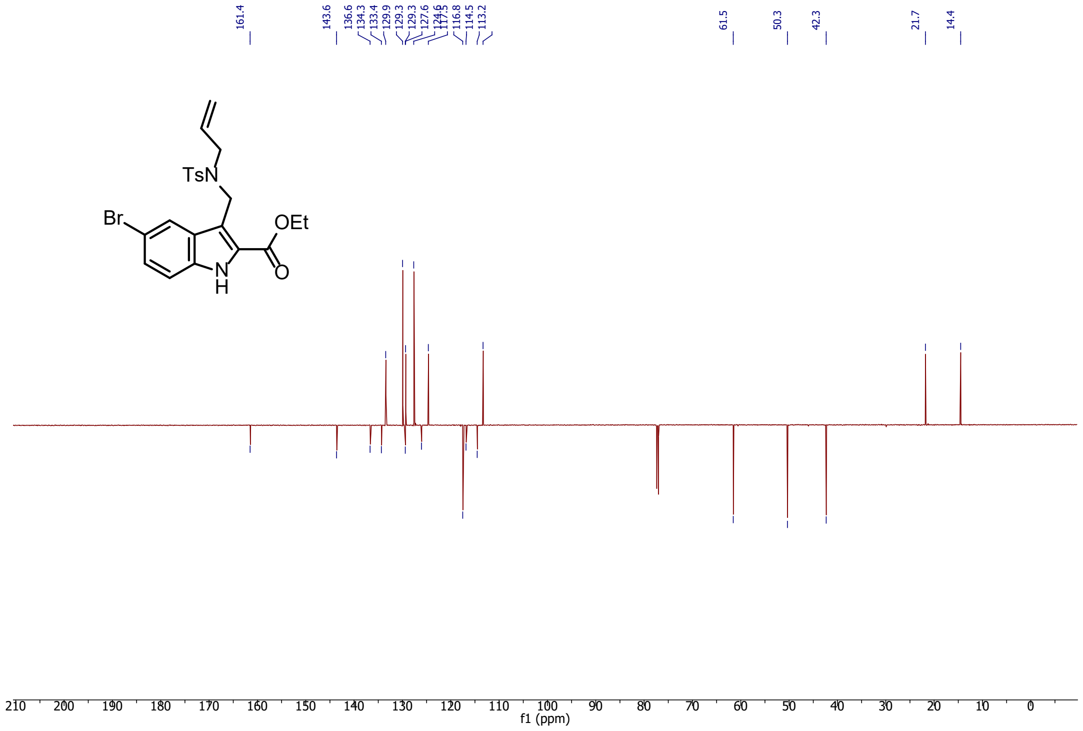

Figure 39: ***^{13}C NMR (176 MHz, $CDCl_3$) of SM-4d-3.***

***N*-allyl-*N*-((5-bromo-2-formyl-1*H*-indol-3-yl)methyl)-4-methylbenzenesulfonamide (SM-4d-5)**

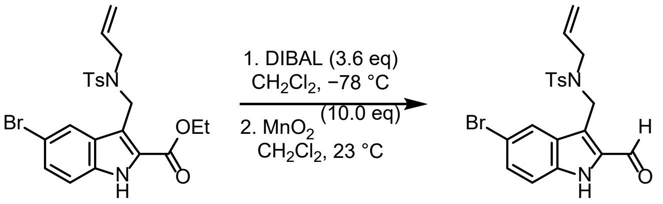

SM-4d-3 **SM-4d-5**: 18% (2 steps)

A 100 mL two-necked round-bottomed flask was charged with **SM-4d-3** (1.04 g, 2.12 mmol, 1.00 eq.), CH_2Cl_2 (42 mL) and the mixture was cooled to −78 °C. DIBAL (1 mol/L in PhMe, 7.6 mL, 7.62 mmol, 3.6 eq.) was added dropwise before being stirred for 1 h at −78 °C. After this time, the mixture was allowed to stirred at 0 °C for 10 min followed by the dropwise addition of H_2O (300 µL), 1 M NaOH (750 µL) and H_2O (750 µL). The mixture was allowed to return to ambient temperature and was stirred for 15 min. $MgSO_4$ was added, stirred for 15 min followed by filtration over Celite using CH_2Cl_2. The filtrate was concentrated under reduced pressure and the crude mixture was partially purified by flash chromatography using to yield **SM-4d-4** that was used as such in the next step.

In a 50 mL round-bottomed flask was charged **SM-4d-4** (200 mg, 0.45 mmol, 1.00 eq.), CH_2Cl_2 (4.5 mL) followed by MnO_2 (387 mg, 4.45 mmol, 10.0 eq.) and $MgSO_4$ (387 mg). The mixture was stirred at 23 °C for 20 h, before being filtered over Celite, eluted with CH_2Cl_2 and concentrated under reduced pressure. The crude mixture was purified by flash chromatography using a gradient of heptanes/EtOAc to yield **SM-4d-5** as a white solid (170 mg, 0.38 mmol, 18% over 2 steps).

^{1}H NMR (400 MHz, CDCl$_3$) δ 10.06 (s, 1H), 8.91 (br s, 1H), 7.83 (d, *J* = 1.8 Hz, 1H), 7.76 (d, *J* = 8.3 Hz, 2H), 7.46 (dd, *J* = 8.8, 1.9 Hz, 1H), 7.35 (d, *J* = 7.9 Hz, 2H), 5.50 (ddt, *J* = 16.6, 10.2, 6.3 Hz, 1H), 5.06 (dd, *J* = 10.2, 1.2 Hz, 1H), 4.95 (dd, *J* = 17.1, 1.3 Hz, 1H), 4.77 (s, 2H), 3.79 (d, *J* = 6.3 Hz, 2H), 2.46 (s, 3H). *One aromatic proton is missing due to signal overlap with the solvent peak.*

^{13}C NMR (101 MHz, CDCl$_3$) δ 181.2, 144.0, 136.8, 135.7, 134.0, 132.6, 130.8, 130.1 (2C), 128.9, 127.4 (2C), 124.4, 119.6, 114.7, 114.1, 49.9, 40.2, 21.8. *One quaternary carbon was not observed in those conditions.*

HRMS (ESI-TOF) m/z: $[M + Na]^+$ Calcd for $C_{20}H_{19}{}^{79}BrN_2O_3SNa$ 469.0192; Found 469.0189.

IR (neat) vmax: 3312, 3065, 2920, 2864, 1655, 1597, 1539, 1453, 1331, 1289, 1213, 1158, 1090, 1060.

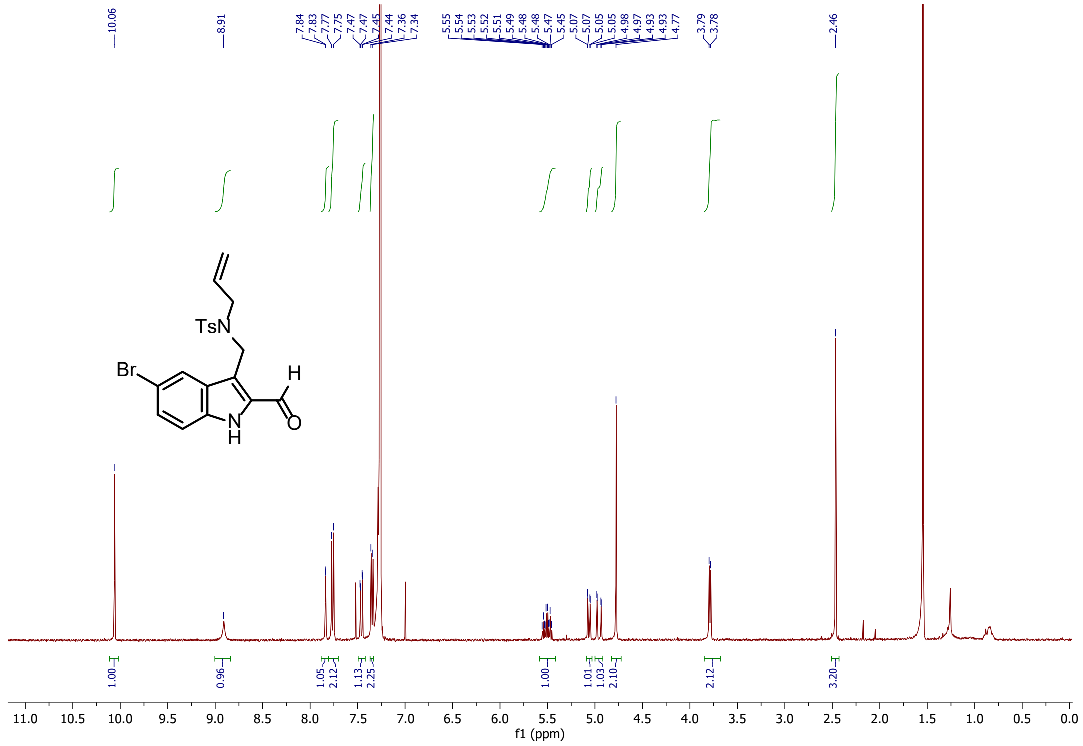

Figure 40: ^{1}H NMR (400 MHz, CDCl3) of SM-4d-5.

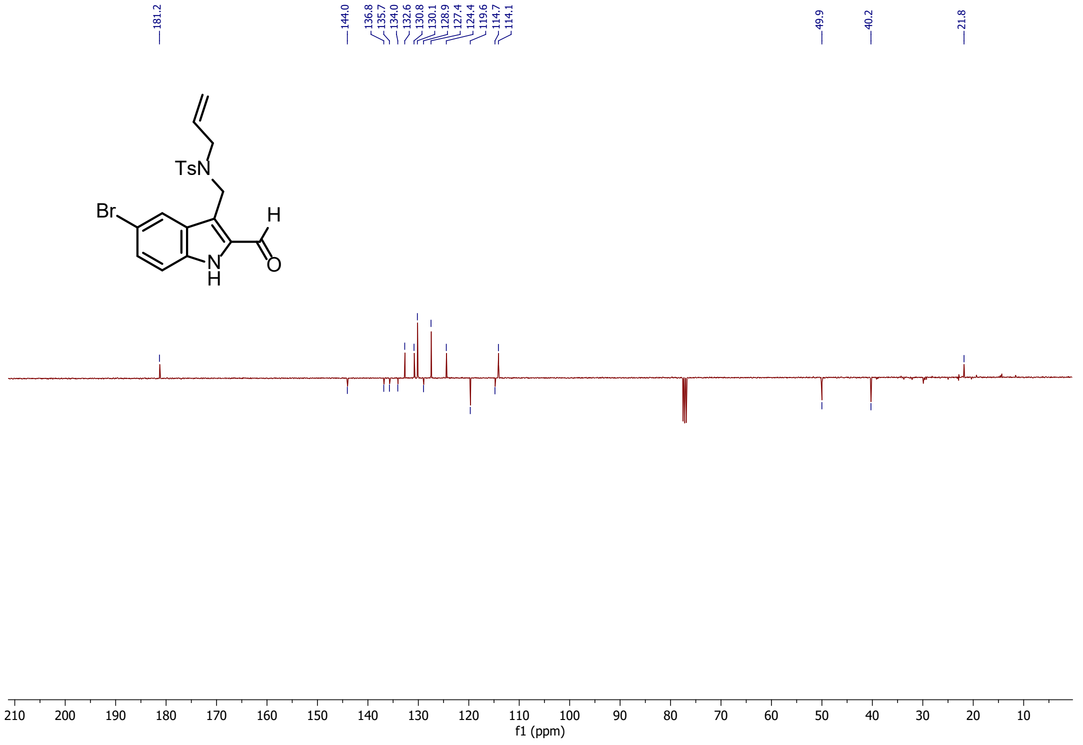

Figure 41: ^{13}C NMR (101 MHz, CDCl3) of SM-4d-5.

Methyl (*E*)-4-((*N*-((5-bromo-2-formyl-1*H*-indol-3-yl)methyl)-4-methylphenyl)sulfonamido)but-2-enoate (4d)

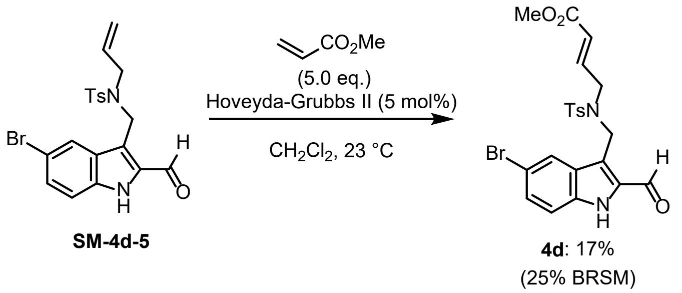

A 25 mL round-bottomed flask was charged **SM-4d-5** (170 mg, 0.38 mmol, 1.00 eq.) followed by CH_2Cl_2 (15 mL), methyl acrylate (160 µL, 1.90 mmol, 5.00 eq.) and Hoveyda-Grubbs 2nd generation catalyst (12 mg, 0.02 mmol, 5 mol%). The mixture was stirred for 18 h at 23 °C, before being concentrated under reduced pressure. The crude mixture was purified by flash chromatography using a gradient of heptanes/EtOAc to yield a purple solid (33 mg, 0.07 mmol, 17% yield, 25% BRSM).

^{1}H NMR (400 MHz, $CDCl_3$) δ 10.05 (s, 1H), 9.02 (br s, 1H), 7.78 – 7.73 (m, 2H), 7.46 (dd, *J* = 8.8, 1.8 Hz, 1H), 7.37 (d, *J* = 7.9 Hz, 2H), 7.28 (m, d, *J* = 8.9 Hz, 2H), 6.52 (dt, *J* = 15.7, 6.0 Hz, 1H), 5.64 (dt, *J* = 15.7, 1.6 Hz, 1H), 4.76 (s, 2H), 3.89 (dd, *J* = 6.0, 1.5 Hz, 2H), 3.65 (s, 3H), 2.47 (s, 3H).

^{13}C NMR (101 MHz, $CDCl_3$) δ 180.7, 165.7, 144.4, 142.2, 136.0, 135.4, 134.1, 130.9, 130.3 (2C), 128.9, 127.5 (2C), 124.1, 123.8, 118.7, 115.1, 114.1, 51.8, 48.3, 41.0, 21.8.

HRMS (ESI-TOF) m/z: $[M + Na]^+$ Calcd for $C_{22}H_{21}{}^{79}BrN_2O_5SNa$ 527.0247; Found 527.0244.

IR (neat) vmax: 3312, 2948, 2925, 2850, 2363, 1719, 1660, 1597, 1540, 1453, 1437, 1376, 1331, 1306, 1277.

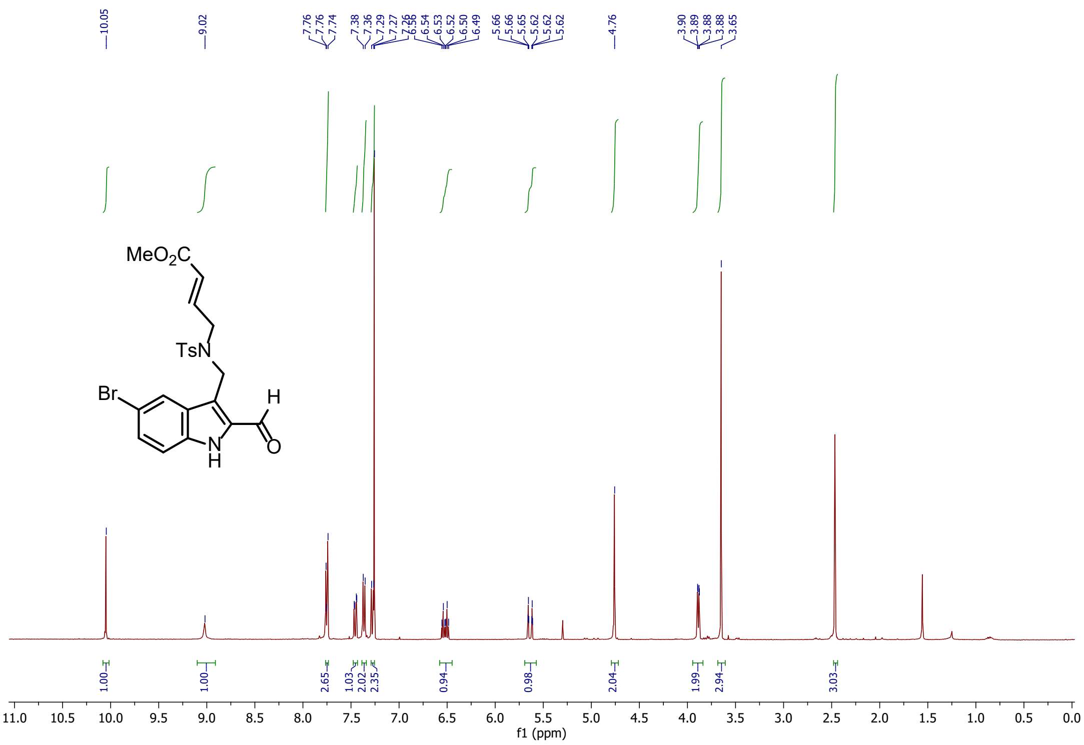

Figure 42: *[1]**H NMR (400 MHz, CDCl3) of 4d.***

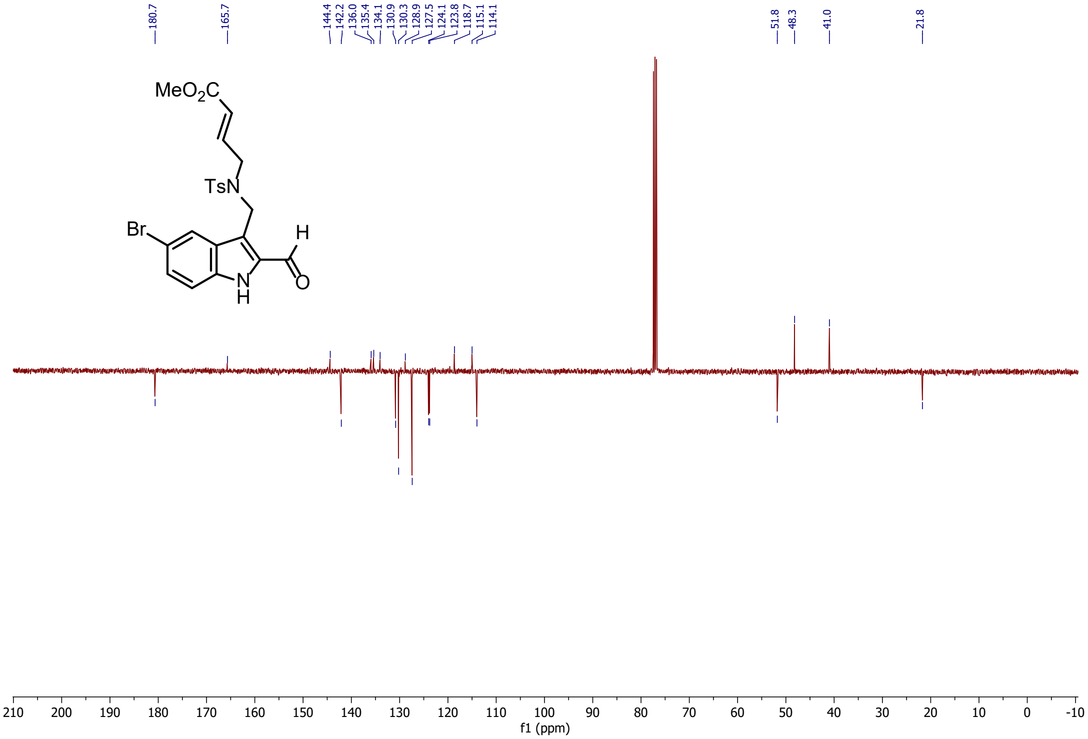

Figure 43: *[13]**C NMR (101 MHz, CDCl3) of 4d.***

h. Synthesis of methyl (*E*)-4-((*N*-((5-chloro-2-formyl-1*H*-indol-3-yl)methyl)-4-methylphenyl)sulfonamido)but-2-enoate (4e)

Ethyl 3-((allylamino)methyl)-5-chloro-1H-indole-2-carboxylate (SM-4e-2)

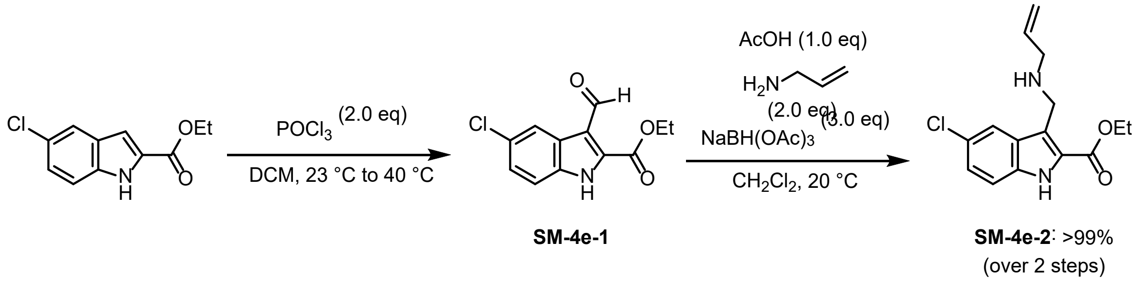

A 500 mL three-necked round-bottomed flask was charged with ethyl 5-chloro-1*H*-indole-2-carboxylate (5.00 g, 21.9 mmol, 1.00 eq.), CH_2Cl_2 (150 mL), and DMF (2.54 mL, 32.9 mmol, 1.50 eq.), and stirred at 23 °C for 10 min. $POCl_3$ (3.06 mL, 32.9 mmol, 1.50 eq.) was added dropwise at the same temperature, and the reaction was stirred under reflux for 20 h. After that, the crude was concentrated under reduced pressure, suspended in a saturated solution of NaOAc, and stirred for 4 h. The crude was then filtered, and washed with water and pentane to afford **SM-4e-1**, which was used in the next step without further purification.

In 250 mL round-bottomed flask, **SM-4e-1** (assumed pure, 1.95 g, 7.73 mmol, 1.00 eq) was dissolved in CH_2Cl_2 (26 mL) followed by the addition of allylamine (1.18 mL, 16 mmol, 2.0 eq), $NaBH(OAc)_3$ (4.91 g, 23.2 mmol, 3.00 eq) and AcOH (443 µL, 7.73 mmol, 1.00 eq). The mixture was stirred for 20 h at 20 °C, before being poured into a sat. aq. solution of Na_2CO_3 (100 mL) and diluted with CH_2Cl_2 (70 mL). The phases were separated and the aqueous phase was extracted twice more with CH_2Cl_2 (100 mL). The organic phases were combined, dried over Na_2SO_4 and filtered. The solution was concentrated under reduced pressure to cleanly yield **SM-4e-2** (2.26 g, 7.72 mmol, > 99% yield) as a pale yellow solid.

^{1}H NMR (600 MHz, CDCl$_3$) δ 9.18 (br s, 1H), 7.74 (d, *J* = 1.5 Hz, 1H), 7.28 (d, *J* = 8.7 Hz, 1H), 7.26 – 7.23 (m, 1H), 5.96 (ddt, *J* = 16.4, 10.4, 6.0 Hz, 1H), 5.20 (dd, *J* = 17.2, 1.4 Hz, 1H), 5.12 (dd, *J* = 10.2, 0.9 Hz, 1H), 4.43 (q, *J* = 7.1 Hz, 2H), 4.18 (s, 2H), 3.31 (d, *J* = 6.0 Hz, 2H), 1.43 (t, *J* = 7.1 Hz, 3H).

^{13}C NMR (151 MHz, CDCl$_3$) δ 161.9, 137.0, 134.1, 129.1, 126.4, 126.3, 125.4, 122.0, 120.4, 116.2, 113.1, 61.4, 52.0, 42.5, 14.5.

HRMS (ESI-TOF) m/z: $[M + H]^+$ Calcd for $C_{15}H_{18}ClN_2O_2$ 293.1051; Found 293.1051.

IR (neat) νmax: 1702, 1546, 1446, 1398, 1326, 1294, 1198, 1134, 1017.

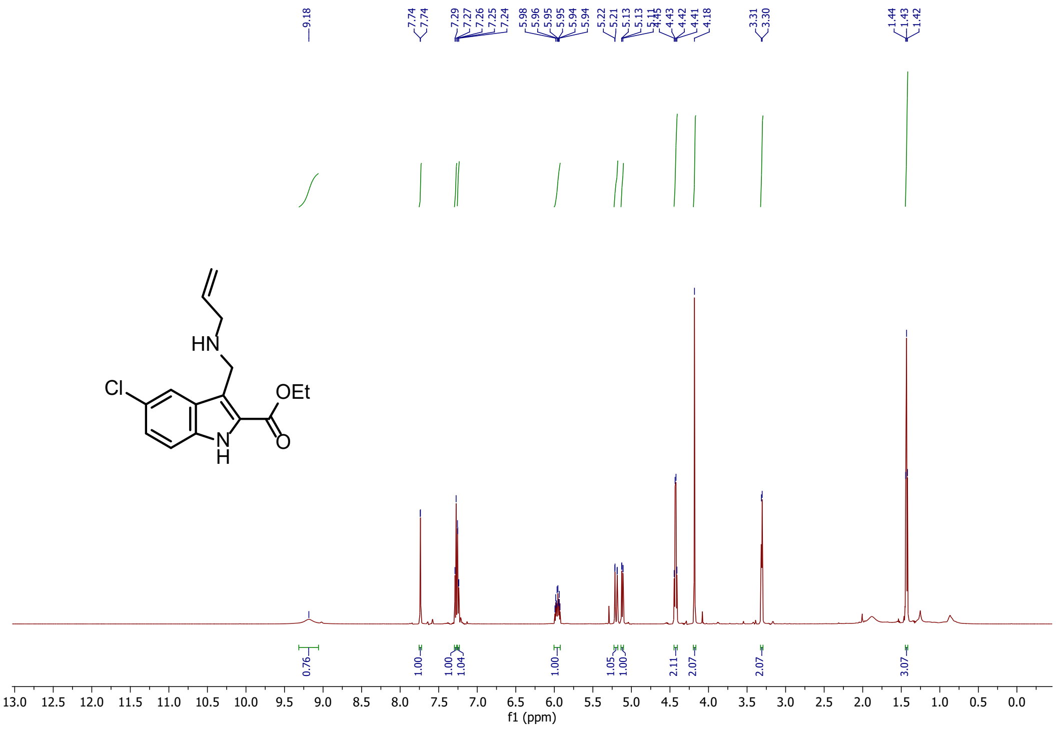

Figure 44: ***^{1}H NMR (600 MHz, $CDCl_3$) of SM-4e-2.***

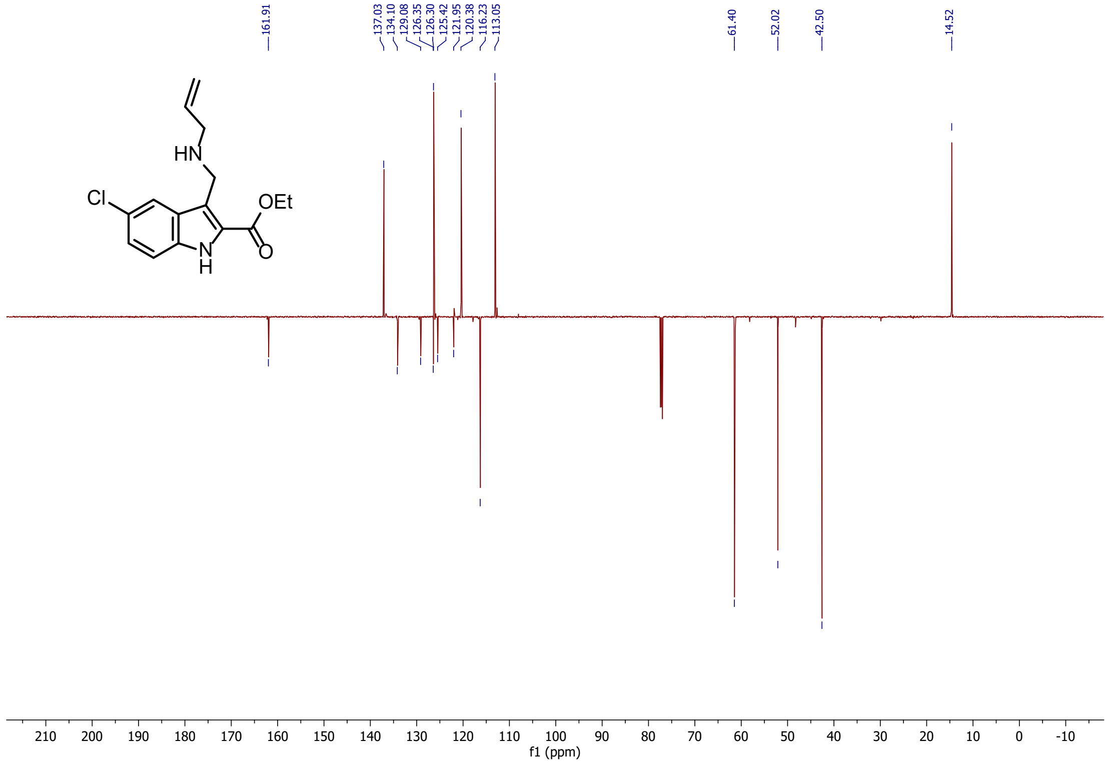

Figure 45: ***^{13}C NMR (151 MHz, $CDCl_3$) of SM-4e-2.***

Ethyl 3-(((*N*-allyl-4-methylphenyl)sulfonamido)methyl)-5-chloro-1H-indole-2-carboxylate (SM-4e-3)

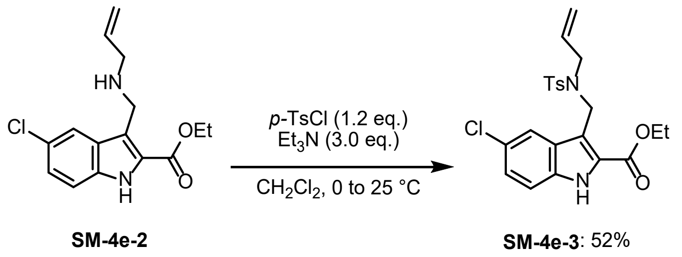

A Schlenk flask was charged with **SM-4e-2** (401 mg, 1.37 mmol, 1.00 eq.), CH_2Cl_2 (6 mL), and cooled to 0 °C. Et_3N (0.57 mL, 4.11 mmol, 3.00 eq.) and *p*-TsCl (313 mg, 1.64 mmol, 1.20 eq.) were then added. The mixture was stirred for 30 min at 20 °C, before being diluted with CH_2Cl_2 (25 mL) and a sat. aq. solution of NH_4Cl (25 mL). The phases were separated and the aqueous phase was extracted twice with CH_2Cl_2 (25 mL). The organic phases were combined, dried over Na_2SO_4 and filtered. The solution was concentrated under reduced pressure and the crude mixture was purified by flash chromatography using a gradient of heptanes/EtOAc to yield **SM-4e-3** as a white solid (319 mg, 0.714 mmol, 52% yield).

^{1}H NMR (700 MHz, $CDCl_3$) δ 8.79 (br s, 1H), 7.90 (s, 1H), 7.79 – 7.76 (m, 2H), 7.33 (d, *J* = 7.9 Hz, 2H), 7.29 – 7.28 (m, 2H), 5.45 (ddt, *J* = 16.5, 10.2, 6.2 Hz, 1H), 4.90 (s, 2H), 4.86 – 4.80 (m, 2H), 4.36 (q, *J* = 7.1 Hz, 2H), 3.71 (d, *J* = 6.2 Hz, 2H), 2.45 (s, 3H), 1.35 (t, *J* = 7.1 Hz, 3H).

^{13}C NMR (176 MHz, $CDCl_3$) δ 161.5, 143.6, 136.7, 134.0, 133.4, 129.9, 128.7, 127.6, 127.0, 126.8, 126.1, 121.5, 117.4, 117.0, 112.8, 61.5, 50.3, 42.3, 21.7, 14.4.

HRMS (ESI-TOF) m/z: $[M + Na]^+$ Calcd for $C_{22}H_{23}ClN_2O_4SNa$ 469.0959; Found 469.0958.

IR (neat) νmax: 3313, 1681, 1545, 1457, 1380, 1346, 1290, 1254, 1157, 1113, 1090, 1066, 1049.

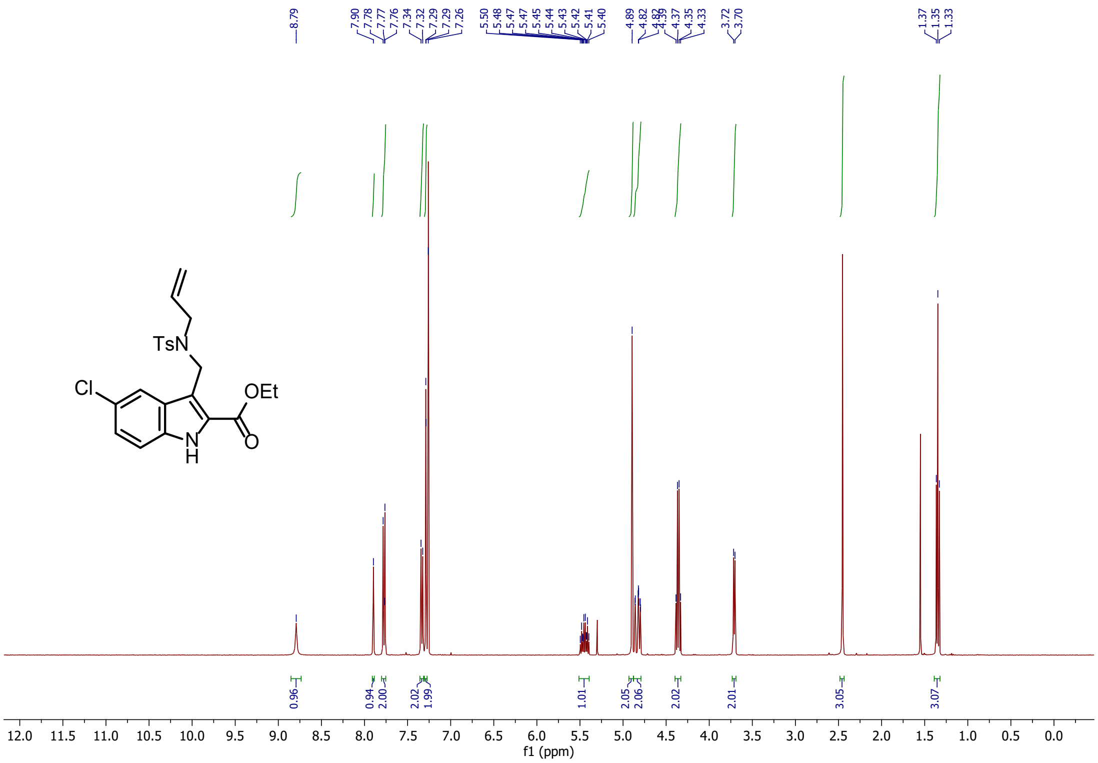

Figure 46: ***^{1}H NMR (700 MHz, $CDCl_3$) of SM-4e-3.***

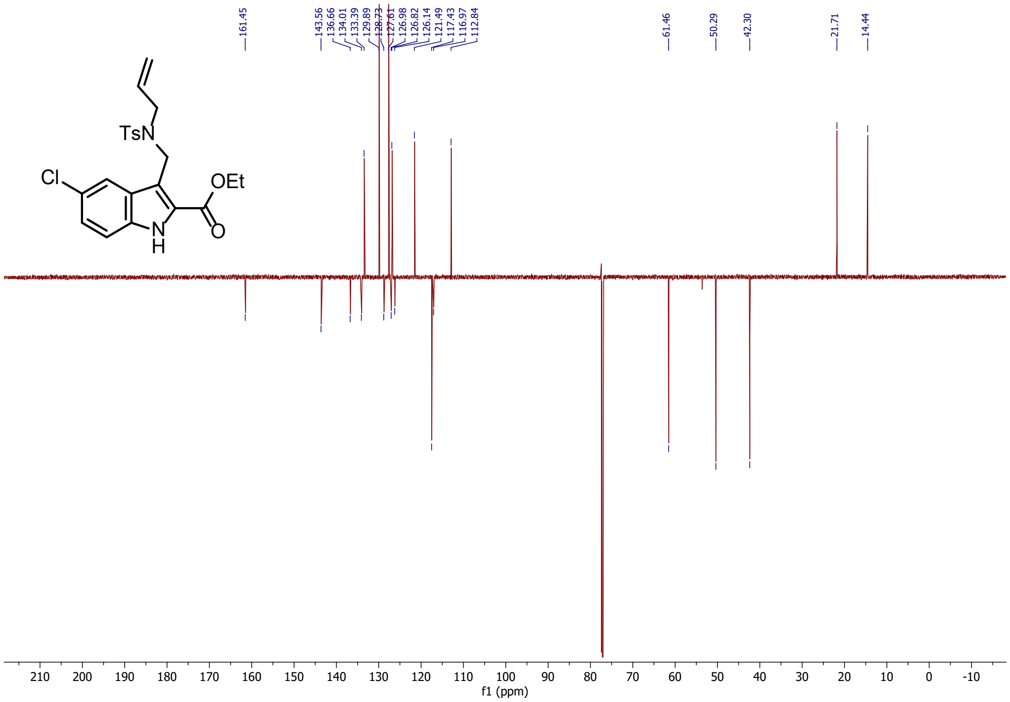

Figure 47: ***^{13}C NMR (176 MHz, $CDCl_3$) of SM-4e-3.***

N-allyl-*N*-((5-chloro-2-formyl-1*H*-indol-3-yl)methyl)-4-methylbenzenesulfonamide (SM-4e-5)

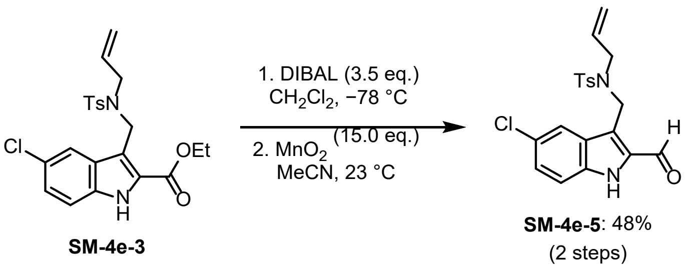

A 10 mL Schlenk flask was charged with **SM-4e-3** (258 mg, 0.577 mmol, 1.00 eq.), CH_2Cl_2 (3 mL) and the mixture was cooled to −78 °C. DIBAL (1 mol/L in PhMe, 2.02 mL, 2.02 mmol, 3.50 eq.) was added dropwise, and the crude stirred for 1 h at −78 °C. After this time, the mixture was quenched by the addition of sat. aq. solution of Rochelle salt (3 mL), allowed to warm to room temperature and stirred for 1 h. The resulting suspension was extracted three times with CH_2Cl_2 (25 mL), dried over $MgSO_4$, filtered and concentrated under reduced pressure, yielding **SM-4e-4**, which was used in the next step without further purification.

A 10 mL Schlenk flask was charged with **SM-4e-4** (258 mg, 0.577 mmol, 1.00 eq.), MeCN (3 mL) and MnO_2 (753 mg, 8.66 mmol, 15.0 eq.). The mixture was stirred at 23 °C for 21 h, before being filtered over Celite, eluted with CH_2Cl_2 and concentrated under reduced pressure. The crude mixture was purified by flash chromatography using a gradient of heptanes/EtOAc to yield **SM-4e-5** as a white solid (112 mg, 0.278 mmol, 48% over 2 steps).

^{1}H NMR (700 MHz, $CDCl_3$) δ 10.05 (s, 1H), 8.95 (br s, 1H), 7.78 – 7.75 (m, 2H), 7.68 – 7.67 (m, 1H), 7.36 – 7.32 (m, 4H), 5.49 (ddt, *J* = 16.6, 10.2, 6.3 Hz, 1H), 5.05 (dd, *J* = 10.2, 1.2 Hz, 1H), 4.97 – 4.93 (m, 1H), 4.78 (s, 2H), 3.79 (d, *J* = 6.3 Hz, 2H), 2.46 (s, 3H).

^{13}C NMR (176 MHz, $CDCl_3$) δ 180.9, 143.9, 136.7, 135.1, 134.1, 132.5, 130.0, 128.2, 128.2, 127.3, 127.2, 121.2, 119.4, 113.4, 49.8, 40.1, 21.6.

HRMS (ESI-TOF) m/z: $[M + Na]^+$ Calcd for $C_{20}H_{19}ClN_2O_3SNa$ 425.0697; Found 425.0697.

IR (neat) νmax: 3293, 2981, 1713, 1598, 1494, 1438, 1416, 1374, 1320, 1228, 1155, 1106.

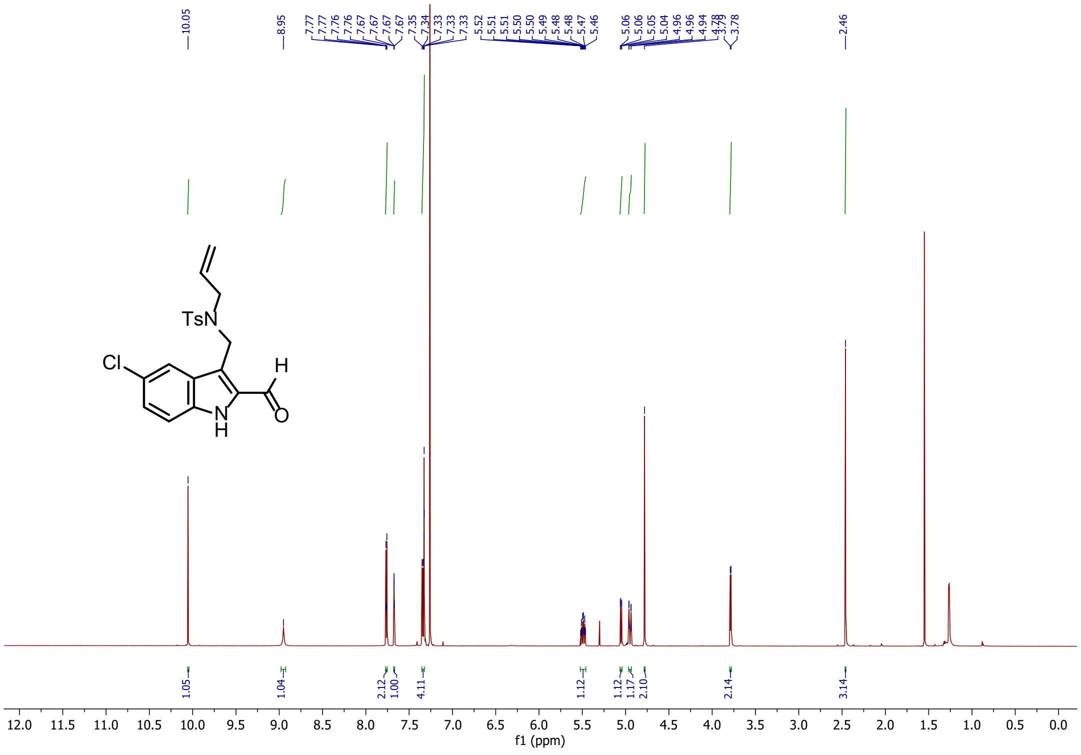

Figure 48: ***^{1}H NMR (700 MHz, CDCl₃) of SM-4e-5.***

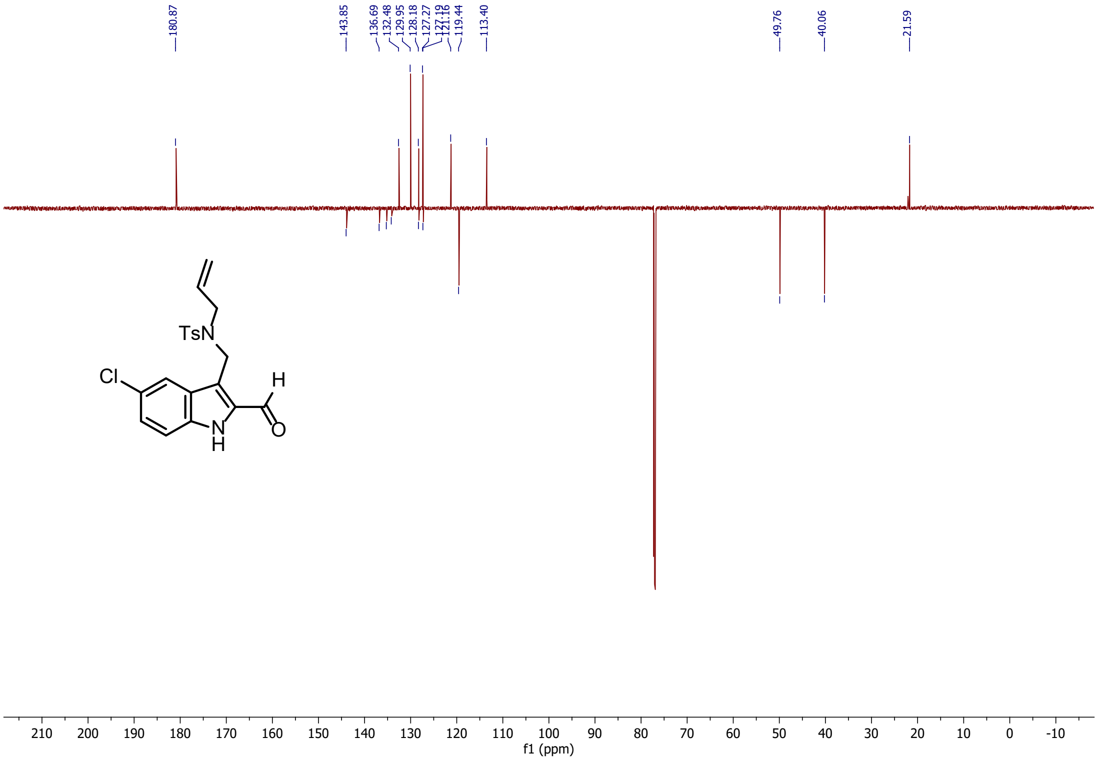

Figure 49: ***^{13}C NMR (176 MHz, CDCl₃) of SM-4e-5***

Methyl (*E*)-4-((*N*-((5-chloro-2-formyl-1*H*-indol-3-yl)methyl)-4-methylphenyl)sulfonamido)but-2-enoate (4e)

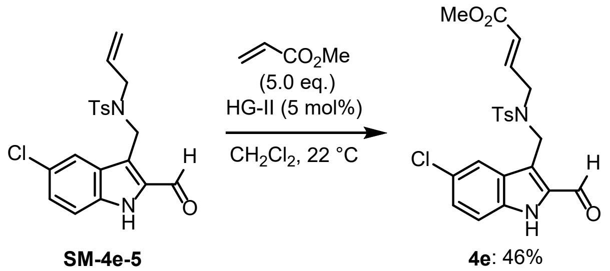

A Schlenk flask was charged with **SM-4e-5** (112 mg, 0.278 mmol, 1.00 eq.), CH_2Cl_2 (1 mL), methyl acrylate (0.13 mL, 1.4 mmol, 5.0 eq.) and Hoveyda-Grubbs 2nd generation catalyst (8.7 mg, 0.013 mmol, 5.0 mol%). The mixture was stirred for 18 h at 22 °C, before being concentrated under reduced pressure. The crude mixture was purified by flash chromatography using a gradient of heptanes/EtOAc to yield **4e** as a light-purple solid (59 mg, 0.19 mmol, 46% yield, 52% BRSM).

^{1}H NMR (600 MHz, CDCl₃) δ 10.04 (s, 1H), 9.08 (br s, 1H), 7.75 (d, *J* = 8.1 Hz, 2H), 7.59 (s, 1H), 7.36 (d, *J* = 8.0 Hz, 2H), 7.33 (d, *J* = 0.6 Hz, 2H), 6.52 (dt, *J* = 15.7, 6.0 Hz, 1H), 5.63 (d, *J* = 15.7 Hz, 1H), 4.77 (s, 2H), 3.89 (d, *J* = 5.1 Hz, 2H), 3.65 (s, 3H), 2.47 (s, 3H).

^{13}C NMR (151 MHz, $CDCl_3$) δ 180.7, 165.7, 144.4, 142.2, 136.1, 135.2, 130.3, 128.4, 128.3, 127.6, 127.5, 123.8, 120.9, 118.9, 113.7, 77.2, 51.8, 48.3, 41.1, 21.8.

HRMS (ESI-TOF) m/z: $[M + Na]^+$ Calcd for $C_{22}H_{21}ClN_2O_5SNa$ 483.0752; Found 483.0748.

IR (neat) vmax: 3300, 1732, 1651, 1542, 1455, 1427, 1356, 1319, 1242, 1157, 1116, 1086, 1039.

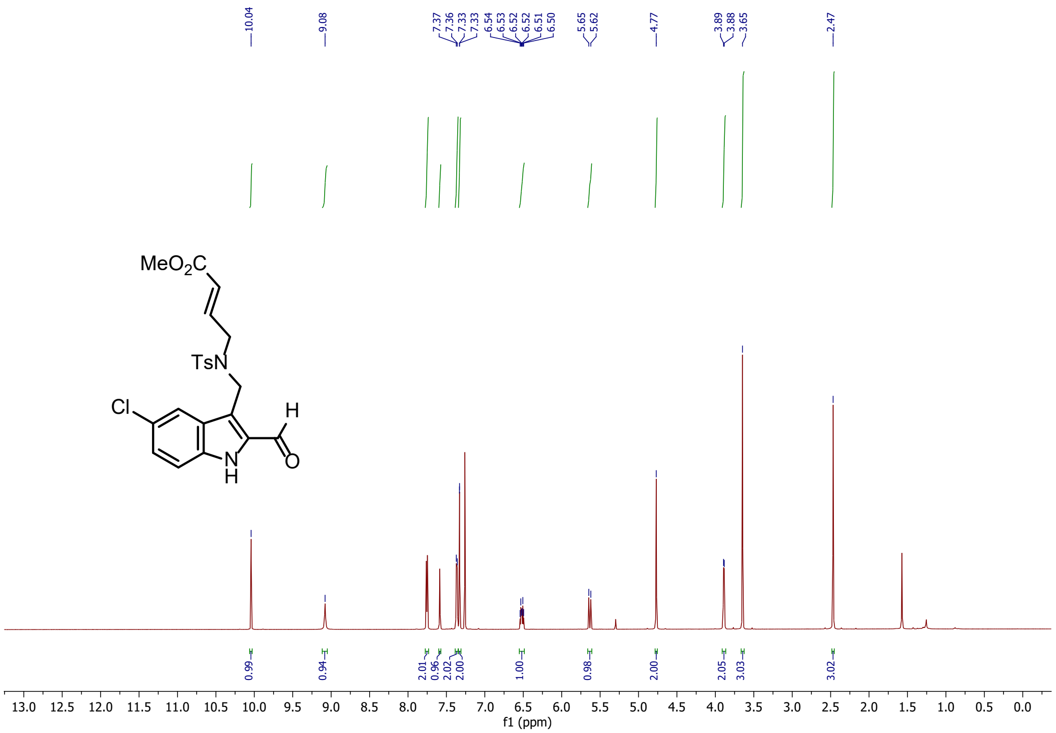

Figure 50: ***¹H NMR (600 MHz, CDCl₃) of 4e.***

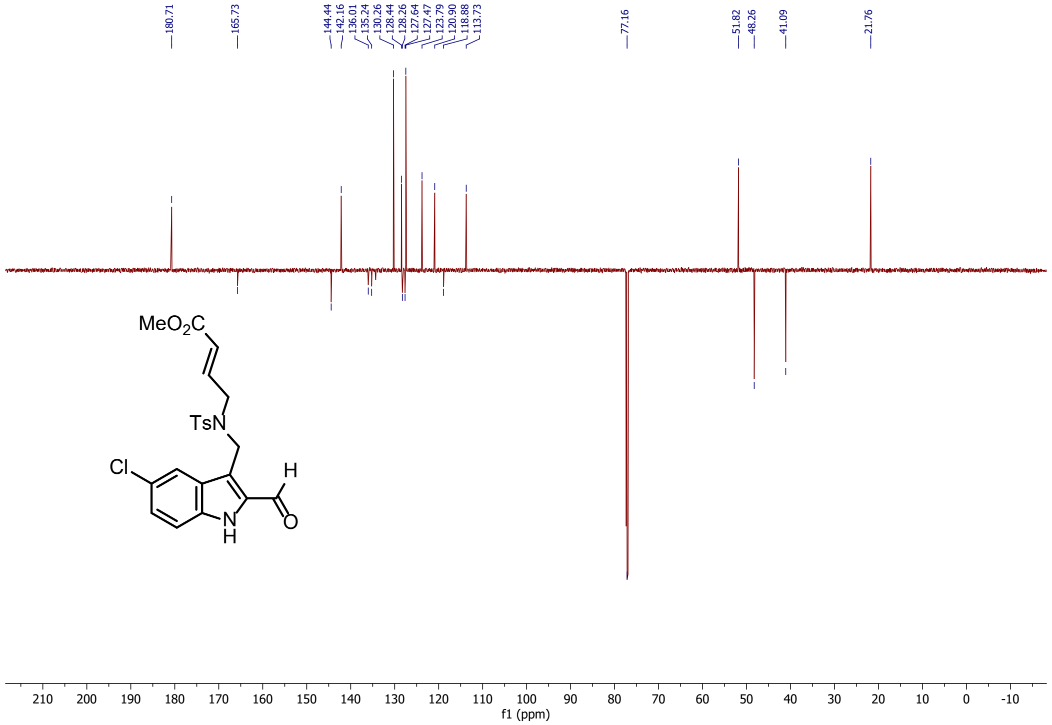

Figure 51: ***¹³C NMR (151 MHz, CDCl₃) of 4e.***

i. Synthesis of Methyl (*E*)-4-((2-formyl-1*H*-indol-3-yl)methoxy)but-2-enoate (4f)

Ethyl 3-(hydroxymethyl)-1*H*-indole-2-carboxylate (SM-4f-1)

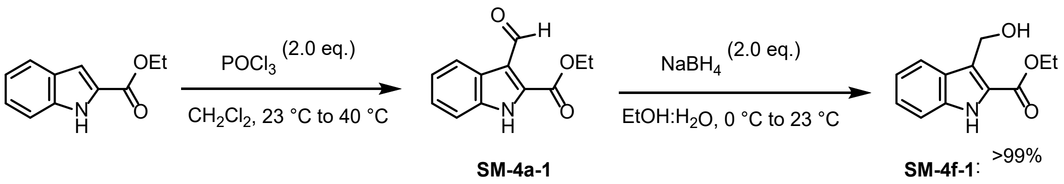

A 1 L three-necked round-bottomed flask was charged with ethyl 5-chloro-1*H*-indole-2-carboxylate (10.0 g, 52.9 mmol, 1.00 eq.), CH_2Cl_2 (350 mL), and DMF (6.13 mL, 79.3 mmol, 1.50 eq.), and stirred at 23 °C for 10 min. $POCl_3$ (7.39 mL, 79.3 mmol, 1.50 eq.) was added dropwise at the same temperature, and the reaction was stirred under reflux for 20 h. After that, the crude was concentrated under reduced pressure, suspended in a saturated solution of NaOAc, and stirred for 4 h. The crude was then filtered, and washed with water (20 mL) and pentane (20 mL) to afford **SM-4a-1**, which was used in the next step without further purification.

A 2 L round-bottomed flask was charged with **SM-4a-1** (11.5 g, 52.9 mmol, 1.00 eq.), EtOH (670 mL) and H_2O (200 mL). The suspension was cooled to 0 °C before the addition of $NaBH_4$ (4.00 g, 106 mmol, 2.00 eq.). The resulting mixture was allowed to warm to room temperature, and stirred for 1 h. The EtOH was removed under reduced pressure, and the resulting aqueous phase was extracted three times with EtOAc (150 mL), dried over $MgSO_4$, filtered and concentrated under reduced pressure, affording **SM-4f-1** as a white solid (11.6 g, 52.8 mmol, >99%). The analytical data is in accordance to that previously reported.[5]

^{1}H NMR (400 MHz, $CDCl_3$) δ 8.82 (br s, 1H), 7.78 (d, *J* = 7.9 Hz, 1H), 7.42 – 7.33 (m, 2H), 7.22 – 7.17 (m, 1H), 5.09 (s, 2H), 4.46 (q, *J* = 7.1 Hz, 2H), 3.38 (br s, 1H), 1.45 (t, *J* = 7.1 Hz, 3H).

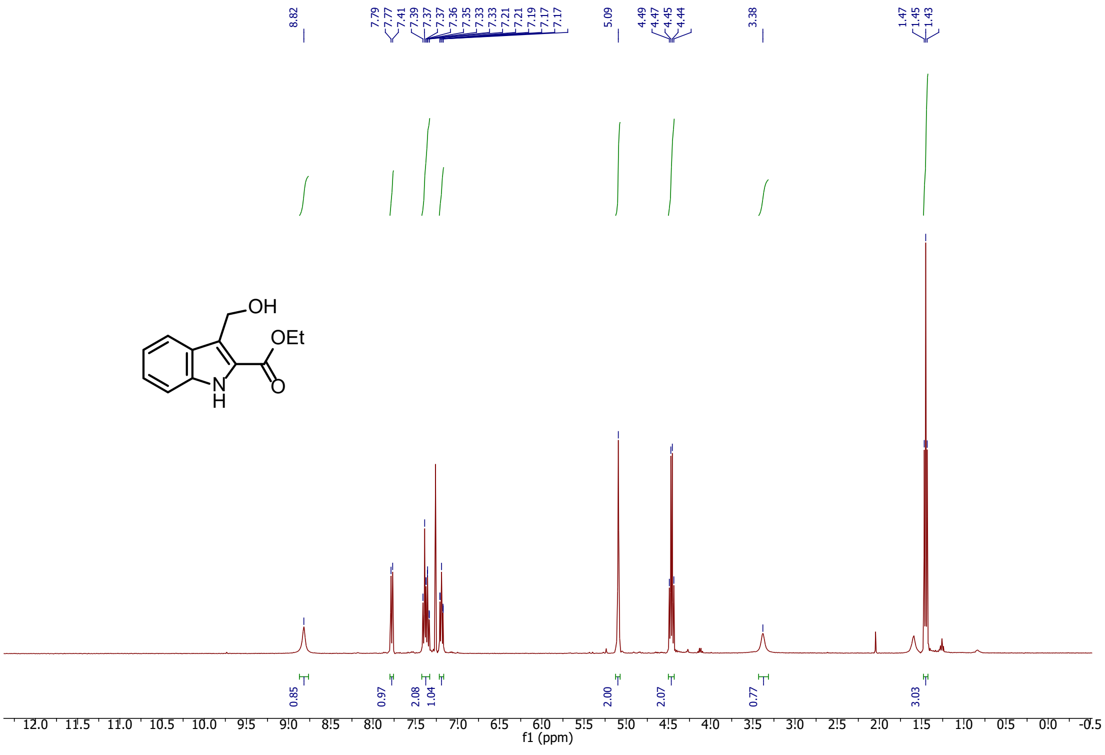

Figure 52: ***^{1}H NMR (400 MHz, $CDCl_3$) of SM-4f-1.***

Ethyl 3-((allyloxy)methyl)-1*H*-indole-2-carboxylate (SM-4f-3)

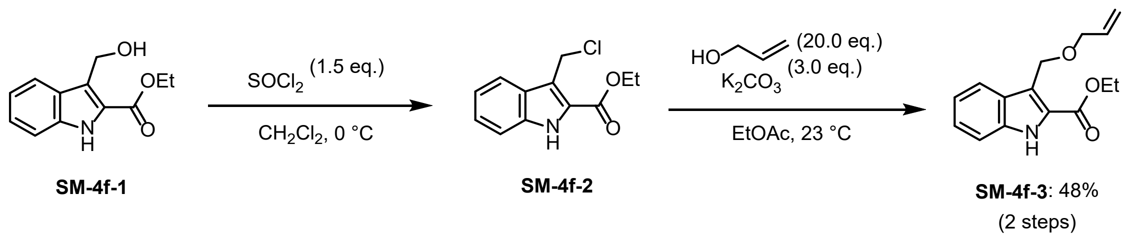

A 500 mL Schlenk-flask was charged with **SM-4f-1** (3.00 g, 13.7 mmol, 1.00 eq.), CH_2Cl_2 (100 mL), and was cooled to 0 °C. $SOCl_2$ (1.5 mL, 21 mmol, 1.5 eq.) was added dropwise to the mixture, which was then left stirring at the same temperature for 3 h. The solvent was then removed under reduced pressure, yielding **SM-4f-2** which was used in the next step without further purification.

A 250 mL three-necked round-bottomed was charged with allyl alcohol (7.78 mL, 114 mmol, 20.0 eq.), K_2CO_3 (2.37 g, 17.2 mmol, 3.00 eq.), and dry EtOAc (25 mL). To the mixture, **SM-4f-2** (1.36 g, 5.72 mmol, 1.00 eq.) was added dropwise over 2 h as a solution in dry EtOAc (25 mL). The resulting crude was left stirring for 12 h, after which it was washed with sat. aq. solution of NH_4Cl (40 mL) and a sat. aq. solution of brine (40 mL). The organic phase was dried over $MgSO_4$, filtered and concentrated under reduced pressure. The crude was purified by flash chromatography using a gradient of heptane/EtOAc to yield **SM-4f-3** as an off-white solid (710 mg, 2.74 mmol, 48% yield over 2 steps).

^{1}H NMR (600 MHz, $CDCl_3$) δ 8.89 (br s, 1H), 7.90 (d, *J* = 8.1 Hz, 1H), 7.39 (d, *J* = 8.3 Hz, 1H), 7.35 – 7.32 (m, 1H), 7.19 – 7.16 (m, 1H), 5.99 (ddt, *J* = 11.2, 10.6, 5.6 Hz, 1H), 5.34 – 5.29 (m, 1H), 5.20 (dd, *J* = 10.4, 1.4 Hz, 1H), 5.10 (s, 2H), 4.44 (q, *J* = 7.1 Hz, 2H), 4.11 – 4.07 (m, 2H), 1.44 (t, *J* = 7.1 Hz, 3H).

^{13}C NMR (151 MHz, $CDCl_3$) δ 162.2, 135.9, 135.2, 128.1, 125.9, 124.5, 122.0, 120.9, 119.9, 117.1, 111.8, 71.3, 63.1, 61.2, 14.6.

HRMS (ESI-TOF) m/z: $[M + Na]^+$ Calcd for $C_{15}H_{17}NO_3Na$ 282.1101; Found 282.1101.

IR (neat) vmax: 3334, 2361, 2337, 1739, 1698, 1558, 1378, 1247, 1133, 1068.

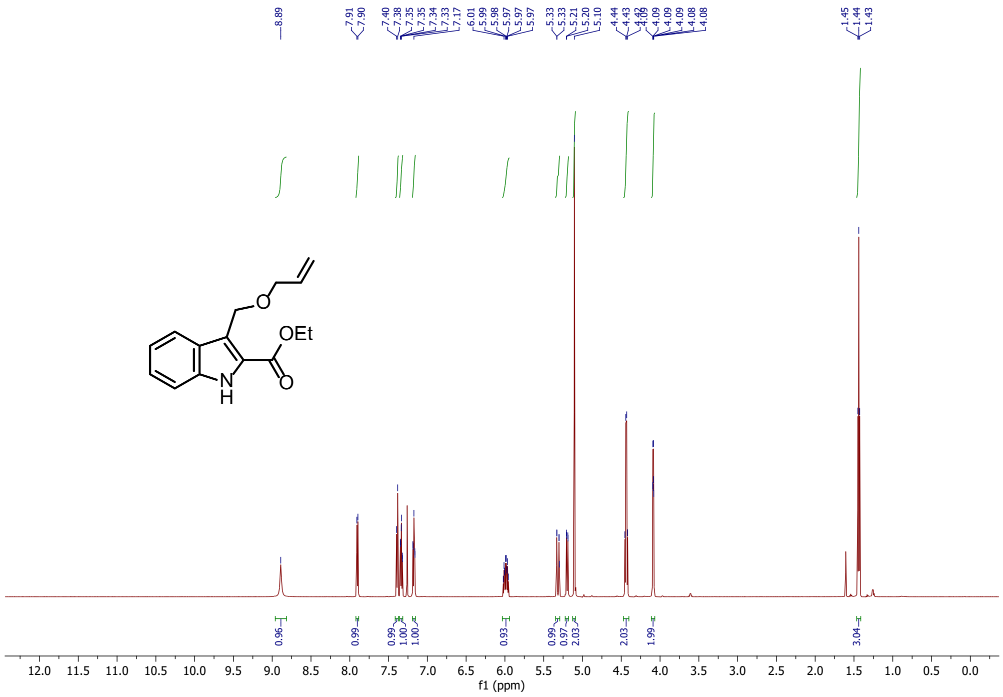

*Figure 53: [1]**H NMR (600 MHz, CDCl₃) of SM-4f-3.***

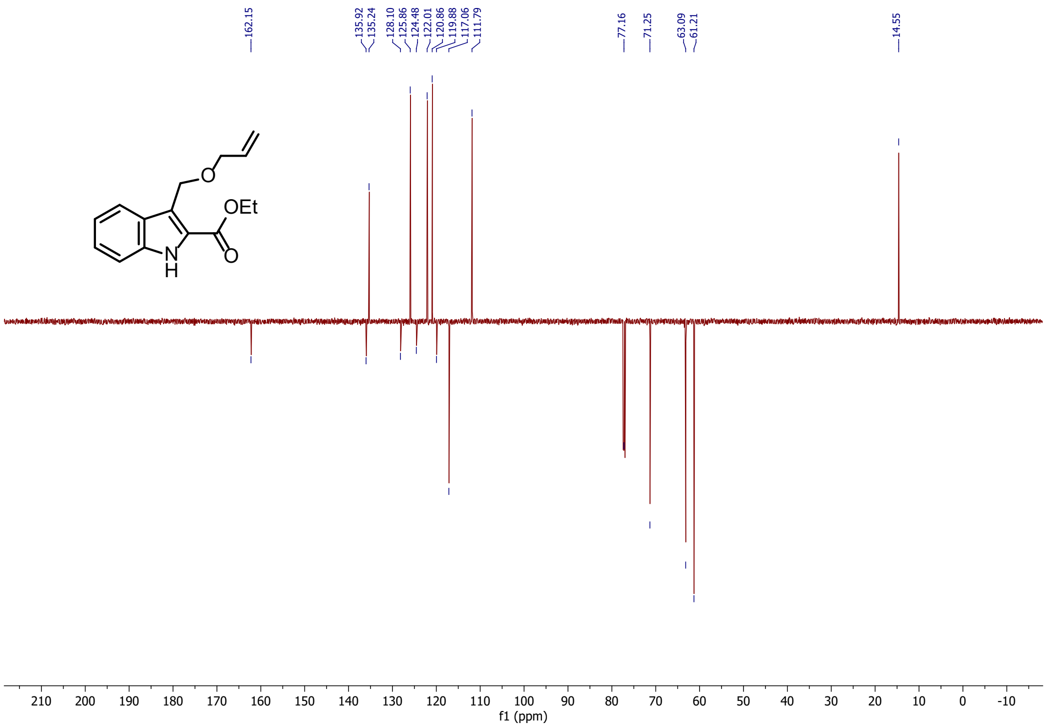

*Figure 54: [13]**C NMR (151 MHz, CDCl₃) of SM-4f-3.***

3-((allyloxy)methyl)-1*H*-indole-2-carbaldehyde (SM-4f-5)

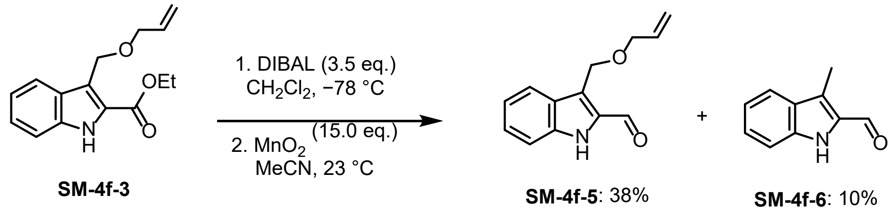

A 50 mL Schlenk flask was charged with **SM-4f-3** (599 mg, 2.31 mmol, 1.00 eq.), CH_2Cl_2 (11.5 mL) and the mixture was cooled to −78 °C. DIBAL (1 mol/L in PhMe, 8.09 mL, 8.09 mmol, 3.50 eq.) was added dropwise, and the crude stirred for 1 h at −78 °C. After this time, the mixture was quenched by the addition of sat. aq. solution of Rochelle salt (10 mL), allowed to warm up to room temperature and stirred for 4 h. The resulting suspension was extracted three times with CH_2Cl_2 (50 mL), dried over $MgSO_4$, filtered and concentrated under reduced pressure, yielding **SM-4f-4**, which was used without further purification.

A 25 mL Schlenk flask was charged with **SM-4f-4** (502 mg, 2.31 mmol, 1.00 eq), MeCN (11.5 mL) and MnO_2 (3.01 g, 34.6 mmol, 15.0 eq.). The mixture was stirred at 23 °C for 21 h, before being filtered over Celite, eluted with CH_2Cl_2 and concentrated under reduced pressure. The crude mixture was purified by flash chromatography using a gradient of heptane/EtOAc to yield **SM-4f-5** (190 mg, 0.88 mmol, 38% over 2 steps) and **SM-4f-6** (36 mg, 0.23 mmol, 10%), as off-white solids.

SM-4f-5

^{1}H NMR (600 MHz, $CDCl_3$) δ 10.16 (s, 1H), 9.00 (br s, 1H), 7.80 (d, *J* = 8.1 Hz, 1H), 7.43 – 7.38 (m, 2H), 7.21 – 7.17 (m, 1H), 5.98 (ddd, *J* = 22.7, 10.9, 5.7 Hz, 1H), 5.36 – 5.29 (m, 1H), 5.25 (d, *J* = 10.4 Hz, 1H), 5.03 (s, 2H), 4.13 (d, *J* = 5.6 Hz, 2H).

^{13}C NMR (151 MHz, $CDCl_3$) δ 181.8, 137.2, 134.6, 133.1, 127.7, 127.3, 123.8, 121.9, 121.3, 117.9, 112.4, 71.6, 62.3.

HRMS (ESI-TOF) m/z: $[M + Na]^+$ Calcd for $C_{13}H_{13}NO_2Na$ 238.0838; Found 238.0838.

IR (neat) vmax: 3305, 2853, 1644, 1541, 1457, 1364, 1265, 1226, 1113, 1064.

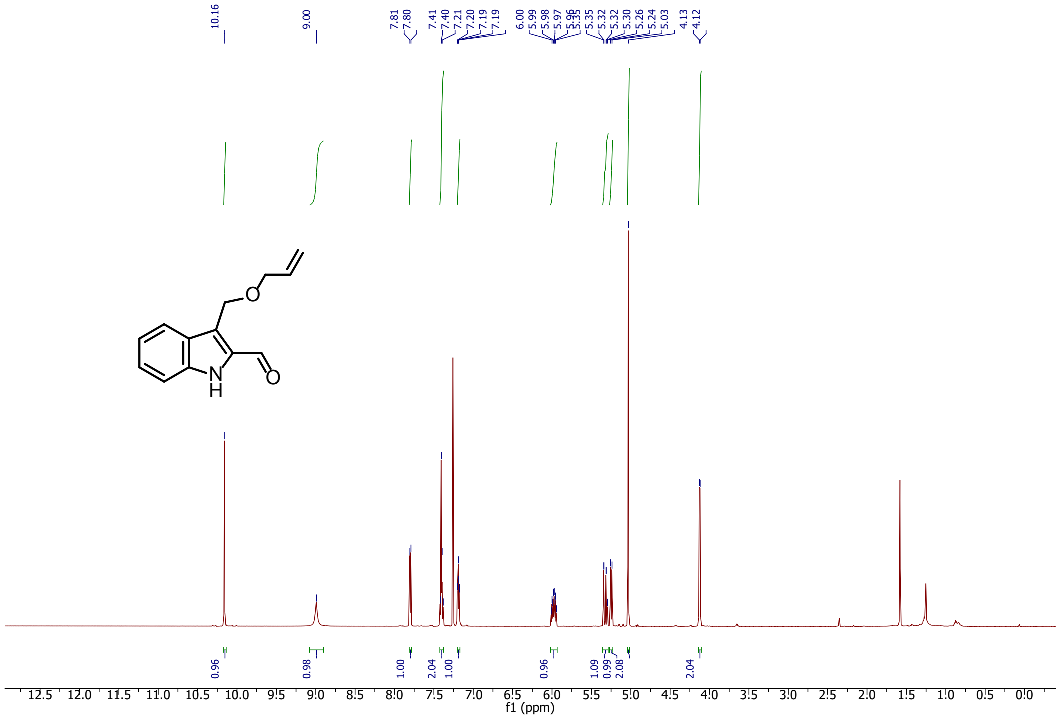

Figure 55: ***^{1}H NMR (600 MHz, $CDCl_3$) of SM-4f-5.***

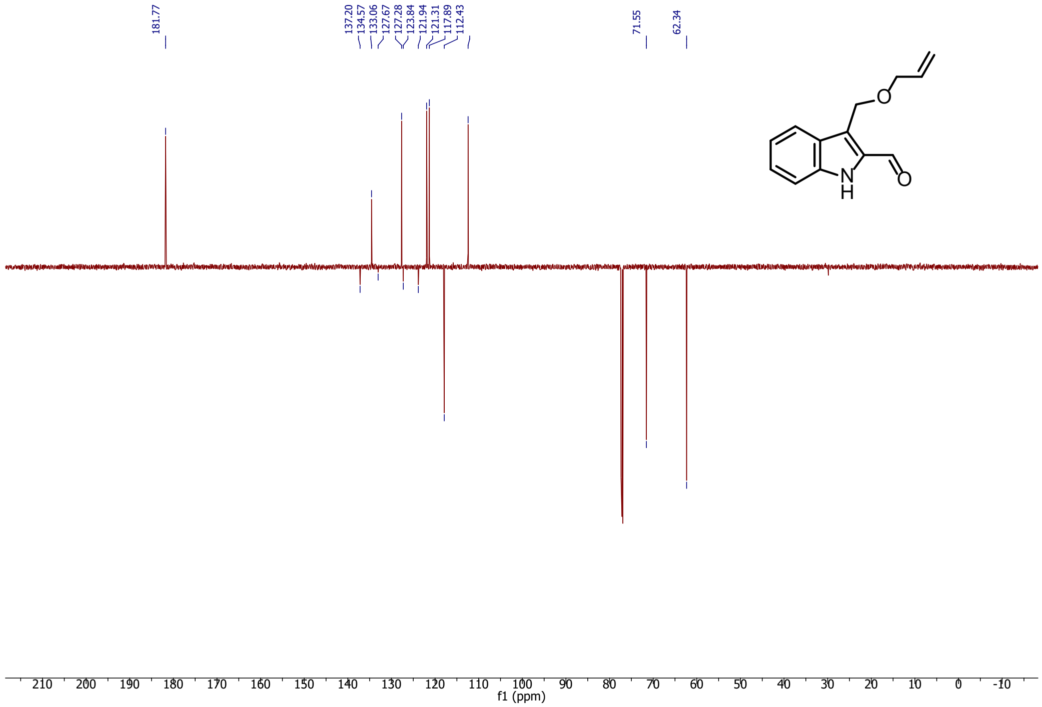

Figure 56: ***^{13}C NMR (151 MHz, $CDCl_3$) of SM-4f-5.***

SM-4f-6

The analytical data is in accordance to that previously reported.[6]

^{1}H NMR (400 MHz, CDCl$_3$) δ 10.04 (s, 1H), 8.70 (br, 1H), 7.71 (d, *J* = 8.4 Hz, 1H), 7.42 – 7.35 (m, 2H), 7.16 (ddd, *J* = 8.0, 5.8, 2.1 Hz, 1H), 2.65 (s, 3H).

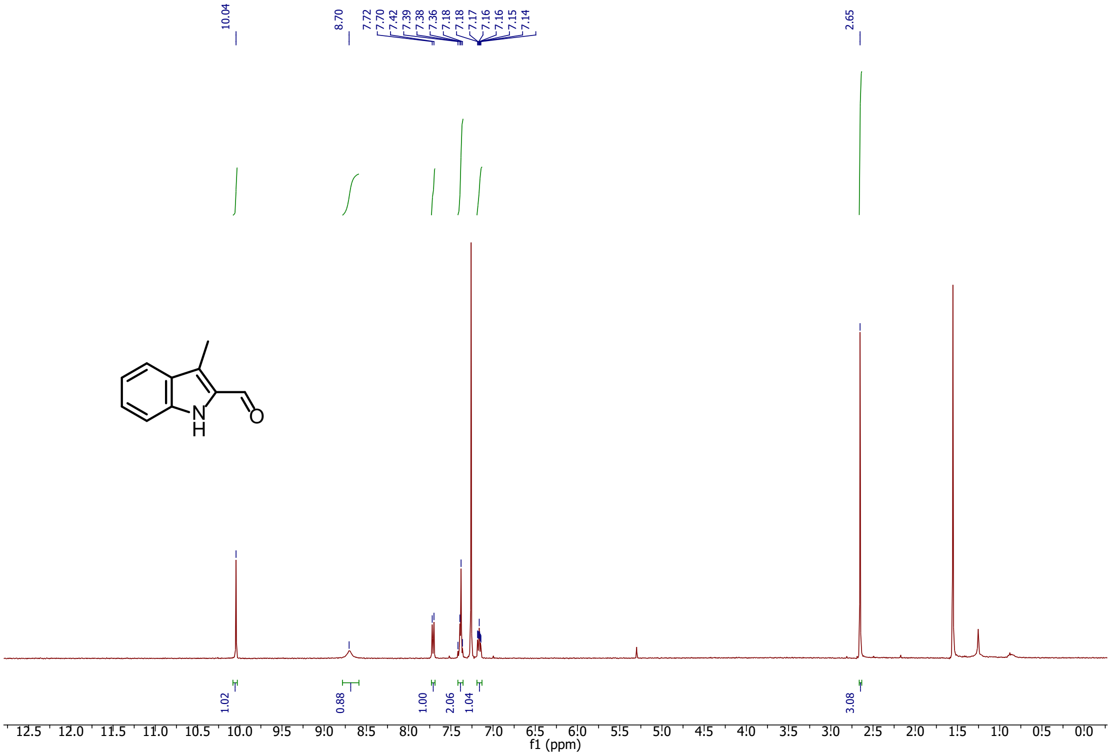

Figure 57: ***^{1}H NMR (400 MHz, CDCl$_3$) of SM-4f-6.***

Methyl (*E*)-4-((2-formyl-1*H*-indol-3-yl)methoxy)but-2-enoate (4f)

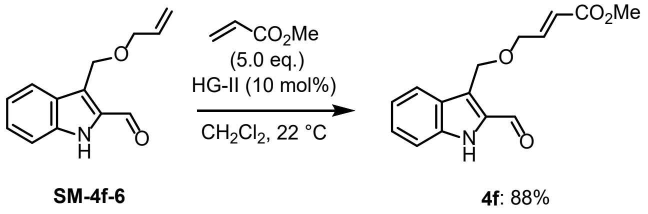

A Schlenk flask was charged with **SM-4f-6** (190 mg, 0.88 mmol, 1.0 eq.), CH_2Cl_2 (5 mL), methyl acrylate (0.40 mL, 4.4 mmol, 5.0 eq.) and Hoveyda-Grubbs 2nd generation catalyst (55 mg, 0.088 mmol, 10 mol%). The mixture was stirred for 10 h at 22 °C, before being concentrated under reduced pressure. The crude mixture was purified by flash chromatography using a gradient of heptanes/EtOAc to yield **4f** as a light-purple solid (212 mg, 0.780 mmol, 88% yield).

^{1}H NMR (600 MHz, $CDCl_3$) δ 10.15 (s, 1H), 9.14 (br s, 1H), 7.79 (d, *J* = 8.1 Hz, 1H), 7.42 (ddd, *J* = 10.7, 9.1, 4.5 Hz, 2H), 7.22 – 7.18 (m, 1H), 7.00 (dt, *J* = 15.8, 4.4 Hz, 1H), 6.12 (dt, *J* = 15.8, 1.9 Hz, 1H), 5.08 (s, 2H), 4.27 (dd, *J* = 4.4, 1.9 Hz, 2H), 3.75 (s, 3H).

^{13}C NMR (151 MHz, $CDCl_3$) δ 181.4, 166.7, 144.1, 137.2, 133.1, 127.8, 127.2, 123.1, 121.8, 121.5 (2C), 112.6, 69.00, 63.2, 51.8.

HRMS (ESI-TOF) m/z: $[M + Na]^+$ Calcd for $C_{15}H_{15}NO_4Na$ 296.0893; Found 296.0887.

IR (neat) νmax: 3306, 2924, 1716, 1383, 1285, 1225, 1153, 1059, 1015, 1006.

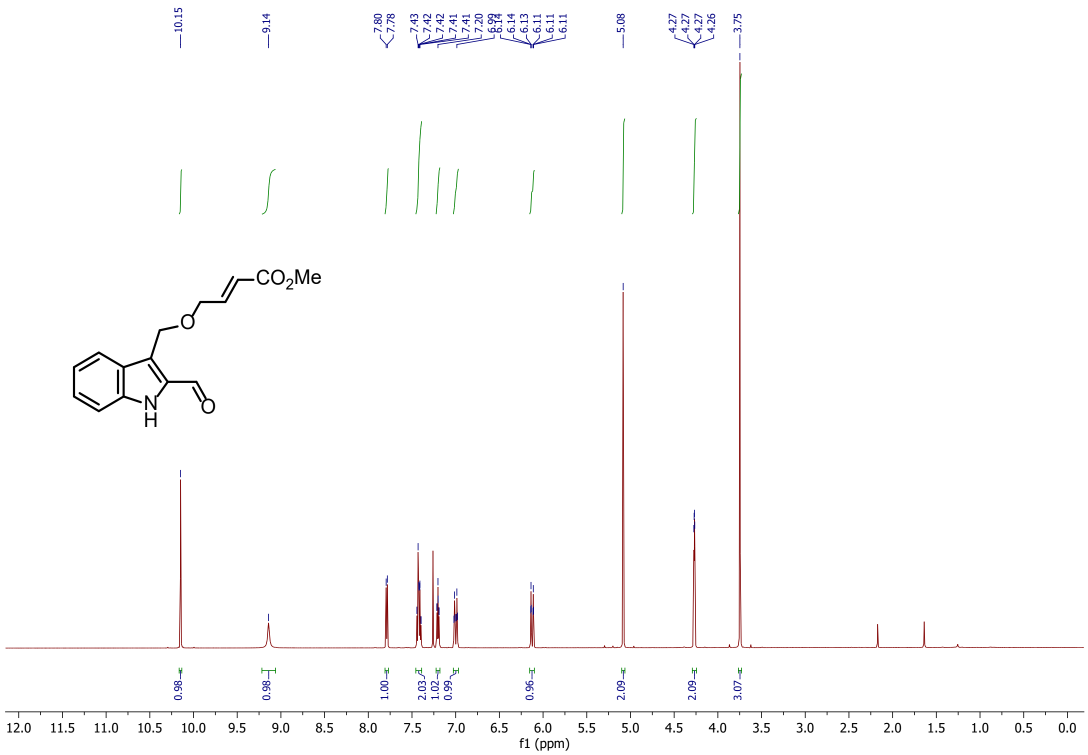

*Figure 58: **¹H NMR (600 MHz, CDCl₃) of 4f.***

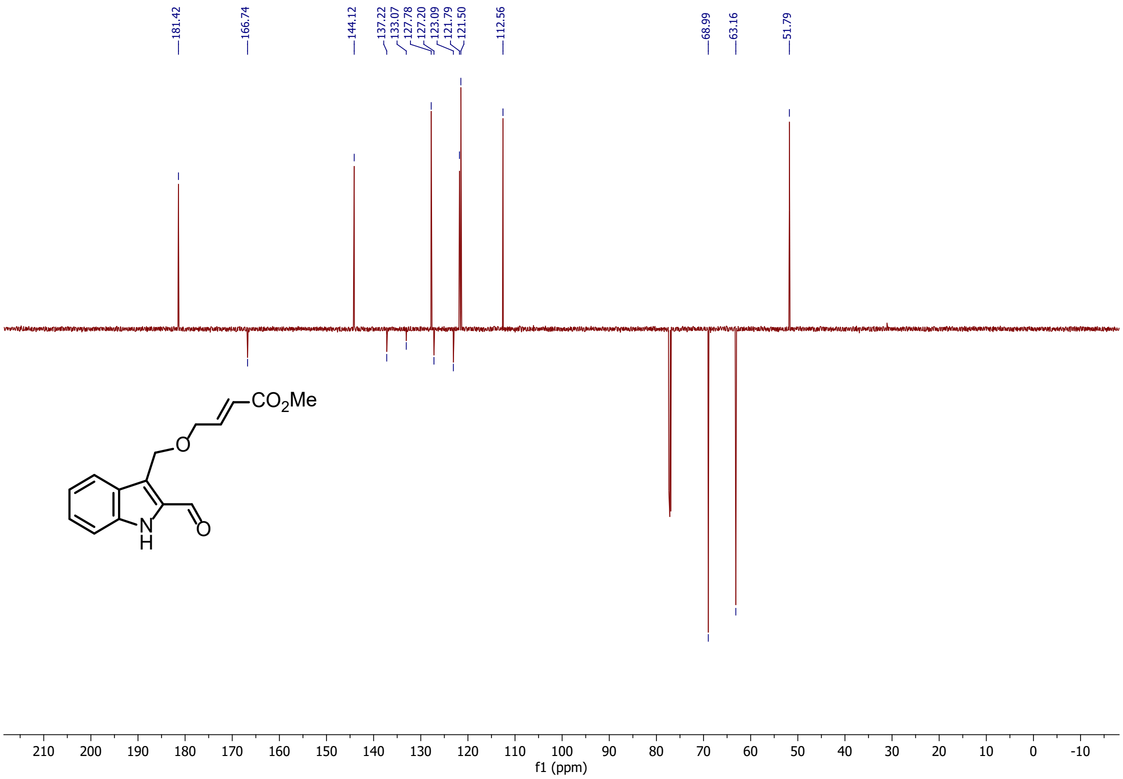

*Figure 59: **¹³C NMR (151 MHz, CDCl₃) of 4f.***

j. Synthesis of methyl (*E*)-4-((*N*-((2-formyl-1H-indol-3-yl)methyl)-2,4,6-tris(trifluoromethyl)phenyl)sulfonamido)but-2-enoate (4g)

Ethyl 3-(((*N*-allyl-2,4,6-tris(trifluoromethyl)phenyl)sulfonamido)methyl)-1*H*-indole-2-carboxylate (SM-4g-2)

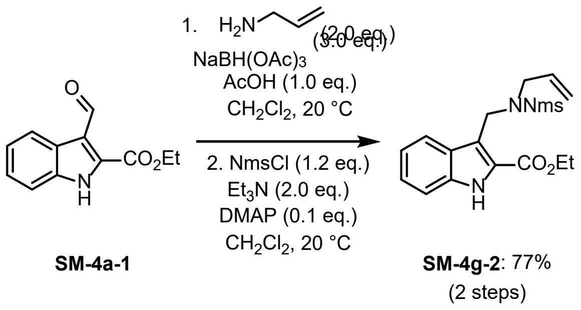

In 50 mL round-bottomed flask, **SM-4a-1** (500 mg, 1.74 mmol, 1.00 eq.) was dissolved in CH_2Cl_2 (17.4 mL) followed by the addition of allylamine (0.27 mL, 3.5 mmol, 2.0 eq.), $NaBH(OAc)_3$ (1.10 g, 5.22 mmol, 3.00 eq.) and AcOH (100 μL, 1.74 mmol, 1.00 eq.). The mixture was stirred for 20 h at 20 °C, before being poured into a sat. aq. solution of Na_2CO_3 (20 mL) and diluted with CH_2Cl_2 (20 mL). The phases were separated and the aqueous phase was extracted twice more with CH_2Cl_2 (10 mL). The organic phases were combined, dried over Na_2SO_4 and filtered. The solution was concentrated under reduced pressure to yield **SM-4g-2** as a yellow oil, which was used as such in the next step.

In a 100 mL round-bottomed flask, **SM-4g-2** (1.00 g, 3.48 mmol, 1.00 eq.) was dissolved in CH_2Cl_2 (35 mL), followed by the addition of Et_3N (0.97 mL, 7.0 mmol, 2.0 eq.) and NmsCl (1.59 g, 4.18 mmol, 1.20 eq.). The mixture was stirred for 5 h at 20 °C. The solvent was removed under reduced pressure and the crude mixture was purified by flash chromatography using a gradient of heptanes/EtOAc to yield **SM-4g-3** as a yellow solid (1.62 g, 2.69 mmol, 77% yield over 2 steps).

^{1}H NMR (600 MHz, $CDCl_3$) δ 8.82 (br s, 1H), 7.93 (s, 2H), 7.89 (d, *J* = 8.1 Hz, 1H), 7.28 – 7.25 (m, 2H), 7.10 (ddd, *J* = 8.1, 5.5, 2.4 Hz, 1H), 5.67 (ddt, *J* = 16.6, 10.3, 6.2 Hz, 1H), 5.15 (s, 2H), 5.06 (dd, *J* = 17.2, 1.2 Hz, 1H), 4.95 (dd, *J* = 10.3, 1.0 Hz, 1H), 4.44 (q, *J* = 7.1 Hz, 2H), 4.01 (d, *J* = 6.2 Hz, 2H), 1.45 (t, *J* = 7.2 Hz, 3H).

^{13}C NMR (151 MHz, $CDCl_3$) δ 161.6, 146.3, 135.3, 133.5, 133.4 (q, *J* = 34.7 Hz), 132.8 (q, *J* = 33.9 Hz), 128.6 (2C), 127.3, 126.1, 125.7, 122.2 (q, *J* = 275.7 Hz), 121.9 (q, *J* = 273.4 Hz), 121.8, 121.4, 118.2, 116.7, 111.5, 61.4, 52.7, 43.1, 14.6.

^{19}F NMR (659 MHz, $CDCl_3$) δ −54.9 (6F), −63.5 (3F).

HRMS (ESI-TOF) m/z: $[M + Na]^+$ Calcd for $C_{24}H_{19}F_9N_2O_4SNa$ 625.0814; Found 625.0812.

IR (neat) vmax: 1678, 1360, 1268, 1254, 1186, 1168, 1153, 1141, 1104, 1086, 1014, 977.

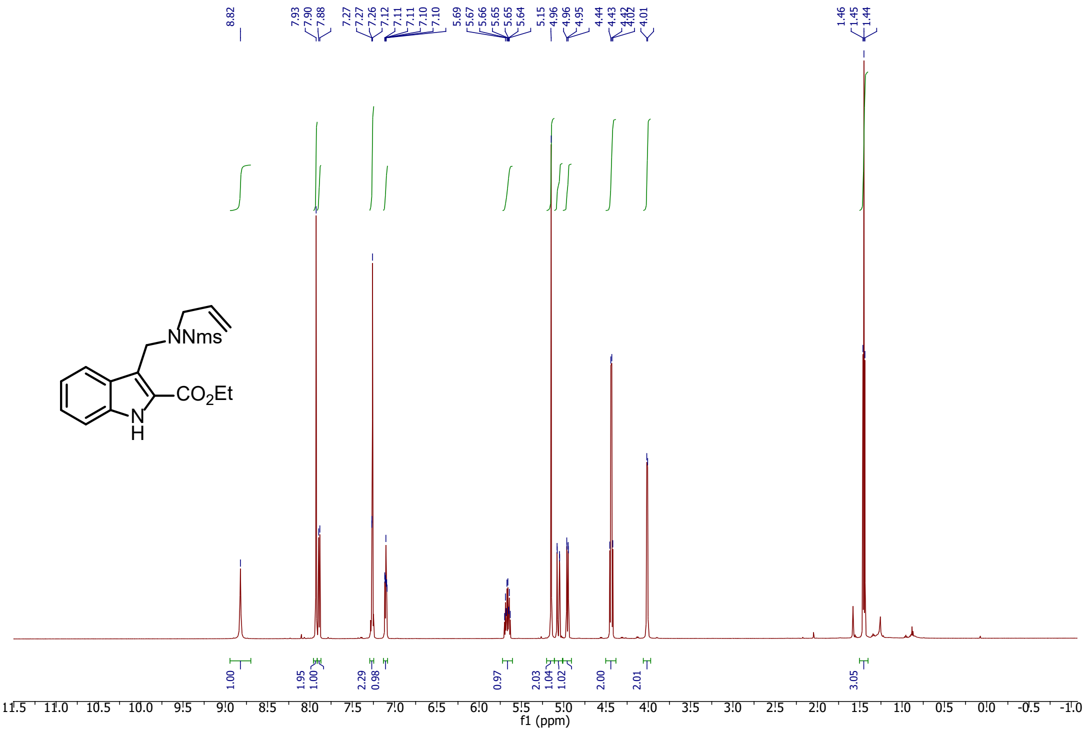

Figure 60: ¹H NMR (600 MHz, CDCl3) of SM-4g-2.

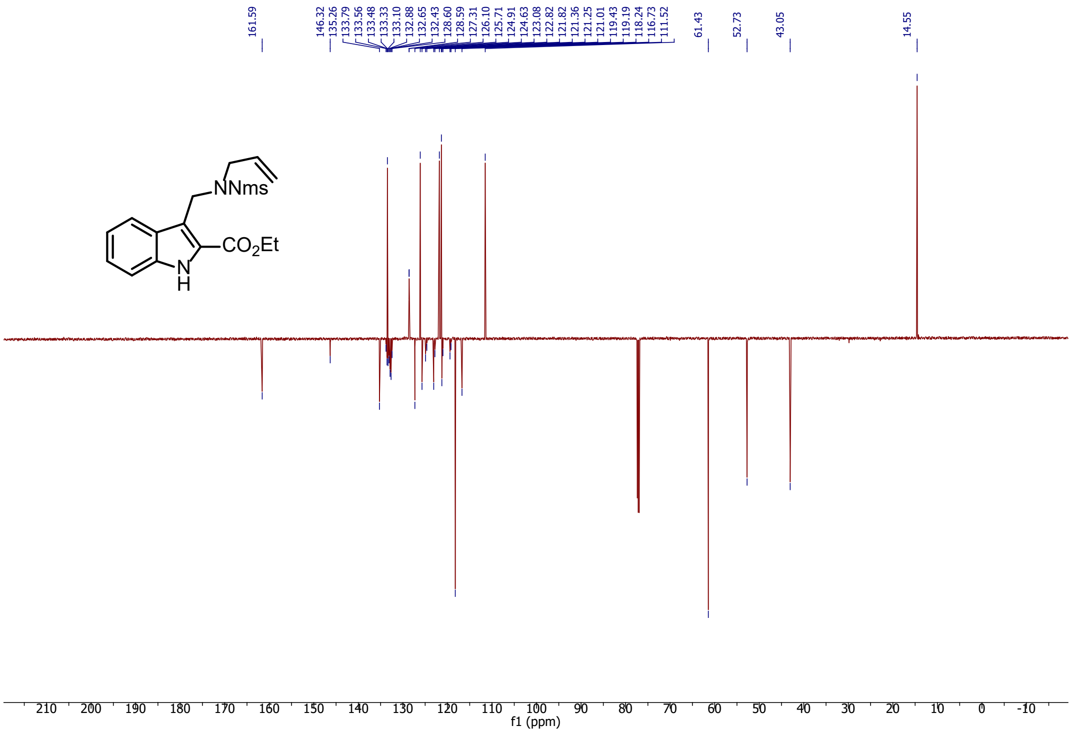

Figure 61: ***^{13}C NMR (151 MHz, CDCl₃) of SM-4g-2.***

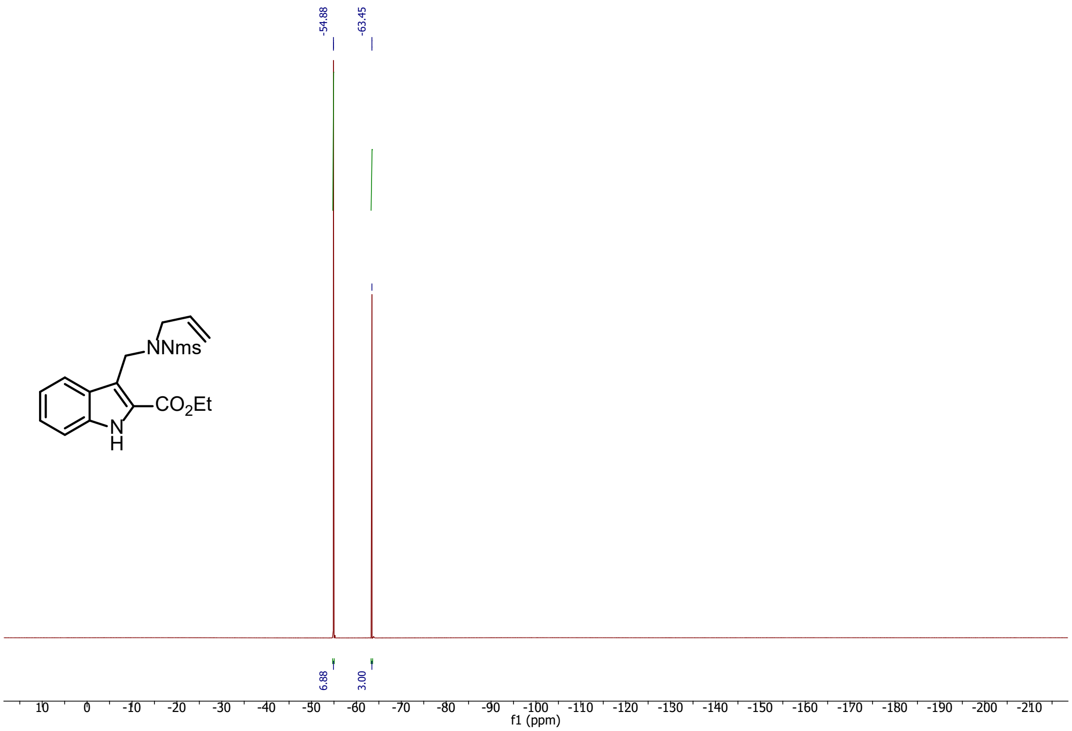

Figure 62: ***^{19}F NMR (659 MHz, CDCl₃) SM-4g-2.***

***N*-allyl-*N*-((2-formyl-1*H*-indol-3-yl)methyl)-2,4,6-tris(trifluoromethyl)benzenesulfonamide (SM-4g-4)**

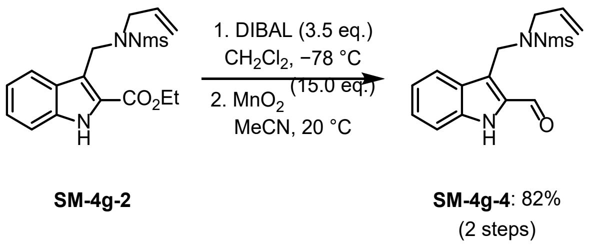

A 100 mL Schlenk flask was charged with **SM-4g-2** (1.62 g, 2.69 mmol, 1.00 eq.), CH_2Cl_2 (26.9 mL) and the mixture was cooled to −78 °C. DIBAL (1 mol/L in PhMe, 9.4 mL, 9.4 mmol, 3.5 eq.) was added dropwise before being stirred for 1 h at −78 °C. After this time, the mixture was quenched by the dropwise addition of a sat. aq. Solution of Rochelle's salt (26.9 mL) and stirred at 20 °C for 2 h. The phases were separated and the aqueous phase was extracted three times with CH_2Cl_2 (30 mL). The organic phases were combined, dried over Na_2SO_4 and filtered. The solution was concentrated under reduced pressure to yield **SM-4g-3** as a yellow oil, which was used as such in the next step.

A 100 mL round-bottomed flask was charged **SM-4g-4** (assumed pure, 1.45 g, 2.60 mmol, 1.00 eq.), acetonitrile (26.9 mL) followed by MnO_2 (3.5 g, 40 mmol, 15 eq.). The mixture was stirred at 23 °C for 17 h, before being filtered over Celite, eluted with EtOAc and concentrated under reduced pressure. The crude mixture was purified by flash chromatography using a gradient of heptanes/EtOAc to yield **SM-4g-4** as a yellow solid (1.23 g, 2.20 mmol, 82% over 2 steps).

^{1}H NMR (600 MHz, $CDCl_3$) δ 10.05 (s, 1H), 8.98 (br s, 1H), 7.98 (s, 2H), 7.73 (d, *J* = 8.2 Hz, 1H), 7.35 (t, *J* = 7.4 Hz, 1H), 7.31 (d, *J* = 8.3 Hz, 1H), 7.14 (t, *J* = 7.5 Hz, 1H), 5.82 – 5.63 (m, 1H), 5.12 (dd, *J* = 13.7, 2.6 Hz, 2H), 4.99 (s, 2H), 3.99 (d, *J* = 6.3 Hz, 2H), 1.59 (s, 1H).

^{13}C NMR (151 MHz, $CDCl_3$) δ 180.9, 145.7, 136.7, 133.9 (q, *J* = 37.1 Hz), 132.8 (q, *J* = 34.0 Hz), 132.5, 128.9, 127.8, 127.1, 122.0 (q, *J* = 275.8 Hz), 121.8 (q, *J* = 273.7 Hz), 121.7 (2C), 120.0, 118.8, 112.3, 52.4, 41.9.

^{19}F NMR (659 MHz, $CDCl_3$) δ −54.7 (6F), −63.4 (3F).

HRMS (ESI-TOF) m/z: $[M + Na]^+$ Calcd for $C_{22}H_{15}F_9N_2O_3SNa$ 581.0552; Found 581.0551.

IR (neat) vmax: 3322, 2922, 1667, 1457, 1440, 1366, 1351, 1341, 1328, 1271, 1188, 1148, 1112, 1083.

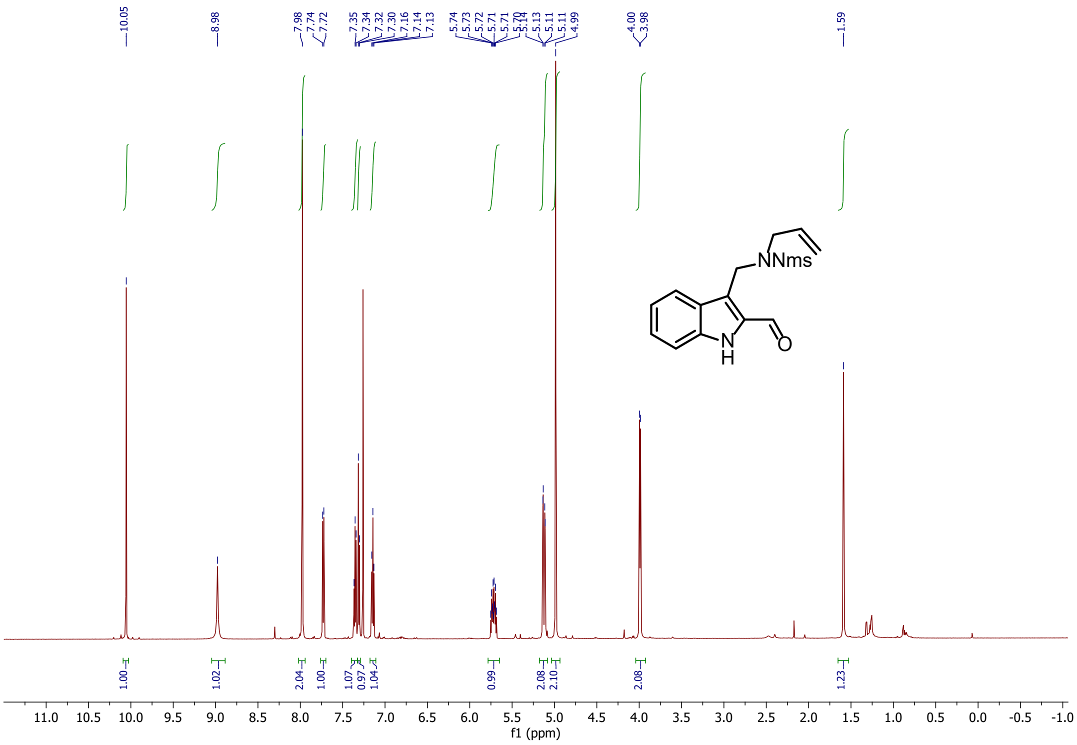

*Figure 63: **^{1}H NMR (600 MHz, CDCl3) of SM-4g-4.***

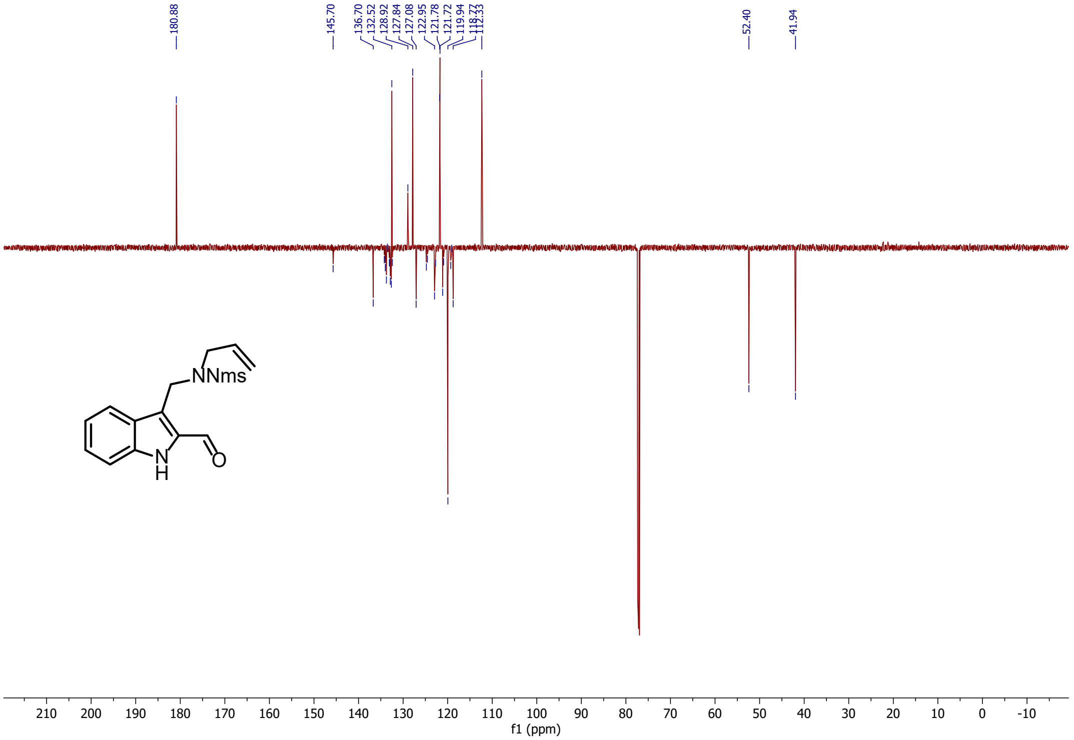

*Figure 64: **^{13}C NMR (151 MHz, CDCl3) of SM-4g-4.***

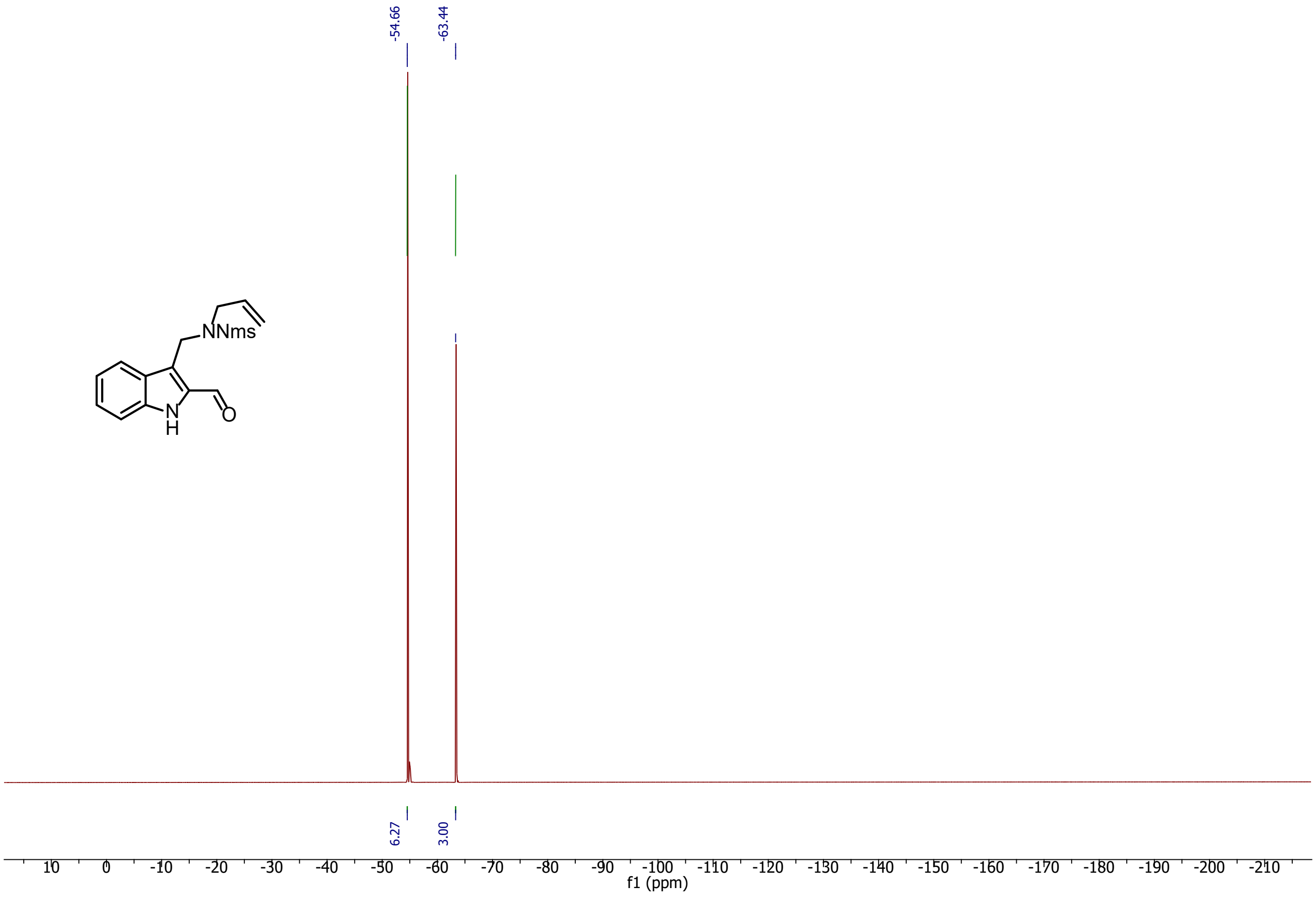

Figure 65: ***^{19}F NMR (659 MHz, $CDCl_3$) of SM-4g-4.***

Methyl (*E*)-4-((*N*-((2-formyl-1H-indol-3-yl)methyl)-2,4,6-tris(trifluoromethyl)phenyl)sulfonamido)but-2-enoate (4g)

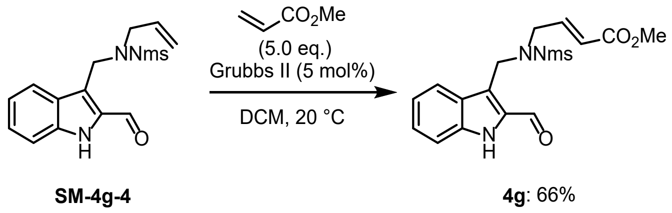

A 25 mL round-bottomed flask was charged with **SM-4g-4** (1.23 g, 2.20 mmol, 1.00 eq.) followed by CH_2Cl_2 (11.0 mL), methyl acrylate (1.0 mL, 11 mmol, 5.0 eq.) and Hoveyda-Grubbs 2nd generation catalyst (69 mg, 0.11 mmol, 5 mol%). The mixture was stirred for 20 h at 20 °C, before being concentrated under reduced pressure. The crude mixture was purified by flash chromatography using a gradient of heptanes/EtOAc to yield **4g** as a light purple solid (900 mg, 1.46 mmol, 66% yield).

^{1}H NMR (600 MHz, $CDCl_3$) δ 10.07 (s, 1H), 9.02 (br s, 1H), 8.06 (s, 2H), 7.77 (d, *J* = 8.2 Hz, 1H), 7.38 (t, *J* = 7.3 Hz, 1H), 7.33 (d, *J* = 8.3 Hz, 1H), 7.19 (t, *J* = 7.5 Hz, 1H), 6.61 (dt, *J* = 15.8, 6.1 Hz, 1H), 5.67 (d, *J* = 15.8 Hz, 1H), 5.05 (s, 2H), 4.04 (dd, *J* = 6.1, 1.1 Hz, 2H), 3.63 (s, 3H).

^{13}C NMR (151 MHz, $CDCl_3$) δ 180.6, 165.5, 145.4, 141.5, 136.7, 134.24 (q, *J* = 35.2 Hz), 133.8, 132.8 (q, *J* = 33.8 Hz), 129.2, 128.0, 127.2, 124.1, 122.1 (2C, q, *J* = 275.8 Hz), 121.8 (dd, *J* = 547.4, 273.6 Hz), 121.6, 118.3, 112.4, 51.9, 50.1, 42.6.

^{19}F NMR (659 MHz, $CDCl_3$) δ −54.9 (6F), −63.5 (3F).

HRMS (ESI-TOF) m/z: $[M + Na]^+$ Calcd for $C_{24}H_{17}F_9N_2O_5SNa$ 639.0607; Found 639.0597.

IR (neat) vmax: 3363, 2921, 1733, 1656, 1620, 1460, 1435, 1361, 1345, 1330, 1272, 1254, 1193, 1167.

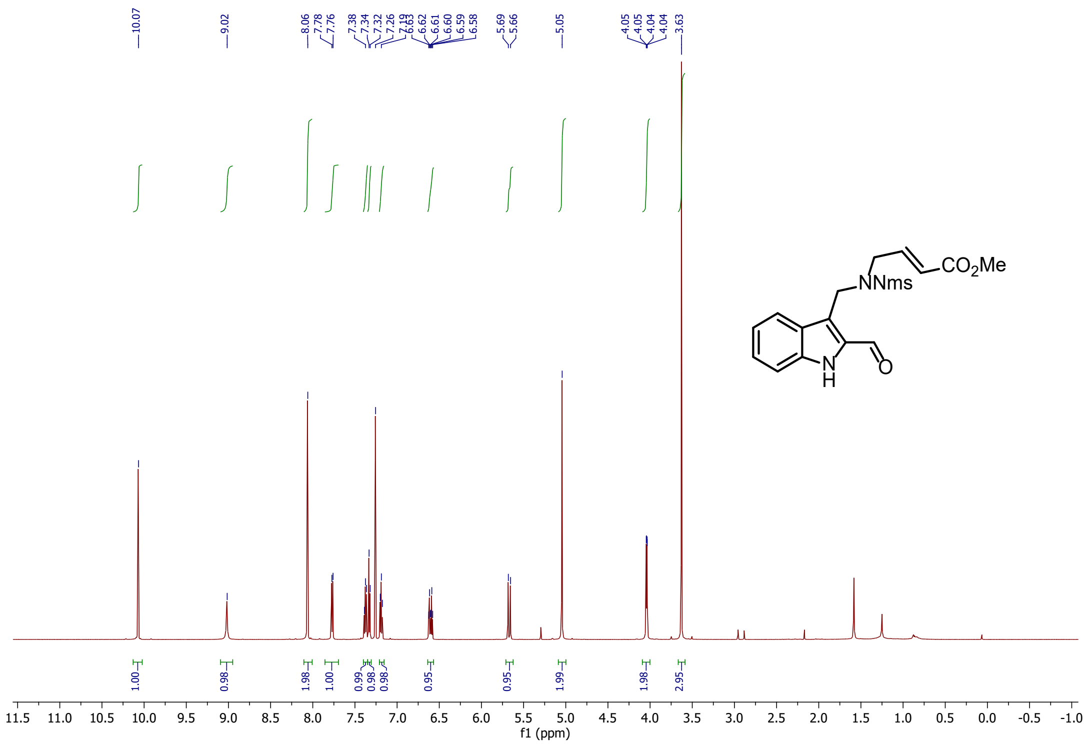

Figure 66: **^{1}H NMR (600 MHz, CDCl$_3$) of 4g.**

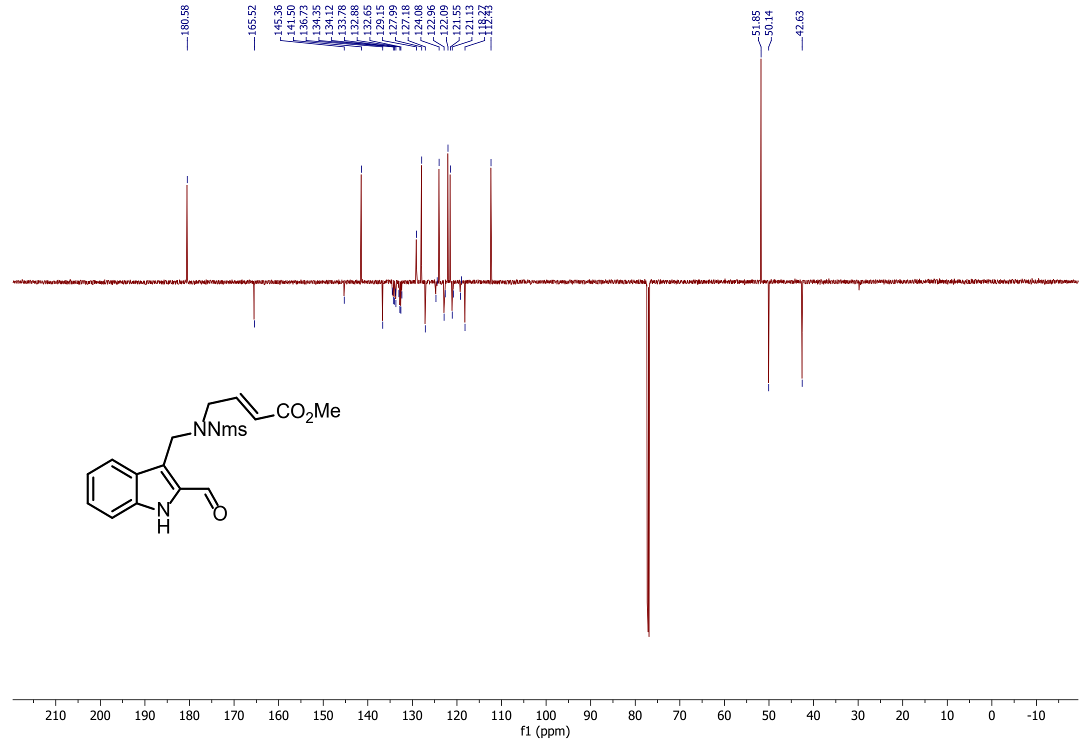

Figure 67: **^{13}C NMR (151 MHz, CDCl$_3$) of 4g.**

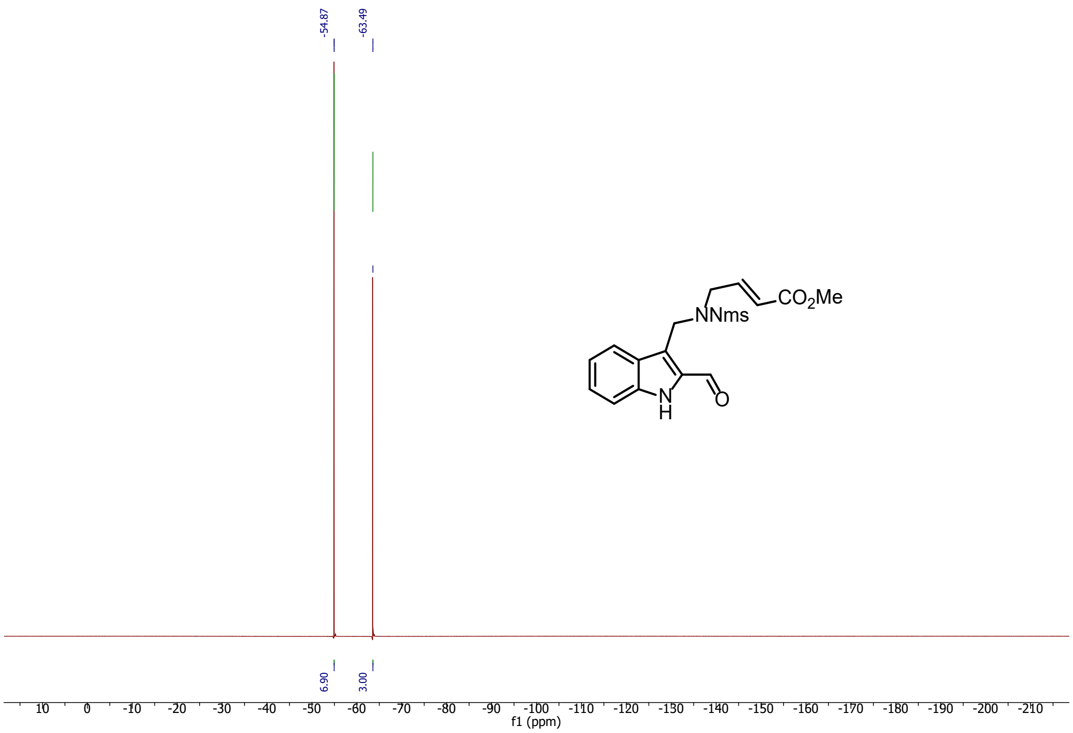

Figure 68: ***^{19}F NMR (659 MHz, $CDCl_3$) of 4g.***

k. Synthesis of methyl (*E*)-4-((tert-butoxycarbonyl)((2-formyl-1*H*-indol-3-yl)methyl)amino)but-2-enoate (4h)

Ethyl 3-((allyl(tert-butoxycarbonyl)amino)methyl)-1*H*-indole-2-carboxylate (SM-4h-2)

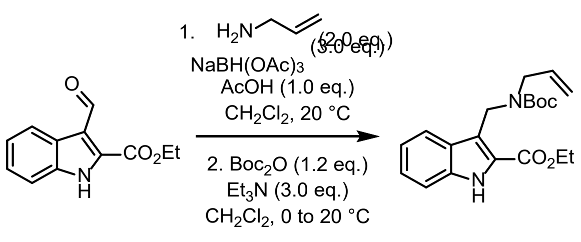

In 50 mL round-bottomed flask, **SM-4a-1** (500 mg, 1.74 mmol, 1.00 eq.) was dissolved in CH_2Cl_2 (17.4 mL) followed by the addition of allylamine (0.27 mL, 3.5 mmol, 2.0 eq.), $NaBH(OAc)_3$ (1.10 g, 5.22 mmol, 3.00 eq.) and AcOH (100 µL, 1.74 mmol, 1.00 eq.). The mixture was stirred for 20 h at 20 °C, before being poured into a sat. aq. solution of Na_2CO_3 (20 mL) and diluted with CH_2Cl_2 (20 mL). The phases were separated and the aqueous phase was extracted twice with CH_2Cl_2 (10 mL). The organic phases were combined, dried over Na_2SO_4 and filtered. The solution was concentrated under reduced pressure to yield **SM-4h-2** as a yellow oil, which was used as such in the next step.

In a 250 mL round-bottomed flask, **SM-4h-2** (500 mg, 1.74 mmol, 1.00 eq.) was dissolved in CH_2Cl_2 (17.4 mL) and cooled to 0 °C. Et_3N (0.73 mL, 5.2 mmol, 3.0 eq.) and Boc-anhydride (0.48 mL, 5.2 mmol, 1.2 eq.) were then added. The mixture was stirred for 20 h at 20 °C, before being diluted with CH_2Cl_2 (20 mL) and a sat. aq. solution of NH_4Cl (20 mL). The phases were separated and the aqueous phase was extracted twice with CH_2Cl_2 (10 mL). The organic phases were combined, dried over Na_2SO_4 and filtered. The solution was concentrated under reduced pressure and the crude mixture was purified by flash chromatography using a gradient of heptanes/EtOAc to yield **SM-4h-3** as a yellow oil (480 mg, 1.34 mmol, 77% yield over 2 steps).

^{1}H NMR (700 MHz, $CDCl_3$) δ 8.78 (br s, 1H), 8.00-7.78 (m, 1H), 7.38 (d, *J* = 8.1 Hz, 1H), 7.35 – 7.30 (m, 1H), 7.14 (t, *J* = 7.5 Hz, 1H), 5.69 (app. s, 1H), 5.09 (s, 2H), 5.01 (dd, *J* = 17.2, 1.3 Hz, 2H), 4.42 (q, *J* = 7.1 Hz, 2H), 3.81 – 3.51 (m, 2H), 1.50 (s, 9H), 1.42 (t, *J* = 7.1 Hz, 3H).

^{13}C NMR (176 MHz, $CDCl_3$) δ 162.3, 155.9, 136.0, 134.2, 128.0, 126.0, 124.9, 122.5, 120.9, 120.1, 115.5, 111.6, 79.7, 61.3, 47.9, 39.4, 28.6, 14.6 (3C).

HRMS (ESI-TOF) m/z: $[M + Na]^+$ Calcd for $C_{20}H_{26}N_2O_4Na$ 381.1785; Found 381.1772.

IR (neat) νmax: 3351, 3062, 2981, 2925, 1707, 1377, 1326, 1245, 1196, 1158, 1134, 1091, 747, 706, 664, 614, 548.

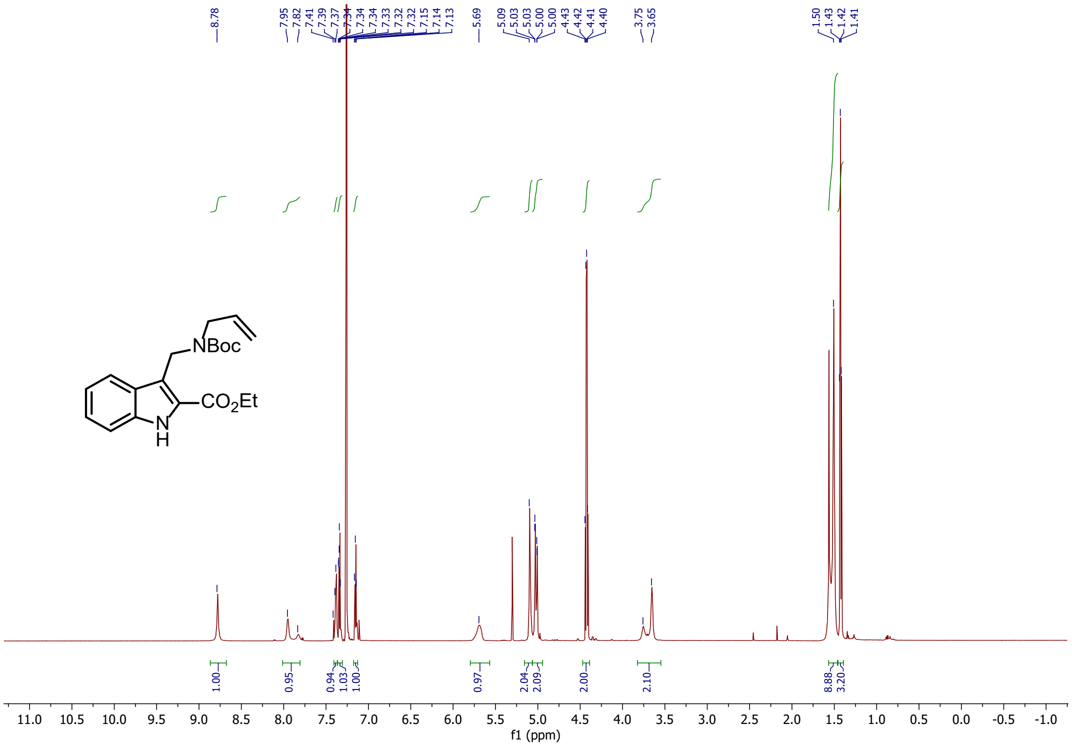

Figure 69: ***^{1}H NMR (700 MHz, CDCl$_3$) of SM-4h-2.***

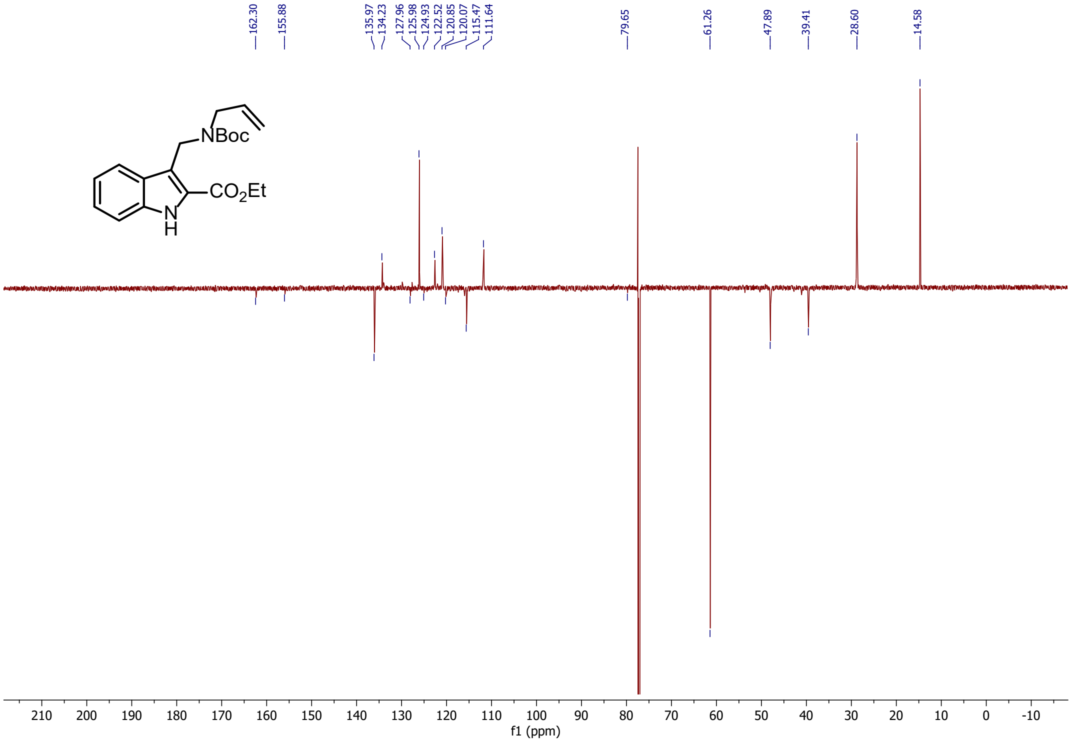

Figure 70: ***^{13}C NMR (176 MHz, CDCl$_3$) of SM-4h-2.***

Tert-butyl allyl((2-formyl-1*H*-indol-3-yl)methyl)carbamate (SM-4h-4)

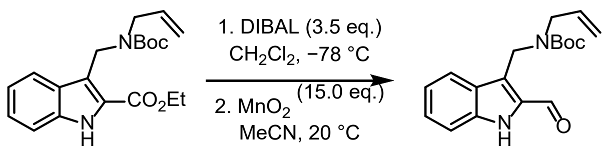

SM-4h-2 **SM-4h-4**: 57% (2 steps)

A 100 mL Schlenk flask was charged with **SM-4h-2** (348 mg, 0.970 mmol, 1.00 eq.), CH_2Cl_2 (16.6 mL) and the mixture was cooled to −78 °C. DIBAL (1 mol/L in PhMe, 3.4 mL, 3.4 mmol, 3.5 eq.) was added dropwise before being stirred for 1 h at −78 °C. After this time, the mixture was allowed to stirred at 0 °C for 10 min followed by the dropwise addition of H_2O (0.11 mL), 1 M NaOH (0.34 mL) and H_2O (0.11 mL). The mixture was allowed to warm to 20 °C and was stirred for 15 min. $MgSO_4$ was added, stirred for 15 min followed by filtration over Celite eluting with CH_2Cl_2. The filtrate was concentrated under reduced pressure to yield **SM-4h-3** as a yellow solid, which was used as such in the next step.

A 250 mL round-bottomed flask was charged with **SM-4h-3** (assumed pure, 307 mg, 0.970 mmol, 1.00 eq), acetonitrile (9.7 mL) followed by MnO_2 (1.26 g, 14.5 mmol, 15.0 eq). The mixture was stirred at 23 °C for 17 h, before being filtered over Celite, eluted with EtOAc and concentrated under reduced pressure. The crude mixture was purified by flash chromatography using a gradient of heptanes/EtOAc to yield **SM-4h-4** as a yellow solid (174 mg, 0.550 mmol, 57% over 2 steps).

^{1}H NMR (600 MHz, $CDCl_3$) δ 10.09 (br s, 1H), 8.90 (br s, 1H), 7.86 (app. s, 1H), 7.42 – 7.38 (m, 2H), 7.18 (ddd, *J* = 8.1, 4.9, 3.0 Hz, 1H), 5.73 (app. s, 1H), 5.15 (d, *J* = 10.0 Hz, 1H), 5.13 – 5.04 (m, 1H), 4.96 (s, 2H), 3.73 (app. s, 2H), 1.51 (s, 9H).

^{13}C NMR (MHz, $CDCl_3$) δ 181.3, 137.1, 133.6, 127.6, 122.1, 121.2, 116.6, 112.2, 47.8, 37.8, 28.4. *Four quaternary sp^2-carbons and one quaternary sp^3 carbon could not be found with ^{13}C-NMR. Based on the correlation in HMBC, the three of the sp^2-carbons are to expected at 123.5, 127.5 and 155.4 ppm.*

HRMS (ESI-TOF) m/z: $[M + Na]^+$ Calcd for $C_{18}H_{22}N_2NaO_3$ 337.1523; Found 337.1510.

IR (neat) νmax: 3307, 2978, 2928, 1662, 1456, 1410, 1366, 1330, 1249, 1170, 872, 748.

Mp.: 98-99 °C

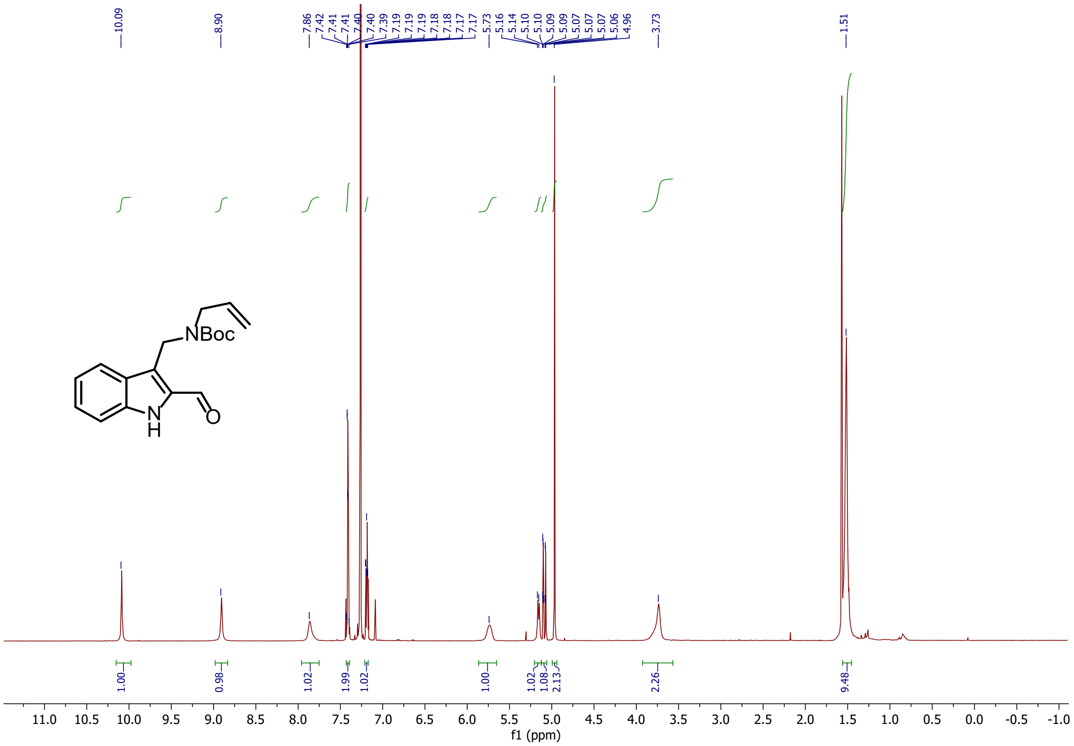

Figure 71: ***^{1}H NMR (600 MHz, CDCl₃) of SM-4h-4.***

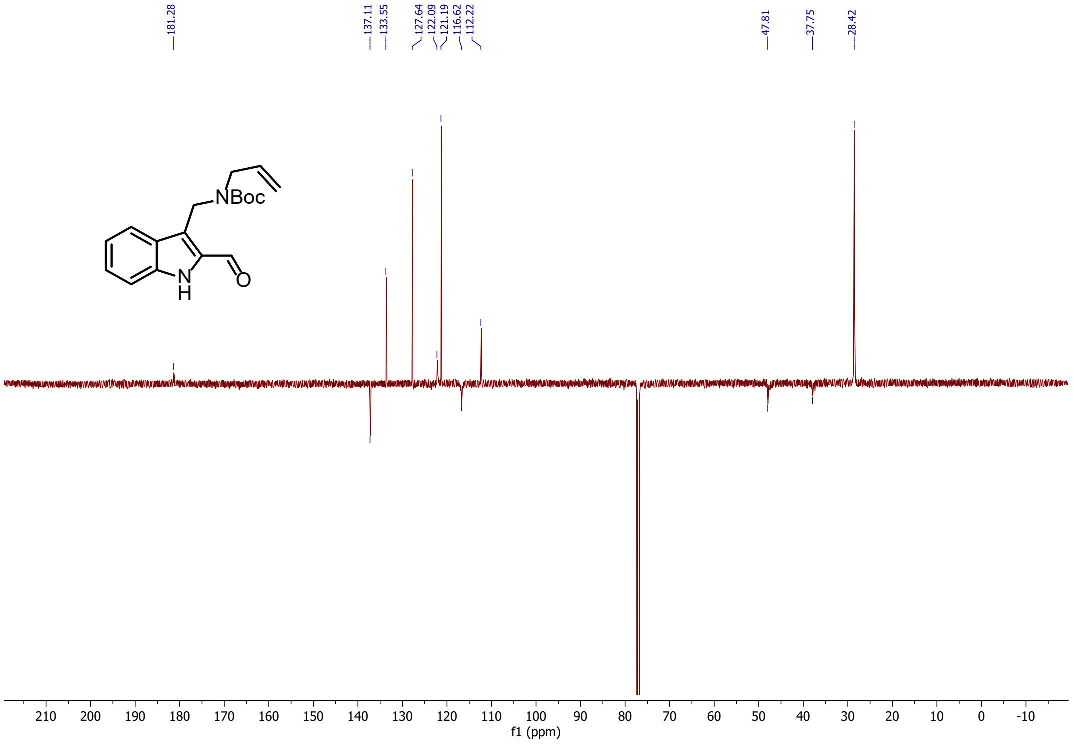

Figure 72: ***^{13}C NMR (151 MHz, CDCl₃) of SM-4h-4.***

Methyl (*E*)-4-((tert-butoxycarbonyl)((2-formyl-1*H*-indol-3-yl)methyl)amino)but-2-enoate (4h)

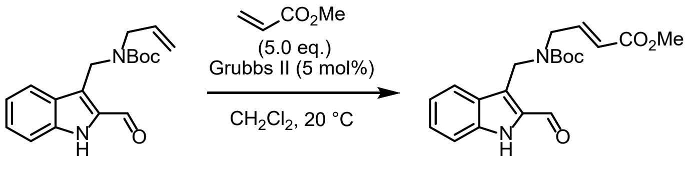

A 25 mL round-bottomed flask was charged with **SM-4h-4** (160 mg, 0.509 mmol, 1.00 eq), CH_2Cl_2 (5.1 mL), methyl acrylate (0.23 mL, 0.22 mmol, 5.0 eq.) and Hoveyda-Grubbs 2nd generation catalyst (16 mg, 0.025 mmol, 5 mol%). The mixture was stirred for 20 h at 20 °C, before being concentrated under reduced pressure. The crude mixture was purified twice by flash chromatography using a gradient of heptanes/EtOAc to yield **4h** as a light purple solid (139 mg, 0.373 mmol, 73% yield).

^{1}H NMR (400 MHz, $CDCl_3$) δ 10.08 (br s, 1H), 9.69 (br s, 1H), 7.78 (app. s, 1H), 7.45 (d, *J* = 8.3 Hz, 1H), 7.39 (app. t, *J* = 7.5 Hz, 1H), 7.17 (app. t, *J* = 7.5 Hz, 1H), 6.88 – 6.74 (m, 1H), 5.84 (d, *J* = 15.8 Hz, 1H), 4.97 (s, 2H), 3.88 (app. s, 2H), 3.73 (s, 3H), 1.49 (s, 9H).

^{13}C NMR (101 MHz, $CDCl_3$) δ 181.4, 166.4, 155.1, 143.7, 137.5, 133.4, 127.7, 127.4, 123.0, 121.6, 121.4, 80.8, 51.7, 46.3, 38.3, 28.4 (3C). *One quaternary sp^2 carbon could not be found under these conditions.*

HRMS (ESI-TOF) m/z: $[M + Na]^+$ Calcd for $C_{20}H_{24}N_2O_5Na$ 395.1577; Found 395.1563.

IR (neat) vmax: 3453, 3059, 2924, 2860, 1721, 1664, 1612, 1344, 1160, 1092, 906, 747, 664, 549.

Mp.: 103-104 °C

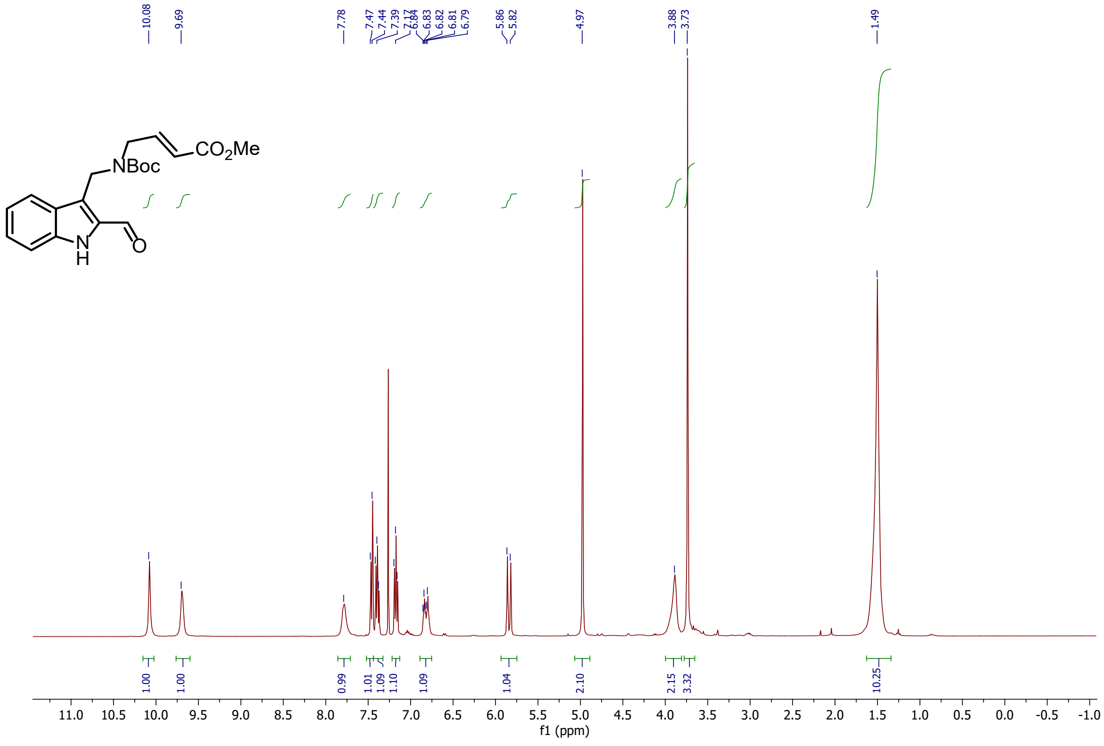

Figure 73: ***^{1}H NMR (400 MHz, CDCl3) of 4h.***

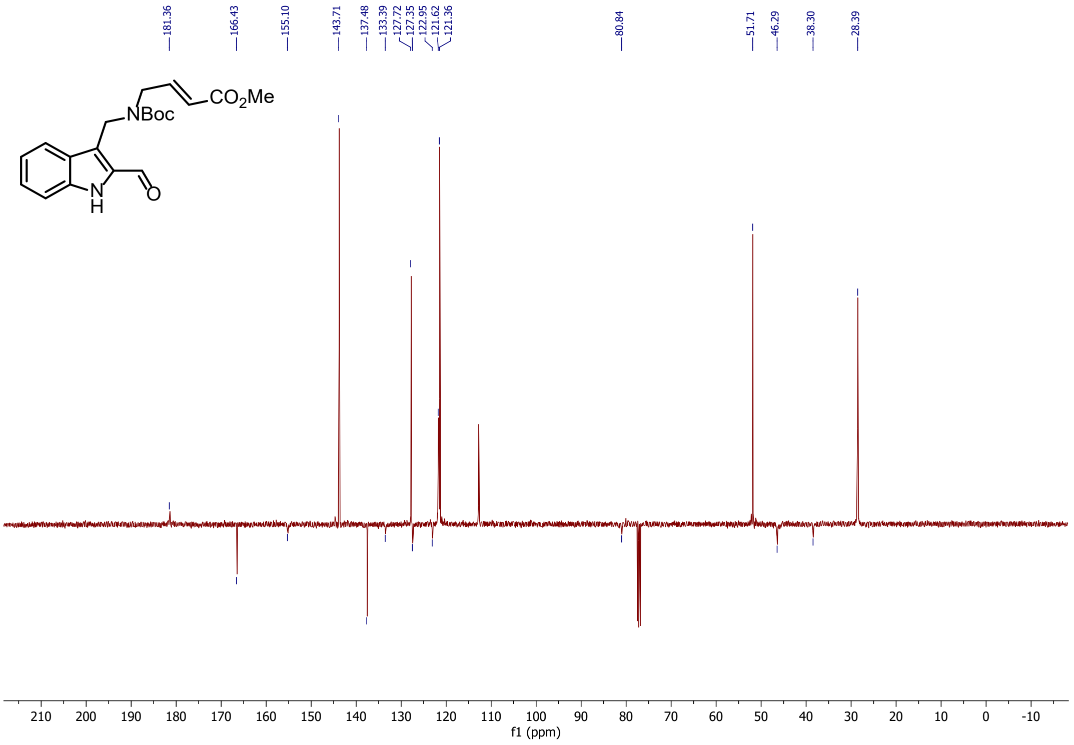

Figure 74: ***^{13}C NMR (101 MHz, CDCl3) of 4h.***

Synthesis of (*Z*)-1-(3-(((*N*-allyl-4-methylphenyl)sulfonamido)methyl)-1*H*-indol-2-yl)-N-benzylmethanimine oxide (6b)

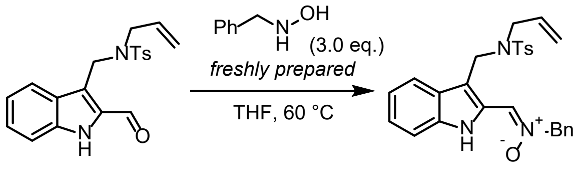

SM-4a-5 **6b**: 92%

N-benzylhydroxyamine was freshly prepared before use: A separation funnel was charged with *N*-benzylhydroxyamine hydrochloride (270 mg, 1.63 mmol), CH_2Cl_2 (30 mL) and a sat. aq. sol. of $NaHCO_3$ (25 mL). After extraction and separation of phases, the aqueous layer was extracted an additional two times with CH_2Cl_2 (10 ml). The combined organic layers were dried over $MgSO_4$, filtered and the solvent was removed under reduced pressure to afford the free hydroxylamine as a colorless solid.

A 12 mL vial was charged with **SM-4a-5** (111 mg, 0.300 mmol, 1.00 eq.), *N*-benzylhydroxyamine (111 mg, 0.900 mmol, 3.00 eq.) and THF (3 mL). The vial was sealed and the mixture was stirred for 67 h at 60 °C. Work up was performed by dilution with CH_2Cl_2 (20 mL) and an aq. sol. of HCl (1M, 20 mL). The phases were separated and the aqueous phase was extracted twice with CH_2Cl_2 (10 mL). The organic phases were combined, dried over Na_2SO_4 and filtered. The solution was concentrated under reduced pressure and the crude mixture was purified by flash chromatography using a gradient of heptanes/EtOAc to yield **6b** as a light orange solid (130 mg, 0.274 mmol, 92% yield).

^{1}H NMR (600 MHz, $CDCl_3$) δ 11.56 (br s, 1H), 8.20 (s, 1H), 7.68 – 7.61 (m, 2H), 7.44 (dd, *J* = 7.7, 1.7 Hz, 2H), 7.38 (dd, *J* = 8.1, 0.7 Hz, 1H), 7.34 – 7.29 (m, 3H), 7.26 (d, *J* = 8.3 Hz, 1H), 7.22 (d, *J* = 8.1 Hz, 2H), 7.16 – 7.10 (m, 1H), 6.98 (ddd, *J* = 8.0, 7.0, 0.9 Hz, 1H), 5.32 (ddt, *J* = 16.6, 10.2, 6.3 Hz, 1H), 4.97 (s, 2H), 4.83 (ddd, *J* = 18.4, 13.7, 1.3 Hz, 2H), 4.51 (s, 2H), 3.61 (d, *J* = 6.3 Hz, 2H), 2.35 (s, 3H).

^{13}C NMR (151 MHz, $CDCl_3$) δ 143.6, 137.2, 134.6, 133.3, 132.2, 129.9 (2C), 129.3 (2C), 129.2, 129.1 (2C), 128.4, 127.3 (2C), 127.2, 126.4, 124.6, 120.6, 119.2, 118.7, 112.2, 112.0, 70.2, 49.4, 40.4, 21.6.

HRMS (ESI-TOF) m/z: $[M + Na]^+$ Calcd for $C_{27}H_{27}N_3O_3SNa$ 496.1665; Found 496.1668.

IR (neat) vmax: 3331, 3063, 3033, 2982, 2922, 2249, 1597, 1445, 1153, 905, 723, 699, 659, 546.

Mp.: 139-140 °C

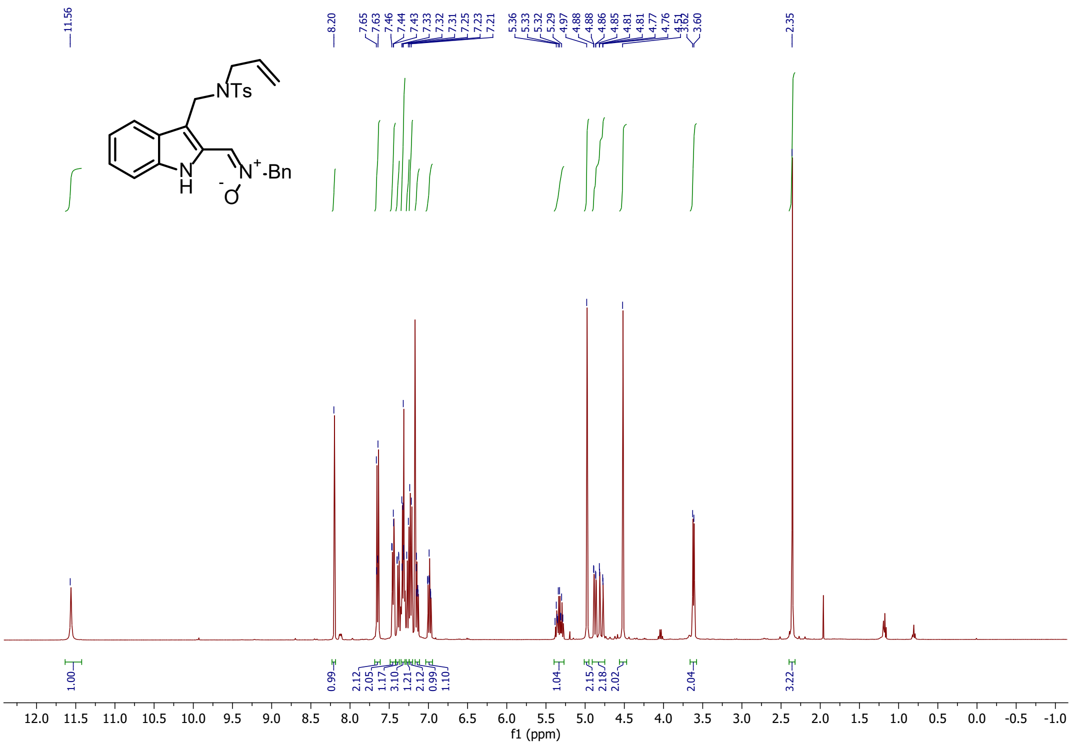

*Figure 75: ^{1}**H NMR (600 MHz, CDCl$_3$) of 6b.***

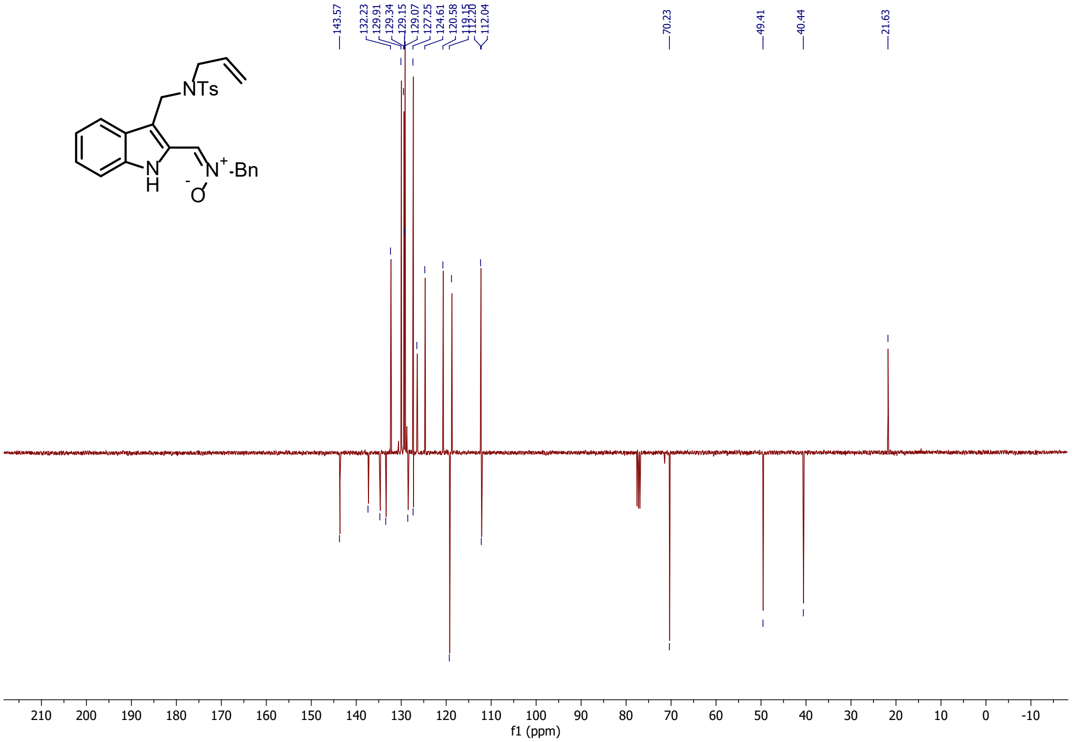

*Figure 76: ^{13}**C NMR (151 MHz, CDCl$_3$) of 6b.***

Synthesis of methyl (*E*)-4-((tert-butoxycarbonyl)((2-formyl-1*H*-indol-3-yl)methyl)amino)but-2-enoate (6c)

(3-Bromoprop-1-en-2-yl)benzene (SM-6c-1)

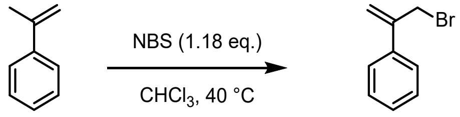

SM-6c-1

SM-6c-1 was synthesized according to a reported procedure.[7]

^{1}H NMR (400 MHz, CDCl$_3$) δ 7.53 – 7.46 (m, 2H), 7.43 – 7.30 (m, 3H), 5.56 (s, 1H), 5.50 (d, *J* = 0.6 Hz, 1H), 4.39 (d, *J* = 0.4 Hz, 2H).

The analytical data is in accordance to that previously reported.[7]

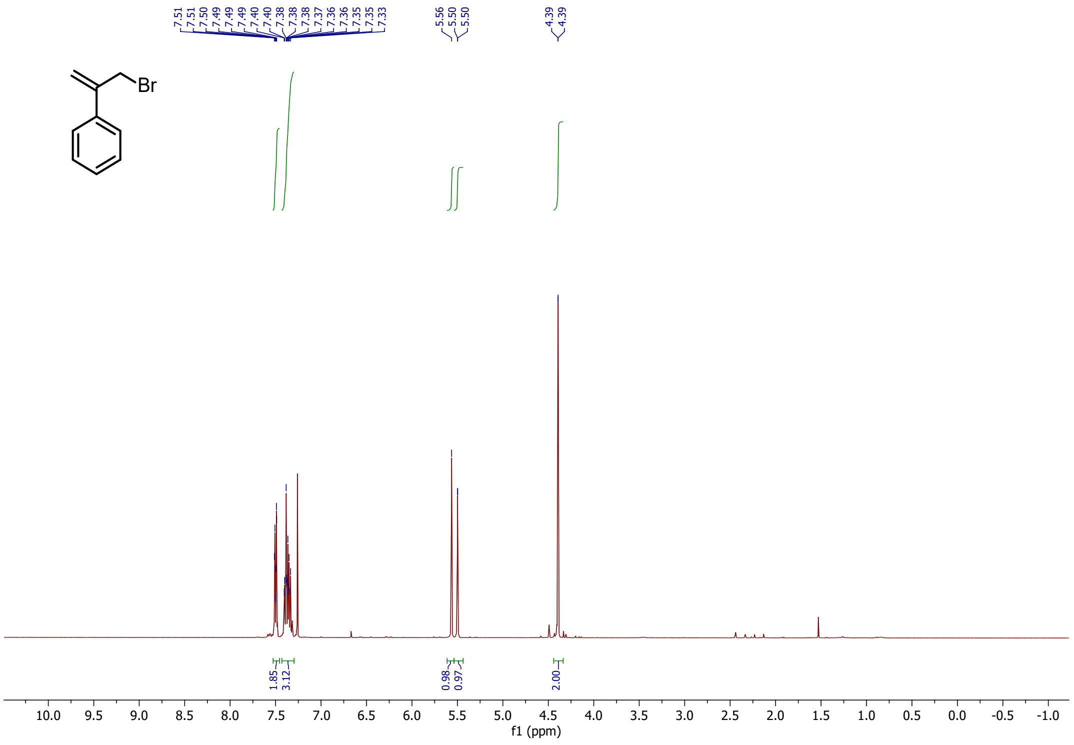

Figure 77: ^{1}H NMR (400 MHz, CDCl$_3$) of SM-6c-1.

2-Phenylprop-2-en-1-amine (SM-6c-2)

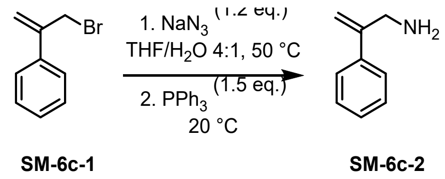

SM-6c-2 was synthesized according to a reported procedure and was directly used in the next step without further purification.[8]

Ethyl 3-(((4-methyl-*N*-(2-phenylallyl)phenyl)sulfonamido)methyl)-1H-indole-2-carboxylate (SM-6c-4)

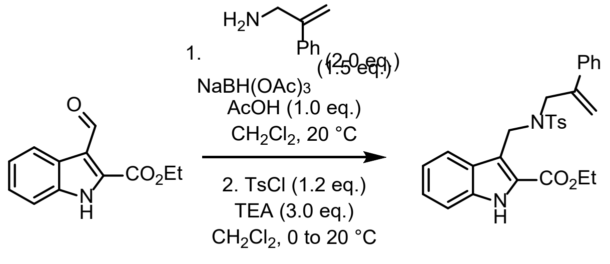

In a 50 mL round-bottomed flask, **SM-4a-1** (2.17 g, 4.82 mmol, 1.00 eq.) was dissolved in CH_2Cl_2 (24 mL) followed by the addition of **SM-6c-2** (assumed pure, 1.31 g, 9.64 mmol, 2.00 eq.), $Na(OAc)_3BH$ (1.53 g, 7.23 mmol, 1.50 eq.) and AcOH (0.28 mL, 4.8 mmol, 1.00 eq.). The mixture was stirred for 20 h at 20 °C, before being poured into a sat. aq. solution of Na_2CO_3 (25 mL) and diluted with CH_2Cl_2 (25 mL). The phases were separated and the aqueous phase was extracted twice with CH_2Cl_2 (15 mL). The organic phases were combined, dried over Na_2SO_4 and filtered. The solution was concentrated under reduced pressure to yield **SM-6c-3** as a yellow oil, which was used as such in the next step.

In a 250 mL round-bottomed flask, **SM-6c-3** (assumed pure, 1.49 g, 4.48 mmol, 1.00 equiv.) was dissolved in CH_2Cl_2 (23 mL). The solution was cooled to 0 °C, followed by the addition of Et_3N (1.87 mL, 13.4 mmol, 3.00 eq.) and *p*-TsCl (1.02 g, 5.38 mmol, 1.20 eq.). The mixture was stirred for 1 h at 20 °C, before being diluted with CH_2Cl_2 (25 mL) and a sat. aq. solution of NH_4Cl (25 mL). The phases were separated and the aqueous phase was extracted twice with CH_2Cl_2 (15 mL). The organic phases were combined, dried over Na_2SO_4 and filtered. The solution was concentrated under reduced pressure and the crude mixture was purified by flash chromatography using a gradient of heptanes/EtOAc to yield **SM-6c-4** as a yellow solid (1.39 g, 2.84 mmol, 64% yield over 2 steps).

^{1}H NMR (600 MHz, CDCl$_3$) δ 8.67 (br.s, 1H), 7.88 (dd, *J* = 8.1, 0.6 Hz, 1H), 7.76 (d, *J* = 8.2 Hz, 2H), 7.74 – 7.69 (m, 1H), 7.33 (d, *J* = 8.0 Hz, 2H), 7.31 – 7.27 (m, 4H), 7.25 – 7.22 (m, 1H), 7.10 – 7.01 (m, 4H), 6.95 – 6.87 (m, 2H), 4.93 (s, 2H), 4.91 (dd, *J* = 13.9, 0.9 Hz, 2H), 4.23 (q, *J* = 7.1 Hz, 2H), 4.16 (s, 2H), 2.46 (s, 3H), 1.27 (t, *J* = 7.1 Hz, 3H).

^{13}C NMR (MHz, CDCl$_3$) δ 161.7, 143.5, 143.4, 139.0, 135.9, 135.6, 129.9, 129.8, 128.7 (2C), 128.2, 127.9 (2C), 127.6 (2C), 127.4, 126.2, 126.0, 124.8, 122.2, 121.1, 114.1, 111.4, 61.1, 52.6, 44.0, 21.7, 21.7, 14.4.

HRMS (ESI-TOF) m/z: $[M + Na]^+$ Calcd for $C_{28}H_{28}N_2O_4SNa$ 511.1662; Found 511.1665.

IR (neat) νmax: 3341, 3059, 2981, 2934, 1705, 1396, 1326, 1244, 1158, 1091, 548.

Mp.: 133-134 °C.

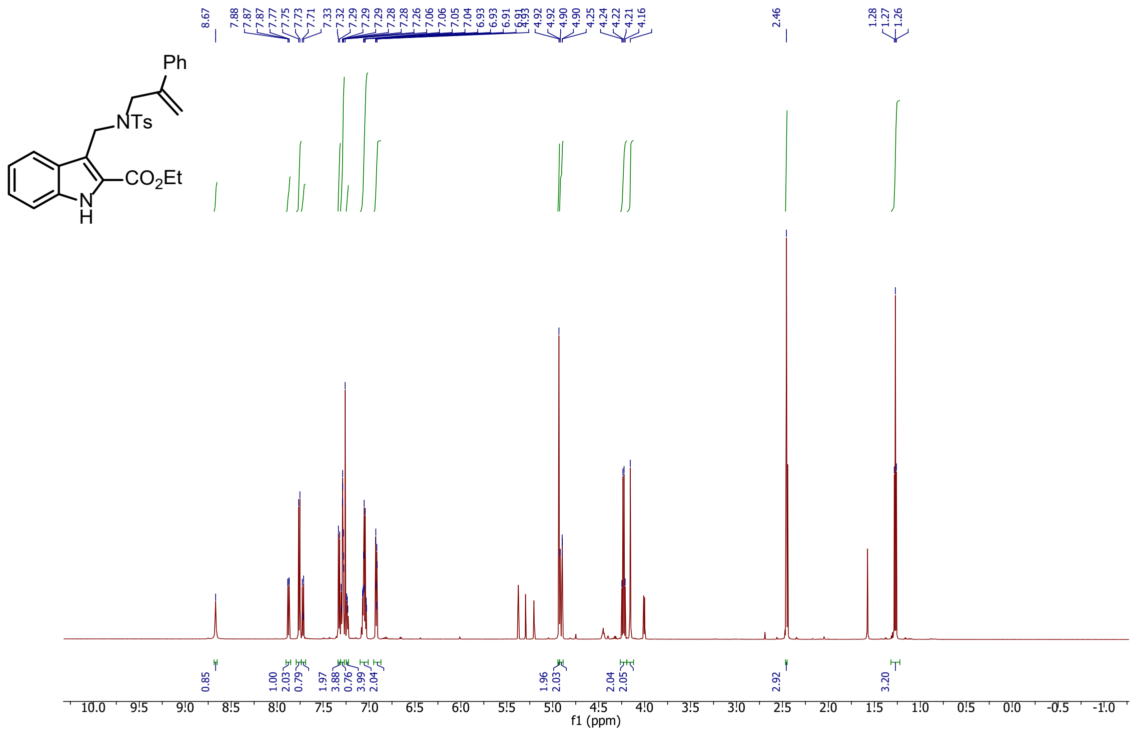

Figure 78: ***^{1}H NMR (700 MHz, $CDCl_3$) of SM-6c-4.***

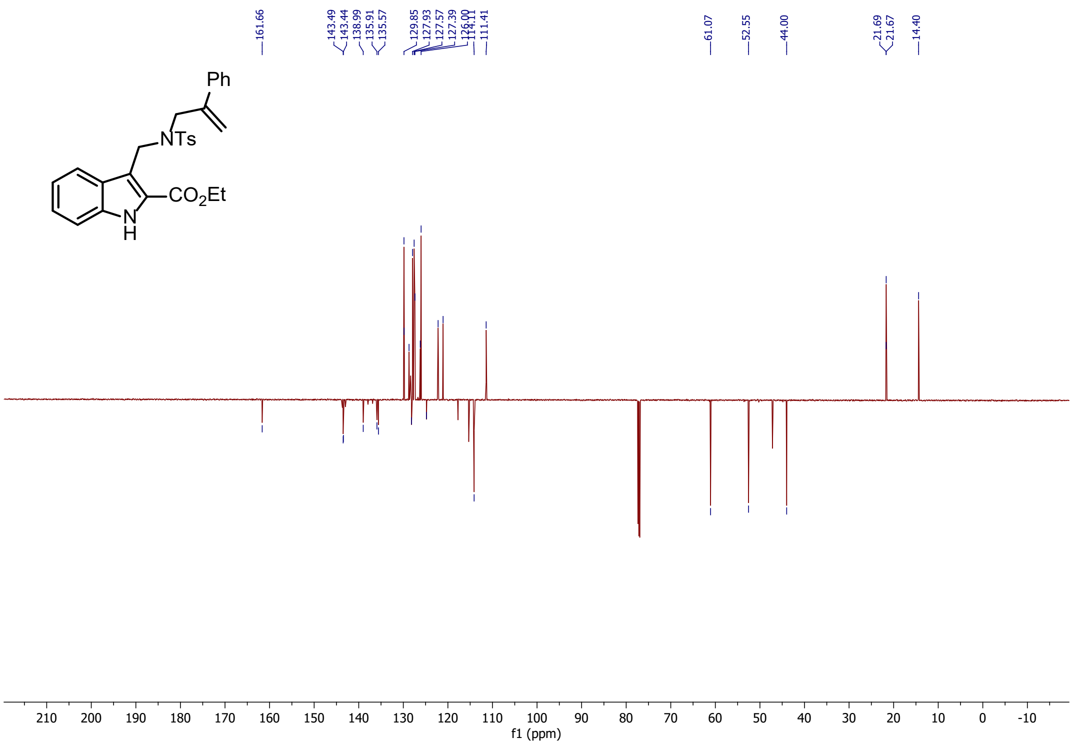

Figure 79: ^{13}C NMR (176 MHz, CDCl$_3$) of SM-6c-4.

***N*-((2-formyl-1H-indol-3-yl)methyl)-4-methyl-*N*-(2-phenylallyl)benzenesulfonamide (SM-6c-6)**

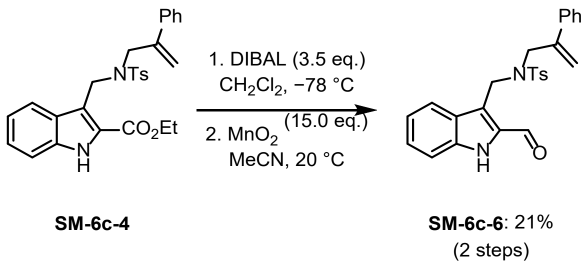

A 100 mL Schlenk flask was charged with **SM-6c-4** (1.37 g, 2.52 mmol, 1.00 eq.), CH_2Cl_2 (40 mL) and the mixture was cooled to −78 °C. DIBAL (1 mol/L in PhMe, 12.6 mL, 8.83 mmol, 3.50 eq.) was added dropwise before being stirred for 1 h at −78 °C. After this time, the mixture was allowed to stirred at 0 °C for 10 min followed by the dropwise addition of H_2O (0.88 mL), 1 M NaOH (0.88 mL) and H_2O (2.6 mL). The mixture was allowed to return to ambient temperature and was stirred for 15 min. $MgSO_4$ was added, stirred for 15 min followed by filtration over Celite eluting with CH_2Cl_2. The filtrate was concentrated under reduced pressure to yield **SM-6c-5** as a yellow solid, which was used as such in the next step.

A 250 mL round-bottomed flask was charged with **SM-6c-5** (assumed pure, 1.13 g, 2.52 mmol, 1.00 eq), acetonitrile (25 mL) followed by MnO_2 (3.29 g, 37.8 mmol, 15.0 eq). The mixture was stirred at 20 °C for 15 h, before being

filtered over Celite, eluted with EtOAc and concentrated under reduced pressure. The crude mixture was purified by flash chromatography using a gradient of heptanes/EtOAc to yield **SM-6c-6** as a yellow solid (230 mg, 0.520 mmol, 21% over 2 steps).

^{1}H NMR (600 MHz, $CDCl_3$) δ 9.86 (s, 1H), 8.71 (s, 1H), 7.69 (d, *J* = 8.3 Hz, 2H), 7.59 (dd, *J* = 8.2, 0.7 Hz, 1H), 7.33 – 7.28 (m, 4H), 7.18 – 7.08 (m, 3H), 7.04 – 6.91 (m, 3H), 5.14 (s, 1H), 5.02 (s, 1H), 4.77 (s, 2H), 4.25 (s, 2H), 2.44 (s, 3H).

^{13}C NMR (MHz, $CDCl_3$) δ 180.9, 143.8, 143.0, 138.1, 136.7, 136.0, 133.2, 129.9 (2C), 128.3 (2C), 128.1, 127.5 (2C), 127.5, 127.4, 126.1 (2C), 122.0, 121.3, 120.1, 115.9, 112.1, 52.8, 42.0, 21.7.

HRMS (ESI-TOF) m/z: $[M + Na]^+$ Calcd for $C_{26}H_{24}N_2O_3SNa$ 467.1400; Found 467.1396.

IR (neat) νmax: 3315, 3060, 2921, 2858, 1711, 1653, 1330, 1159, 911, 878, 709.

Mp.: 173-174 °C.

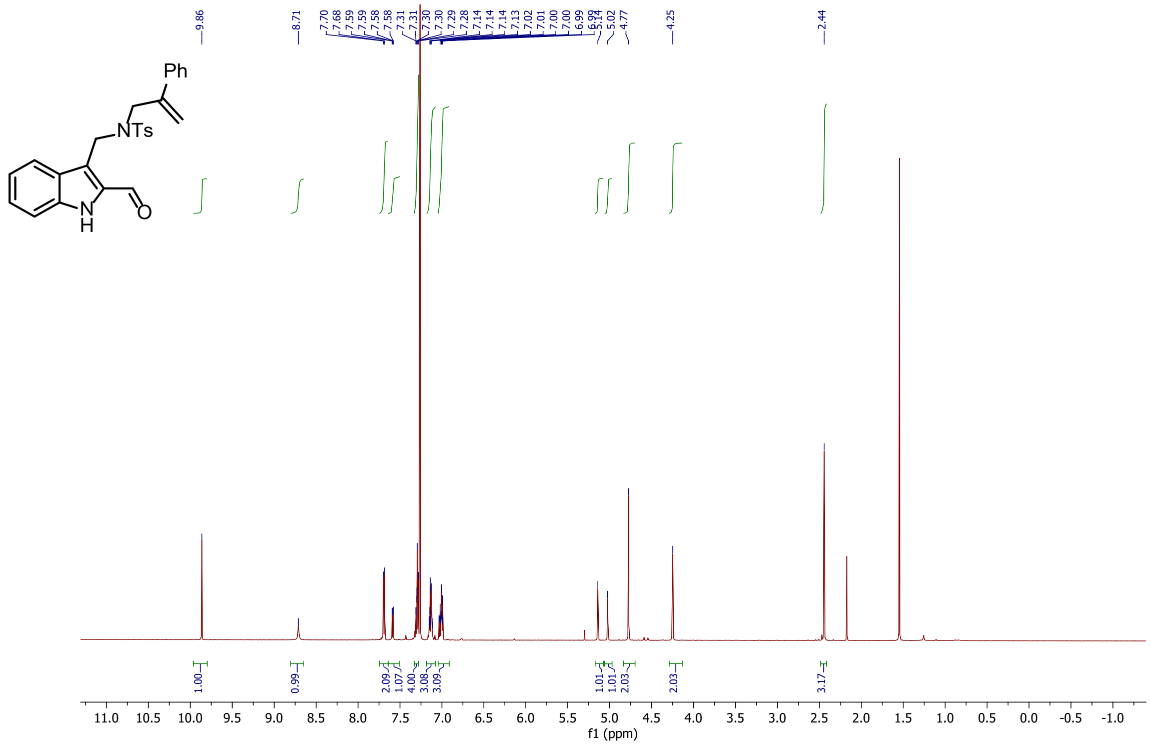

Figure 80: ***^{1}H NMR (600 MHz, $CDCl_3$) of SM-6c-6.***

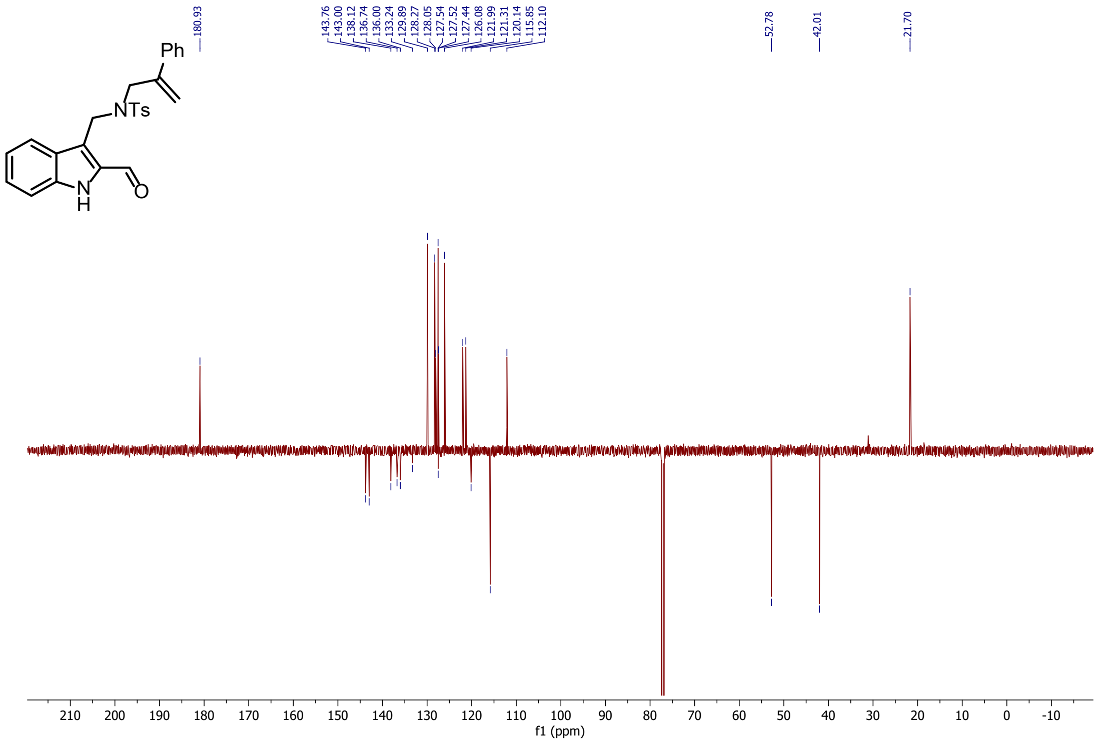

Figure 81: ***^{13}C NMR (151 MHz, $CDCl_3$) of SM-6c-6.***

(*Z*)-*N*-benzyl-1-(3-(((4-methyl-*N*-(2-phenylallyl)phenyl)sulfonamido)methyl)-1H-indol-2-yl)methanimine oxide (6c)

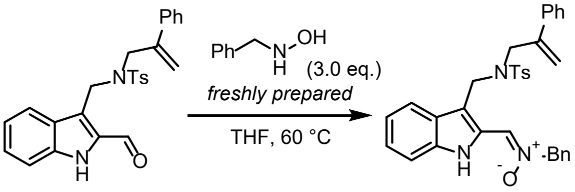

SM-6c-6 **6c**: 89%

N-benzylhydroxyamine was freshly prepared before use: A separation funnel was charged with *N*-benzylhydroxyamine hydrochloride (270 mg, 1.63 mmol), CH_2Cl_2 (30 mL) and a sat. aq. sol. of $NaHCO_3$ (25 mL). After extraction and separation of phases, the aqueous layer was extracted twice with CH_2Cl_2 (10 ml). The combined organic layers were dried over $MgSO_4$, filtered and the solvent was removed under reduced pressure to afford the free hydroxylamine as a colorless solid.

A 12 mL vial was charged with **SM-6c-6** (133 mg, 0.300 mmol, 1.00 eq.), *N*-benzylhydroxyamine (111 mg, 0.900 mmol, 3.00 eq.) and THF (3 mL). The vial was sealed and the mixture was stirred for 67 h at 60 °C. Work up was performed by dilution with CH_2Cl_2 (20 mL) and an aq. sol. of HCl (1M, 20 mL). The phases were separated and the aqueous phase was extracted twice with CH_2Cl_2 (10 mL). The organic phases were combined, dried over Na_2SO_4 and filtered. The solution was concentrated under reduced pressure and the crude mixture was purified by flash chromatography using a gradient of heptanes/EtOAc to yield **6c** as a light orange solid (146 mg, 0.266 mmol, 89% yield).

^{1}H NMR (700 MHz, $CDCl_3$) δ 11.47 (s, 1H), 7.99 (s, 1H), 7.71 – 7.63 (m, 2H), 7.49 – 7.36 (m, 5H), 7.31 – 7.26 (m, 4H), 7.22 – 7.14 (m, 2H), 7.14 – 7.08 (m, 2H), 6.99 (ddd, *J* = 8.0, 6.9, 0.9 Hz, 1H), 6.90 (dt, *J* = 8.4, 1.8 Hz, 2H), 4.96 (s, 1H), 4.93 – 4.90 (m, 1H), 4.85 (s, 2H), 4.51 (s, 2H), 4.17 (s, 2H), 2.44 (s, 3H)..

^{13}C NMR (171 MHz, $CDCl_3$) δ 143.7, 142.7, 138.2, 135.9, 134.4, 133.2, 129.9 (2C), 129.5 (2C), 129.1, 129.1 (2C), 128.7, 128.1 (2C), 127.8, 127.4 (2C), 127.1, 126.4, 126.2 (2C), 124.4, 120.4, 118.6, 115.3, 112.1, 111.7, 70.0, 52.8, 42.4, 21.7..

HRMS (ESI-TOF) m/z: $[M + H]^+$ Calcd for $C_{33}H_{32}N_3O_3S$ 550.2159; Found 550.2153.

IR (neat) vmax: 3331, 3084, 3055, 3032, 2983, 2922, 2860, 1596, 1517, 1495, 1416, 1328, 1153, 1118, 1091, 911, 736, 698, 657, 547.

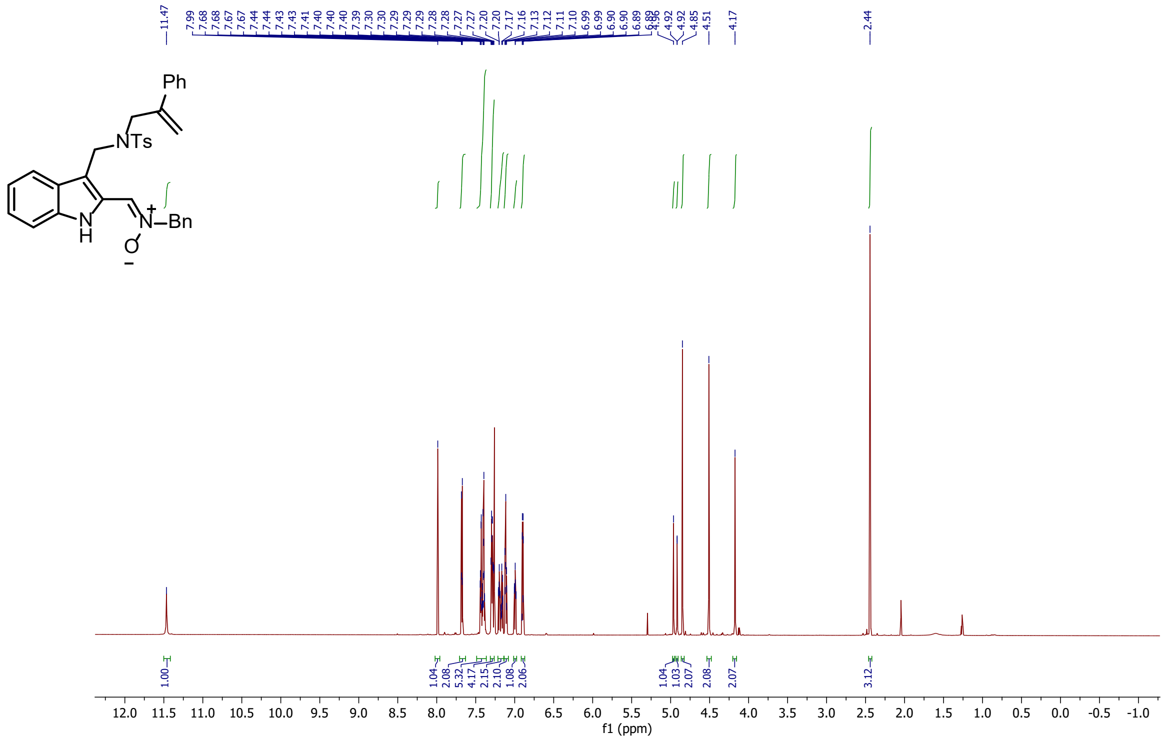

Figure 82: ^{1}H NMR (600 MHz, CDCl3) of 6c.

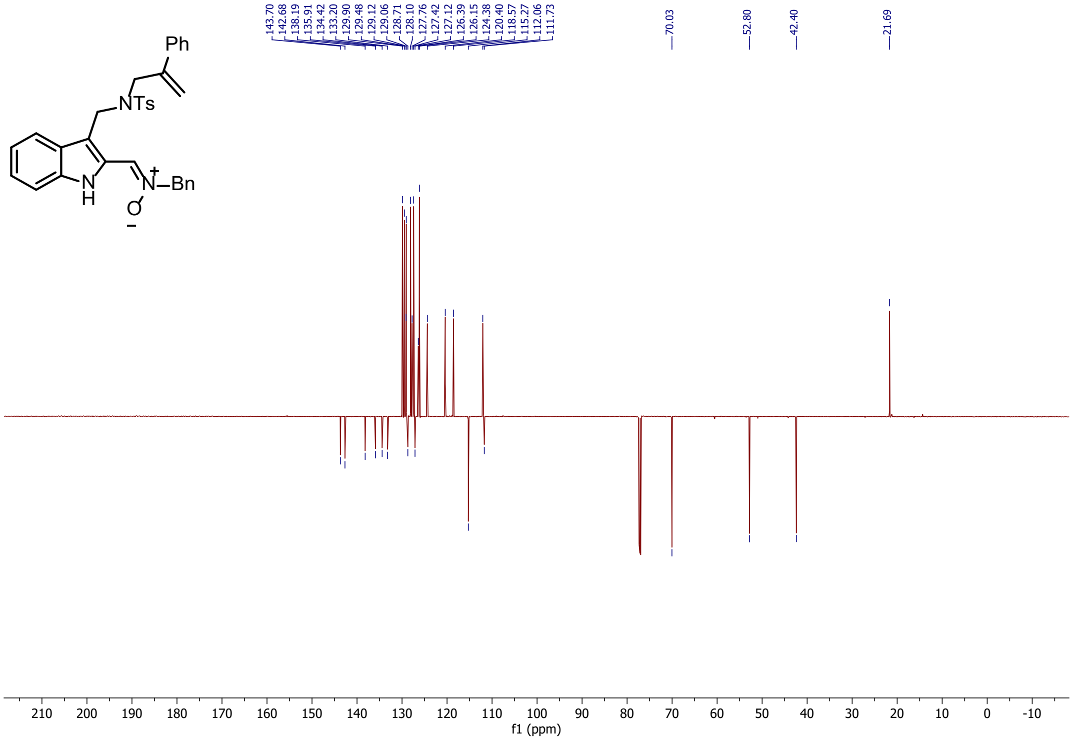

Figure 83: 13C NMR (171 MHz, CDCl3) of 6c.

V. Synthesis and characterization of products

General procedure:

A flame-dried vial was charged with indole-2-carbaldehyde derivative (0.10 mmol, 1.0 eq.) and amino acid (0.30 mmol, 3.0 eq.). The vial was capped and placed under Argon. DMF (1 mL, 0.1 M) was added and the mixture was heated at 100 °C for 18 h, before being allowed to return to ambient temperature. A sat. aq. sol. of brine (2 mL x 0.1 mmol) and EtOAc (2 mL x 0.1 mmol) were added, and the organic phase was then separated. The aqueous phase was extracted three times with EtOAc (5 mL x 0.1 mmol). The combined organic layers were washed four times with a sat. aq. sol. of brine (5 mL x 0.1 mmol), dried over $MgSO_4$, filtered, and concentrated under reduced pressure. The crude mixture was purified by normal-phase flash column chromatography. *Unless stated otherwise diastereomeric ratio (dr) was measured by crude ^1H NMR analysis.*

Methyl (3*S**,3a*S**,11b*R**)-1,11-dimethyl-2,3,3a,4,5,6,11,11b-octahydro-1*H*-pyrrolo[3',2':6,7]cyclohepta[1,2-b]indole-3-carboxylate (3)

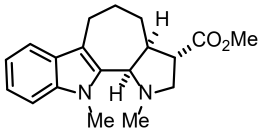

3

Synthesized with deviations from the general procedure using **1** (29 mg, 0.10 mmol, 1.0 eq.) and sarcosine **2a** (27 mg, 0.30 mmol, 3.0 eq.) by heating the reaction mixture at 120 °C and using dried 4 Å molecular sieves. Purification by flash column chromatography (gradient of heptanes/EtOAc) gave **3** as a yellow oil (6.7 mg, 0.021 mmol, 21% yield).

^{1}H NMR (600 MHz, $CDCl_3$) δ 7.54 (d, *J* = 7.8 Hz, 1H), 7.28 (d, *J* = 8.2 Hz, 1H), 7.23 – 7.18 (m, 1H), 7.14 – 7.01 (m, 1H), 3.74 (s, 3H), 3.74 (s, 3H), 3.70-3.63 (m, 1H), 3.40 (t, *J* = 8.3 Hz, 1H), 3.07 – 2.88 (m, 3H), 2.72 (app. td, *J* = 10.0, 5.5 Hz, 1H), 2.49 (t, *J* = 9.1 Hz, 1H), 2.23 – 2.11 (m, 4H), 1.91 – 1.80 (m, 1H), 1.74 – 1.65 (m, 2H).

^{13}C NMR (151 MHz, $CDCl_3$) δ 175.6, 137.0, 128.1, 121.5, 118.9, 118.6, 109.2, 63.8, 58.2, 52.1, 47.6, 44.7, 40.4, 31.3, 30.1, 29.9, 23.5, 21.2. ppm. *Two quaternary sp^2 carbons could not be found under these conditions. Based on correlation in HMBC, one sp^2 quaternary carbon is expected to have a shift of 114.1 ppm.*

HRMS (ESI-TOF) m/z: $[M + H]^+$ Calcd for $C_{19}H_{25}N_2O_2$ 313.1911; Found 313.1909.

IR (neat) νmax: 2923, 2853, 2772, 1733, 1469, 1435, 1195, 1168, 740.

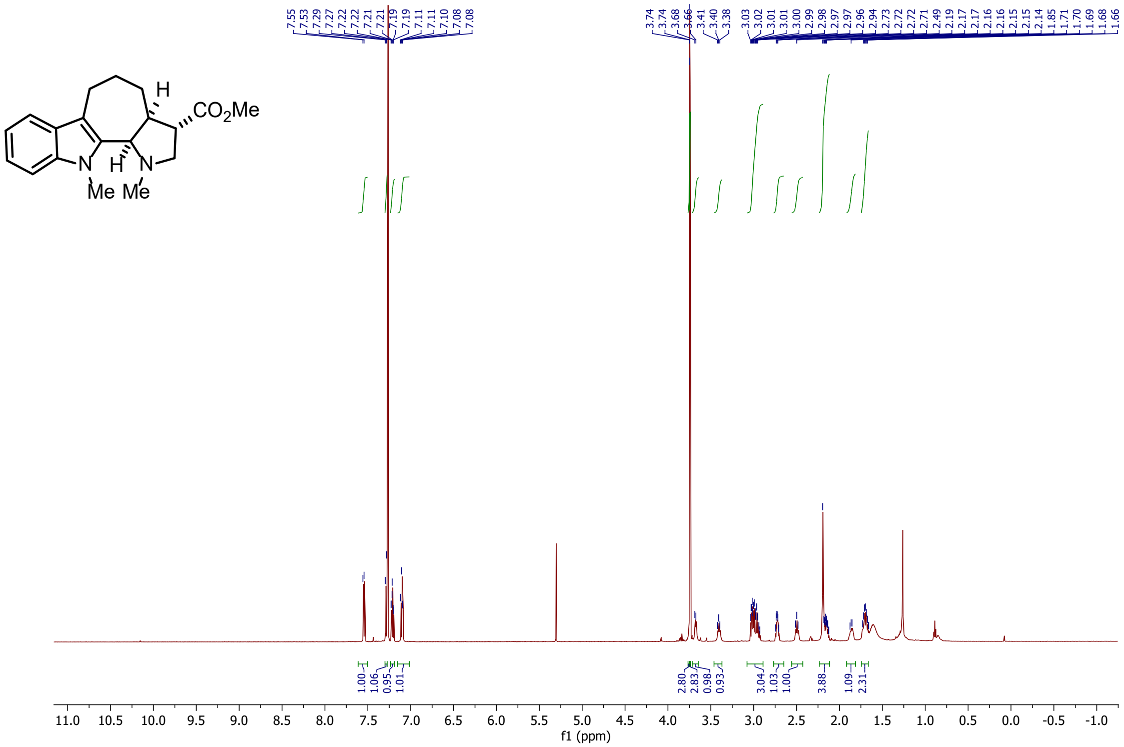

Figure 84: ***^{1}H NMR (600 MHz, CDCl$_3$) of 3.***

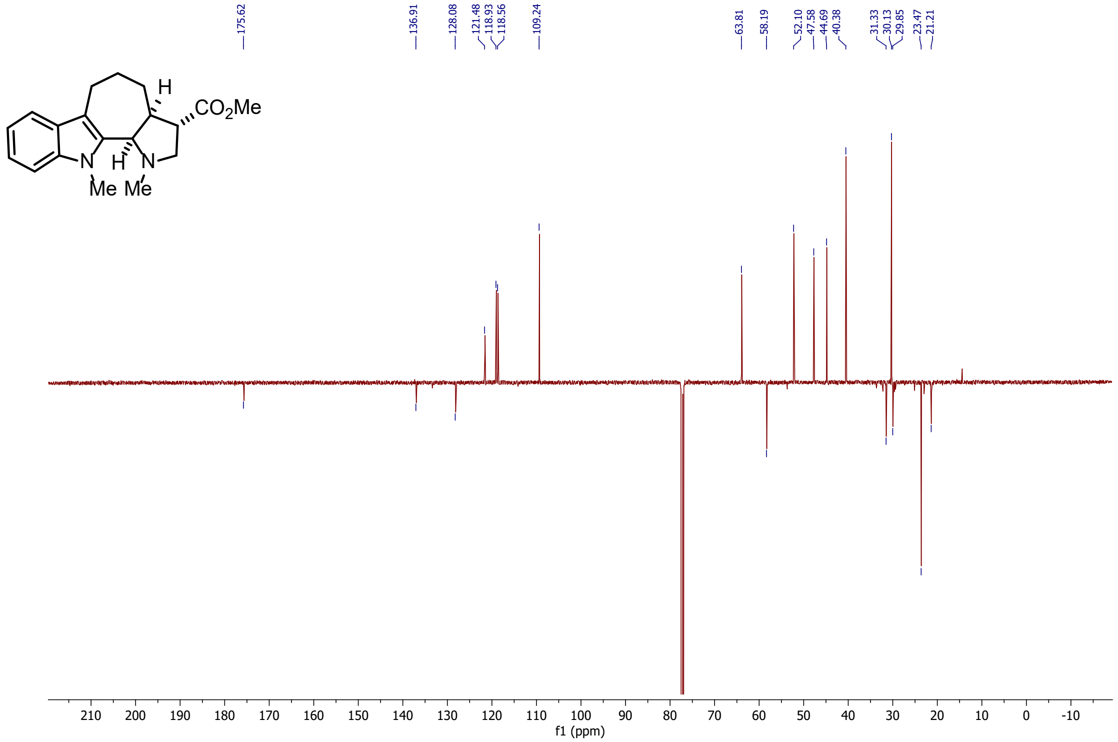

Figure 85: ***^{13}C NMR (151 MHz, CDCl$_3$) of 3.***

Methyl (3*S**,3a*S**,11b*R**)-8-bromo-1,11-dimethyl-2,3,3a,4,5,6,11,11b-octahydro-1*H*-pyrrolo[3',2':6,7]cyclohepta[1,2-b]indole-3-carboxylate (3^{Br})

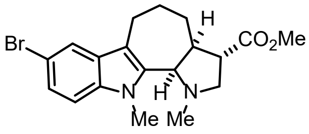

3^{Br}

Synthesized with deviations from the general procedure using $\mathbf{1^{Br}}$ (38 mg, 0.10 mmol, 1.0 eq.) and sarcosine **2a** (27 mg, 0.30 mmol, 3.0 eq.) by heating the reaction mixture at 120 °C. Purification by flash column chromatography (gradient of heptanes/EtOAc) gave $\mathbf{3^{Br}}$ as a light-yellow oil (7.0 mg, 0.018 mmol, 17% yield).

^{1}H NMR (600 MHz, $CDCl_3$) δ 7.64 (d, *J* = 1.8 Hz, 1H), 7.27 – 7.25 (m, 1H), 7.14 (d, *J* = 8.6 Hz, 1H), 3.74 (s, 3H), 3.71 (s, 3H), 3.64 (d, *J* = 7.9 Hz, 1H), 3.39 (t, *J* = 8.6 Hz, 1H), 3.03 – 2.98 (m, 1H), 2.90 (t, *J* = 6.6 Hz, 2H), 2.74 – 2.69 (m, 1H), 2.48 (t, *J* = 9.3 Hz, 1H), 2.17 (s, 3H), 2.15 – 2.09 (m, 1H), 1.87 – 1.81 (m, 1H), 1.69 – 1.62 (m, 2H). *Aromatic signal overlaps with the solvent peak.*

^{13}C NMR (151 MHz, $CDCl_3$) δ 175.5, 135.5, 134.8, 129.7, 124.2, 121.1, 113.6, 112.3, 110.7, 63.8, 58.2, 52.1, 47.4, 44.5, 40.4, 31.0, 30.3, 23.2, 21.0.

HRMS (ESI-TOF) m/z: $[M + H]^+$ Calcd for $C_{19}H_{24}{}^{79}BrN_2O_2$ 391.1016; Found 391.1012.

IR (neat) vmax: 2935, 1733, 1471, 1435, 1267, 1251, 1196, 999.

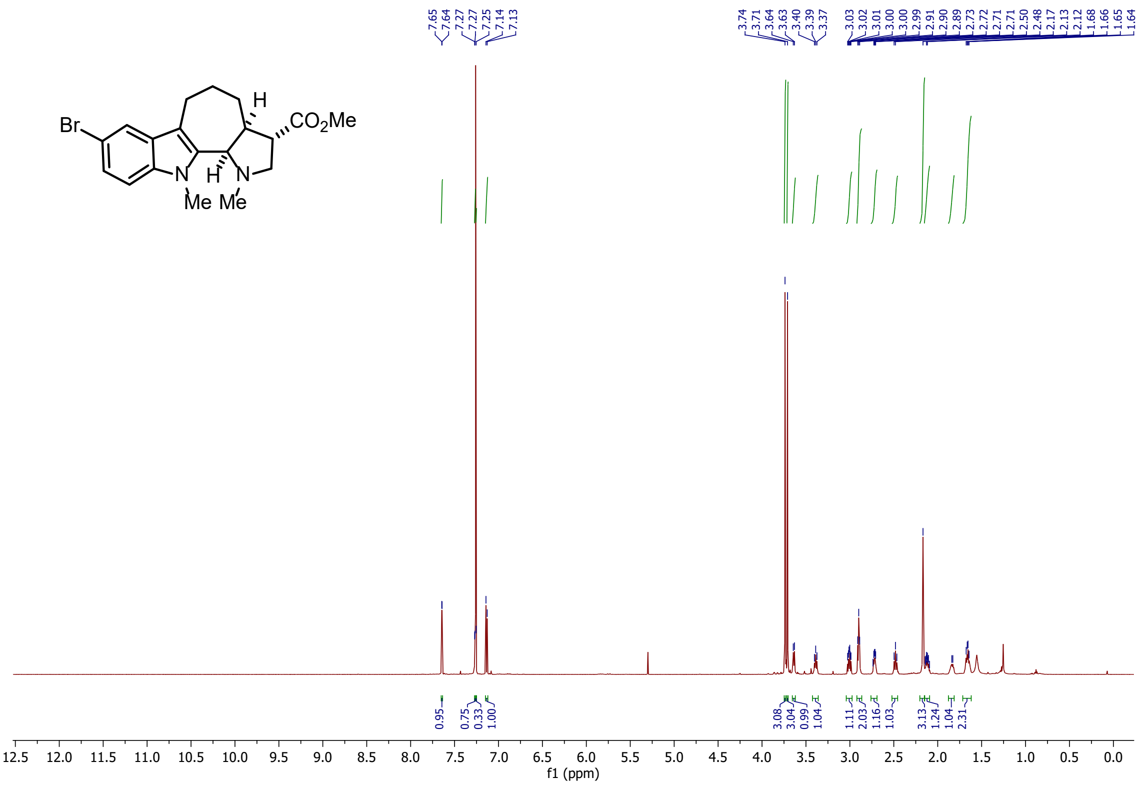

Figure 86: ***^{1}H NMR (600 MHz, CDCl$_3$) of 3^{Br}.***

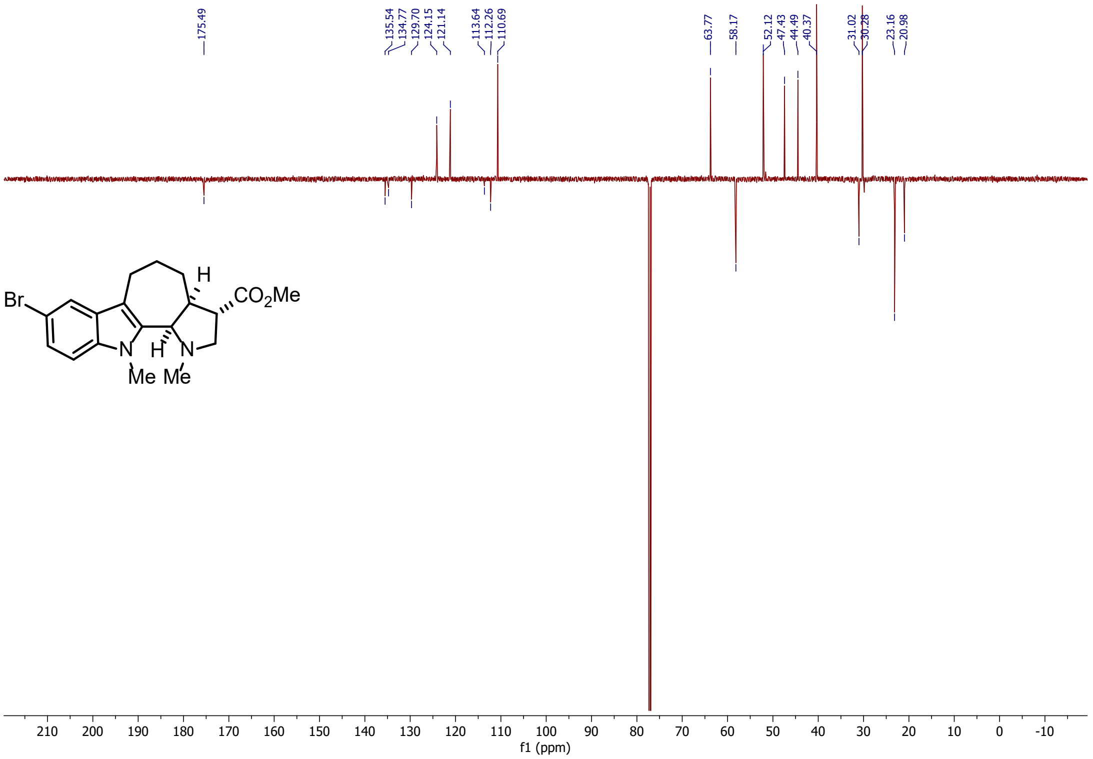

Figure 87: ***^{13}C NMR (151 MHz, CDCl$_3$) of 3^{Br}.***

Methyl (3*S**,3a*S**,11b*R**)-8-methoxy-1,11-dimethyl-2,3,3a,4,5,6,11,11b-octahydro-1*H*-pyrrolo[3',2':6,7]cyclohepta[1,2-b]indole-3-carboxylate (3^{OMe})

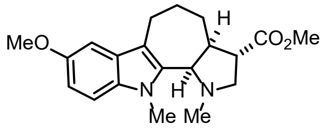

3^{OMe}

Synthesized with deviations from the general procedure using $\mathbf{1^{OMe}}$ (32 mg, 0.10 mmol, 1.0 eq.) and sarcosine **2a** (27 mg, 0.30 mmol, 3.0 eq.) by heating the reaction mixture at 120 °C. Purification by flash column chromatography (gradient of heptanes/EtOAc) gave $\mathbf{3^{OMe}}$ as a dark oil (5.5 mg, 0.016 mmol, 16% yield, 19% BRSM).

^{1}H NMR (600 MHz, $CDCl_3$) δ 7.17 (d, *J* = 8.8 Hz, 1H), 6.98 (d, *J* = 2.4 Hz, 1H), 6.87 (dd, *J* = 8.8, 2.4 Hz, 1H), 3.87 (s, 3H), 3.74 (s, 3H), 3.70 (s, 3H), 3.62 (d, *J* = 8.0 Hz, 1H), 3.62 (d, *J* = 8.0 Hz, 1H), 3.39 (t, *J* = 8.6 Hz, 1H), 3.04 – 2.98 (m, 1H), 2.92 (t, *J* = 6.7 Hz, 2H), 2.71 (td, *J* = 10.0, 5.5 Hz, 1H), 2.47 (t, *J* = 9.3 Hz, 1H), 2.19 – 2.11 (m, 4H), 1.88 – 1.82 (m, 1H), 1.69 (ddd, *J* = 15.4, 10.9, 4.8 Hz, 2H).

^{13}C NMR (151 MHz, $CDCl_3$) δ 175.4, 153.7, 133.9, 132.2, 128.0, 113.3, 111.5, 109.8, 100.2, 63.8, 58.0, 56.0, 51.9, 47.4, 44.5, 40.2, 31.1, 30.1, 23.4, 21.0.

HRMS (ESI-TOF) m/z: $[M + H]^+$ Calcd for $C_{20}H_{27}N_2O_3$ 343.2016; Found 343.2006.

IR (neat) νmax: 2924, 1730, 1619, 1487, 1455, 1214, 1193, 1157, 1036.

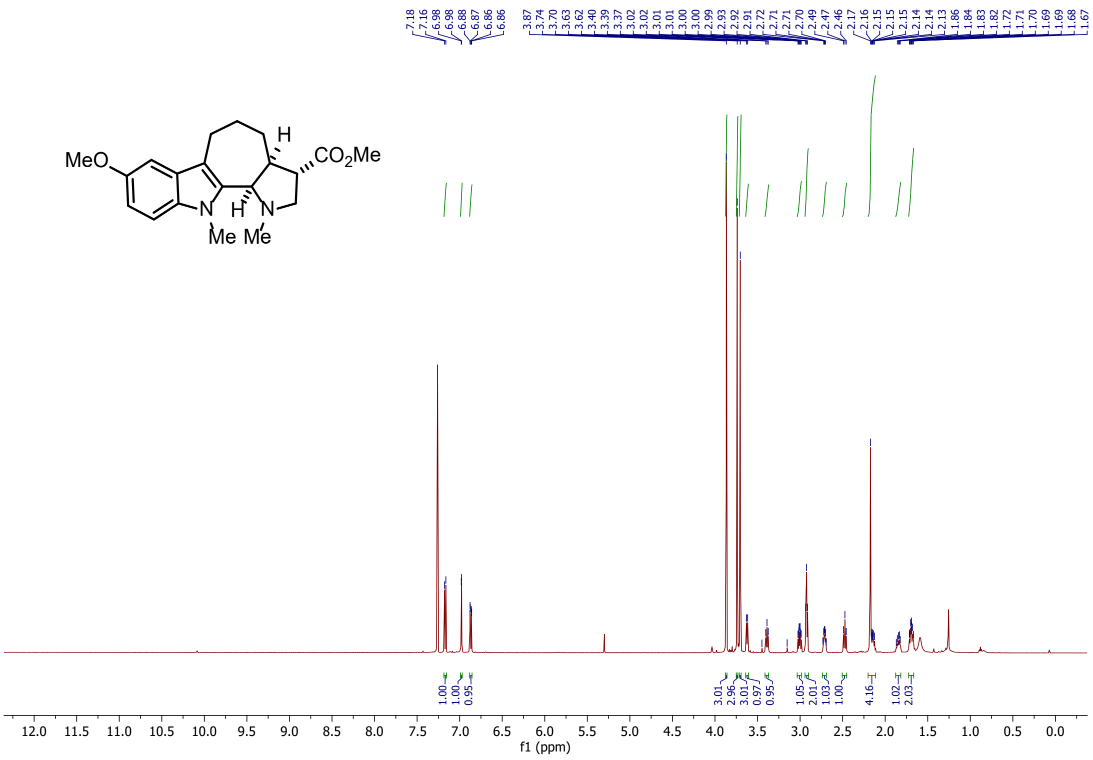

*Figure 88: ^{1}**H NMR (600 MHz, CDCl$_3$) of 3^{OMe}.***

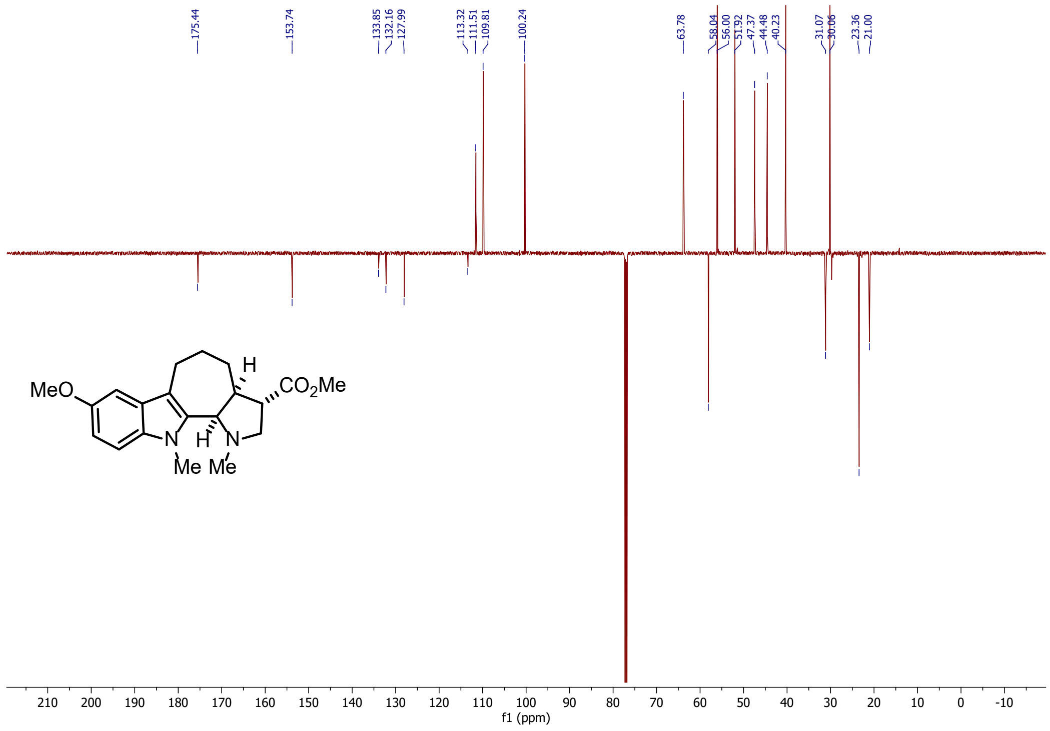

*Figure 89: ^{13}**C NMR (151 MHz, CDCl$_3$) of 3^{OMe}.***

Methyl (3*S,3a*R**,11b*R**)-1-methyl-5-tosyl-2,3,3a,4,5,6,11,11b-octahydro-1*H*-pyrrolo[2',3':5,6]azepino[4,3-b]indole-3-carboxylate (5a) and methyl (3*R**,3a*S**,11b*R**)-1-methyl-5-tosyl-2,3,3a,4,5,6,11,11b-octahydro-1*H*-pyrrolo[2',3':5,6]azepino[4,3-b]indole-3-carboxylate (5a')**

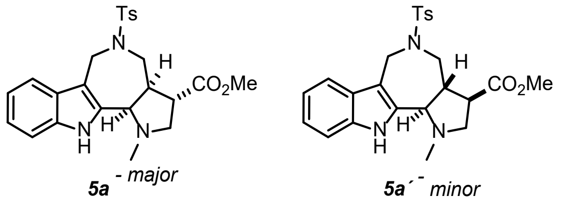

Synthesized following the general procedure using **4a** (426 mg, 1.00 mmol, 1.00 eq.) and sarcosine **2a** (267 mg, 3.00 mmol, 3.00 eq.). Purification by flash column chromatography (gradient of CH_2Cl_2/ DMA) gave **5a** major (423 mg, 0.93 mmol, 93% yield) and **5a´** minor diastereomer, as yellow oils (25 mg, 0.055 mmol, 6% yield).

Major diastereomer:

^{1}H NMR (600 MHz, $CDCl_3$) δ 8.63 (br s, 1H), 7.78 (d, J = 8.2 Hz, 2H), 7.37 – 7.30 (m, 4H), 7.15 (ddd, J = 8.0, 7.1, 0.9 Hz, 1H), 7.08 (ddd, J = 7.8, 6.8, 0.7 Hz, 1H), 4.73 (d, J = 14.0 Hz, 1H), 4.44 (d, J = 14.0 Hz, 1H), 4.02 (d, J = 6.6 Hz, 1H), 3.73 (s, 3H), 3.63 (q, J = 7.9 Hz, 1H), 3.43 (dd, J = 9.4, 7.3 Hz, 1H), 3.18 – 3.09 (m, 2H), 2.93 (dd, J = 15.8, 8.0 Hz, 1H), 2.71 (t, J = 9.8 Hz, 1H), 2.49 (s, 3H), 2.43 (s, 3H).

^{13}C NMR (176 MHz, $CDCl_3$) δ 173.4, 143.5, 135.6, 134.4, 134.2, 129.9 (2C), 127.9, 127.4 (2C), 121.8, 119.8, 117.8, 111.1, 104.7, 65.2, 58.6, 52.2, 48.2, 46.6, 46.2, 46.0, 43.1, 21.6.

HRMS (ESI-TOF) m/z: $[M + H]^+$ Calcd for $C_{24}H_{28}N_3O_4S$ 454.1795; Found 454.1790.

IR (neat) νmax: 3319, 2948, 2855, 1733, 1653, 1598, 1456, 1376, 1335, 1242, 1159, 1092.

Minor diastereomer:

^{1}H NMR (700 MHz, $CDCl_3$) δ 8.25 (br s, 1H), 7.66 (d, J = 7.7 Hz, 1H), 7.30 (d, J = 8.3 Hz, 2H), 7.28 (d, J = 8.0 Hz, 1H), 7.20 – 7.14 (m, 2H), 6.83 (d, J = 8.0 Hz, 2H), 5.32 (dd, J = 15.8, 0.7 Hz, 1H), 4.47 (ddd, J = 14.2, 2.9, 1.4 Hz, 1H), 4.21 (d, J = 16.0 Hz, 1H), 3.71 (s, 3H), 3.61 (d, J = 10.0 Hz, 1H), 3.58 (dd, J = 10.0, 4.6 Hz, 1H), 3.30 (dd, J = 14.2, 11.0 Hz, 1H), 2.64 (q, J = 10.1 Hz, 1H), 2.61 (td, J = 9.6, 4.6 Hz, 1H), 2.52 (s, 3H), 2.23 (s, 3H), 1.92 (qd, J = 10.5, 3.0 Hz, 1H).

^{13}C NMR (176 MHz, $CDCl_3$) 173.1, 142.9, 137.6, 137.3, 133.9, 129.1 (2C), 128.0, 127.2 (2C), 121.5, 120.5, 117.9, 111.1, 108.2, 70.3, 58.5, 53.6, 52.4, 46.8, 44.8, 43.5, 43.4, 21.5.

HRMS (ESI-TOF) m/z: $[M + H]^+$ Calcd for $C_{24}H_{28}N_3O_4S$ 454.1795; Found 454.1790.

IR (neat) νmax: 3318, 2949, 2852, 1731, 1466, 1450, 1437, 1360, 1333, 1307, 1282, 1255, 1227, 1196, 1154.

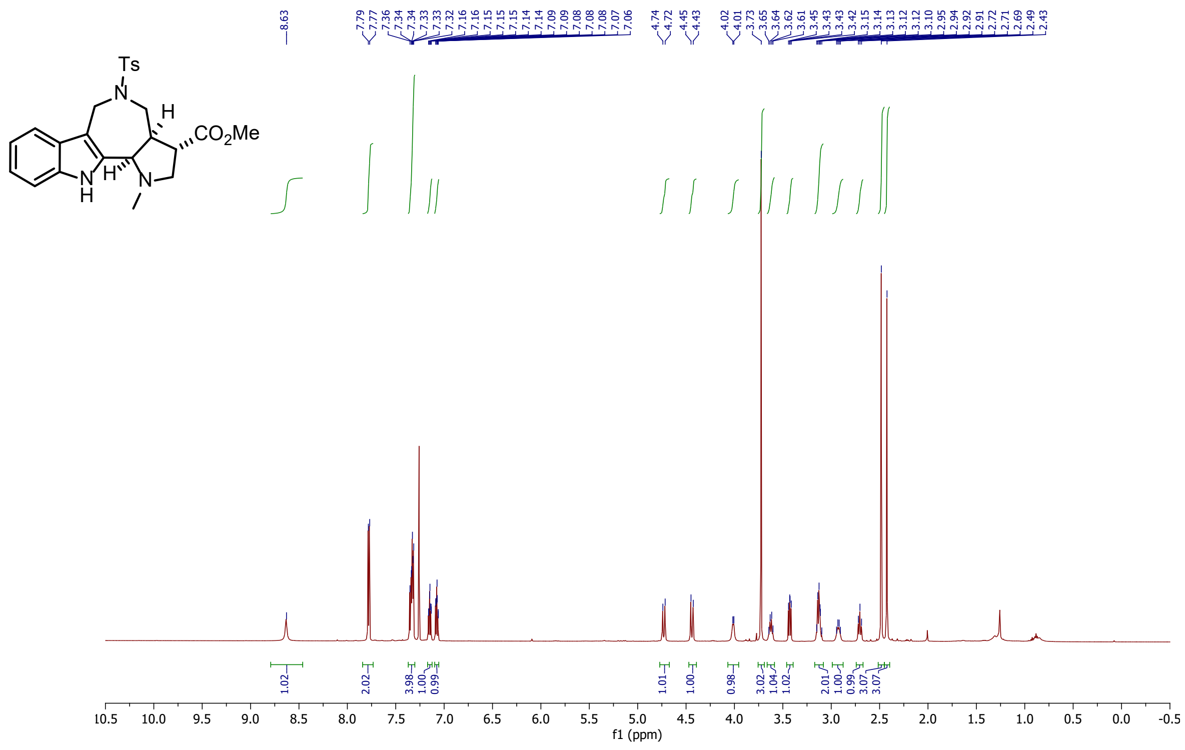

Figure 90: ***¹H NMR (600 MHz, CDCl₃) of 5a – major diastereomer.***

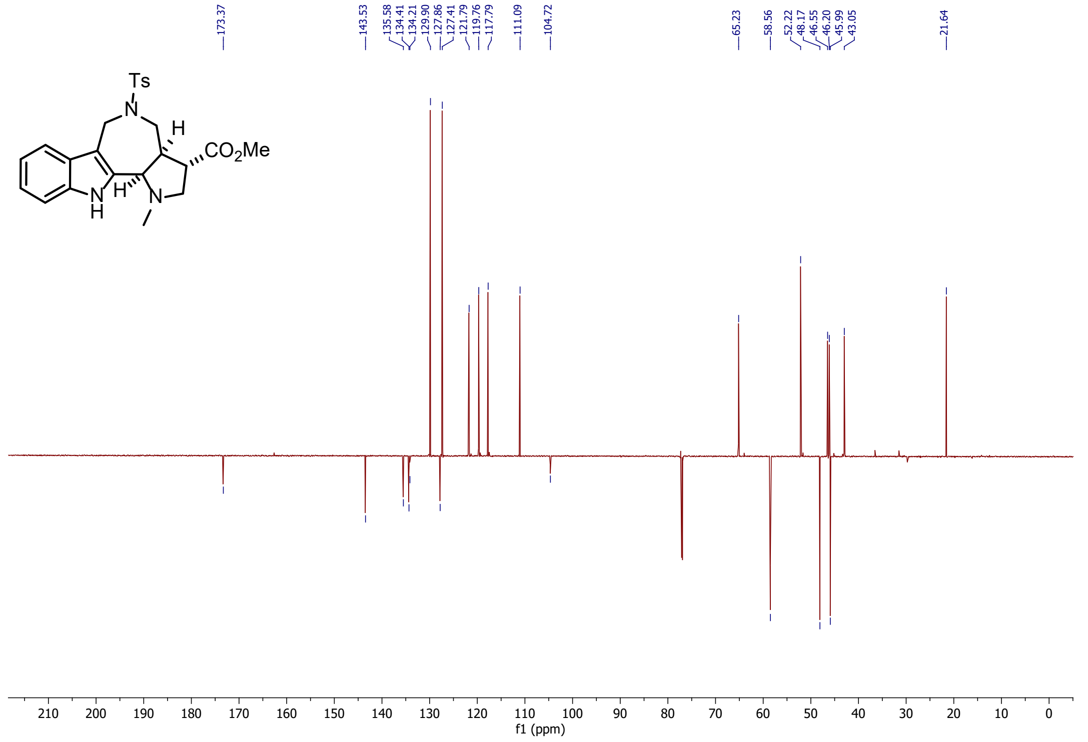

Figure 91: ***¹³C NMR (176 MHz, CDCl₃) of 5a – major diastereomer.***

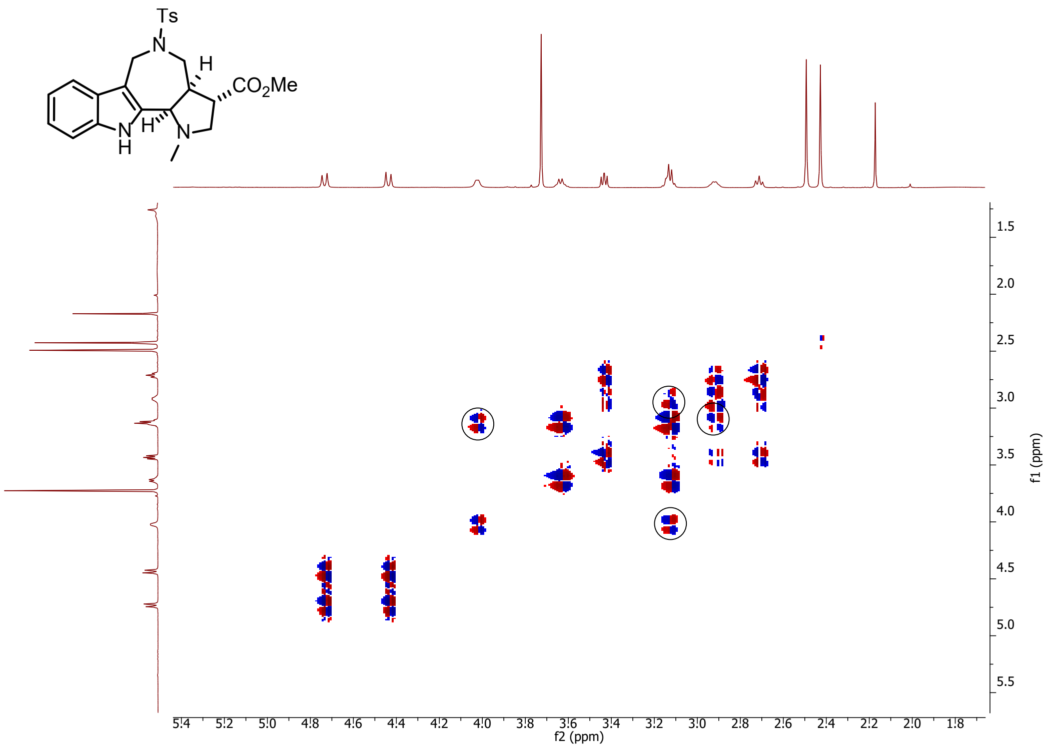

*Figure 92: **COSY (700 MHz, CDCl3) of 5a – major diastereomer.***

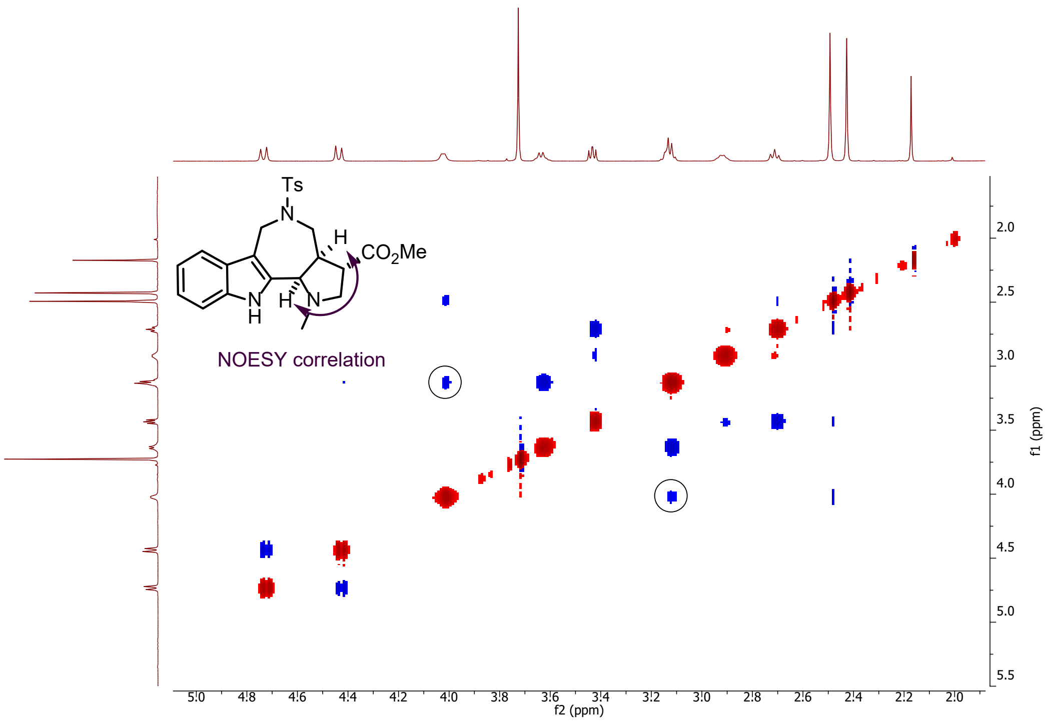

*Figure 93: **NOESY (700 MHz, CDCl3) of 5a – major diastereomer.***

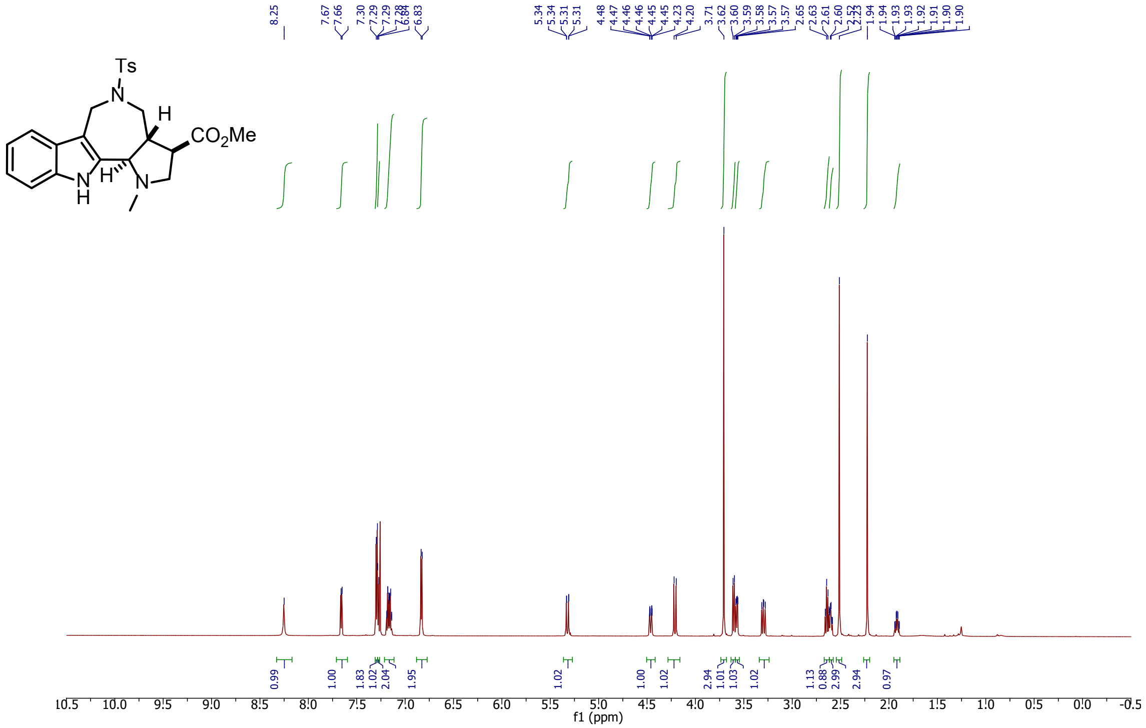

*Figure 94: **¹H NMR (700 MHz, CDCl₃) of 5a – minor diastereomer.***

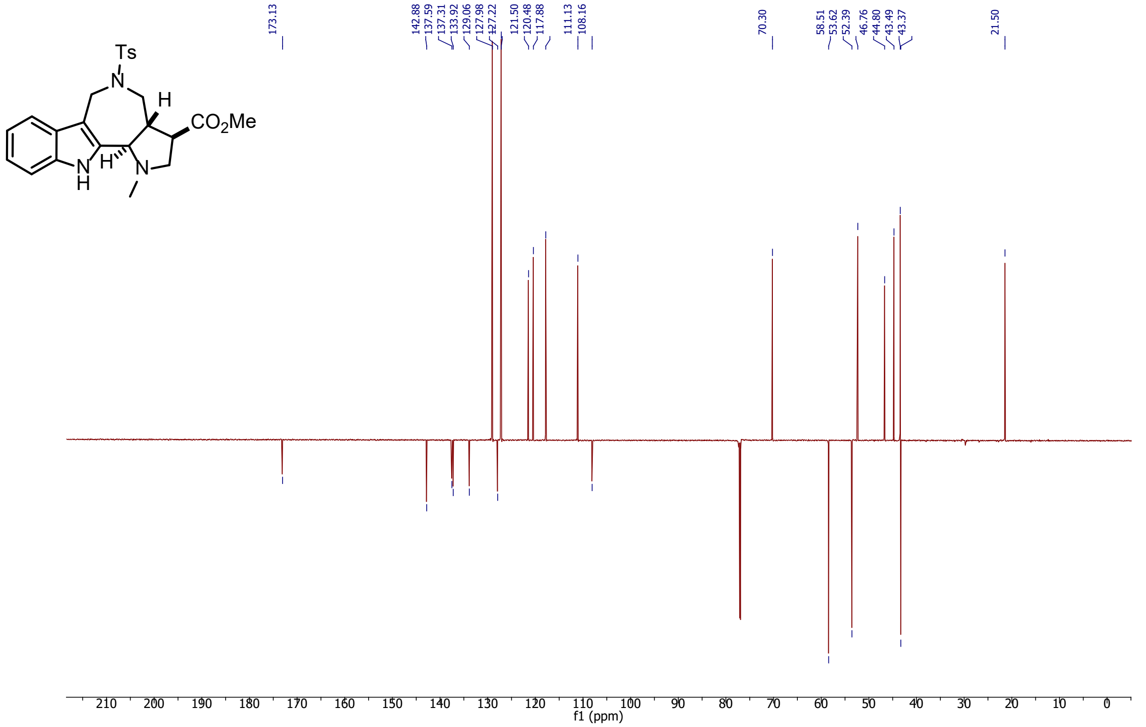

*Figure 95: **¹³C NMR (176 MHz, CDCl₃) of 5a – minor diastereomer.***

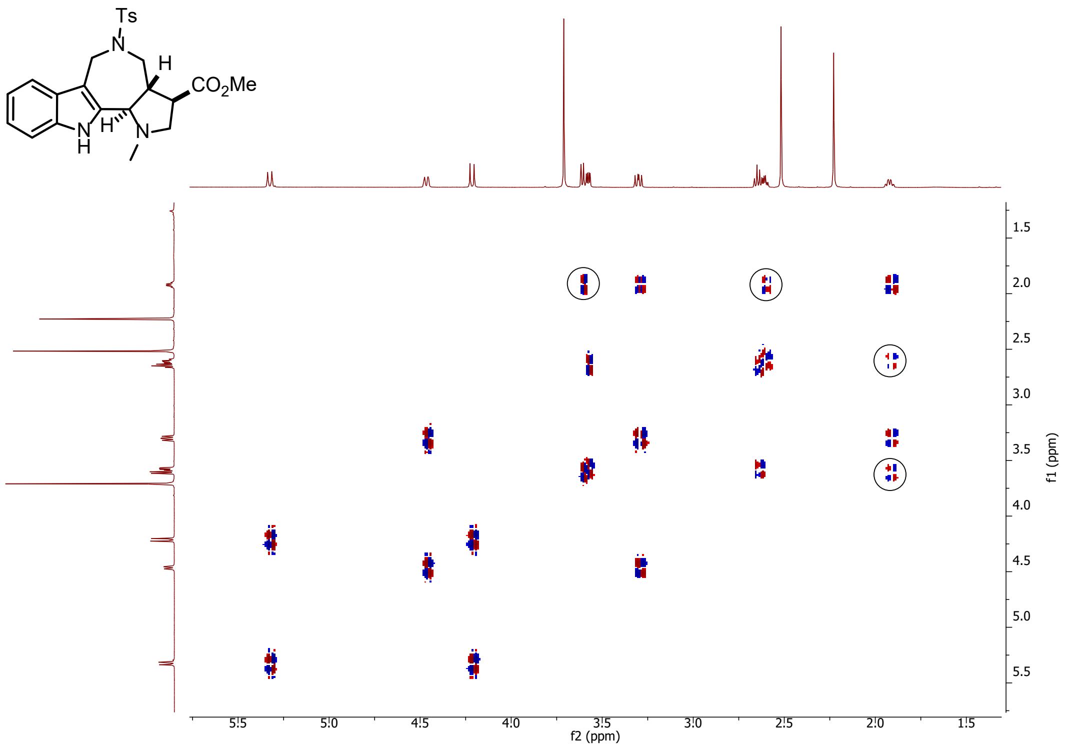

Figure 96: ***COSY (700 MHz, CDCl3) of 5a – minor diastereomer.***

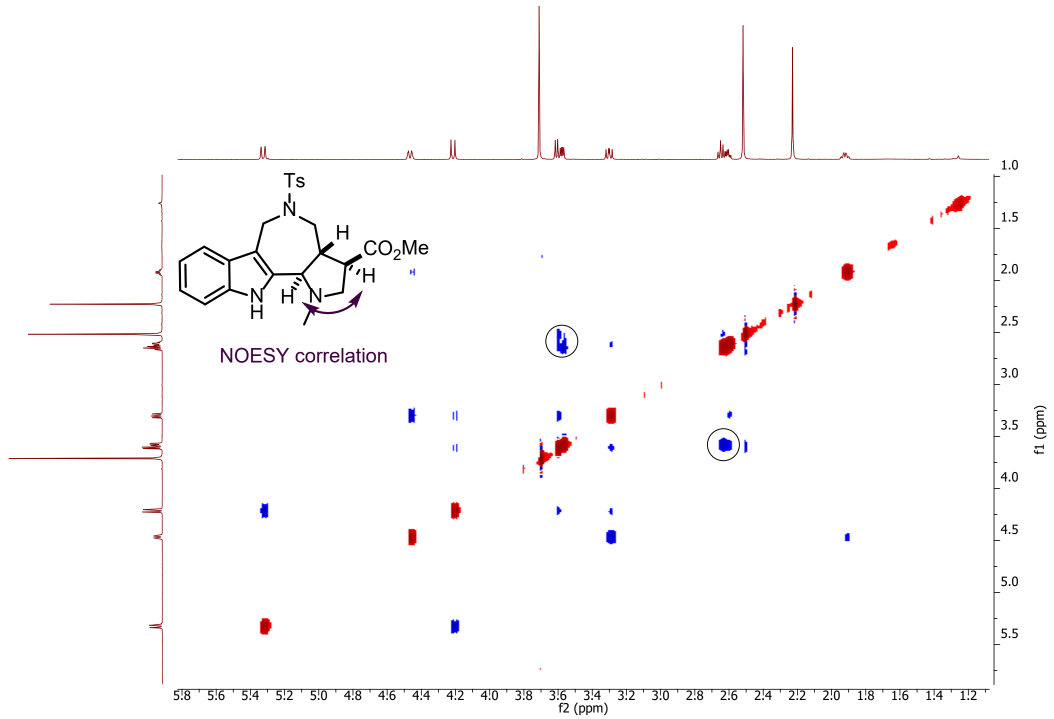

Figure 97: ***NOESY (700 MHz, CDCl3) of 5a – minor diastereomer.***

Methyl (3*S,3a*R**,11b*R**)-1,11-dimethyl-5-tosyl-2,3,3a,4,5,6,11,11b-octahydro-1*H*-pyrrolo[2',3':5,6]azepino[4,3-b]indole-3-carboxylate (5b)**

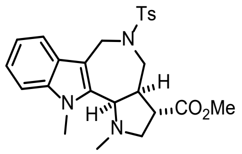

5b

Synthesized following the general procedure using **4b** (34 mg, 0.080 mmol, 1.0 eq.) and sarcosine **2a** (21 mg, 0.24 mmol, 3.0 eq.). Purification by flash column chromatography (gradient of heptane/EtOAc) gave **5b** as a yellow oil (21 mg, 0.045 mmol, 56% yield, d.r. 7.5:1). The minor diasteromer could not be isolated.

^{1}H NMR (600 MHz, CDCl$_3$) δ 7.73 (d, *J* = 8.2 Hz, 2H), 7.34 (d, *J* = 7.9 Hz, 1H), 7.28 (d, *J* = 8.2 Hz, 1H), 7.24 (*overlap with CDCl$_3$*, 1H), 7.20 (app. t, *J* = 7.5 Hz, 1H), 7.09 (app. t, *J* = 7.4 Hz, 1H), 4.81 (d, *J* = 13.8 Hz, 1H), 4.57 (d, *J* = 13.8 Hz, 1H), 3.77 (s, 3H), 3.76 – 3.73 (m, 1H), 3.72 (s, 3H), 3.70 – 3.64 (m, 1H), 3.62 (d, *J* = 8.9 Hz, 1H), 3.45 (app. t, *J* = 8.5 Hz, 1H), 2.92 – 2.86 (m, 1H), 2.84 – 2.78 (m, 1H), 2.43 – 2.35 (m, 4H), 2.17 (s, 3H).

^{13}C NMR (151 MHz, CDCl$_3$) δ 174.8, 143.3, 136.6, 135.9, 135.5, 129.7 (2C), 127.3 (2C), 126.5, 121.9, 119.8, 117.9, 109.6, 107.0, 63.0, 58.1, 52.2, 47.6, 44.5, 44.0, 43.3, 40.3, 29.9, 21.6.

HRMS (ESI-TOF) m/z: $[M + H]^+$ Calcd for $C_{25}H_{30}N_3O_4S$ 468.1952; Found 468.1953.

IR (neat) νmax: 3048, 2945, 2835, 2779, 2255, 1730, 1469, 1153, 908, 728, 665, 548.

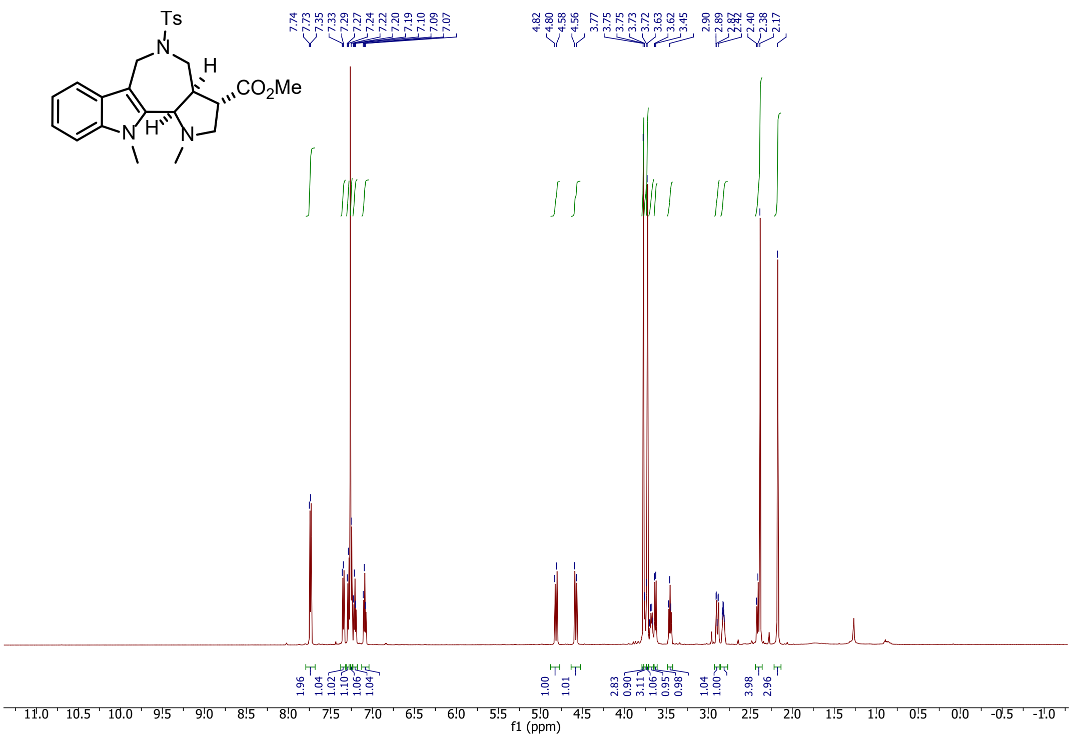

Figure 98: ***^{1}H NMR (600 MHz, CDCl3) of 5b.***

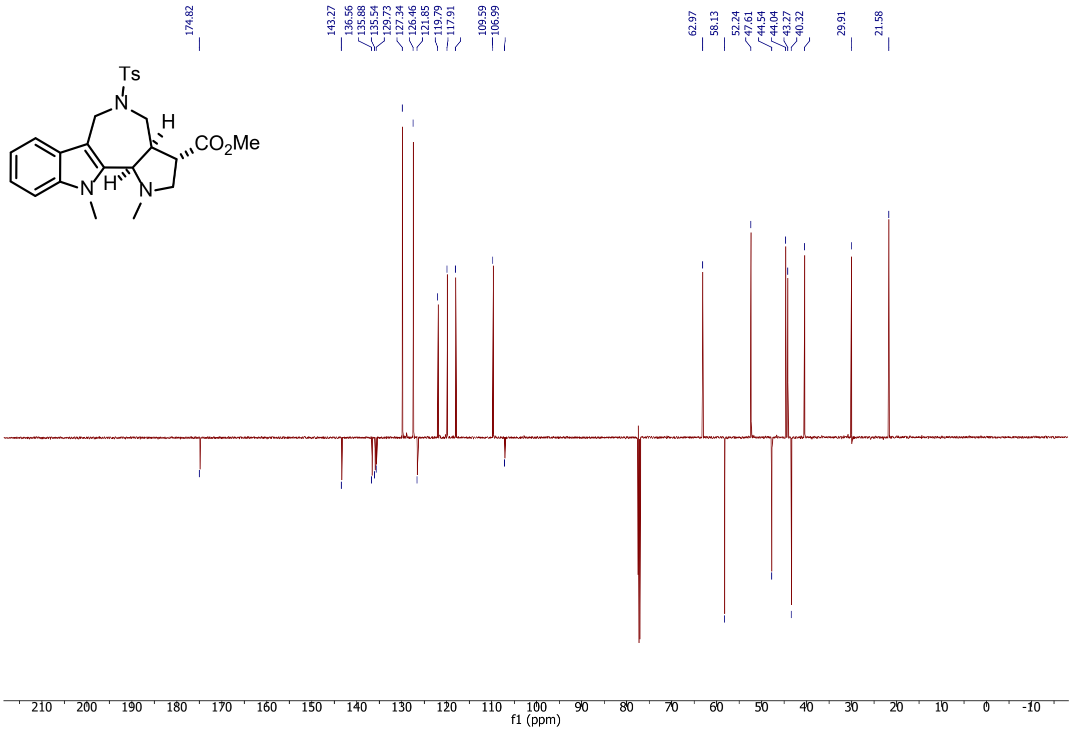

Figure 99: ***^{13}C NMR (151 MHz, CDCl3) of 5b.***

Methyl (3*S**,3a*R**,11b*R**)-8-methoxy-1-methyl-5-tosyl-2,3,3a,4,5,6,11,11b-octahydro-1*H*-pyrrolo[2',3':5,6]azepino[4,3-b]indole-3-carboxylate (5c)

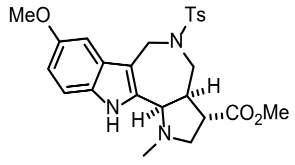

5c

Synthesized following the general procedure using **4c** (9 mg, 0.02 mmol, 1.0 eq.) and sarcosine **2a** (5 mg, 0.06 mmol, 3.0 eq.). Purification by flash chromatography (gradient of heptanes/EtOAc/EtOH) gave **5c** as an orange solid (5 mg, 0.01 mmol, 56% yield, d.r >20:1).

^{1}H NMR (600 MHz, CDCl$_3$) δ 8.48 (s, 1H), 7.78 (t, *J* = 7.8 Hz, 2H), 7.32 (d, *J* = 7.9 Hz, 2H), 7.22 (d, *J* = 8.7 Hz, 1H), 6.81 (dd, *J* = 8.7, 2.4 Hz, 1H), 6.77 (d, *J* = 2.3 Hz, 1H), 4.70 (d, *J* = 13.9 Hz, 1H), 4.40 (d, *J* = 14.0 Hz, 1H), 4.01 – 3.95 (br s, *J* = 6.6 Hz, 1H), 3.83 (s, 3H), 3.72 (s, 3H), 3.68 – 3.62 (m, 1H), 3.42 (dd, *J* = 9.4, 7.3 Hz, 1H), 3.15 – 3.08 (m, 2H), 2.93 – 2.85 (br s, 1H), 2.70 (t, *J* = 9.7 Hz, 1H), 2.48 (s, 3H), 2.43 (s, 3H).

^{13}C NMR (151 MHz, CDCl$_3$) δ 173.3, 154.3, 143.5, 135.7, 135.0*, 129.9 (2C), 129.5, 128.2, 127.5 (2C), 111.88, 111.85, 104.5*, 99.9, 65.2, 58.5, 56.1, 52.3, 48.1, 46.5, 46.2, 45.9, 43.0, 21.7. **Low intensity*

HRMS (ESI-TOF) m/z: [M + H]$^+$ Calcd for $C_{25}H_{30}N_3O_5S$ 484.1901; Found 484.1901.

IR (neat) νmax: 3369, 2948, 2928, 2848, 2784, 1731, 1668, 1625, 1596, 1486, 1455, 1436, 1374, 1333, 1304.

Mp.: 131-133 °C.

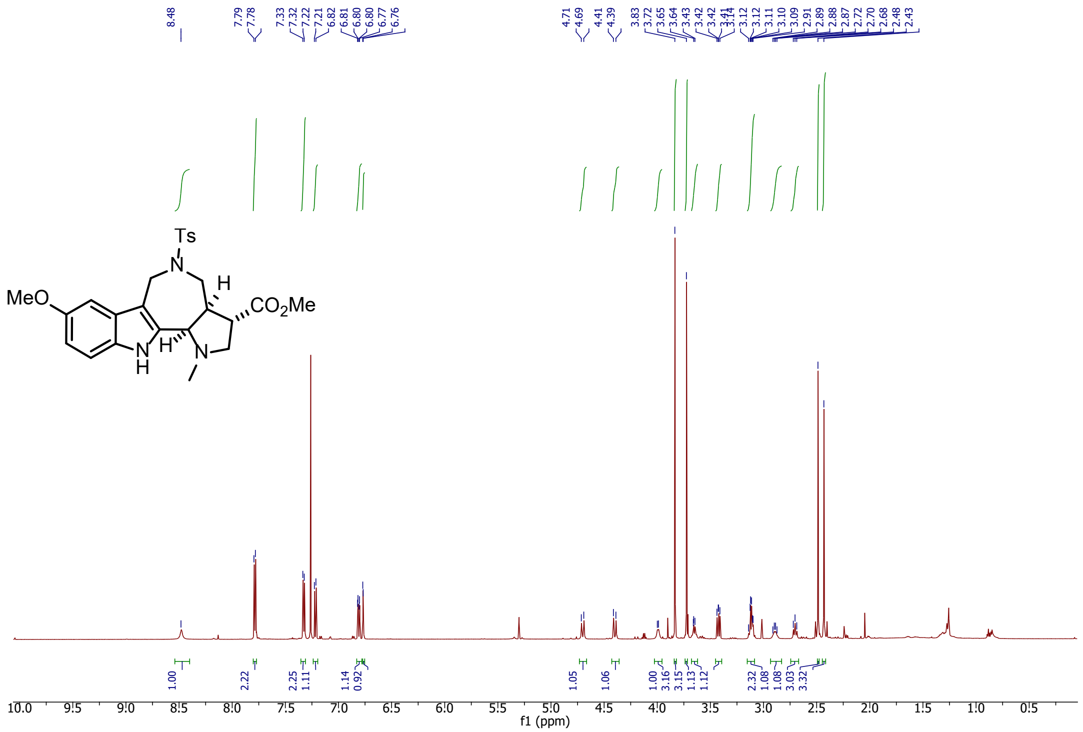

Figure 100: ^{1}H NMR (600 MHz, CDCl$_3$) of 5c.

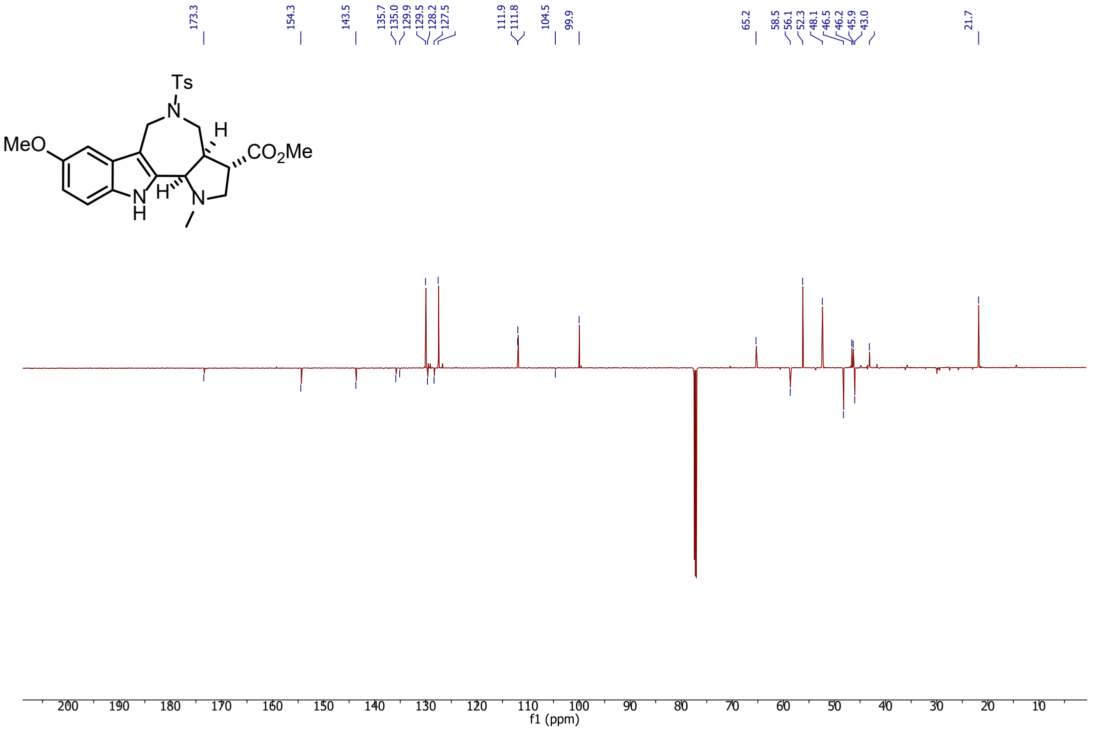

Figure 101: ^{13}C NMR (151 MHz, CDCl$_3$) of 5c.

Methyl (3*S**,3a*R**,11b*R**)-8-bromo-1-methyl-5-tosyl-2,3,3a,4,5,6,11,11b-octahydro-1*H*-pyrrolo[2',3':5,6]azepino[4,3-b]indole-3-carboxylate (5d)

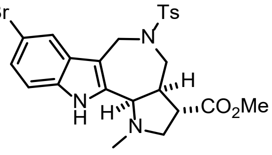

5d

Synthesized following the general procedure using **4d** (30 mg, 0.059 mmol, 1.0 eq.) and sarcosine **2a** (16 mg, 0.18 mmol, 3.0 eq.). Purification by flash chromatography (gradient of heptanes/EtOAc) gave **5d** as a mixture of diastereomers (23 mg, 0.043 mmol, 73% yield, d.r. 12:1). A second purification by flash chromatography (gradient of CH_2Cl_2/DMA) yielded **5d** as a white solid as the major diastereomer (20 mg, 0.037 mmol, 63% yield). The minor diastereomer was not isolated.

^{1}H NMR (400 MHz, $CDCl_3$) δ 8.69 (s, 1H), 7.80 – 7.72 (m, 2H), 7.46 – 7.40 (m, 1H), 7.34 (d, J = 8.0 Hz, 2H), 7.24 – 7.15 (m, 2H), 4.71 (d, J = 14.1 Hz, 1H), 4.29 (d, J = 14.1 Hz, 1H), 4.10 (d, J = 8.9 Hz, 1H), 3.72 (s, 3H), 3.72 – 3.66 (m, 1H), 3.43 (dd, J = 8.8, 6.6 Hz, 1H), 3.24 – 3.12 (m, 1H), 3.00 (dd, J = 14.3, 9.1 Hz, 1H), 2.87 – 2.67 (m, 2H), 2.52 (s, 3H), 2.44 (s, 3H).

^{13}C NMR (101 MHz, $CDCl_3$) δ 173.1, 143.7, 135.9, 135.4, 133.0, 130.0 (2C), 129.6, 127.4 (2C), 124.6, 120.5, 113.0, 112.5, 104.2, 65.0, 58.6, 52.3, 48.2, 46.8, 46.4, 45.9, 43.3, 21.7.

HRMS (ESI-TOF) m/z: $[M + H]^+$ Calcd for $C_{24}H_{27}{}^{79}BrN_3O_4SNa$ 534.0882; Found 534.0879.

IR (neat) νmax: 3353, 2948, 2843, 2788, 1729, 1597, 1437, 1049.

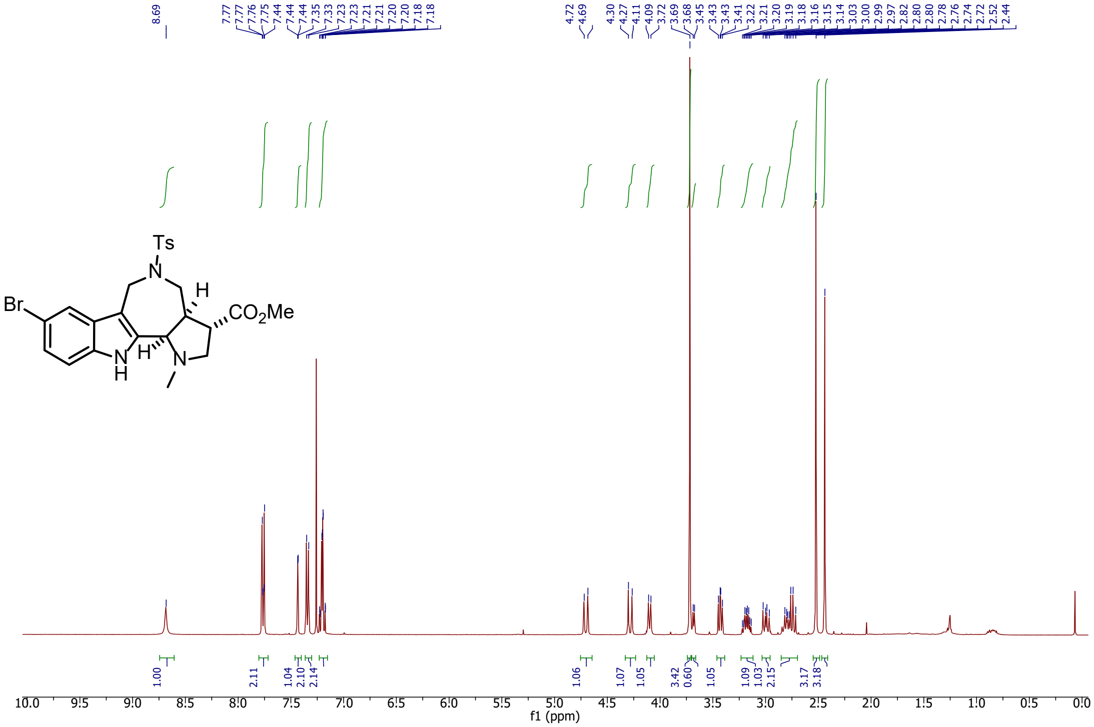

*Figure 102: ^{1}**H NMR (400 MHz, CDCl3) of 5d.***

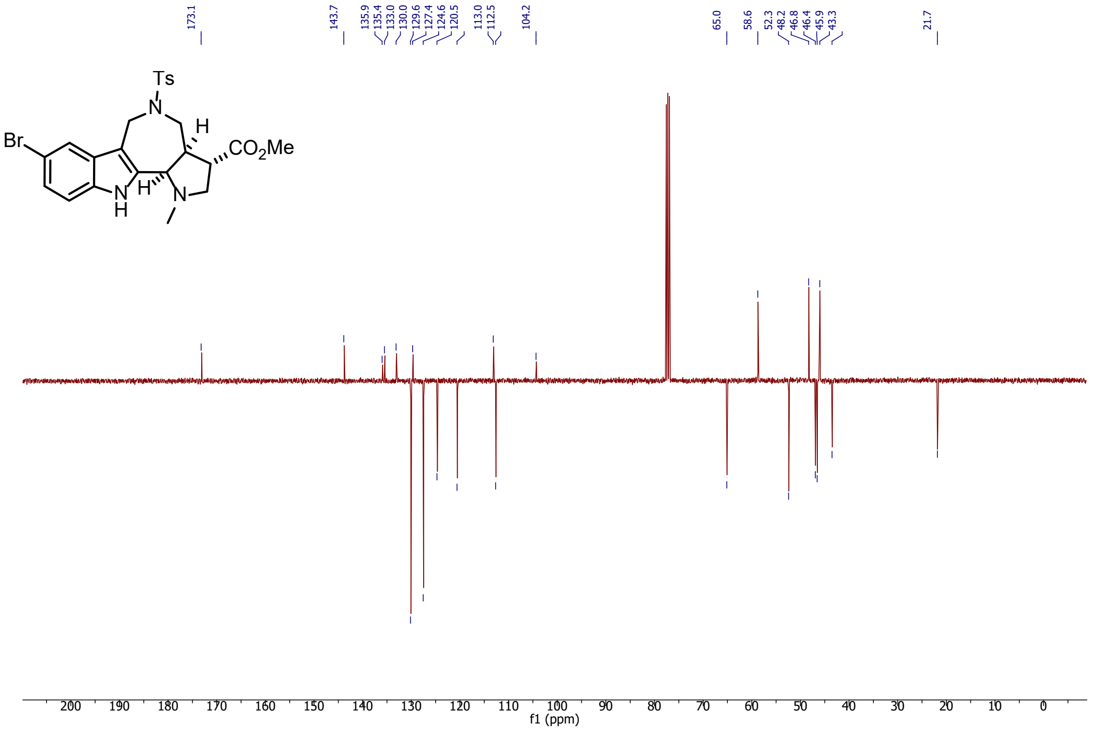

*Figure 103: ^{13}**C NMR (101 MHz, CDCl3) of 5d.***

Methyl (3*S,3a*R**,11b*R**)-8-chloro-1-methyl-5-tosyl-2,3,3a,4,5,6,11,11b-octahydro-1*H*-pyrrolo[2',3':5,6]azepino[4,3-b]indole-3-carboxylate (5e)**

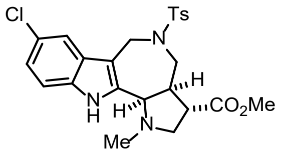

5e

Synthesized following the general procedure using **4e** (16 mg, 0.035 mmol, 1.0 eq.) and sarcosine **2a** (9 mg, 0.10 mmol, 3.0 eq.). Purification by flash column chromatography (gradient of CH_2Cl_2/DMA) yielded **5e** as an off-white oil as the major diastereomer (10 mg, 0.02 mmol, 59% yield, d.r. 10:1). The minor diastereomer was not isolated.

^{1}H NMR (600 MHz, $CDCl_3$) δ 8.67 (br s, 1H), 7.76 (d, J = 8.2 Hz, 1H), 7.34 (d, J = 8.0 Hz, 2H), 7.28 (d, J = 1.6 Hz, 1H), 7.23 (d, J = 8.6 Hz, 1H), 7.09 (dd, J = 8.6, 1.9 Hz, 1H), 4.71 (d, J = 14.1 Hz, 1H), 4.29 (d, J = 14.1 Hz, 1H), 4.09 (d, J = 9.0 Hz, 1H), 3.74 – 3.67 (m, 4H), 3.43 (dd, J = 9.2, 7.1 Hz, 1H), 3.18 (qd, J = 9.3, 5.2 Hz, 1H), 3.00 (dd, J = 14.4, 9.2 Hz, 1H), 2.81 (dd, J = 17.1, 9.9 Hz, 1H), 2.74 (t, J = 9.7 Hz, 1H), 2.52 (s, 3H), 2.44 (s, 3H).

^{13}C NMR (151 MHz, $CDCl_3$) δ 173.1, 143.7, 136.0, 135.4, 132.7, 130.0, 128.9, 127.4, 125.5, 122.0, 117.4, 112.1, 104.3, 65.0, 58.6, 52.3, 48.2, 46.8, 46.4, 45.9, 43.4, 21.7.

HRMS (ESI-TOF) m/z: $[M + H]^+$ Calcd for $C_{24}H_{27}ClN_3O_4S$ 488.1405; Found 488.1405.

IR (neat) vmax: 3353, 2950, 1730, 1664, 1448, 1373, 1305, 1291, 1266, 1197, 1090, 1017.

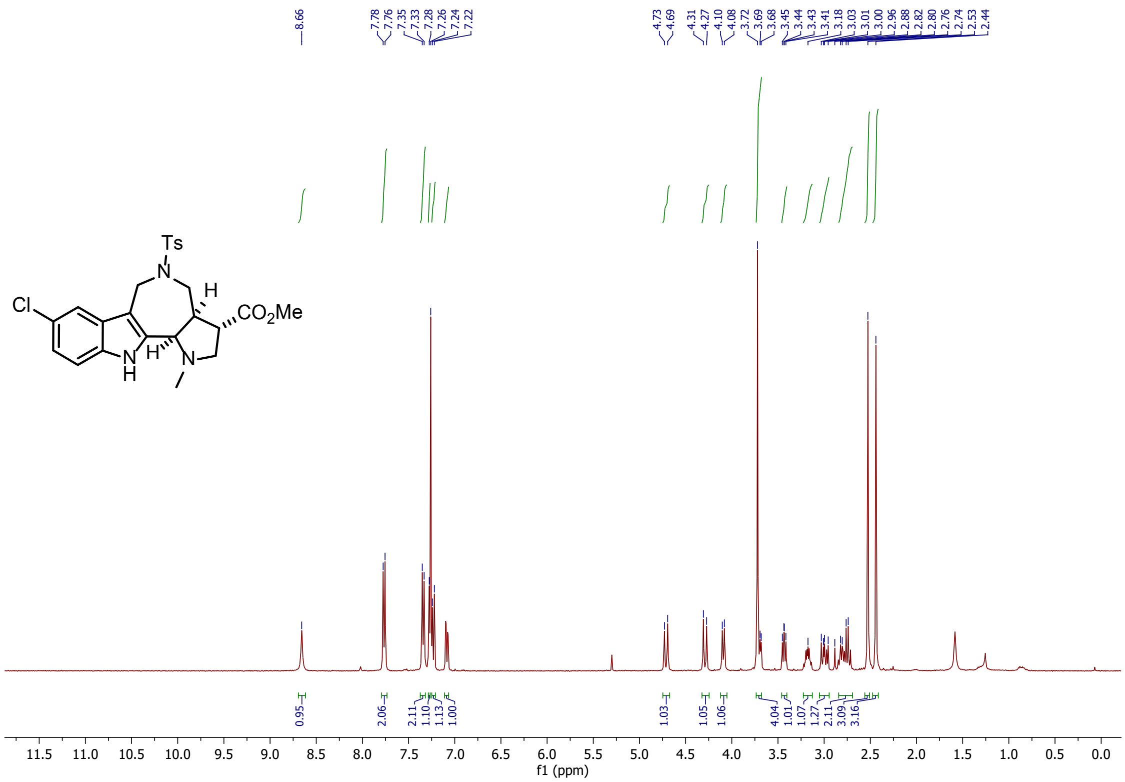

Figure 104: ***^{1}H NMR (600 MHz, $CDCl_3$) of 5e.***

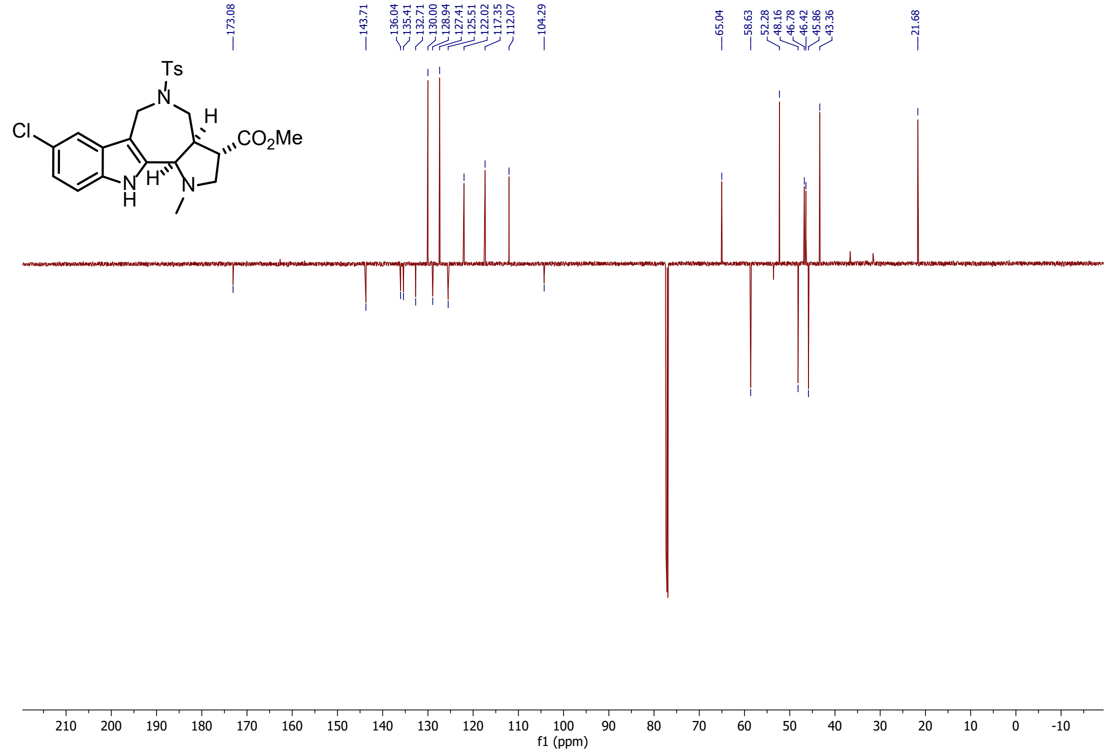

Figure 105: ***^{13}C NMR (151 MHz, $CDCl_3$) of 5e***

Methyl (3*S**,3a*S**,11b*R**)-1-methyl-1,2,3,3a,4,6,11,11b-octahydropyrrolo[2',3':5,6]oxepino[4,3-b]indole-3-carboxylate (5f) and methyl (3*R**,3a*R**,11b*R**)-1-methyl-1,2,3,3a,4,6,11,11b-octahydropyrrolo[2',3':5,6]oxepino[4,3-b]indole-3-carboxylate (5f')

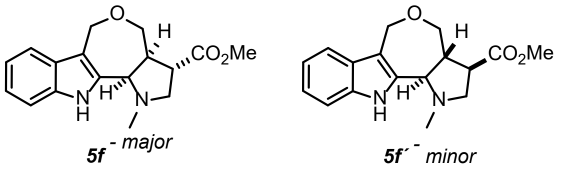

Synthesized following the general procedure using **4f** (78 mg, 0.29 mmol, 1.0 eq.) and sarcosine **2a** (77 mg, 0.86 mmol, 3.0 eq.). Purification by flash column chromatography (gradient of CH_2Cl_2/DMA) yielded **5f** as a pale-orange oil as the major diastereomer (37 mg, 0.12 mmol, 43% yield) and **5f'** as a pale-orange oil as the minor diastereomer (7.4 mg, 0.025 mmol, 9% yield).

Major diastereomer:

^{1}H NMR (600 MHz, $CDCl_3$) δ 8.33 (s, 1H), 7.40 (d, J = 7.9 Hz, 1H), 7.34 (d, J = 8.1 Hz, 1H), 7.20 – 7.16 (m, 1H), 7.12 – 7.08 (m, 1H), 5.11 (d, J = 14.4 Hz, 1H), 5.02 (d, J = 14.4 Hz, 1H), 3.94 (qd, J = 12.8, 4.3 Hz, 2H), 3.75 – 3.69 (m, 4H), 3.39 (dd, J = 9.7, 7.9 Hz, 1H), 3.15 (dd, J = 17.3, 7.9 Hz, 1H), 2.94 (ddd, J = 12.6, 8.3, 4.3 Hz, 1H), 2.58 (t, J = 9.6 Hz, 1H), 2.42 (s, 3H).

^{13}C NMR (151 MHz, $CDCl_3$) δ 174.6, 135.1, 132.9, 127.1, 122.1, 119.7, 118.4, 111.0, 110.9, 70.4, 67.7, 66.9, 58.2, 52.2, 47.1, 45.4, 42.3.

HRMS (ESI-TOF) m/z: $[M + H]^+$ Calcd for $C_{17}H_{21}N_2O_3$ 301.1547; Found 301.1547.

IR (neat) vmax: 3264, 2929, 1731, 1458, 1397, 1340, 1262, 1238, 1163, 1123, 1090.

Minor diastereomer:

^{1}H NMR (600 MHz, $CDCl_3$) δ 8.47 (br s, 1H), 7.51 – 7.49 (m, 1H), 7.34 (dd, J = 6.3, 2.2 Hz, 1H), 7.15 – 7.10 (m, 2H), 5.10 (d, J = 14.1 Hz, 1H), 4.65 (d, J = 14.1 Hz, 1H), 4.47 (dd, J = 12.0, 3.2 Hz, 1H), 3.88 – 3.80 (m, 2H), 3.70 – 3.67 (m, 4H), 2.77 – 2.72 (m, 1H), 2.68 – 2.62 (m, 4H), 2.51 (ddd, J = 13.1, 10.3, 3.3 Hz, 1H).

^{13}C NMR (151 MHz, $CDCl_3$) δ 173.5, 138.9, 134.3, 127.7, 121.3, 120.2, 117.4, 111.3, 111.0, 76.0, 70.9, 64.8, 59.1, 52.4, 50.3, 44.0, 43.9.

HRMS (ESI-TOF) m/z: $[M +H]^+$ Calcd for $C_{17}H_{21}N_2O_3$ 301.1547; Found 301.1542.

IR (neat) vmax: 3279, 2848, 1725, 1448, 1435, 1365, 1266, 1193, 1094, 1077, 994.

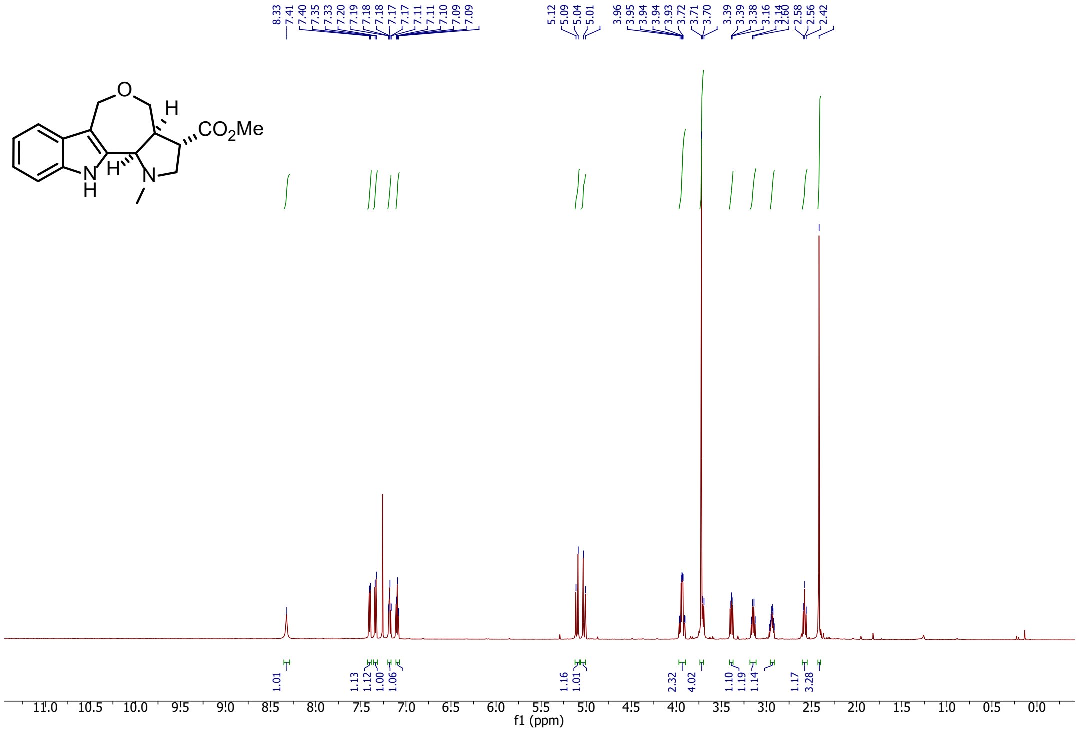

Figure 106: ¹H NMR (600 MHz, CDCl₃) of 5f – major diastereomer.

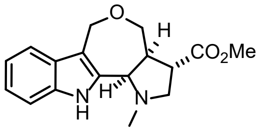

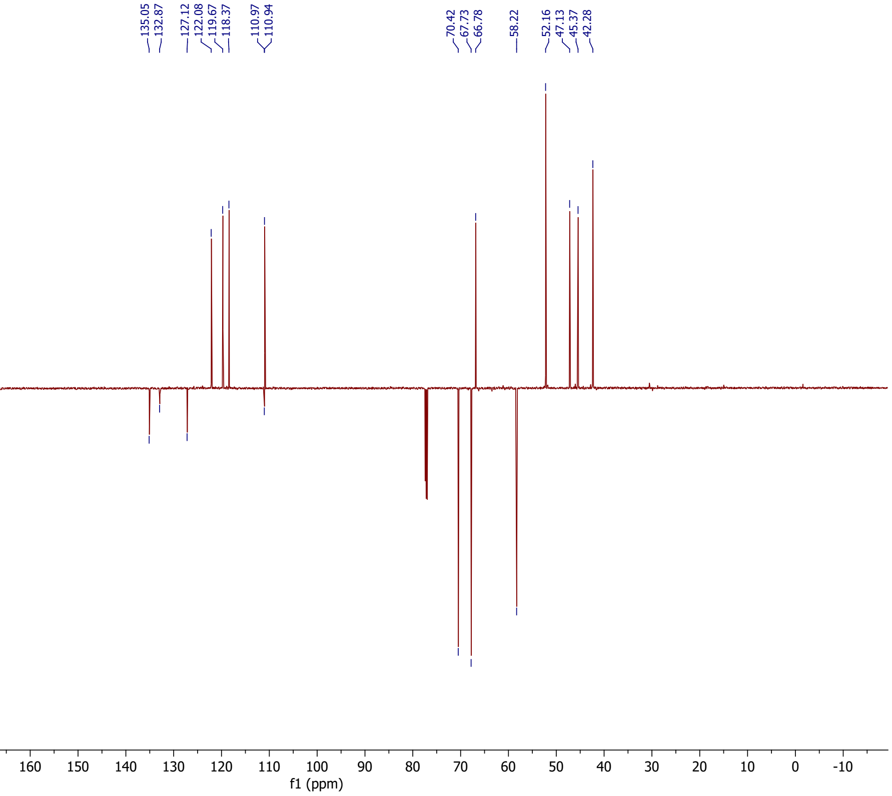

Figure 107: ***^{13}C NMR (151 MHz, CDCl3) of 5f – major diastereomer.***

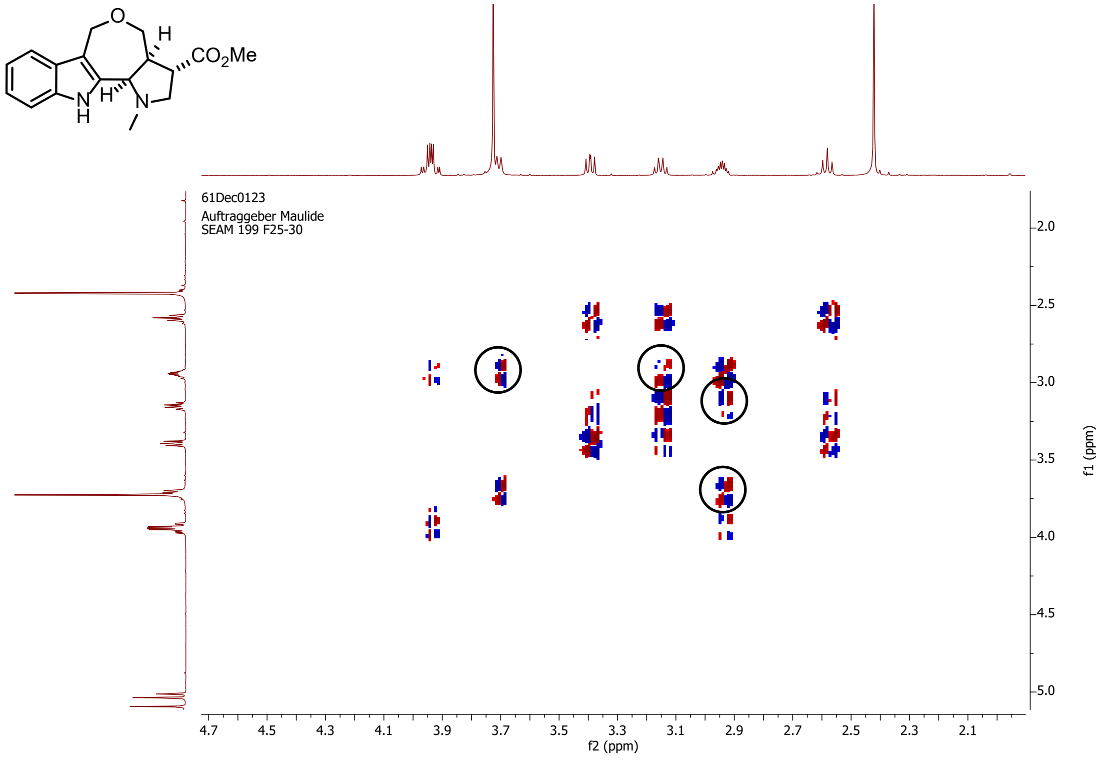

Figure 108: ***COSY (600 MHz, CDCl3) of 5f – major diastereomer.***

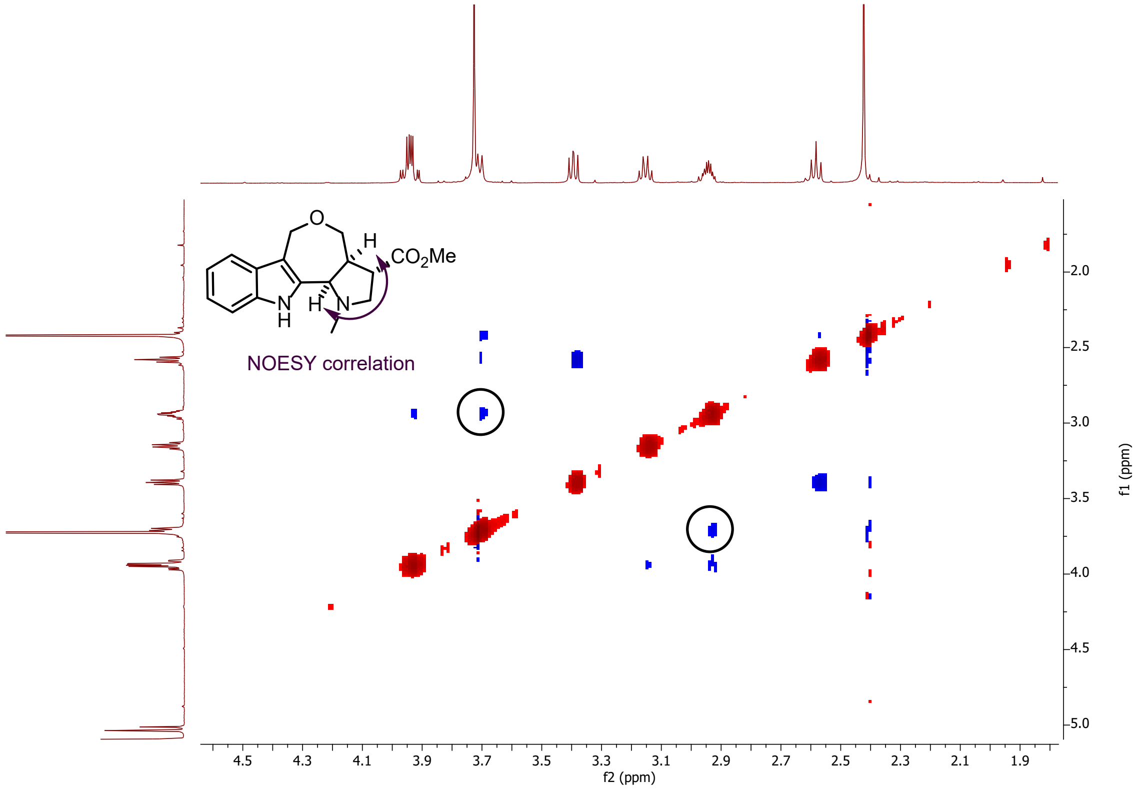

Figure 109: ***NOESY (600 MHz, CDCl3) of 5f – major diastereomer.***

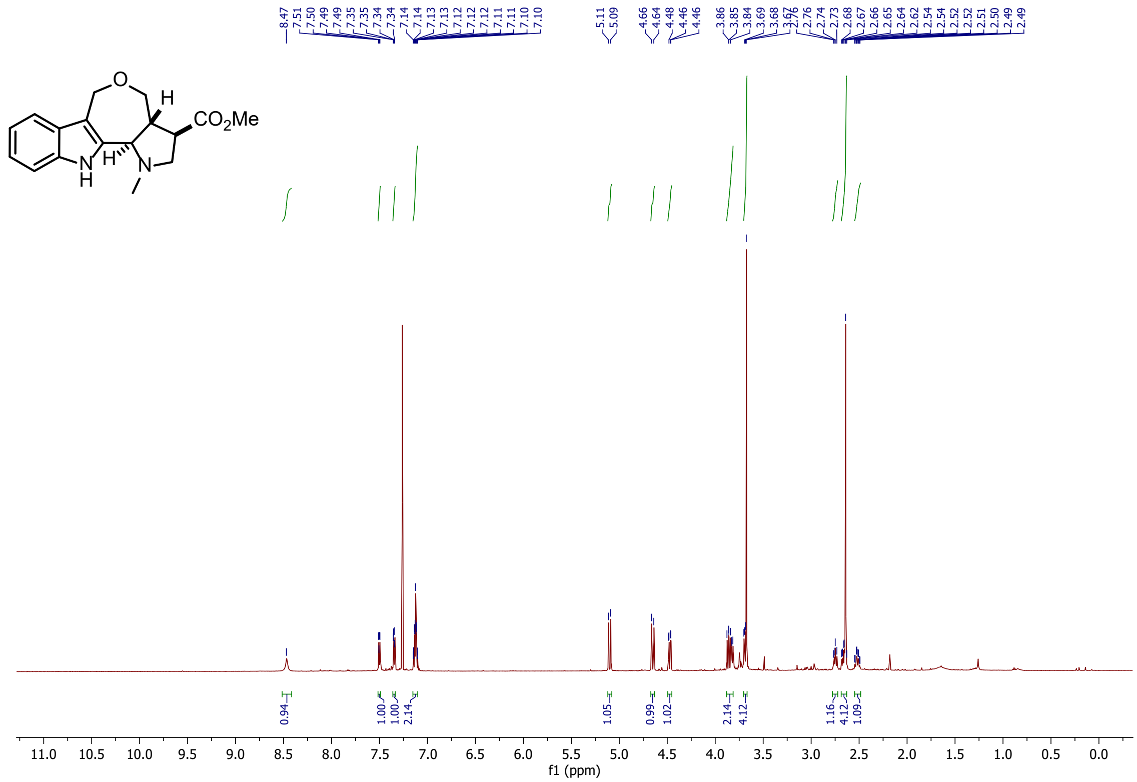

Figure 110: ***^{1}H NMR (600 MHz, CDCl3) of 5f' – minor diastereomer.***

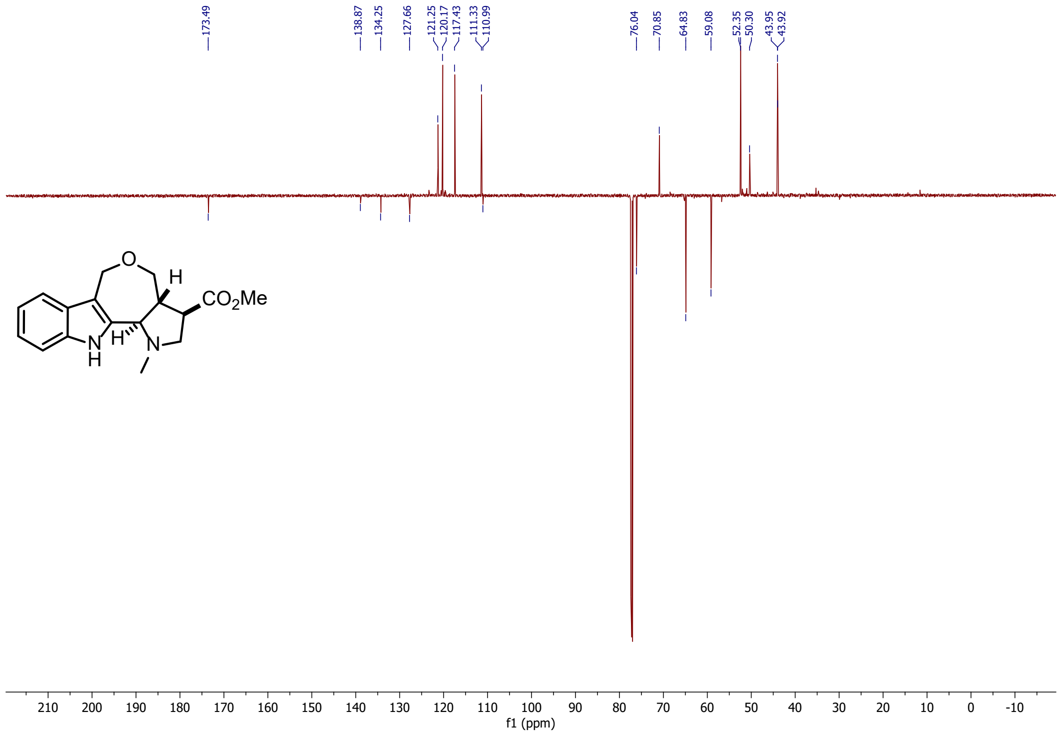

Figure 111: ***^{13}C NMR (151 MHz, CDCl3) of 5f' – minor diastereomer.***

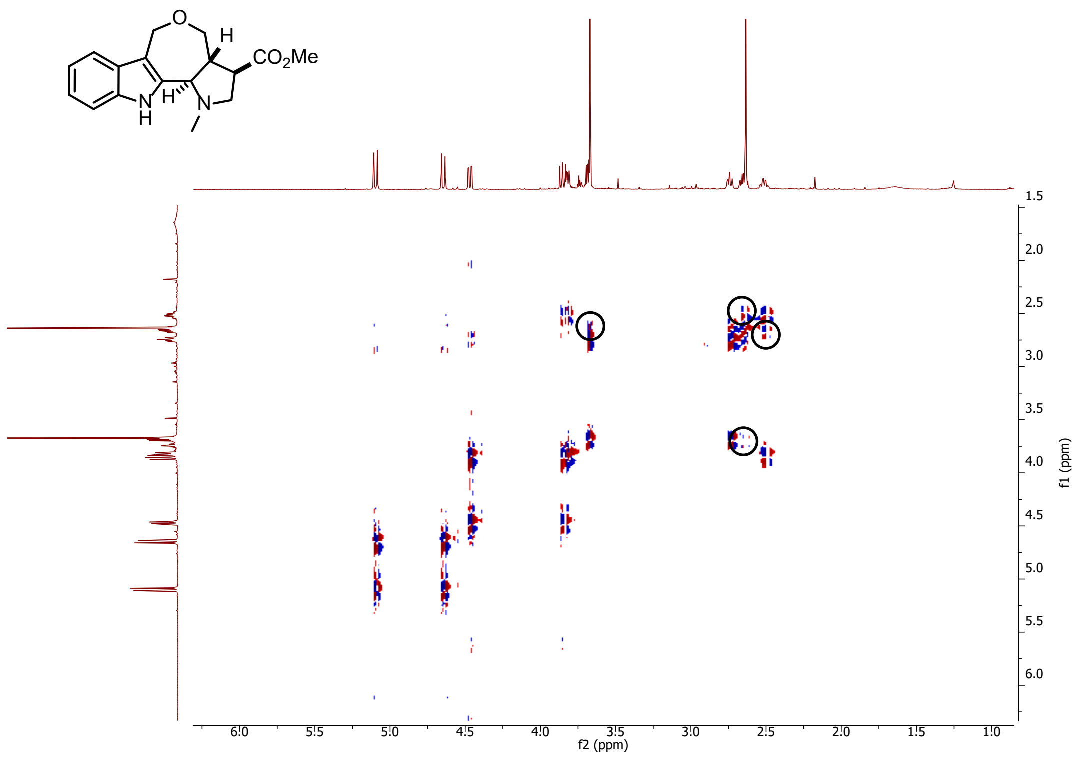

Figure 112: ***COSY (600 MHz, CDCl₃) of 5f' – minor diastereomer.***

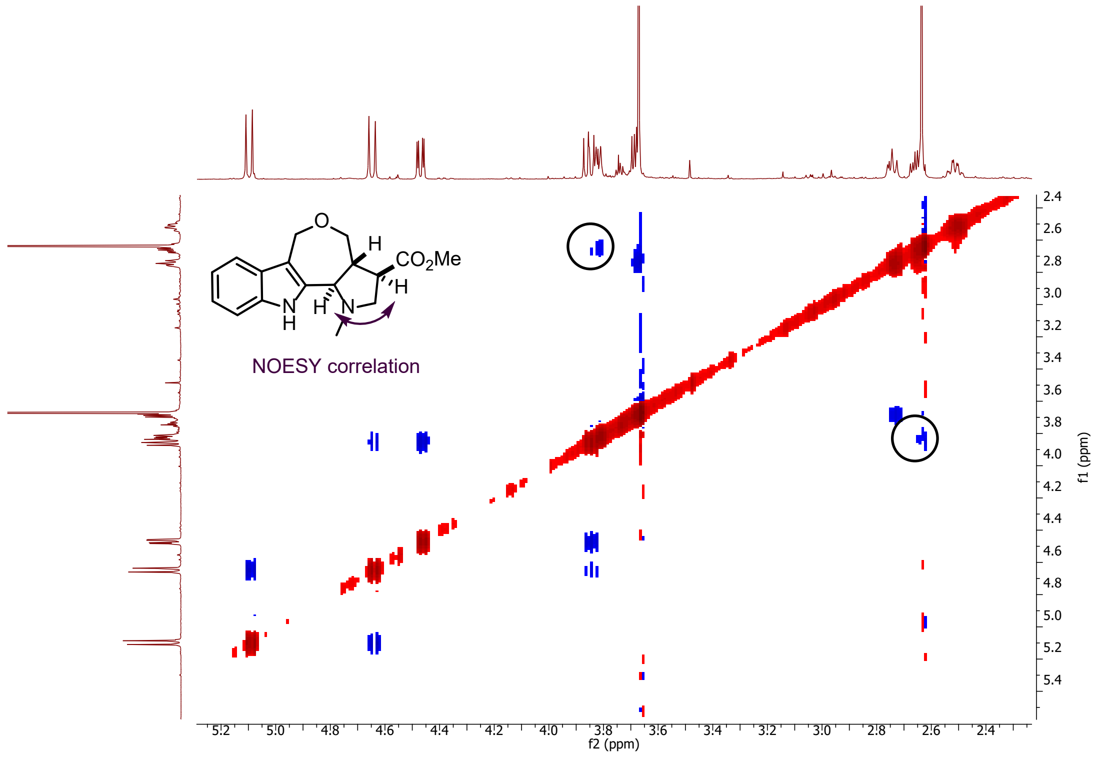

Figure 113: ***NOESY (600 MHz, CDCl₃) of 5f' – minor diastereomer.***

Methyl (3*S**,3a*R**,11b*R**)-5-tosyl-2,3,3a,4,5,6,11,11b-octahydro-1*H*-pyrrolo[2',3':5,6]azepino[4,3-b]indole-3-carboxylate (5g)

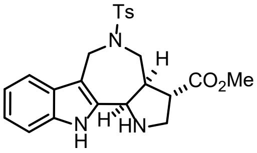

5g

Synthesized following the general procedure using **4a** (43 mg, 0.10 mmol, 1.0 eq.) and glycine **2b** (23 mg, 0.30 mmol, 3.0 eq.). Purification by flash column chromatography (gradient of CH_2Cl_2/DMA) yielded **5g** as a mixture of diastereomers (19 mg, 0.044 mmol, 44% yield, d.r. 3:1). The mixture was repurified by prep-TLC using 30% DMA in CH_2Cl_2, affording **5g** as the major diastereomer as an orange oil (11 mg, 0.025 mmol, 25% yield).

^{1}H NMR (600 MHz, $CDCl_3$) δ 8.63 (br s, 1H), 7.58 – 7.54 (m, 1H), 7.46 (d, J = 8.0 Hz, 2H), 7.29 – 7.27 (m, 1H), 7.17 – 7.12 (m, 2H), 7.02 (d, J = 8.0 Hz, 2H), 4.70 (d, J = 16.2 Hz, 1H), 4.63 (d, J = 16.2 Hz, 1H), 4.09 (dd, J = 13.2, 4.0 Hz, 1H), 3.91 (d, J = 10.4 Hz, 1H), 3.72 (s, 3H), 3.46 – 3.40 (m, 1H), 3.38 – 3.27 (m, 2H), 2.73 (td, J = 9.5, 6.2 Hz, 1H), 2.30 (s, 3H), 2.29 – 2.23 (m, 1H).

^{13}C NMR (151 MHz, $CDCl_3$) δ 173.9, 143.2, 136.7, 136.1, 134.4, 129.4 (2C), 127.2 (3C), 127.2, 121.8, 120.2, 117.8, 111.2, 108.8, 62.4, 52.5, 51.2, 49.8, 49.4, 48.7, 43.0, 21.6.

HRMS (ESI-TOF) m/z: $[M + H]^+$ Calcd for $C_{23}H_{26}N_3O_4S$ 440.1639; Found 440.1639.

IR (neat) vmax: 3390, 2923, 1730, 1451, 1437, 1361, 1333, 1306, 1288, 1221, 1154, 1090, 906.

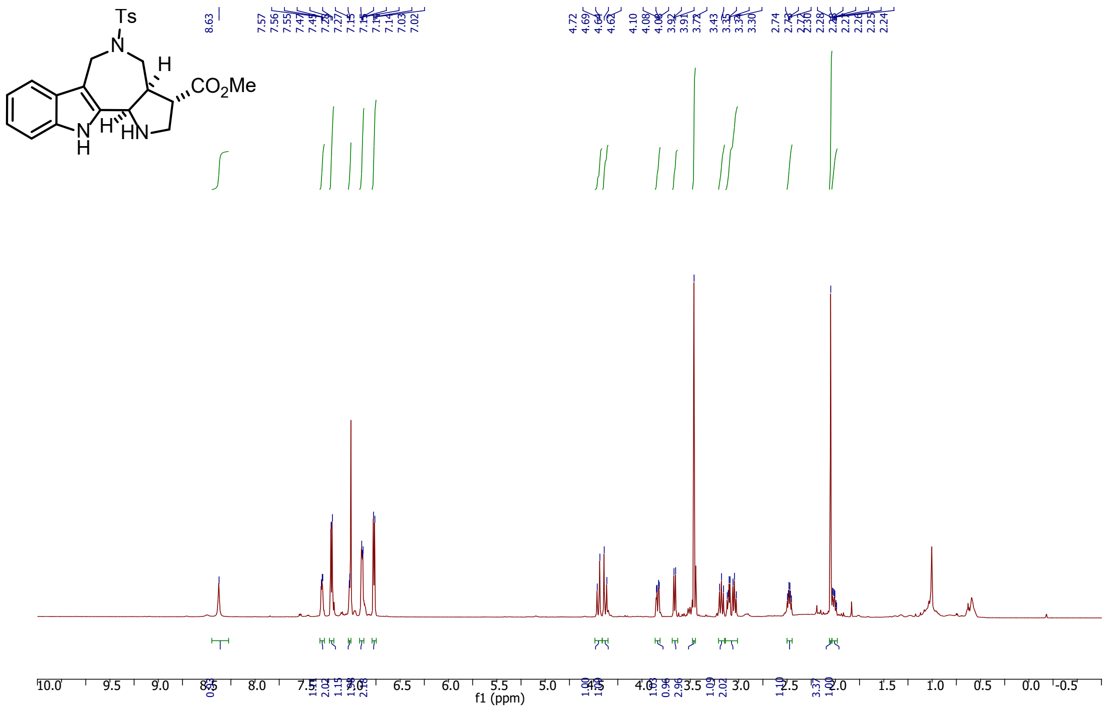

Figure 114: ***^{1}H NMR (600 MHz, CDCl3) of 5g.***

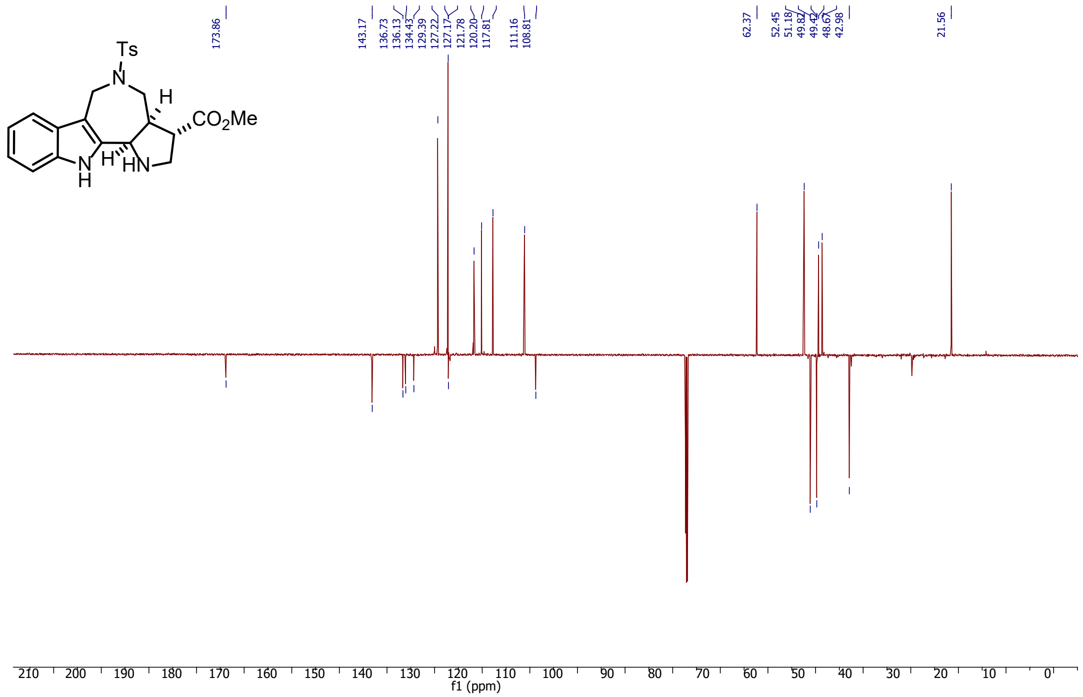

Figure 115: ***^{13}C NMR (151 MHz, CDCl3) of 5g.***

Methyl (3*S**,3a*R**,11b*R**)-1-benzyl-5-tosyl-2,3,3a,4,5,6,11,11b-octahydro-1*H*-pyrrolo[2',3':5,6]azepino[4,3-b]indole-3-carboxylate (5h)

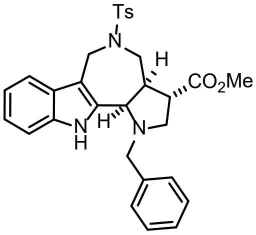

5h

Synthesized following the general procedure using **4a** (43 mg, 0.10 mmol, 1.0 eq.) and *N*-benzylglycine HCl **2c** (62 mg, 0.30 mmol, 3.0 eq.). Purification by flash column chromatography (gradient of CH_2Cl_2/DMA), followed by prep-TLC (5% DMA in CH_2Cl_2) yielded **5h** as an orange oil as a single diastereomer (26 mg, 0.049 mmol, 49% yield, d.r. >20:1).

^{1}H NMR (600 MHz, $CDCl_3$) δ 8.57 (br s, 1H), 7.78 (d, *J* = 8.3 Hz, 2H), 7.36 – 7.32 (m, 7H), 7.31 (d, *J* = 8.1 Hz, 1H), 7.28 – 7.24 (m, 1H), 7.15 (ddd, *J* = 8.1, 7.2, 1.0 Hz, 1H), 7.07 (ddd, *J* = 7.9, 7.1, 0.8 Hz, 1H), 4.76 (d, *J* = 14.0 Hz, 1H), 4.47 (d, *J* = 14.0 Hz, 1H), 4.34 (d, *J* = 8.3 Hz, 1H), 3.96 (d, *J* = 13.0 Hz, 1H), 3.71 (s, 3H), 3.69 – 3.63 (m, 2H), 3.36 (dd, *J* = 9.9, 7.6 Hz, 1H), 3.24 – 3.13 (m, 2H), 2.88 (dd, *J* = 17.0, 8.9 Hz, 1H), 2.77 (t, *J* = 9.9 Hz, 1H), 2.43 (s, 3H).

^{13}C NMR (151 MHz, $CDCl_3$) δ 173.6, 143.6, 138.8, 135.6, 134.3, 134.1, 129.9 (2C), 128.8 (2C), 128.7 (2C), 127.8, 127.5, 127.4 (2C), 121.8, 119.8, 117.8, 111.1, 104.9, 64.0, 60.6, 56.1, 52.3, 48.2, 46.4, 46.1, 46.0, 21.7.

HRMS (ESI-TOF) m/z: $[M + H]^+$ Calcd for $C_{30}H_{32}N_3O_4S$ 530.2108; Found 530.2095.

IR (neat) νmax: 3371, 2951, 2923, 1728, 1493, 1453, 1436, 1368, 1332, 1288, 1235, 1198, 1152, 1091.

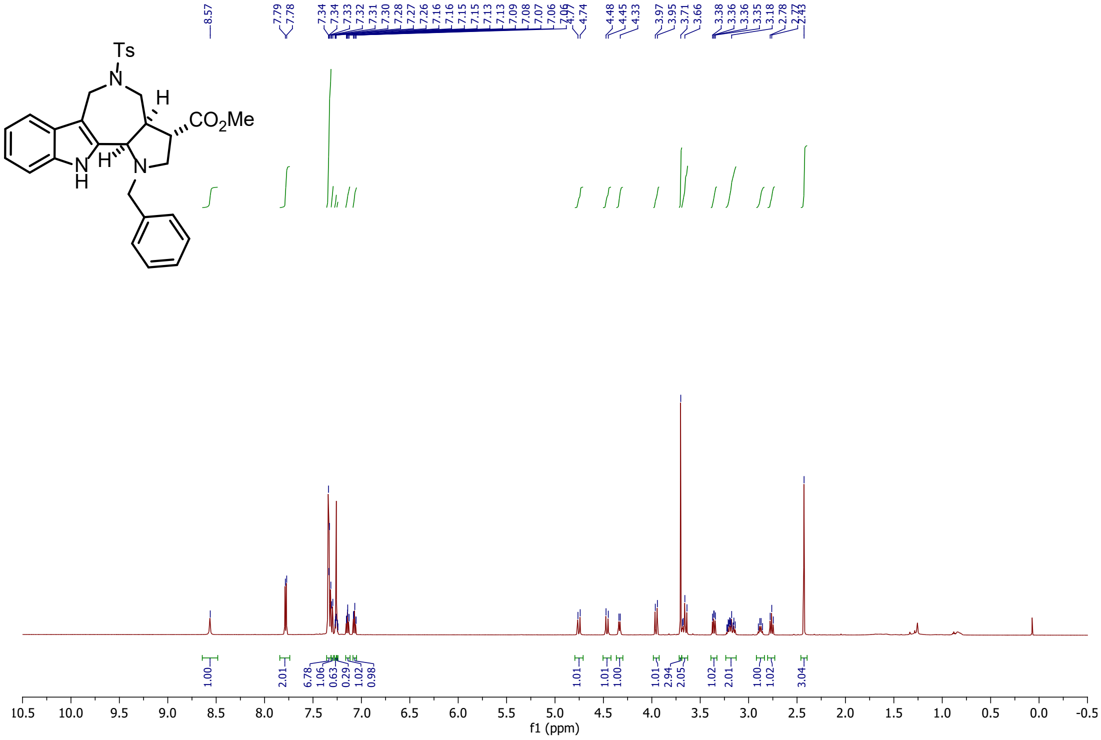

Figure 116: ^{1}H NMR (600 MHz, CDCl3) of 5h.

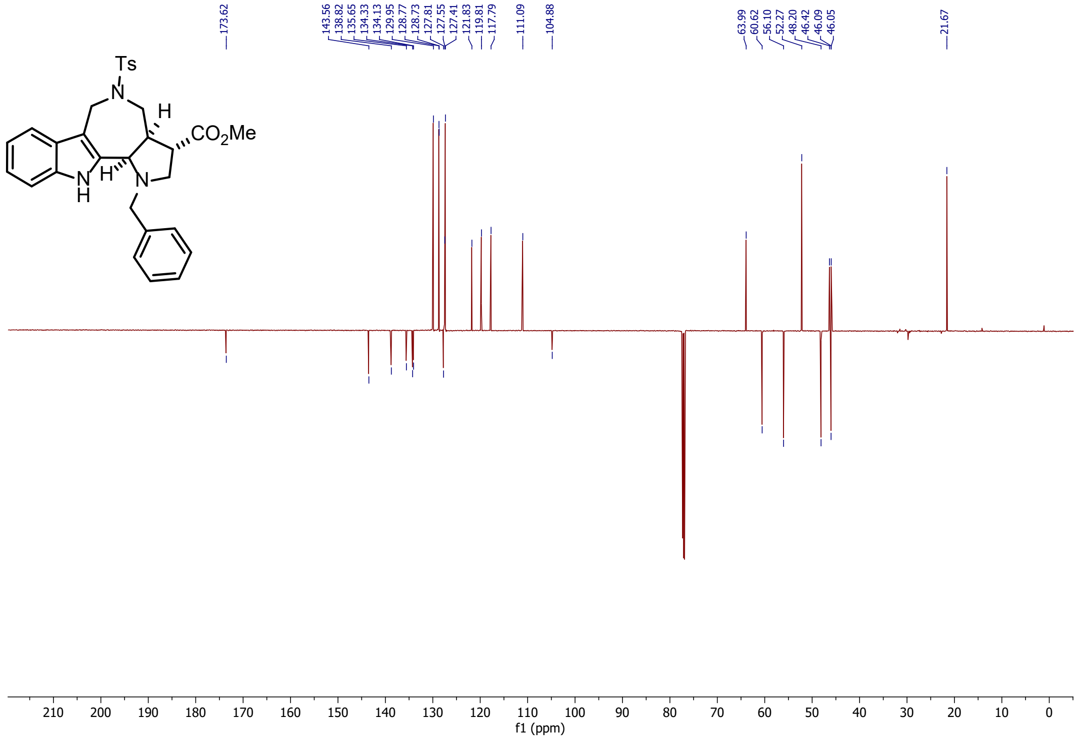

Figure 117: ^{13}C NMR (151 MHz, CDCl3) of 5h.

Methyl (3a*S**,4*S**,4a*R**,12b*R**)-6-tosyl-2,3,3a,4,4a,5,6,7,12,12b-decahydro-1*H*-pyrrolizino[3',2':5,6]azepino[4,3-b]indole-4-carboxylate (5i)

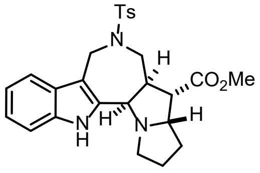

5i

Synthesized following the general procedure using **4a** (43 mg, 0.10 mmol, 1.0 eq.) and (*L*)-proline **2d** (35 mg, 0.30 mmol, 3.0 eq.). Purification by flash column chromatography (gradient of CH_2Cl_2/DMA) yielded **5i** as a pale-yellow solid as a single diastereomer (40 mg, 0.084 mmol, 84% yield, d.r. >20:1).

^{1}H NMR (600 MHz, $CDCl_3$) δ 9.00 (br s, 1H), 7.80 (d, J = 8.1 Hz, 2H), 7.38 – 7.32 (m, 4H), 7.14 (t, J = 7.5 Hz, 1H), 7.07 (t, J = 7.5 Hz, 1H), 4.78 (d, J = 14.2 Hz, 1H), 4.44 (d, J = 7.6 Hz, 1H), 4.33 (d, J = 14.2 Hz, 1H), 3.92 – 3.81 (m, 2H), 3.67 (s, 3H), 3.36 (ddd, J = 9.3, 6.9, 2.1 Hz, 1H), 3.28 – 3.18 (m, 1H), 2.95 (dd, J = 14.4, 9.1 Hz, 1H), 2.82 – 2.71 (m, 2H), 2.44 (s, 3H), 1.96 – 1.88 (m, 1H), 1.88 – 1.72 (m, 2H), 1.45 – 1.34 (m, 1H).

^{13}C NMR (151 MHz, $CDCl_3$) δ 172.6, 143.5, 135.9, 135.4, 134.1, 129.9 (2C), 127.9, 127.4 (2C), 121.4, 119.5, 117.7, 111.0, 103.7, 65.9, 65.2, 56.9, 51.9, 50.4, 48.7, 47.6, 43.3, 29.5, 26.6, 21.7.

HRMS (ESI-TOF) m/z: $[M+H]^+$ Calcd for $C_{26}H_{30}N_3O_4S$ 480.1952; Found 480.1952.

IR (neat) νmax: 3397, 2950, 1729, 1461, 1437, 1330, 1303, 1237, 1220, 1158, 1120, 1092, 1047.

Mp.: 167-169 °C.

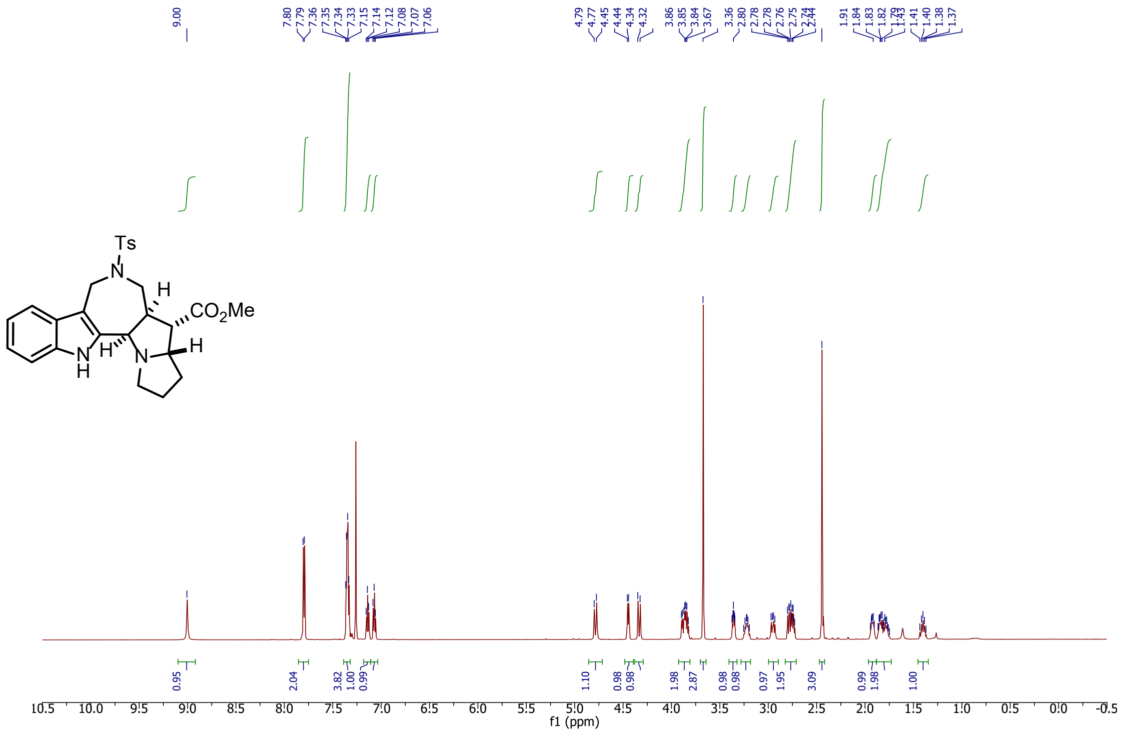

*Figure 118: **^{1}H NMR (600 MHz, CDCl₃) of 5i.***

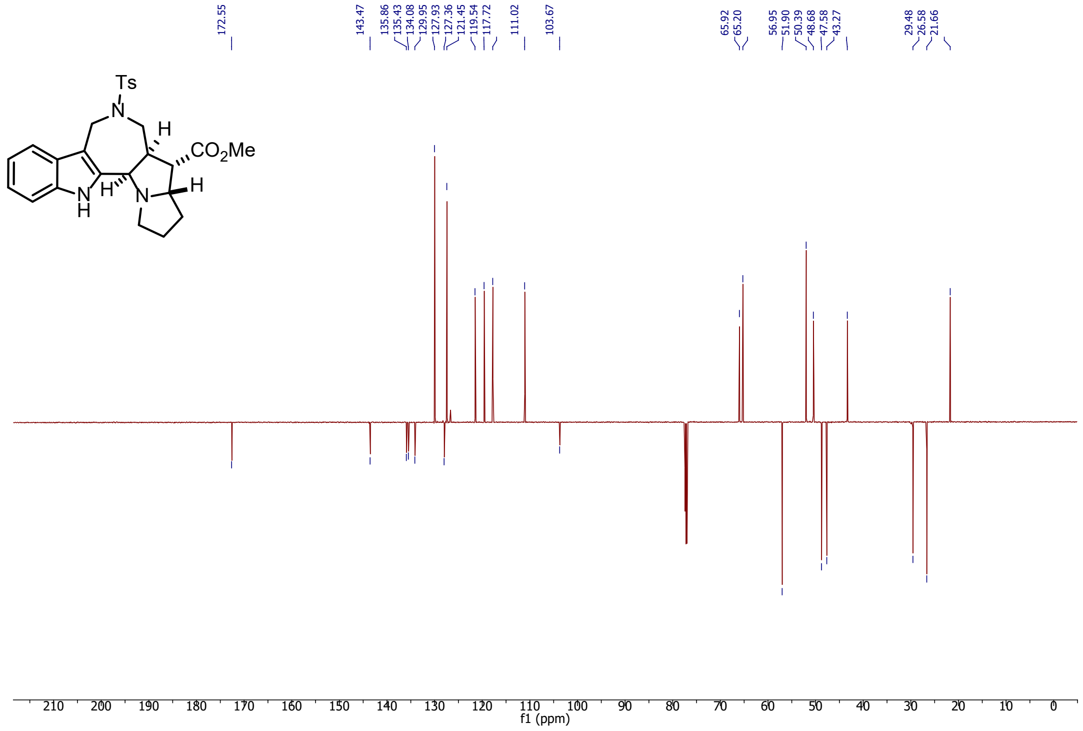

*Figure 119: **^{13}C NMR (151 MHz, CDCl₃) of 5i.***

Methyl (7a*R**,8*S**,8a*S**,13a*R**)-6-tosyl-5,6,7,7a,8,8a,9,10,11,12,13a,14-dodecahydroindolizino[3',2':5,6]azepino[4,3-b]indole-8-carboxylate (5j)

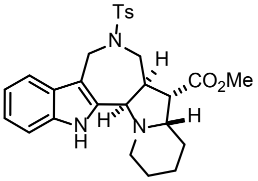

5j

Synthesized following the general procedure using **4a** (43 mg, 0.10 mmol, 1.0 eq.) and pipecolic acid **2e** (39 mg, 0.30 mmol, 3.0 eq.). Purification by flash column chromatography (gradient of CH_2Cl_2/DMA) yielded **5j** as an orange solid as a single diastereomer (41 mg, 0.084 mmol, 84% yield, d.r. >20:1).

^{1}H NMR (700 MHz, $CDCl_3$) δ 8.48 (br s, 1H), 7.79 (d, *J* = 8.3 Hz, 2H), 7.36 – 7.30 (m, 4H), 7.14 (ddd, *J* = 8.1, 7.1, 1.1 Hz, 1H), 7.07 (ddd, *J* = 8.0, 7.1, 0.9 Hz, 1H), 4.72 (d, *J* = 13.9 Hz, 1H), 4.56 (d, *J* = 9.7 Hz, 1H), 4.51 (d, *J* = 13.9 Hz, 1H), 3.71 (s, 3H), 3.63 (dd, *J* = 14.2, 4.2 Hz, 1H), 3.47 (ddd, *J* = 10.9, 6.9, 3.6 Hz, 1H), 3.41 (qd, *J* = 9.7, 4.3 Hz, 1H), 3.01 (d, *J* = 13.7 Hz, 1H), 2.97 – 2.88 (m, 3H), 2.42 (s, 3H), 1.86 – 1.80 (m, 1H), 1.55 – 1.47 (m, 1H), 1.46 – 1.34 (m, 3H), 1.29 (ddd, *J* = 24.6, 13.1, 3.8 Hz, 1H).

^{13}C NMR (176 MHz, $CDCl_3$) δ 172.2, 143.4, 135.8, 135.6, 134.4, 129.9 (2C), 128.0, 127.5 (2C), 121.7, 119.8, 117.7, 111.1, 103.7, 60.7, 59.1, 52.0, 50.0, 48.5, 47.3, 44.9, 43.4, 25.0, 23.4, 21.6, 21.2.

HRMS (ESI-TOF) m/z: $[M+H]^+$ Calcd for $C_{27}H_{32}N_3O_4S$ 494.2108; Found 494.2100.

IR (neat) νmax: 3369, 2931, 2855, 1733, 1458, 1333, 1231, 1159, 1094, 910.

Mp.: 103-104 °C.

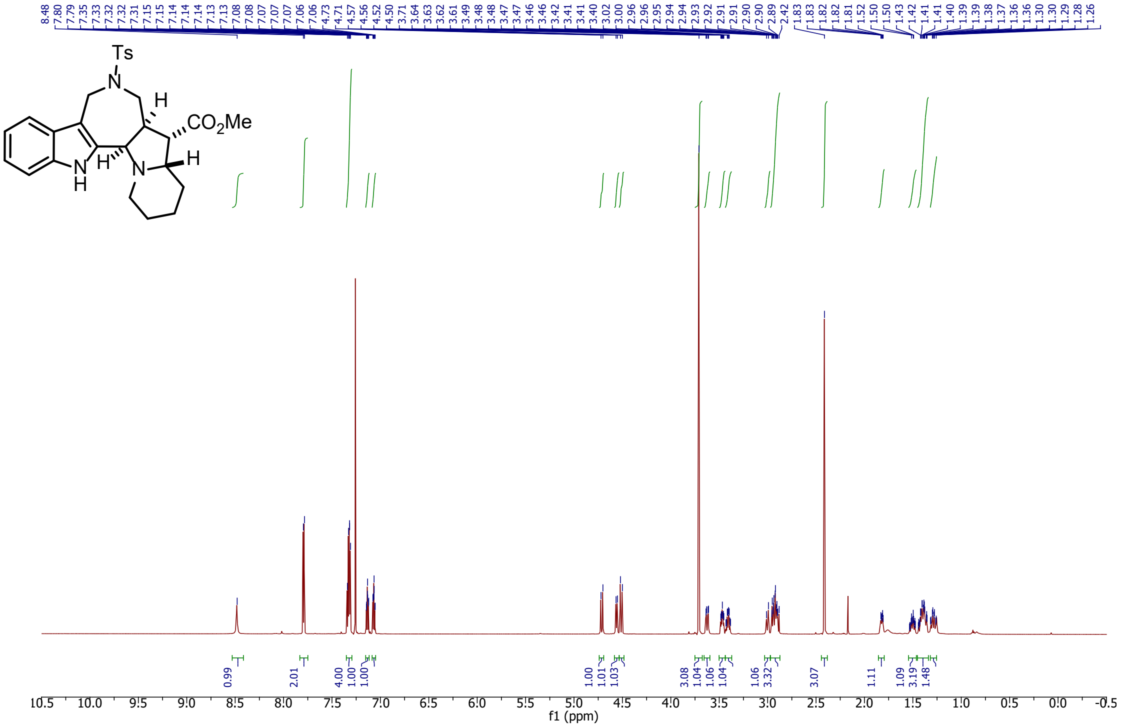

Figure 120: ^{1}H NMR (700 MHz, CDCl₃) of 5j.

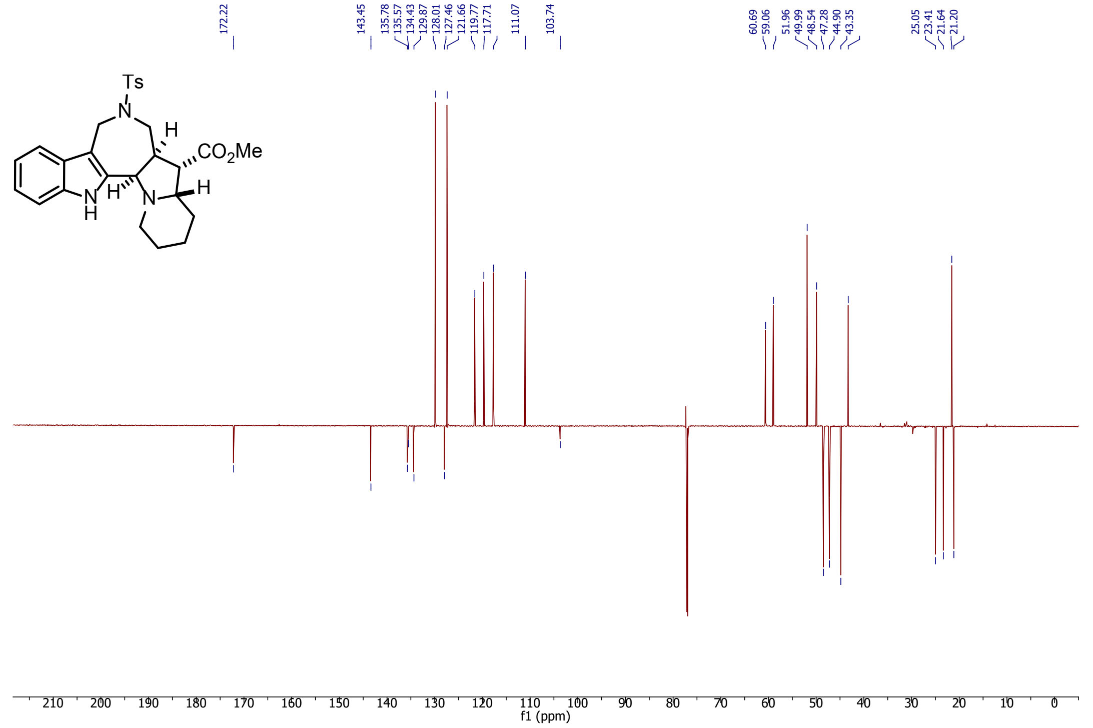

Figure 121: ^{13}C NMR (176 MHz, CDCl₃) of 5j.

Methyl (2a*S**,3*S**,3a*R**,11b*R**)-5-tosyl-1,2,2a,3,3a,4,5,6,11,11b-decahydroazeto[1'',2'':1',5']pyrrolo[2',3':5,6]azepino[4,3-b]indole-3-carboxylate (5k)

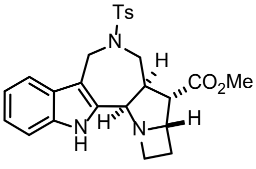

5k

Synthesized following the general procedure using **4a** (43 mg, 0.10 mmol, 1.0 eq.) and azetidine-2-carboxylic acid **2f** (30 mg, 0.30 mmol, 3.0 eq.). Purification by flash column chromatography (gradient of CH_2Cl_2/DMA) gave **5k** as a white solid as a single diastereomer (30 mg, 0.030 mmol, 64% yield, d.r. >20:1).

^{1}H NMR (700 MHz, CDCl$_3$) δ 8.85 (br s, 1H), 7.81 (d, *J* = 8.2 Hz, 2H), 7.38 – 7.34 (m, 3H), 7.30 (d, *J* = 8.1 Hz, 1H), 7.13 (ddd, *J* = 8.1, 7.0, 0.9 Hz, 1H), 7.06 (ddd, *J* = 7.9, 7.2, 0.9 Hz, 1H), 4.62 (d, *J* = 14.2 Hz, 1H), 4.47 (d, *J* = 14.3 Hz, 1H), 4.41 – 4.35 (m, 2H), 3.87 (dd, *J* = 14.6, 5.6 Hz, 1H), 3.84 (td, *J* = 9.4, 5.9 Hz, 1H), 3.79 – 3.73 (m, 1H), 3.68 (s, 3H), 3.29 (td, *J* = 10.0, 7.7 Hz, 1H), 3.25 (dd, *J* = 14.6, 8.2 Hz, 1H), 2.89 (dd, *J* = 11.8, 8.6 Hz, 1H), 2.45 (s, 3H), 2.49 – 2.42 (m, 1H), 2.07 – 2.01 (m, 1H).

^{13}C NMR (176 MHz, CDCl$_3$) δ 172.0, 143.5, 135.9, 134.2, 134.1, 130.0 (2C), 127.5, 127.4 (2C), 121.7, 119.6, 117.8, 111.0, 104.6, 66.7, 66.6, 52.2, 52.1, 51.2, 48.9, 47.6, 44.3, 21.7, 20.6.

HRMS (ESI-TOF) m/z: $[M+H]^+$ Calcd for $C_{25}H_{28}N_3O_4S$ 466.1795; Found 466.1787.

IR (neat) νmax: 3407, 2951, 1729, 1489, 1461, 1333, 1222, 1159, 1093, 909.

Mp.: 210-212 °C.

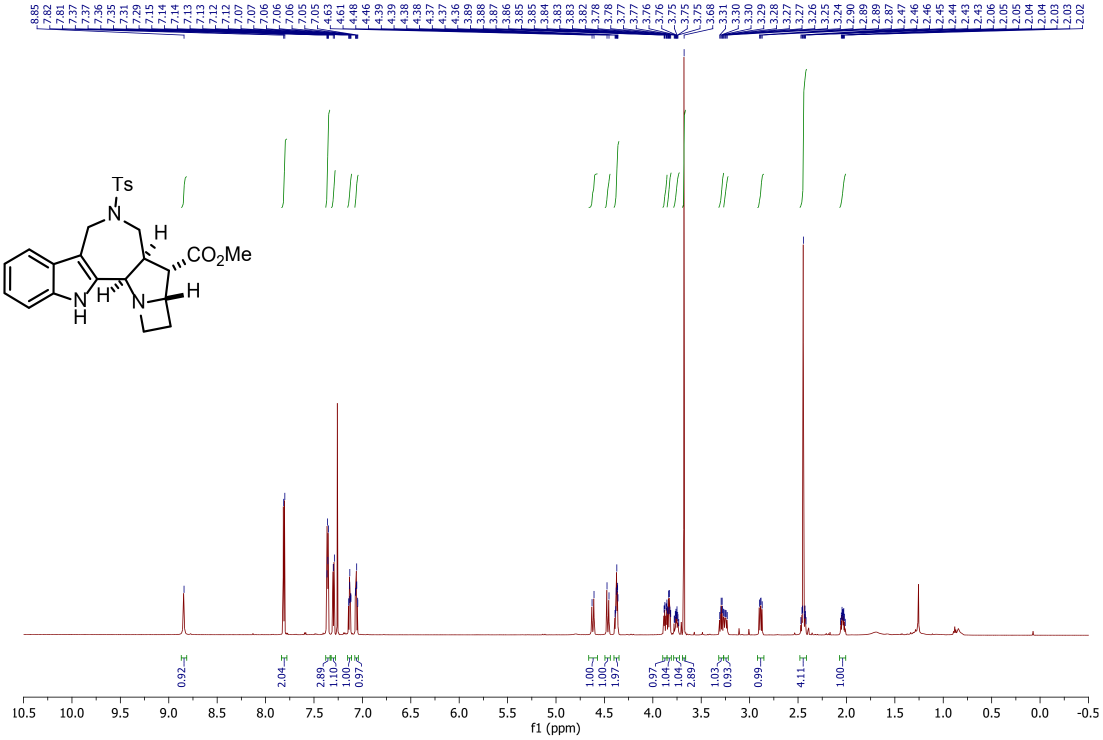

*Figure 122: **^{1}H NMR (700 MHz, CDCl3) of 5k.***

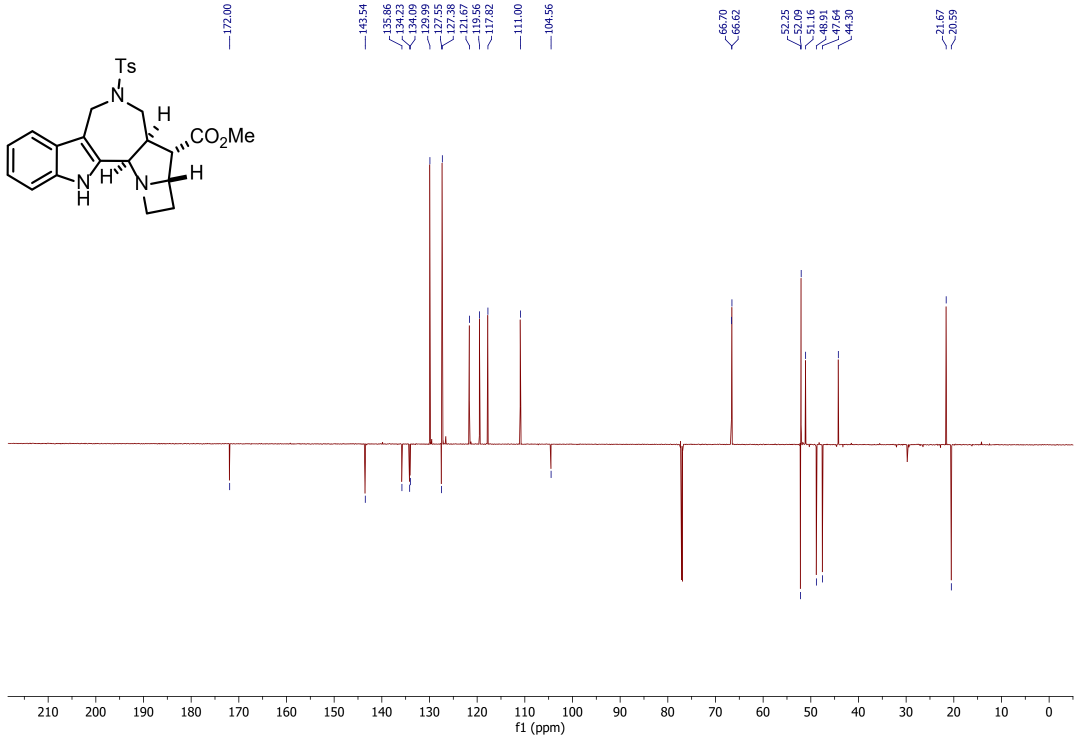

*Figure 123: **^{13}C NMR (176 MHz, CDCl3) of 5k.***

Methyl (3*S**,3a*R**,11b*R**)-1-methyl-5-((2,4,6-tris(trifluoromethyl)phenyl)sulfonyl)-2,3,3a,4,5,6,11,11b-octahydro-1*H*-pyrrolo[2',3':5,6]azepino[4,3-b]indole-3-carboxylate (5l)

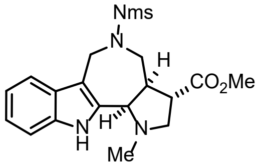

5l

Synthesized following the general procedure using **4g** (62 mg, 0.10 mmol, 1.0 eq.) and sarcosine **2a** (27 mg, 0.30 mmol, 3.0 eq.). Purification by flash column chromatography (gradient of CH_2Cl_2/DMA) gave **5l** as a pale-yellow solid as the major diastereomer (32 mg, 0.049 mmol, 49% yield, d.r. 16:1). The minor diastereomer was not isolated.

^{1}H NMR (700 MHz, $CDCl_3$) 8.62 (br s, 1H), 8.32 (s, 2H), 7.36 (dd, J = 7.8, 4.8 Hz, 2H), 7.17 (ddd, J = 8.0, 7.1, 1.0 Hz, 1H), 7.11 (ddd, J = 8.0, 7.2, 0.8 Hz, 1H), 4.69 (d, J = 14.4 Hz, 1H), 4.57 (d, J = 14.4 Hz, 1H), 4.12 (d, J = 8.7 Hz, 1H), 3.71 – 3.66 (m, 4H), 3.48 – 3.41 (m, 1H), 3.28 (dd, J = 14.7, 9.6 Hz, 1H), 3.22 (qd, J = 9.0, 4.2 Hz, 1H), 2.78 – 2.68 (m, 2H), 2.53 (s, 3H).

^{13}C NMR (176 MHz, $CDCl_3$) δ 172.9, 145.0, 136.2, 135.5, 134.4, 134.3 (q, J_F = 35.1 Hz), 133.2 (2C, q, J_F = 33.9 Hz), 129.5, 127.8, 122.2 (q, J_F = 275.7 Hz), 122.1 (q, J_F = 273.4 Hz), 121.9, 120.1 (2C), 117.7, 111.2, 104.0, 64.8, 58.3, 52.3, 49.0, 46.7, 46.0, 44.7, 42.9.

^{19}F NMR (659 MHz, $CDCl_3$) δ −55.5 (6F), −63.5 (3F).

HRMS (ESI-TOF) m/z: $[M + H]^+$ Calcd for $C_{26}H_{23}F_9N_3O_4S$ 644.1260; Found 644.1252.

IR (neat) νmax: 2956, 2923, 2852, 1726, 1460, 1439, 1370, 1336, 1274, 1193, 1173, 1155, 1133.

Mp.: 175-177 °C.

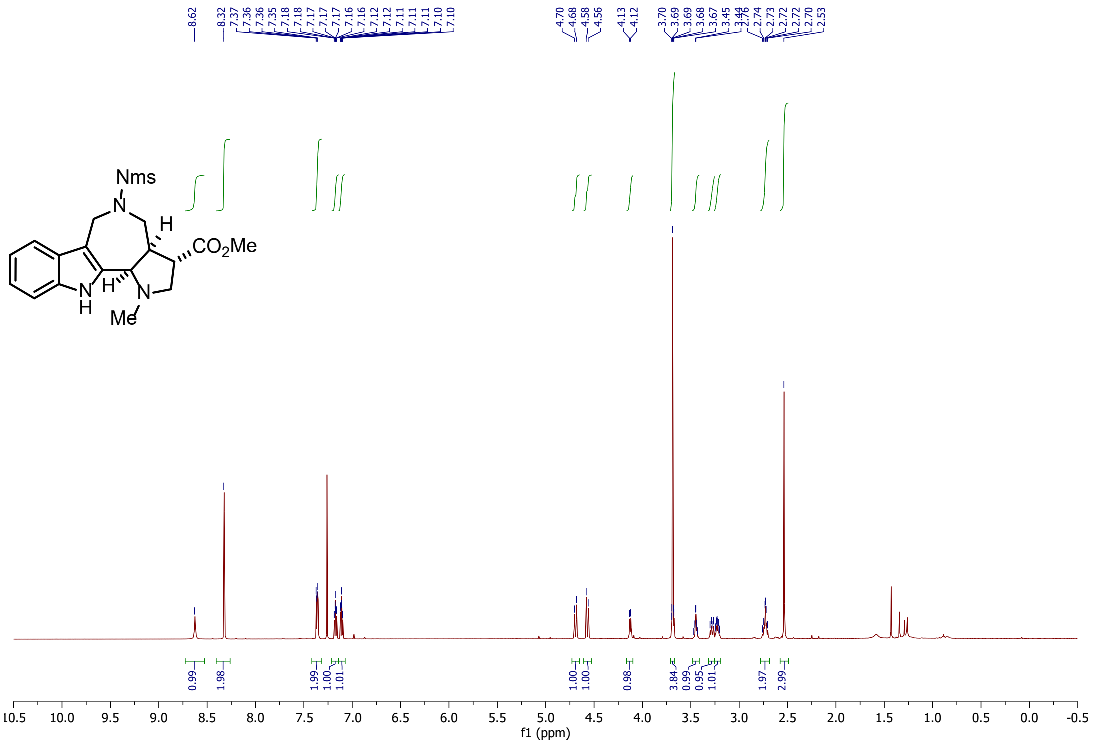

Figure 124: ***^{1}H NMR (700 MHz, $CDCl_3$) of 5l.***

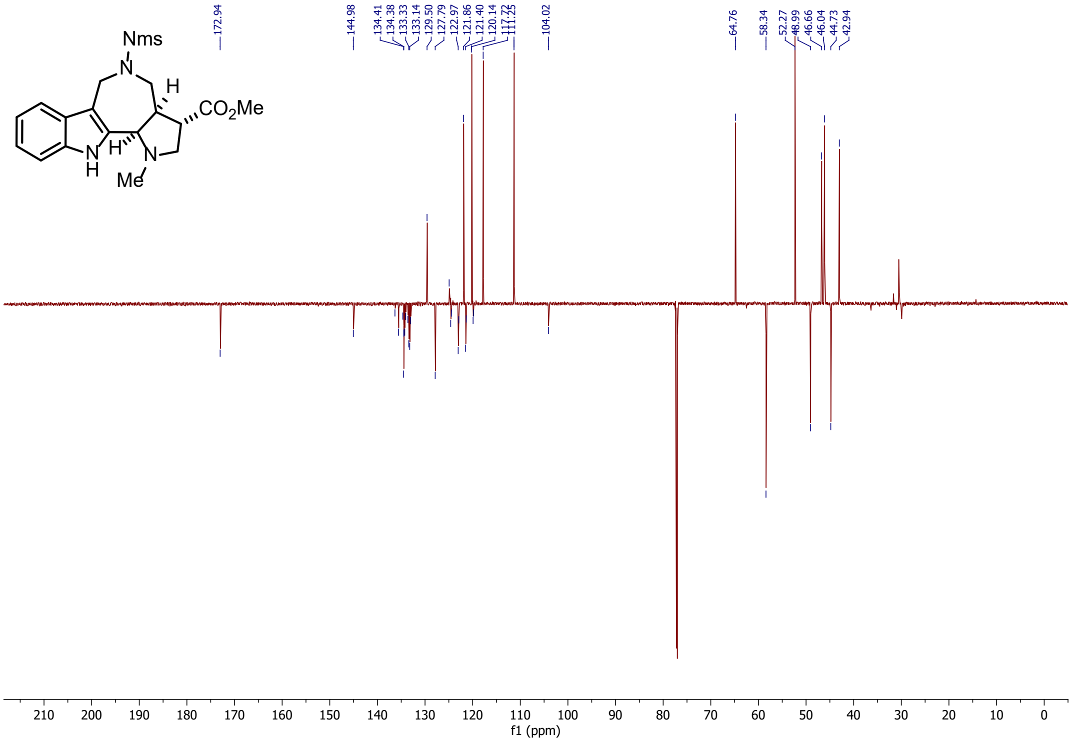

Figure 125: ***^{13}C NMR (176 MHz, $CDCl_3$) of 5l.***

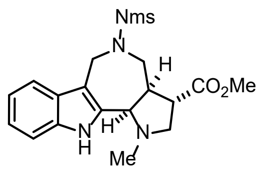

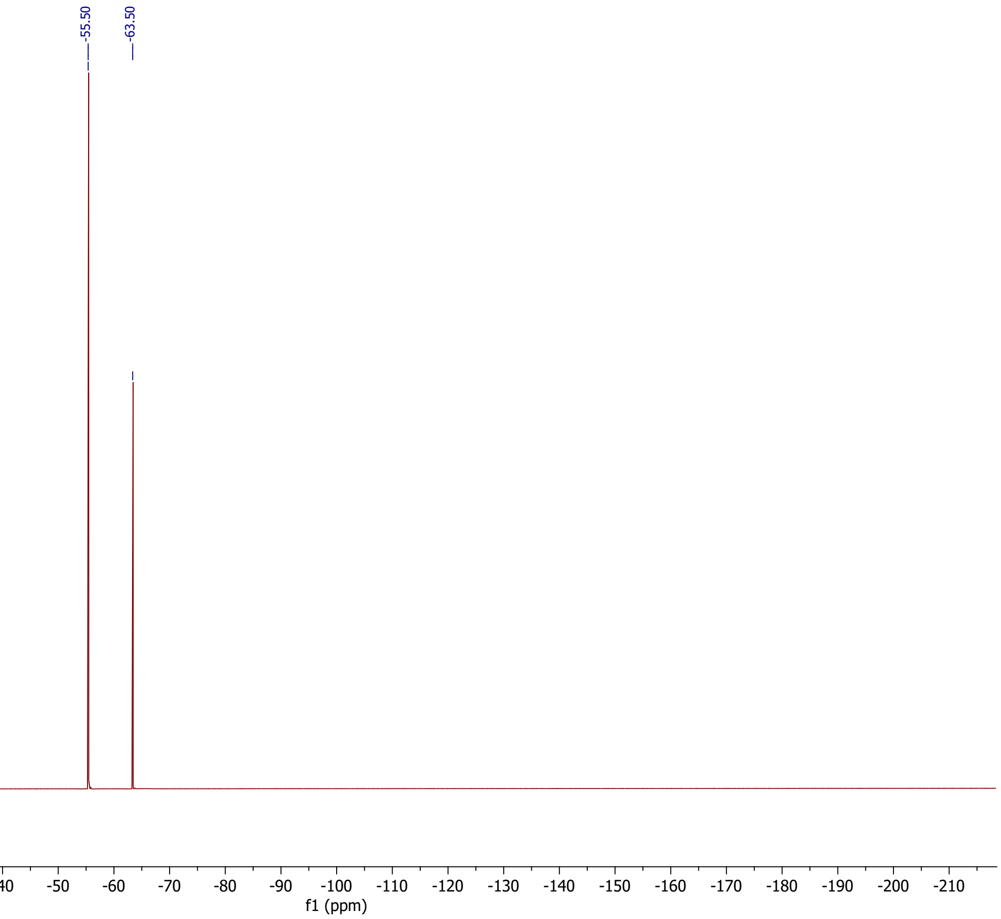

Figure 126: ***^{19}F NMR (659 MHz, CDCl3) of 5l.***

5-(tert-butyl) 3-methyl (3*S**,3a*R**,11b*R**)-1-methyl-1,2,3,3a,4,6,11,11b-octahydro-5*H*-pyrrolo[2',3':5,6]azepino[4,3-b]indole-3,5-dicarboxylate (5m)

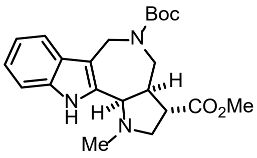

5m

Synthesized following the general procedure using **4h** (37 mg, 0.10 mmol, 1.0 eq.) and sarcosine **2a** (27 mg, 0.30 mmol, 3.0 eq.). Purification by flash column chromatography (gradient of heptanes/EtOAc) yielded **5m** as a mixture of diastereomers and yellow oil (28 mg, 0.070 mmol, 70% yield, d.r. 1.5:1).

^{1}H NMR (600 MHz, $CDCl_3$) δ 8.57 (s, 0.4H, A), 8.53 (s, 0.6H, B), 7.51-7.45 (m, 1H, A+B), 7.38-7.31 (m, 1H, A+B), 7.20-7.14 (m, 1H, A+B), 7.14-7.04 (m, A+B), 5.00-4.88 (m, A+B), 4.62-4.51 (m, 1H, A+B), 3.89 (app. d, *J* = 8.7 Hz, 1H, A+B), 3.76 – 3.65 (m, 4H, A+B), 3.40 (app. t, *J* = 8.2 Hz, 1H, A+B), 3.36 – 3.27 (m, 1H, A+B), 3-20-3.12 (m, 1H, A+B), 2.85 (app. dd, *J* = 16.8, 8.7 Hz, 1H, A+B), 2.70 (app. dd, *J* = 20.9, 10.8 Hz, 1H, A+B), 2.52-2.46 (m, 3H, A+B), 1.52 (s, 5.4H, B), 1.49 (s, 3.6H, A).

^{13}C NMR (151 MHz, $CDCl_3$) δ 173.8 (0.4C, A), 173.7 (0.6C, B), 155.7 (0.4C, A), 155.5 (0.6C, B), 134.5 (0.6C, B), 133.5 (0.4C, A), 128.4 (0.6C, B), 128.0 (0.4C, A), 121.7 (1C, A+B), 119.7 (0.6C, B), 119.6 (0.4C, A), 118.2 (0.6C, B), 118.0 (0.4C, A), 111.0 (0.4C, A), 110.9 (0.6C, B), 107.5 (0.4C, A), 106.9 (0.6C, B), 80.0 (1C, A+B), 65.6 (0.4C, A), 65.5 (0.6 C, B), 58.8 (0.6C, B), 58.6 (0.4C, A), 52.2 (0.4C, A), 52.1 (0.6C, B), 47.1 (0.4C, A), 46.6 (0.4C, A), 46.5 (0.6C, B), 46.1 (0.6C, B), 45.8 (0.6C, B), 45.3 (0.4C, A), 44.4 (0.4C, A), 43.4 (0.6C, B), 43.0 (0.4C, A), 42.9 (0.6C, B), 28.6 (1.8C, B), 28.5 (1.2C, A) ppm. *One quaternary sp^2 carbon could not be found under these conditions.*

HRMS (ESI-TOF) m/z: $[M + H]^+$ Calcd for $C_{22}H_{30}N_3O_4$ 400.2232; Found 400.2218.

IR (neat) νmax: 3307, 2976, 2930, 2850, 2783, 2249, 1730, 1669, 1166, 906, 871, 726, 667.

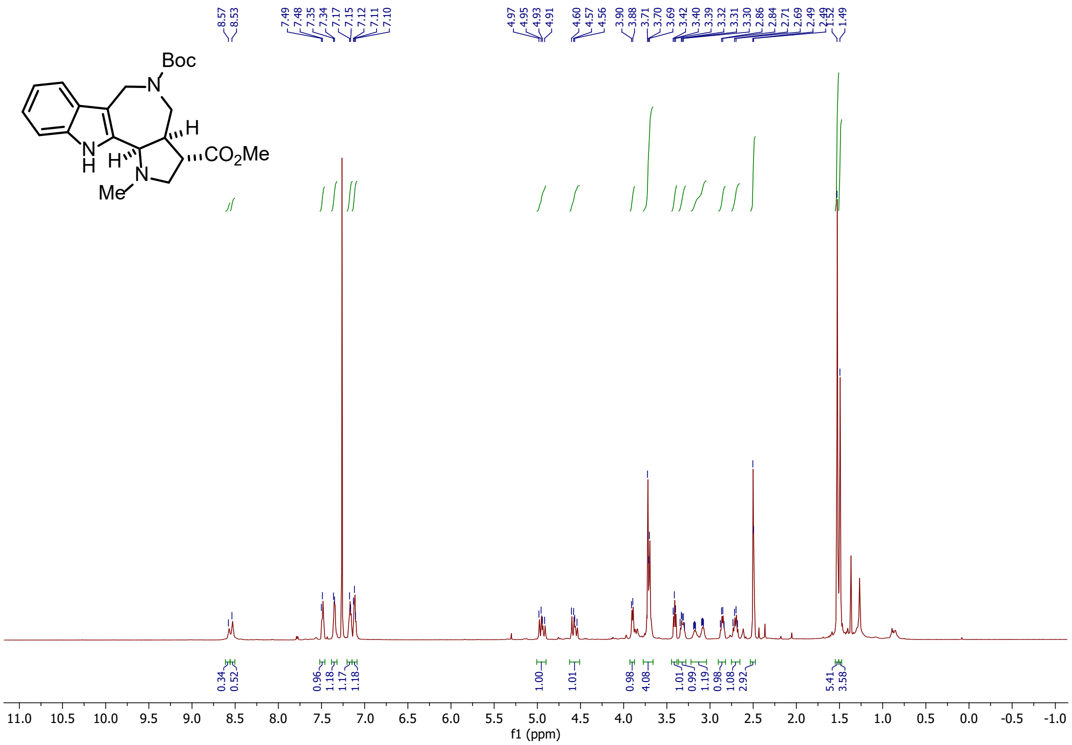

Figure 127: ***^{1}H NMR (600 MHz, CDCl₃) of 5m.***

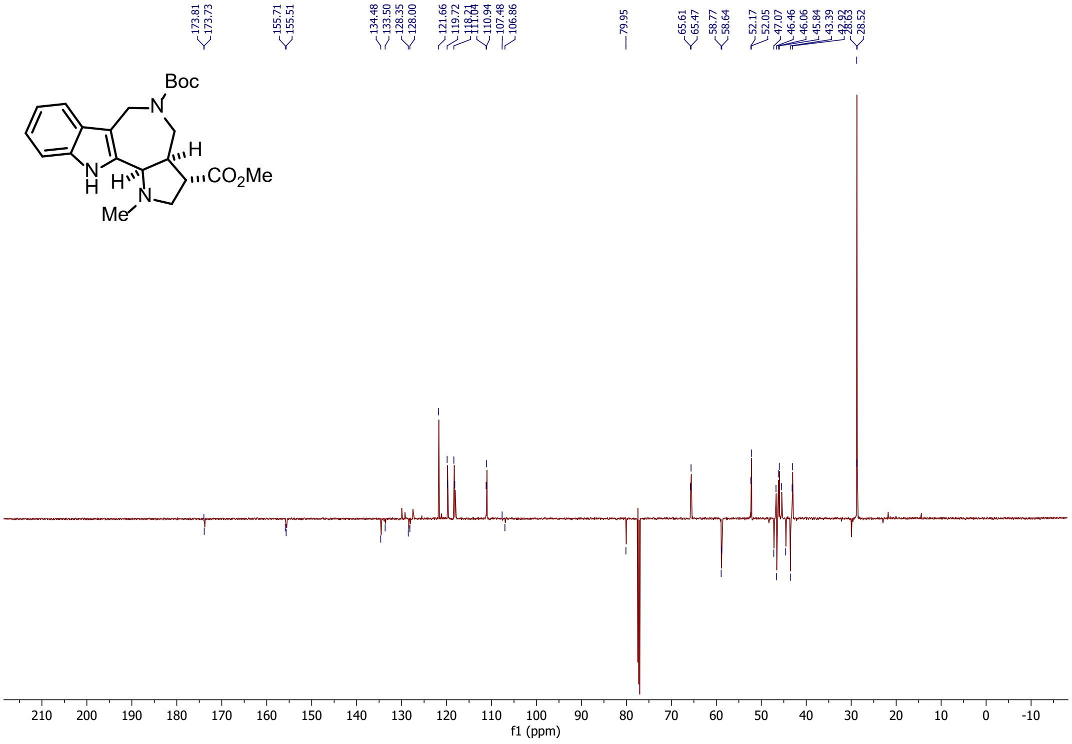

Figure 128: ***^{13}C NMR (151 MHz, CDCl₃) of 5m.***

(3a*R**,11b*R**)-1-benzyl-5-tosyl-1,3,3a,4,5,6,11,11b-octahydroisoxazolo[3',4':5,6]azepino[4,3-*b*]indole (7b)

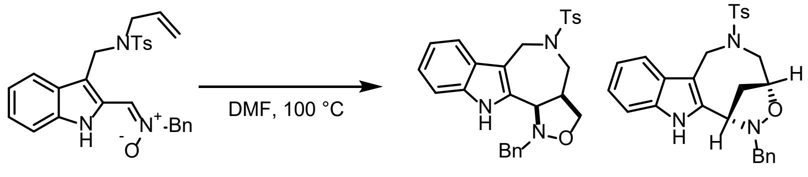

6b **7b** **8b**

A Schlenk flask was charged with **6b** (47 mg, 0.10 mmol, 1.0 eq) and DMF (1.8 mL). The mixture was stirred for 115 h at 100 °C, before cooling to ambient temperature. The work-up was performed by diluting with CH_2Cl_2 (10 mL) and addition of a brine/H_2O mixture (1:1, 10 mL). After separation of phases, the aqueous layer was washed with additional CH_2Cl_2 (5 mL) four times. The combined organic layers were dried over Na_2SO_4, filtered and concentrated under reduced pressure. Purification was performed by flash chromatography using a gradient of heptanes/EtOAc to yield **7b** as a yellow oil (7.5 mg, 0.016 mmol, 16% yield) and **8b** as an orange solid (21 mg, 0.044 mmol, 44% yield).

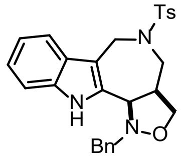

7b

^{1}H NMR (600 MHz, CDCl$_3$) δ 8.91 (br s, 1H), 7.79 (d, *J* = 8.2 Hz, 2H), 7.45 (d, *J* = 7.3 Hz, 2H), 7.38 (t, *J* = 7.6 Hz, 2H), 7.35 (d, *J* = 8.1 Hz, 2H), 7.33 – 7.29 (m, 3H), 7.14 (t, *J* = 7.3 Hz, 1H), 7.05 (t, *J* = 7.3 Hz, 1H), 4.95 (d, *J* = 14.0 Hz, 1H), 4.82 (d, *J* = 8.1 Hz, 1H), 4.30 (t, *J* = 8.7 Hz, 1H), 4.22 (dd, *J* = 13.2, 7.6 Hz, 2H), 3.95 (d, *J* = 12.6 Hz, 1H), 3.93 – 3.90 (m, 1H), 3.89 – 3.80 (m, 1H), 3.45 (t, *J* = 7.3 Hz, 1H), 2.94 (dd, *J* = 14.0, 11.1 Hz, 1H), 2.45 (s, 3H).

^{13}C NMR (151 MHz, CDCl$_3$) δ 143.9, 136.6, 135.2, 134.4, 131.9, 130.1 (2C), 129.2 (2C), 128.9 (2C), 128.1, 127.4 (2C), 127.3, 122.0, 119.6, 117.6, 111.3, 104.8, 69.1, 63.4, 60.3, 48.0, 47.5, 46.9, 21.7.

HRMS (ESI-TOF) m/z: $[M + H]^+$ Calcd for $C_{27}H_{28}N_3O_3S$ 474.1846; Found 474.1835.

IR (neat) νmax: 3416, 3060, 3030, 2924, 2853, 1597, 1494, 1461, 1375, 1335, 1305, 1236, 1184, 1159, 1120.

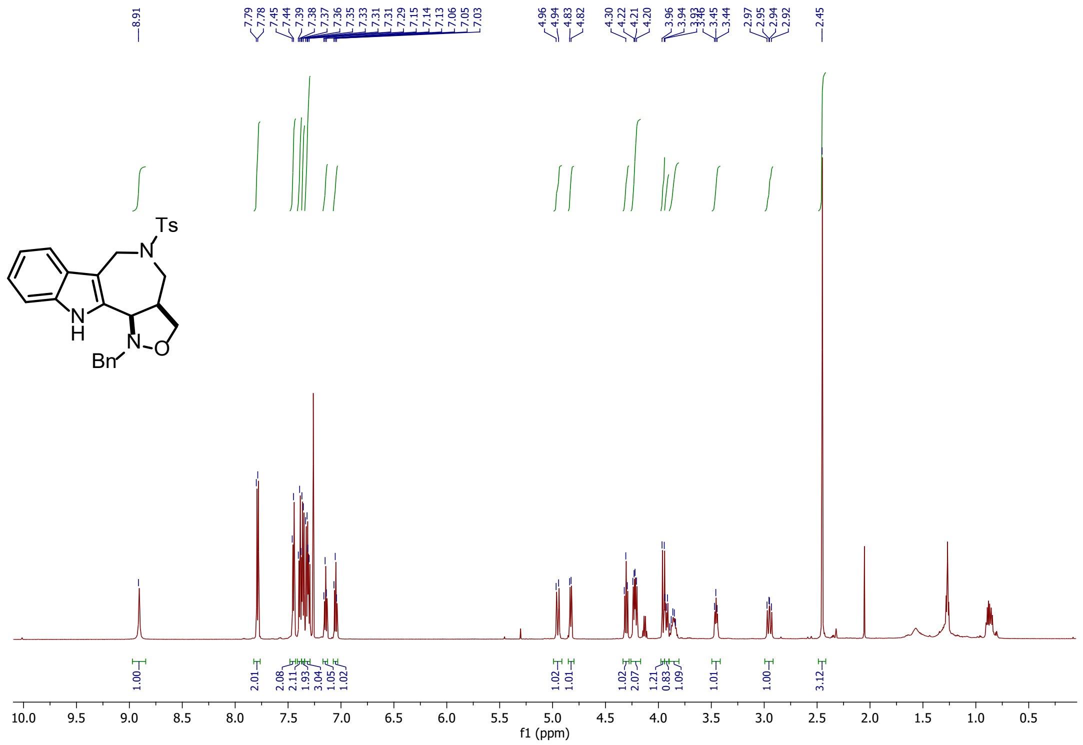

*Figure 129: **^{1}H NMR (600 MHz, CDCl3) of 7b.***

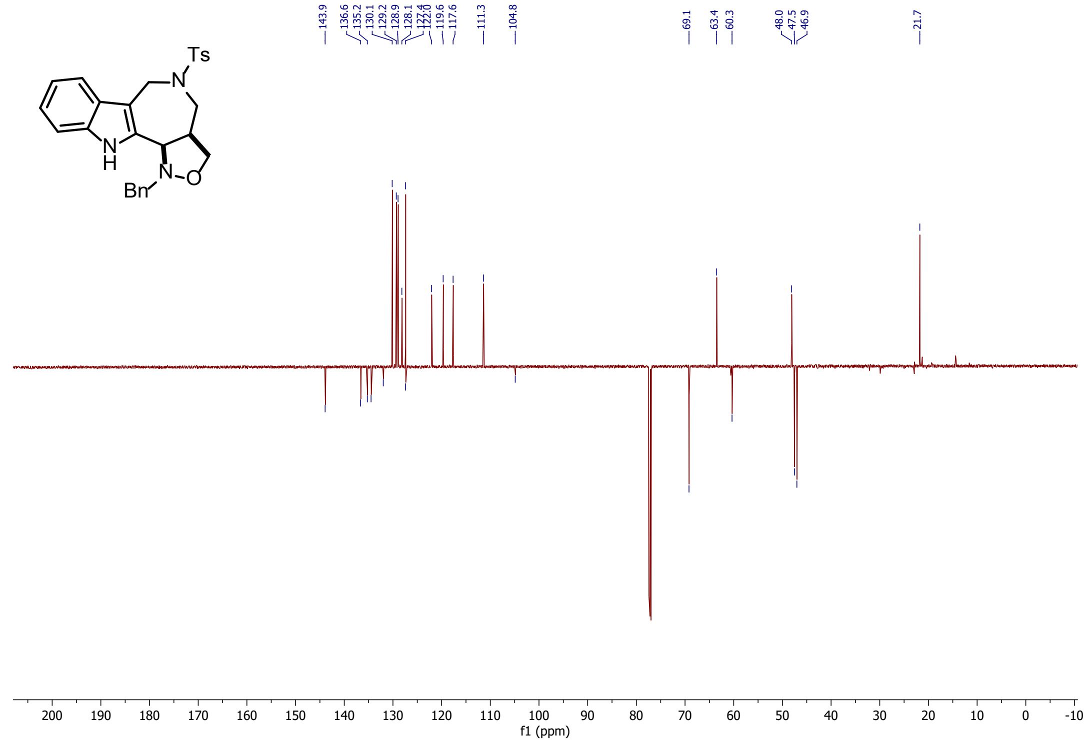

*Figure 130: **^{13}C NMR (151 MHz, CDCl3) of 7b.***

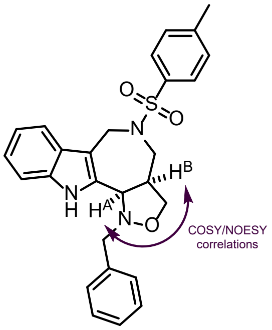

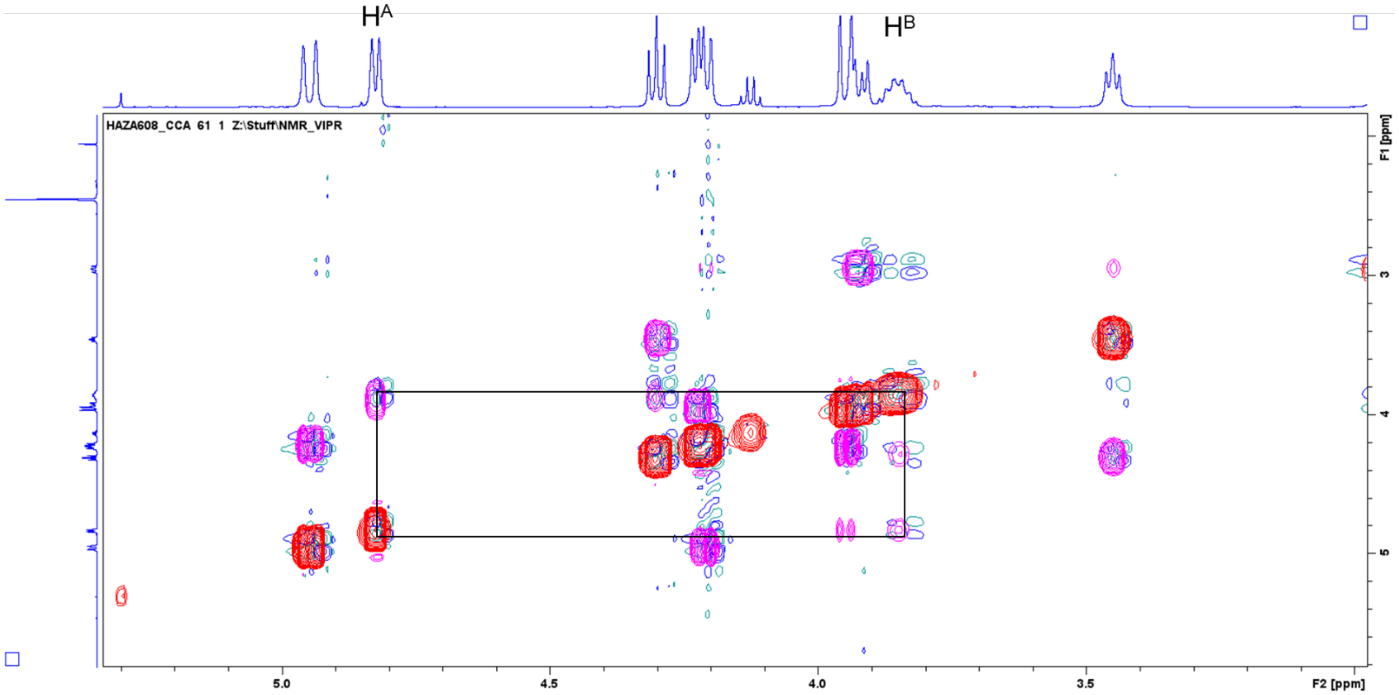

*Figure 131: **COSY (blue/green) and NOESY (fucshia) of 7b.***

2-benzyl-6-tosyl-1,4,5,6,7,12-hexahydro-2*H*-1,4-methano[1,2,7]oxadiazonino[4,5-*b*]indole (8b)

Due to some broad signals, spectra were reported as they appear; structure was further confirmed by reduction of the oxazolidine (cf. X-ray analysis).

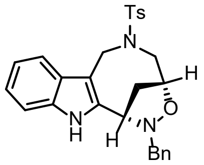

8b

CCDC ID: 2329785.

^{1}H NMR (600 MHz, CDCl$_3$) δ 7.97 (br s, 1H), 7.68 (d, *J* = 8.2 Hz, 3H), 7.25 (dd, *J* = 37.3, 33.7 Hz, 11H), 4.82 (dd, *J* = 139.3, 58.5 Hz, 2H), 4.36 (s, 1H), 4.19 (d, *J* = 8.0 Hz, 1H), 3.95 (t, *J* = 43.6 Hz, 1H), 3.78 (d, *J* = 13.0 Hz, 1H), 3.51 (s, 1H), 3.29 (d, *J* = 14.1 Hz, 1H), 2.70 (s, 1H), 2.49 – 2.36 (m, 4H).

^{13}C NMR (151 MHz, CDCl$_3$) δ 143.4, 136.9 (br), 136.7, 133.3, 129.8, 129.1 (br), 128.6 (br), 127.7 (br), 127.1, 122.1, 120.1, 118.5, 110.8, 62.3 (br), 61.6 (br), 60.9 (br), 56.9 (br), 50.87, 44.5, 43.7, 37.6, 34.4, 21.6. *One CH under the solvent peak.*

HRMS (ESI-TOF) m/z: $[M + H]^+$ Calcd for $C_{27}H_{28}N_3O_3S$ 474.1846; Found 474.1836.

IR (neat) vmax: 3373, 3060, 3031, 2924, 2854, 1598, 1494, 1454, 1336, 1156, 736.

Mp.: 204-205 °C.

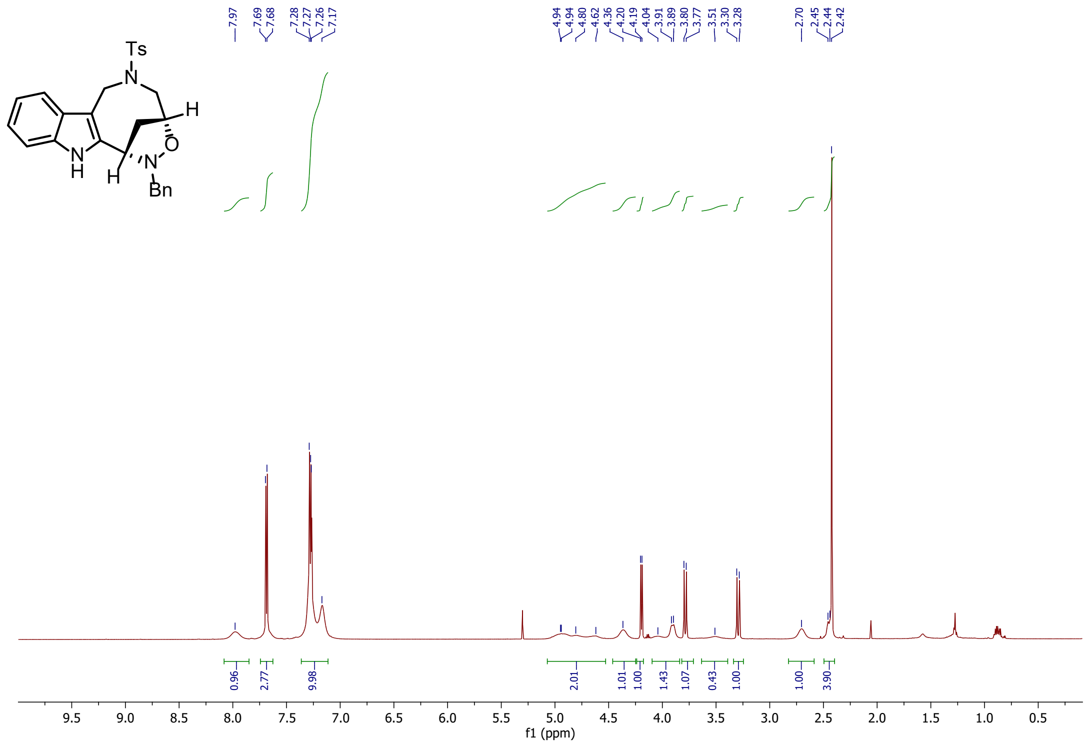

Figure 132: *^{1}H NMR (600 MHz, CDCl3) of 8b.*

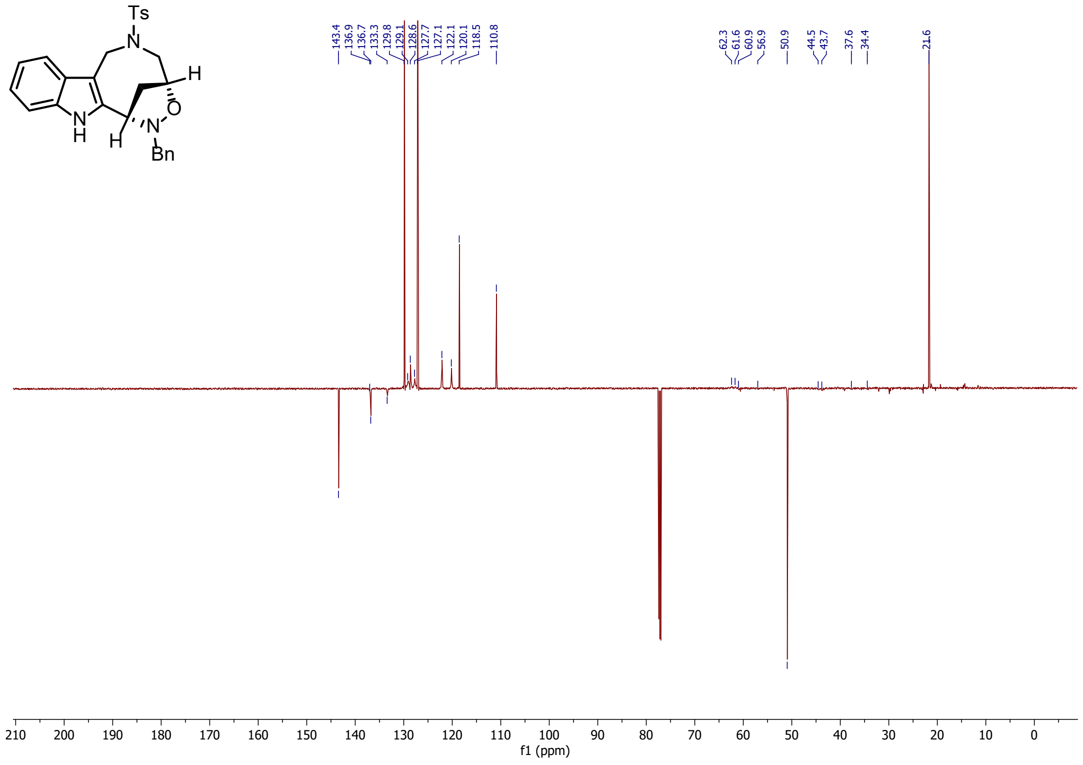

Figure 133: *^{13}C NMR (151 MHz, CDCl3) of 8b.*

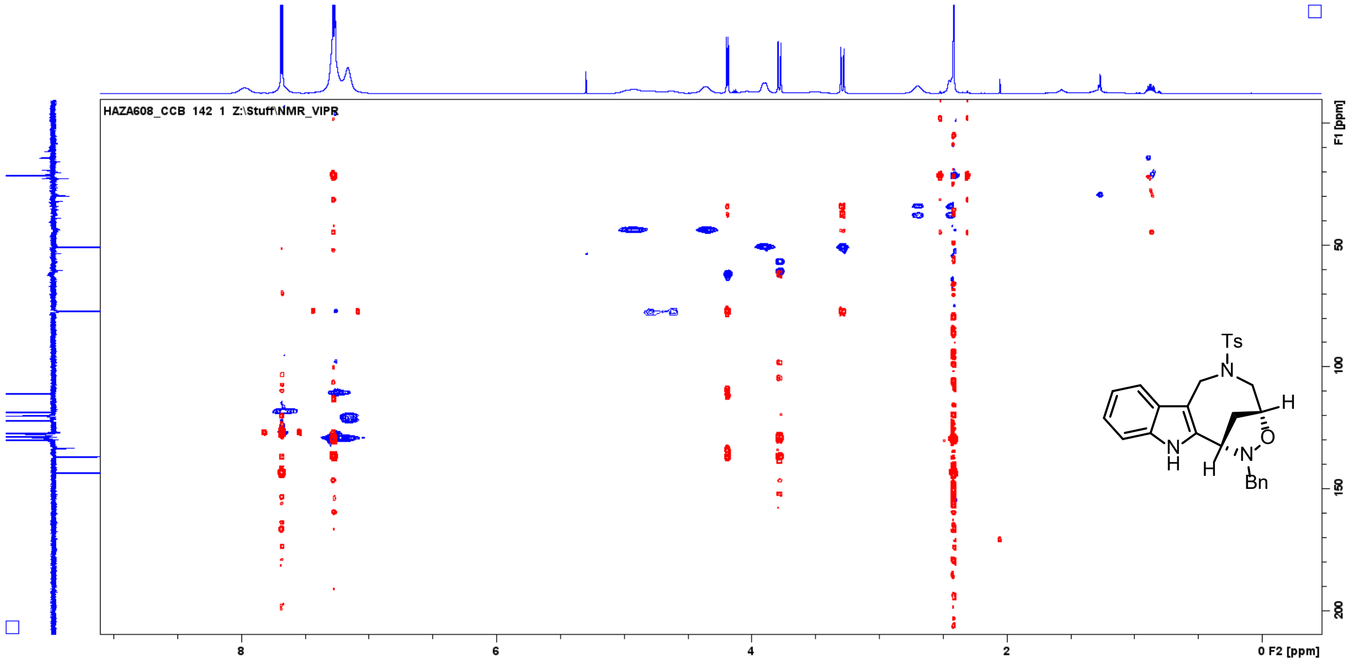

*Figure 134:**HSQC (blue) and HMBC (red) of 8b.***

(4*S,6*R**)-6-(benzylamino)-2-tosyl-2,3,4,5,6,7-hexahydro-1*H*-azocino[4,3-*b*]indol-4-ol (11)**

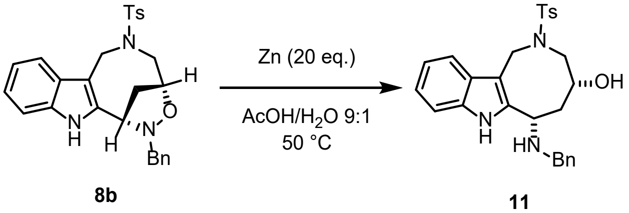

Cycloadduct **8b** (10 mg, 0.021 mmol, 1.0 eq.) was charged in a vial followed by Zn (28 mg, 0.42 mmol, 20 eq.), AcOH (450 μL) and H_2O (50 μL) and was heated at 50 °C for 3 h, before being allowed to return to ambient temperature. The mixture was diluted with EtOAc (5 mL) and a sat. aq. sol. of $NaHCO_3$ (5 mL) and was transferred to a separation funnel. The phases were separated and the aqueous phase was washed twice more with a sat. aq. sol. of $NaHCO_3$ (5 mL), followed by a sat. aq. sol. of NaCl (5 mL). The organic phase was dried over Na_2SO_4, filtered and concentrated under reduced pressure. The crude mixture was purified by flash chromatography using a gradient of heptanes/EtOAc/EtOH to yield a light-yellow solid (8.0 mg, 0.017 mmol, 80% yield).

^{1}H NMR (400 MHz, $CDCl_3$) δ 8.99 (br s, 1H), 7.79 (d, *J* = 8.2 Hz, 2H), 7.40 – 7.24 (m, 9H), 7.18 – 7.13 (m, 1H), 7.08 – 7.01 (m, 1H), 4.85 (d, *J* = 14.0 Hz, 1H), 4.58 (d, *J* = 9.2 Hz, 1H), 4.33 – 4.23 (m, 2H), 3.78 (s, 2H), 3.39 (dd, *J* = 14.7, 2.3 Hz, 1H), 2.84 (dd, *J* = 14.8, 9.1 Hz, 1H), 2.47 (s, 3H), 2.14 (dd, *J* = 13.2, 4.9 Hz, 1H), 1.87 (dt, *J* = 13.2, 10.2 Hz, 1H). *The OH and NH protons were not observed.*

^{13}C NMR (101 MHz, $CDCl_3$) δ 143.9, 139.7, 138.6, 134.68, 134.66, 130.1 (2C), 128.8 (2C), 128.2 (2C), 128.0, 127.7 (2C), 127.5, 122.0, 119.7, 117.6, 111.2, 104.6, 70.3, 52.6, 52.5, 51.4, 44.9, 43.6, 21.7.

HRMS (ESI-TOF) m/z: $[M + H]^+$ Calcd for $C_{27}H_{30}N_3O_3S$ 476.2002; Found 476.2000.

IR (neat) vmax: 3400, 2925, 1456, 1331, 1305, 1159.

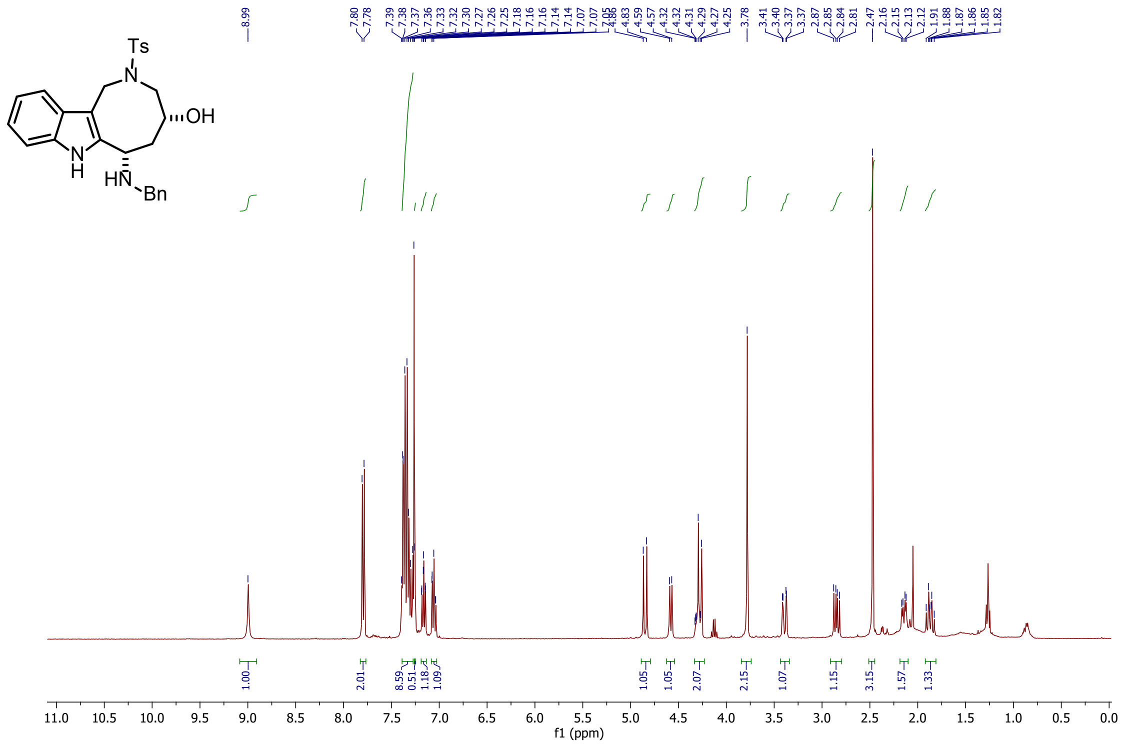

*Figure 135: **^{1}H NMR (400 MHz, CDCl3) of 11.***

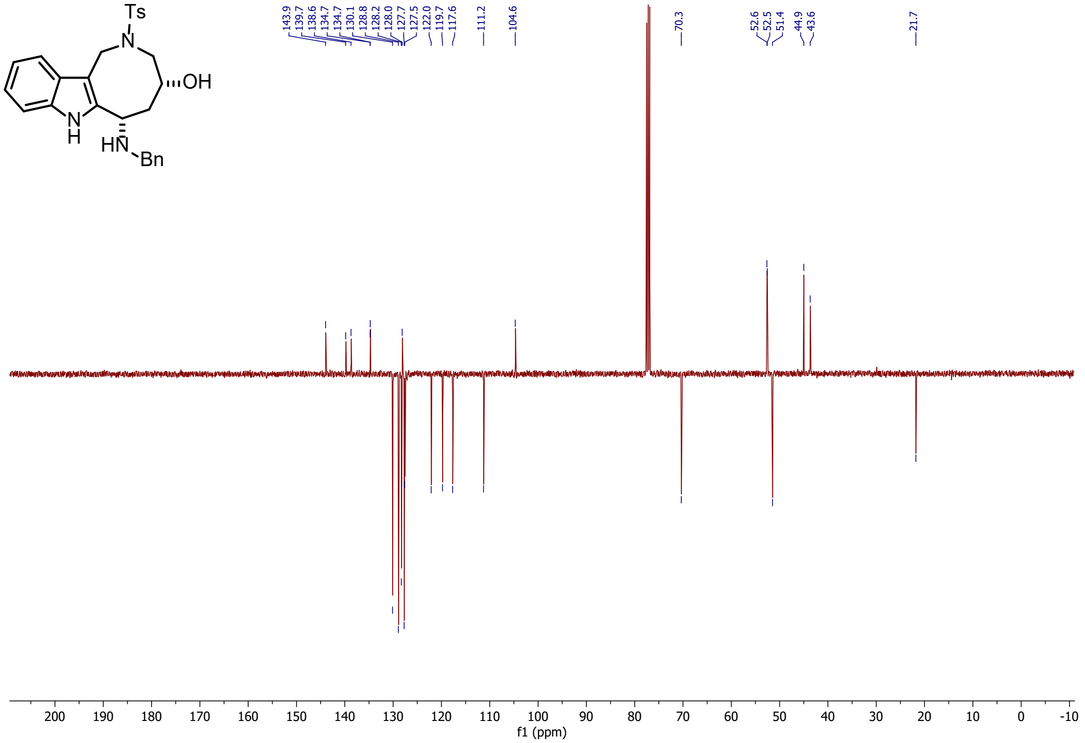

*Figure 136: **^{13}C NMR (101 MHz, CDCl3) of 11.***

Stereochemistry based on 2D NMR:

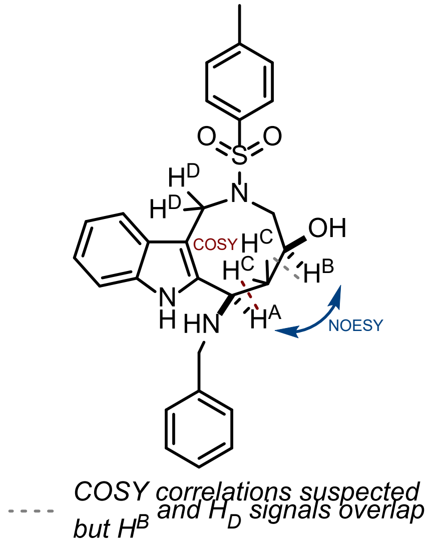

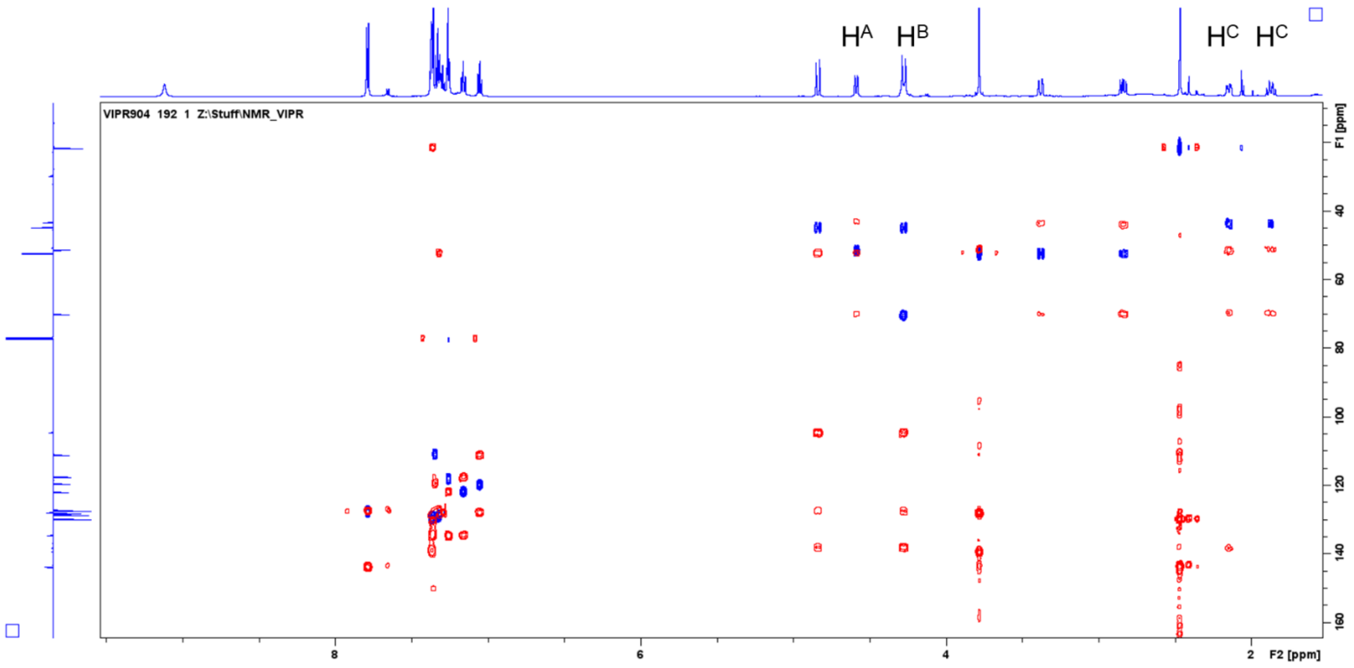

Figure 137: ***HSQC (blue) and HMBC (red) of 11.***

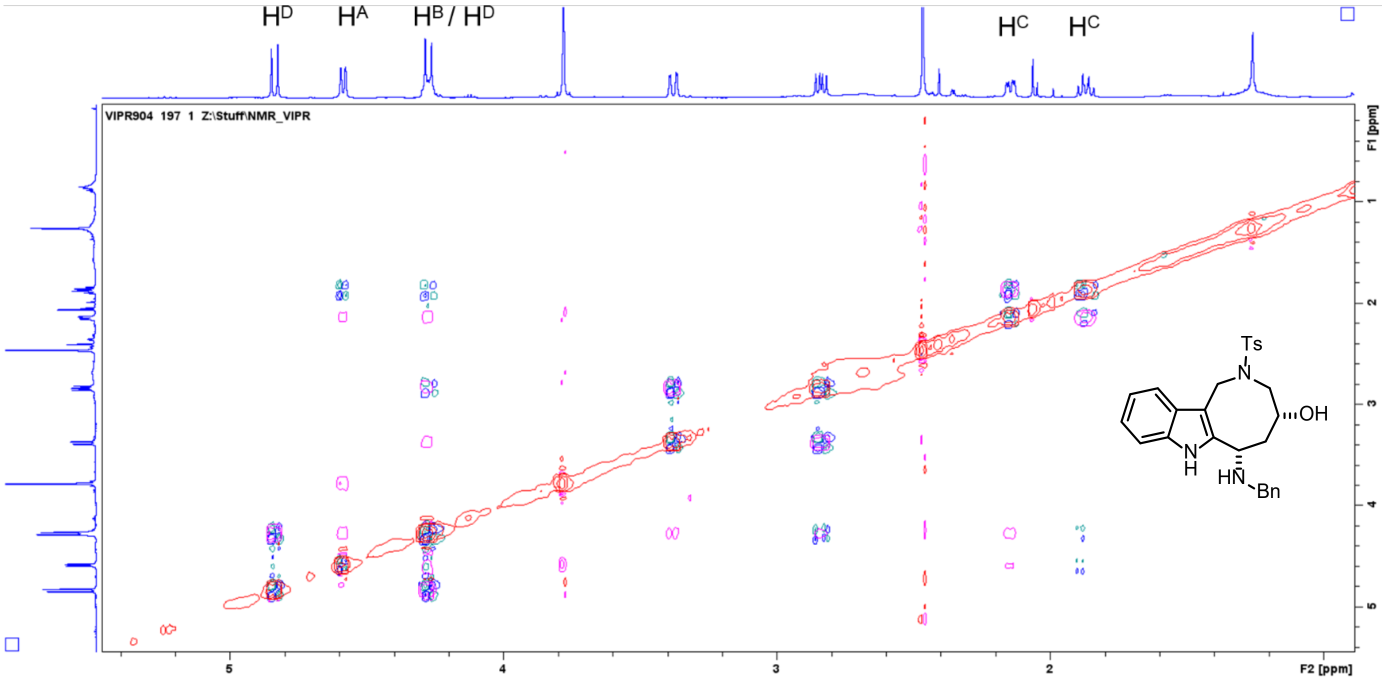

Figure 138: ***COSY (blue/green) and NOESY (fushia) of 11***.

(1S*,4S*)-2-benzyl-4-phenyl-6-tosyl-1,4,5,6,7,12-hexahydro-2H-1,4-methano[1,2,7]oxadiazonino[4,5-b]indole (8c)

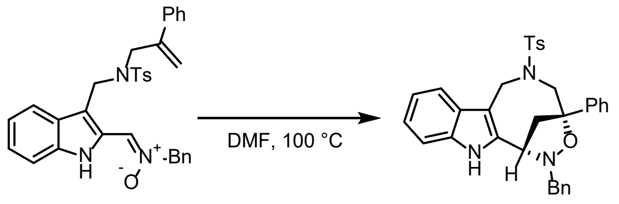

6c **8c**

A Schlenk flask was charged with **6c** (47 mg, 0.10 mmol, 1.0 eq) and DMF (1.8 mL). The mixture was stirred for 115 h at 100 °C, before cooling to ambient temperature. The work up was performed by diluting with CH_2Cl_2 (10 mL) and addition of a brine/H_2O mixture (1:1, 10 ml). After separation of phases, the aqueous layer was washed with additional CH_2Cl_2 (5 mL) four times. The combined organic layers were dried over Na_2SO_4, filtered and concentrated under reduced pressure. Purification was performed by flash chromatography using a gradient of heptanes/EtOAc to yield **8c** as an orange solid (21 mg, 0.044 mmol, 34% yield).

Due to some broad signals, spectra were reported as they appear; structure was further confirmed by reduction of the oxazolidine (cf. (4S*,6S*)-6-(benzylamino)-4-phenyl-2-tosyl-2,3,4,5,6,7-hexahydro-1H-azocino[4,3-b]indol-4-ol (**12**)).

^{1}H NMR (700 MHz, $CDCl_3$) δ 7.83 (br.s, 1H), 7.73 (br.s, 1H), 7.66 (br.s, 2H), 7.33 (br.s, 5H), 7.29 – 7.26 (m, 3H), 7.26 – 7.21 (m, 4H), 7.21 – 7.18 (m, 2H), 5.03 (br.s, 1H), 4.56 (d, *J* = 16.0 Hz, 1H), 4.36 (d, *J* = 7.9 Hz, 1H), 4.02 (br.s, 1H), 3.80 (br.s, 1H), 3.61 (br.s, 1H), 3.38 (d, *J* = 11.9 Hz, 1H), 3.15 (br.s, 1H), 2.99 (brs, 1H), 2.40 (s, 3H).

^{13}C NMR (171 MHz, $CDCl_3$) δ 143.3, 137.1, 133.6, 129.9 (2C), 129.0 (2C), 128.5 (2C), 128.3, 127.5 (2C), 127.0, 125.5, 122.3, 120.3, 118.6, 111.0, 63.7, 57.5, 57.0, 46.4, 44.6, 21.6. *One tertiary Csp^2 and five quaternary Csp^2 could not be observed under this conditions, pressumingly due to their broad nature.*

HRMS (ESI-TOF) m/z: $[M + Na]^+$ Calcd for $C_{33}H_{32}N_3O_3SNa$ 572.1978; Found 572.1967.

IR (neat) νmax: 3375, 3057, 3027, 2925, 2853, 1657, 1455, 1335, 1156, 738, 700, 659, 549.

Mp.: 232-234 °C.

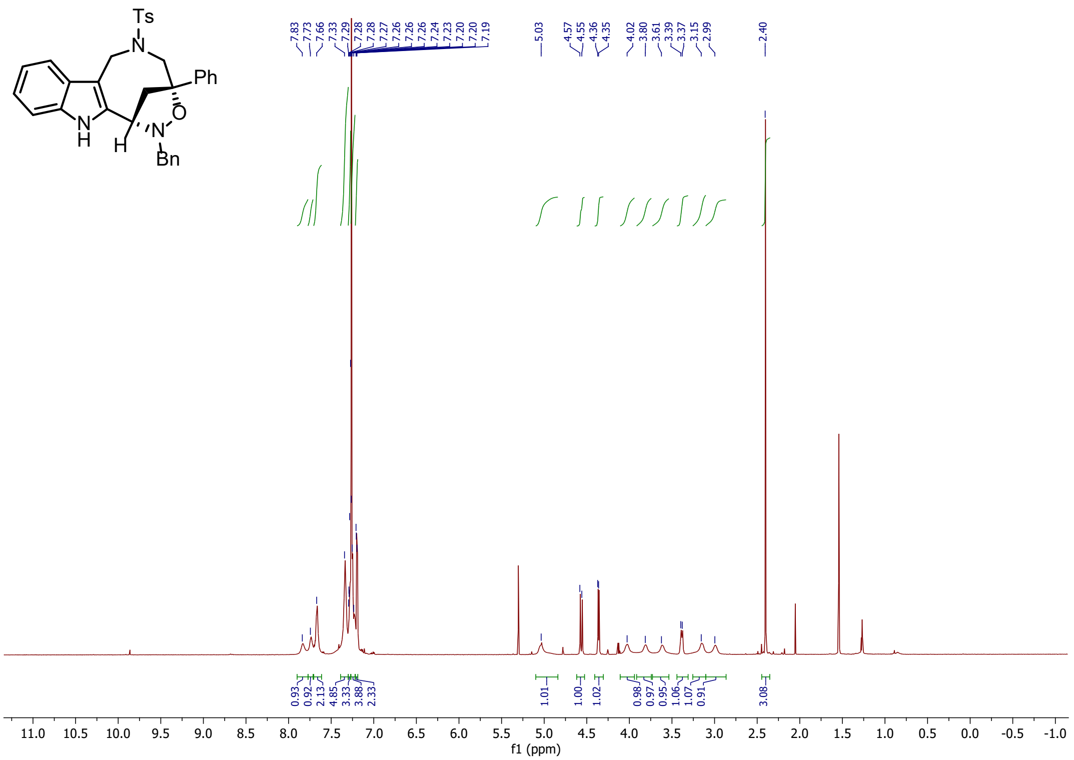

Figure 139: ***¹H NMR (700 MHz, CDCl₃) of 8c***

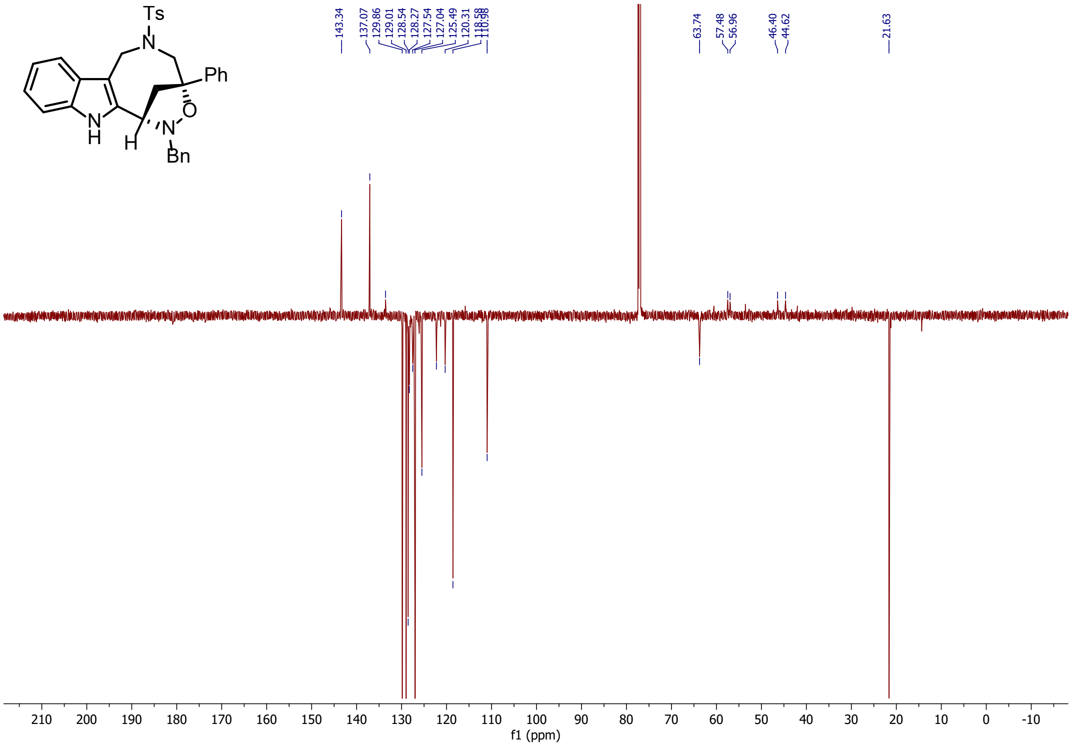

Figure 140: ***¹C NMR (171 MHz, CDCl₃) of 8c***

(4S*,6S*)-6-(benzylamino)-4-phenyl-2-tosyl-2,3,4,5,6,7-hexahydro-1H-azocino[4,3-b]indol-4-ol (12)

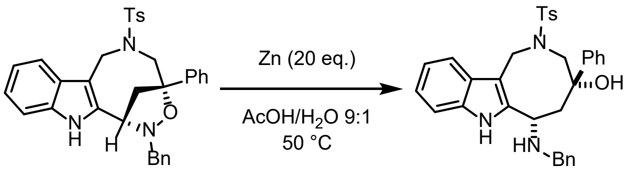

8c **12**

Cycloadduct **8c** (16 mg, 0.029 mmol, 1.0 eq.) was charged in a vial followed by Zn (38 mg, 0.58 mmol, 20 eq.), AcOH (900 µL) and H_2O (100 µL) and was heated at 50 °C for 14 h, before being allowed to return to ambient temperature. The mixture was diluted with EtOAc (5 mL) and a sat. aq. sol. of $NaHCO_3$ (5 mL) and was transferred to a separation funnel. The phases were separated and the aqueous phase was washed twice more with a sat. aq. sol. of $NaHCO_3$ (5 mL), followed by a sat. aq. sol. of NaCl (5 mL). The organic phase was dried over Na_2SO_4, filtered and concentrated under reduced pressure. The crude mixture was purified by flash chromatography using a gradient of heptanes/EtOAc/EtOH to yield **12** as a light-yellow solid (8.1 mg, 0.017 mmol, 50% yield).

^{1}H NMR (700 MHz, $CDCl_3$) δ 7.89 (br s, 1H), 7.81-7.71 (m, 1H), 7.71-7.63 (m 2H), 7.33 (br s, 4H), 7.29 – 7.26 (m, 2H), 7.25 – 7.17 (m, 5H), 5.02 (br s, 1H), 4.55 (d, J = 16.0 Hz, 1H), 4.36 (d, J = 7.9 Hz, 1H), 4.08-3.94 (m, 1H), 3.86-3.71 (m, 1H), 3.61 (br s, 1H), 3.34 (d, J = 12.5 Hz, 1H), 3.14 (br s, 1H), 2.96 (br s, 1H), 2.40 (s, 3H).

^{13}C NMR (171 MHz, $CDCl_3$) δ 145.9, 143.4, 137.2, 137.0, 133.6, 129.9 (2C), 129.0 (2C), 128.5, 128.3, 127.5, 127.4 (2C), 127.0, 125.5, 122.2, 120.3, 118.6, 111.0, 63.7, 57.5, 57.0, 46.3, 44.7, 21.6. *Five quaternary Csp^2 cannot be found under this conditions.*

HRMS (ESI-TOF) m/z: $[M + H]^+$ Calcd for $C_{33}H_{34}N_3O_3S$ 552.2315; Found 552.2307.

IR (neat) νmax:. 3406, 3061, 3028, 2925, 2856,1709, 1598, 1493, 1446, 1399, 1155, 727, 698, 547, 534.

Mp.: 228-230 °C.

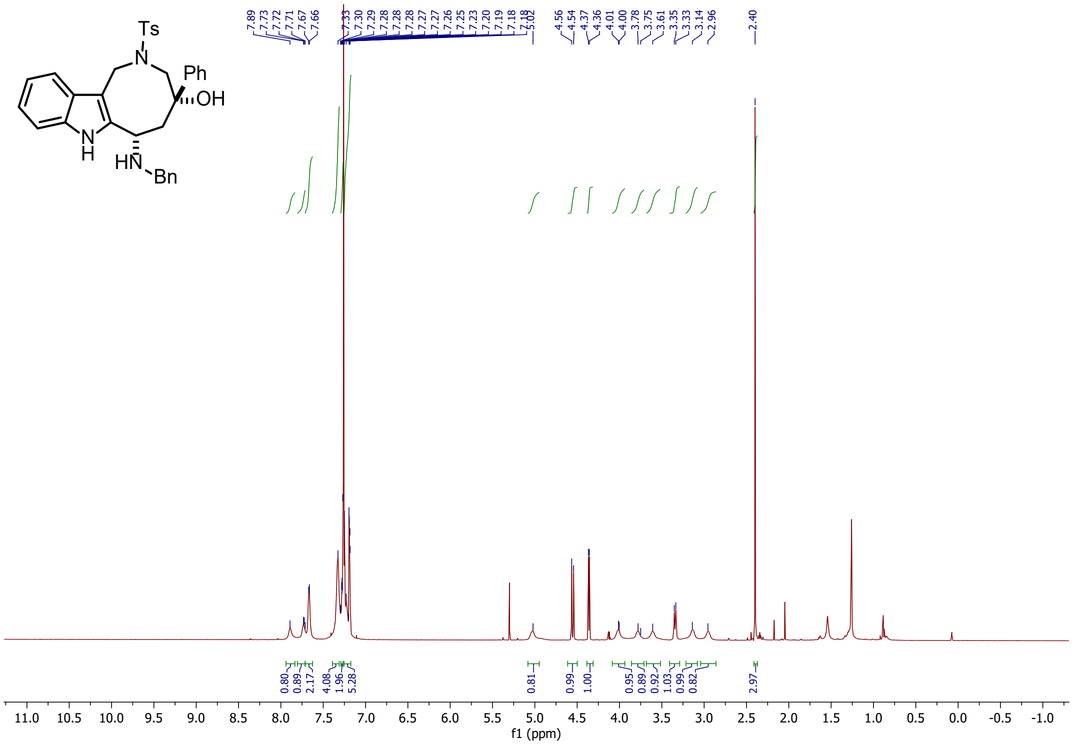

Figure 141: ***^{1}H NMR (700 MHz, CDCl₃) of 12.***

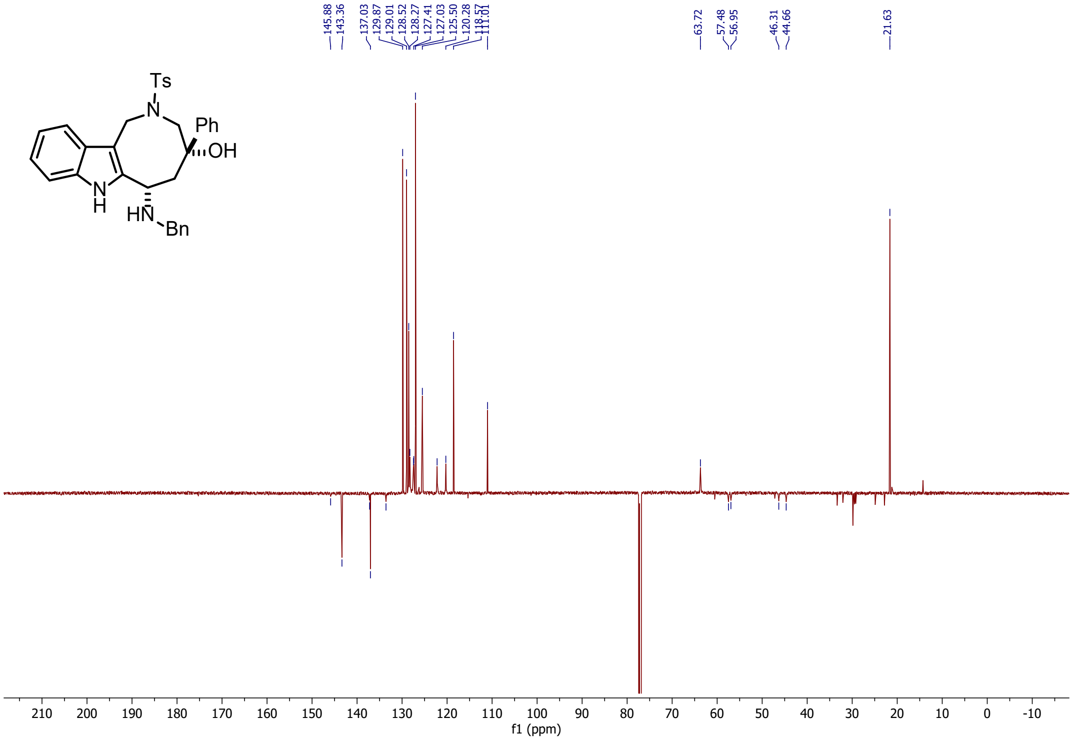

Figure 142: ***^{13}C NMR (171 MHz, CDCl₃) of 12.***

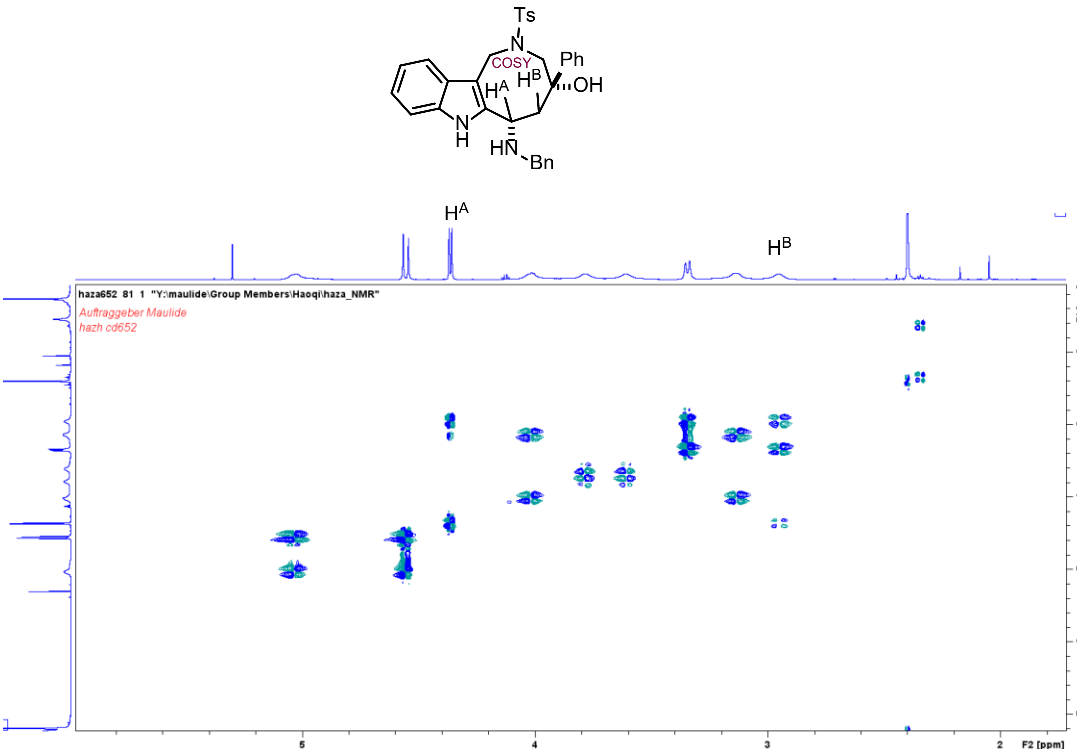

Figure 143: ***COSY of 12***.

***N*-allyl-*N*-((2-((hydroxyimino)methyl)-1-methyl-1H-indol-3-yl)methyl)-4-methylbenzenesulfonamide (9a)**

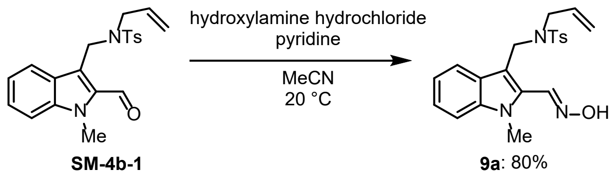

A 25 mL round-bottom flask was charged with **SM-4b-1** (139 mg, 0.363 mmol, 1.00 eq.) followed by MeCN (10 mL). Hydroxylammonium chloride (28 mg, 0.40 mmol, 1.1 eq.) and pyridine (0.18 mL, 2.2 mmol, 6.0 eq.) were added and the mixture was stirred for 5 h at 20 °C. Work-up was performed by dilution with Et_2O (10 mL) and H_2O (10 mL). The phases were separated and the aqueous phase was extracted twice with Et_2O (10 mL). The organic phases were combined, dried over $MgSO_4$ and filtered. The solution was concentrated under reduced pressure and the crude mixture was purified by flash chromatography using a gradient of heptanes/EtOAc to yield **9a** as a colourless solid (115 mg, 0.289 mmol, 80% yield).

^{1}H NMR (700 MHz, CD_2Cl_2) δ 8.41 (s, 1H), 7.76 – 7.71 (m, 3H), 7.63 (s, 1H), 7.33 (app. dd, J = 10.4, 8.2 Hz, 3H), 7.29 (ddd, J = 8.3, 6.8, 1.1 Hz, 1H), 7.11 (ddd, J = 7.9, 6.9, 1.0 Hz, 1H), 5.45 – 5.37 (m, 1H), 5.04 – 4.80 (m, 2H), 4.62 (s, 2H), 3.92 (s, 3H), 3.68 (d, J = 6.2 Hz, 2H), 2.45 (s, 3H).

^{13}C NMR (176 MHz, CD_2Cl_2) δ 144.1, 143.2, 139.3, 137.6, 133.4, 130.3, 130.0, 127.8, 127.6, 124.5, 120.7, 120.5, 118.8, 112.9, 110.1, 110.1, 49.8, 41.9, 32.8, 21.8, 21.8.

HRMS (ESI-TOF) m/z: $[M + H]^+$ Calcd for $C_{21}H_{23}O_3N_3SNa$ 420.1352; Found 420.1347.

IR (neat) νmax: 3410, 3055, 2917, 2849, 1719, 1682, 1597, 1151, 741, 545.

Mp.: 107-108 °C.

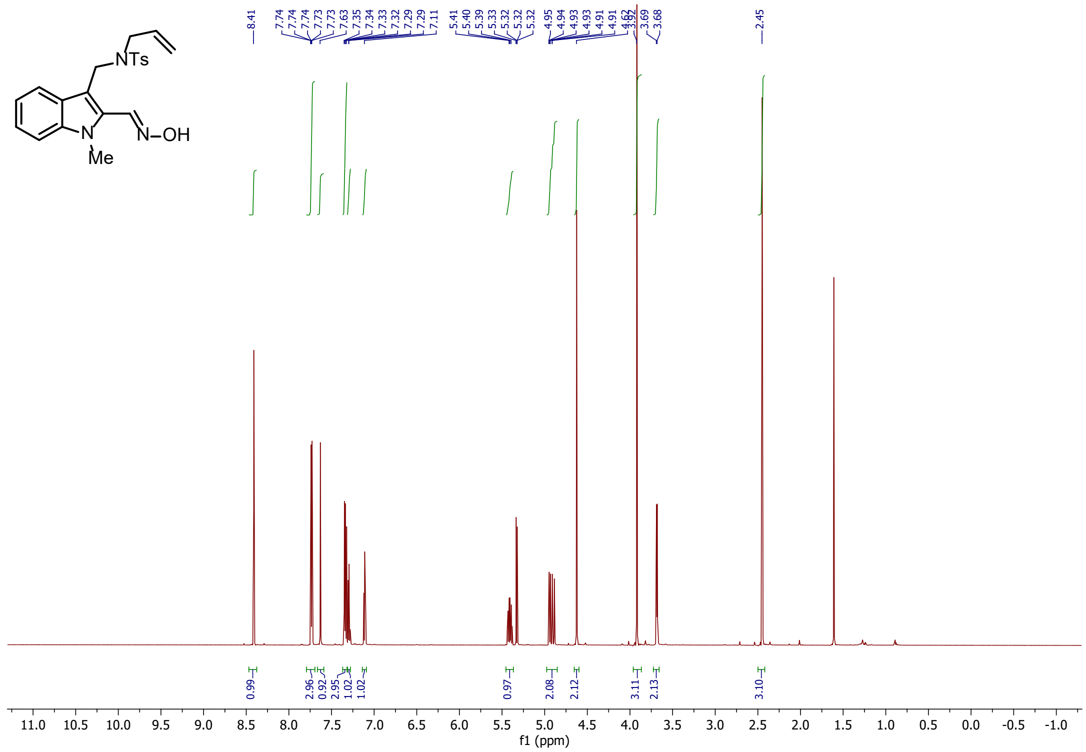

Figure 144: ¹H NMR (700 MHz, CD₂Cl₂) of 9a.

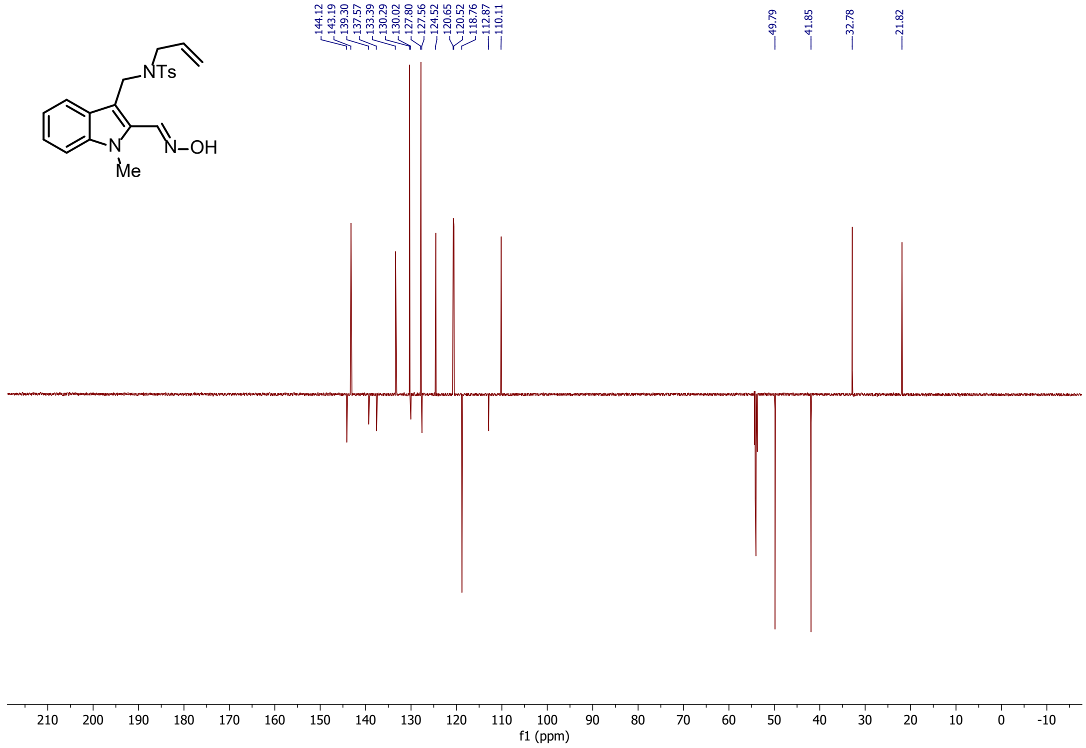

Figure 145: ¹³C NMR (176 MHz, CD₂Cl₂) of 9a.

11-methyl-5-tosyl-3,3a,4,5,6,11-hexahydroisoxazolo[3',4':5,6]azepino[4,3-b]indole (10a)

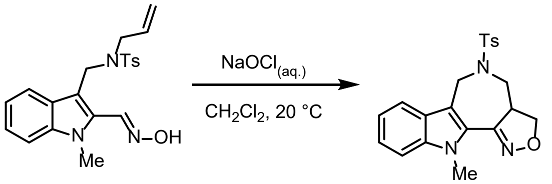

A 12 mL vial was charged with **9a** (60 mg, 0.15 mmol, 1.0 eq.), CH_2Cl_2 (5 mL) and a 13.5% aqueous sodium hypochlorite solution (0.14 mL, 0.30 mmol, 2.0 eq.). The mixture was stirred at 20 °C for 5 h before dilution with CH_2Cl_2 (3 mL) and H_2O (5 mL). The phases were separated and the aqueous phase was extracted twice with CH_2Cl_2 (3 mL). The organic phases were combined, dried over $MgSO_4$ and filtered. The solution was concentrated under reduced pressure and the crude mixture was purified by flash chromatography using a gradient of heptanes/EtOAc to yield **10a** as a colourless solid (47 mg, 0.12 mmol, 79% yield).

^{1}H NMR (600 MHz, CDCl$_3$) δ 7.60 (d, J = 8.0 Hz, 1H), 7.56 (d, J = 8.3 Hz, 2H), 7.33 (ddd, J = 22.1, 11.8, 4.5 Hz, 2H), 7.18 (ddd, J = 7.9, 6.8, 1.1 Hz, 1H), 7.13 (d, J = 8.1 Hz, 2H), 5.01 (d, J = 16.3 Hz, 1H), 4.72 – 4.64 (m, 1H), 4.56 (d, J = 16.3 Hz, 1H), 4.14 (dd, J = 13.6, 2.8 Hz, 1H), 4.01 – 3.92 (m, 2H), 3.89 (s, 3H), 3.23 (ddd, J = 13.5, 8.1, 2.6 Hz, 1H), 2.35 (s, 3H).

^{13}C NMR (151 MHz, CDCl$_3$) δ 152.8, 143.7, 138.71, 136.0, 129.7, 127.2, 125.9, 125.7, 124.7, 120.5, 119.0, 115.3, 110.0, 71.7, 50.6, 49.9, 44.2, 32.5, 21.6.

HRMS (ESI-TOF) m/z: $[M + H]^+$ Calcd for $C_{21}H_{21}O_3N_3SNa$ 418.1196; Found 418.1192.

IR (neat) vmax: 3045, 2924, 2875, 1596, 1523, 1394, 1157, 542.

Mp.: 204-206 °C.

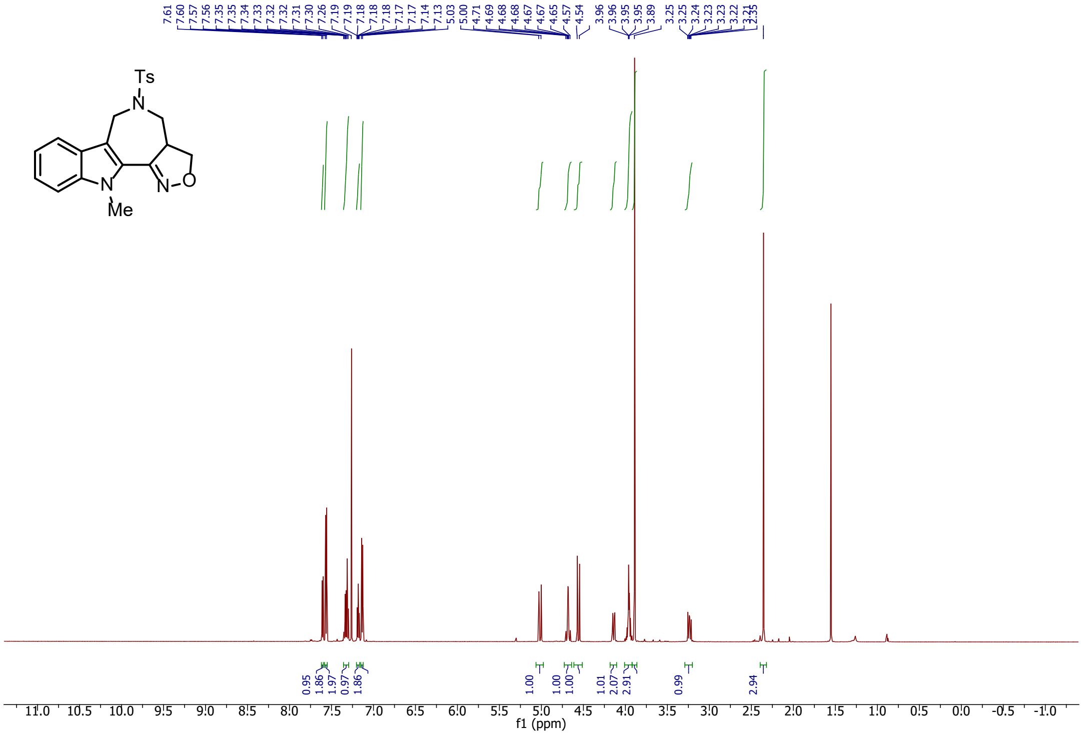

Figure 146: ¹H NMR (600 MHz, $CDCl_3$) of 10a.

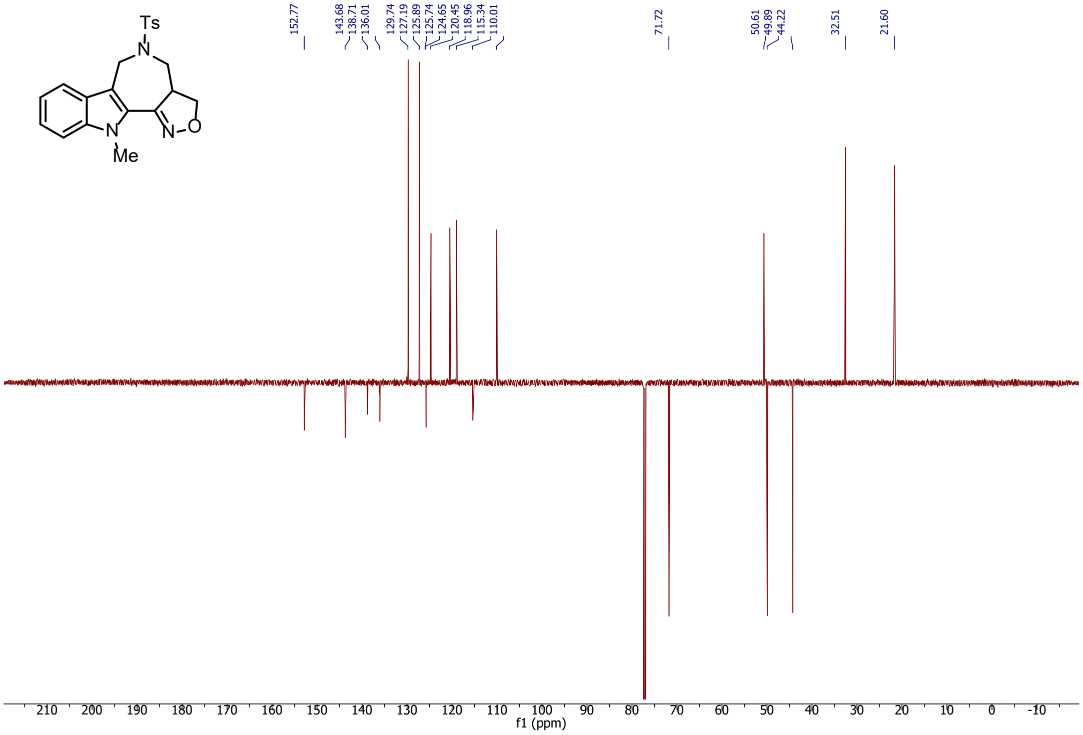

Figure 147: ¹³C NMR (151 MHz, $CDCl_3$) of 10a.

methyl-4-*N*-(2-(hydroxyimino)methyl)-1-methyl-1H-indol-3-yl)methyl)-4-methylphenyl)sulfonamido)but-2-enoate (9b)

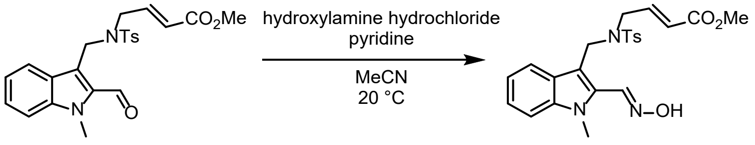

4b **9b**: 57%

A 25 mL round-bottom flask was charged with **SM-4b-1** (135 mg, 0.306 mmol, 1.00 eq.) followed by MeCN (10 mL). Hydroxylammonium chloride (23 mg, 0.34 mmol, 1.1 eq.) and pyridine (0.15 mL, 1.8 mmol, 6.0 eq.) were added and the mixture was stirred for 5 h at 20 °C. Work-up was performed by dilution with Et_2O (10 mL) and H_2O (10 mL). The phases were separated and the aqueous phase was extracted twice with Et_2O (5 mL). The organic phases were combined, dried over $MgSO_4$ and filtered. The solution was concentrated under reduced pressure and the crude mixture was purified by flash chromatography using a gradient of heptanes/EtOAc to yield **9b** as a colourless solid (80 mg, 0.18 mmol, 57% yield).

^{1}H NMR (600 MHz, $CDCl_3$) δ 8.32 (s, 1H), 7.74 (d, *J* = 8.2 Hz, 2H), 7.70 (d, *J* = 8.0 Hz, 1H), 7.44 (d, *J* = 7.3 Hz, 1H), 7.35 – 7.27 (m, 4H), 7.13 (ddd, *J* = 7.9, 6.3, 1.6 Hz, 1H), 6.47 (dt, *J* = 15.7, 6.0 Hz, 1H), 5.52 (dt, *J* = 15.7, 1.5 Hz, 1H), 5.30 (s, 3H), 4.66 (s, 2H), 3.87 (s, 3H), 3.79 (dd, *J* = 6.0, 1.5 Hz, 2H), 3.62 (s, 3H), 2.46 (s, 3H).

^{13}C NMR (151 MHz, $CDCl_3$) δ 166.3, 143.8, 143.3, 142.3, 138.6, 136.4, 130.0, 129.7, 127.6, 127.3, 124.4, 122.4, 120.7, 120.1, 111.5, 109.6, 51.7, 47.6, 42.1, 32.0, 21.7.

HRMS (ESI-TOF) m/z: $[M + H]^+$ Calcd for $C_{23}H_{25}O_5N_3SNa$ 478.1407; Found 478.1406.

IR (neat) vmax: 3301, 2951, 1721, 1659, 1471, 1152, 898, 745, 544.

Mp.: 146-148 °C.

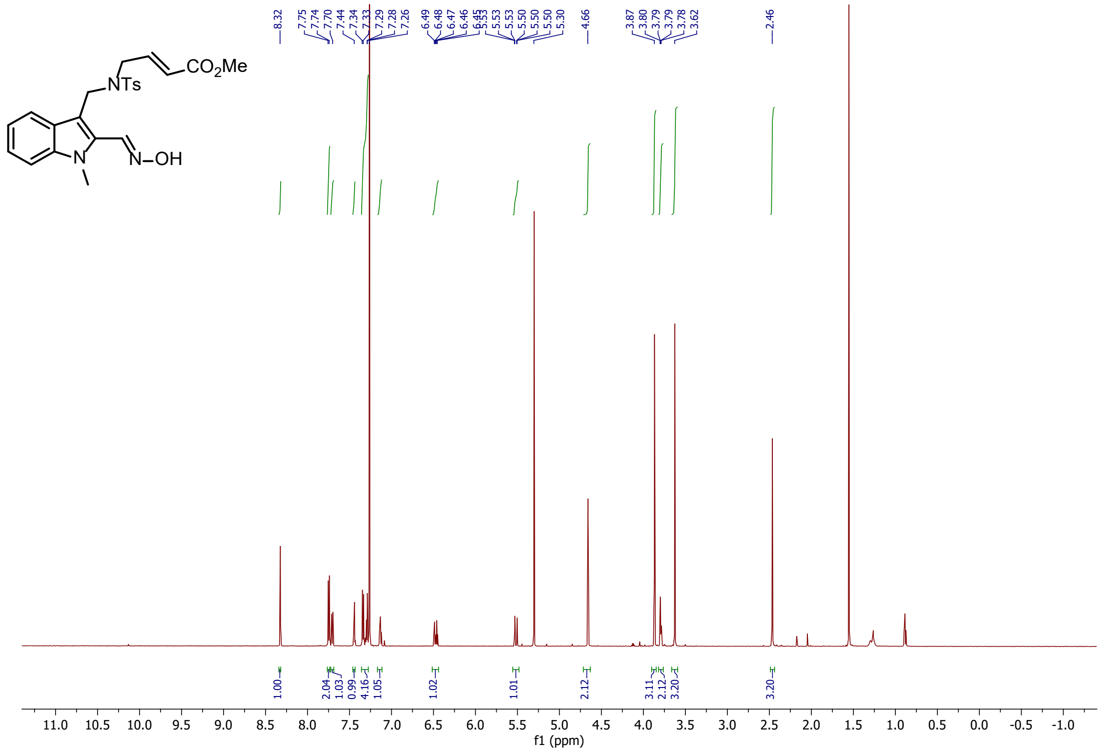

Figure 148: ***^{1}H NMR (600 MHz, CDCl3) of 9b.***

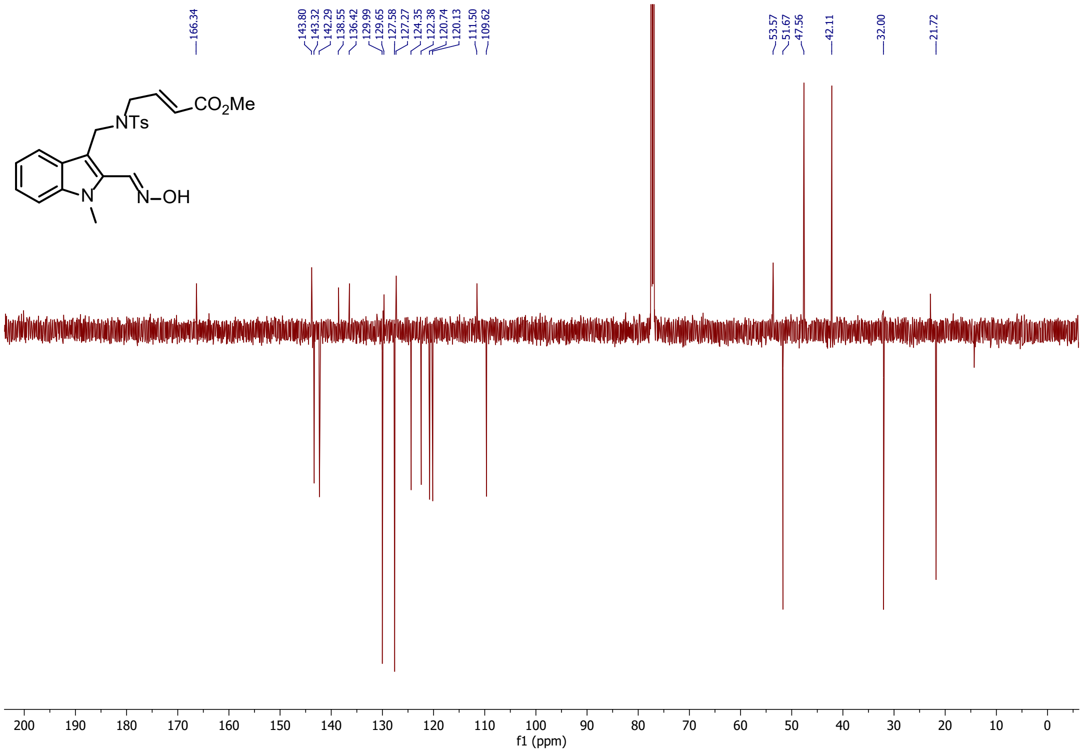

Figure 149: ***^{13}C NMR (151 MHz, CDCl3) of 9b.***

methyl (3*S**,3a*S**)-11-methyl-5-tosyl-3,3a,4,5,6,11-hexahydroisoxazolo[3',4':5,6]azepino[4,3-b]indole-3-carboxylate (10b)

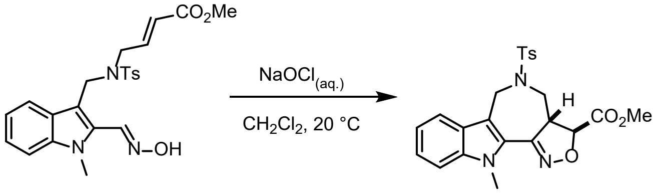

A 12 mL vial was charged with **9b** (50 mg, 0.11 mmol, 1.0 eq.), CH_2Cl_2 (5 mL) and a 13.5% aqueous sodium hypochlorite solution (0.10 mL, 0.22 mmol, 2.0 eq.). The mixture was stirred at 20 °C for 5 h before dilution with CH_2Cl_2 (3 mL) and H_2O (5 mL). The phases were separated and the aqueous phase was extracted twice with CH_2Cl_2 (3 mL). The organic phases were combined, dried over $MgSO_4$ and filtered. The solution was concentrated under reduced pressure and the crude mixture was purified by flash chromatography using a gradient of heptanes/EtOAc to yield **10b** as a colourless solid (35 mg, 0.077 mmol, 70% yield).

^{1}H NMR (600 MHz, $CDCl_3$) δ 7.61 (d, J = 8.0 Hz, 1H), 7.51 (d, J = 8.3 Hz, 2H), 7.38 – 7.32 (m, 1H), 7.30 (d, J = 8.3 Hz, 1H), 7.21 – 7.16 (m, 1H), 7.09 (d, J = 8.0 Hz, 2H), 4.95 (d, J = 16.5 Hz, 1H), 4.69 (t, J = 13.1 Hz, 2H), 4.21 (dd, J = 13.2, 4.6 Hz, 1H), 4.06 (ddd, J = 10.9, 9.8, 4.8 Hz, 1H), 3.88 (s, 3H), 3.86 (s, 3H), 3.45 (dd, J = 13.4, 11.0 Hz, 1H), 2.34 (s, 3H).

^{13}C NMR (151 MHz, $CDCl_3$) δ 169.3, 152.1, 143.7, 138.8, 136.1, 129.7, 127.2, 125.7, 125.0, 124.8, 120.6, 119.1, 116.3, 110.1, 80.8, 54.4, 53.3, 50.0, 43.9, 32.4, 21.6.

HRMS (ESI-TOF) m/z: $[M + H]^+$ Calcd for $C_{23}H_{23}O_5N_3SNa$ 476.1251; Found 476.1250.

IR (neat) νmax: 2950, 2923, 2913, 1763, 1666, 1334, 1160, 752, 658, 544.

Mp.: 180-181 °C.

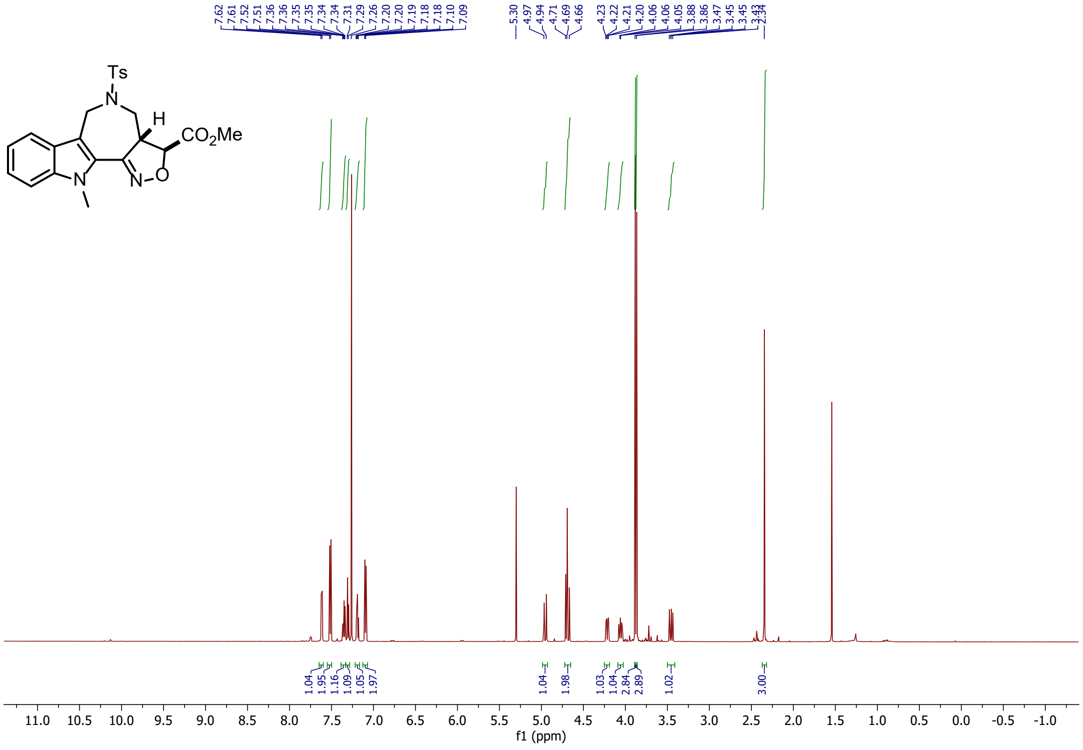

*Figure 150: [1]**H NMR (600 MHz, CDCl3) of 10b.***

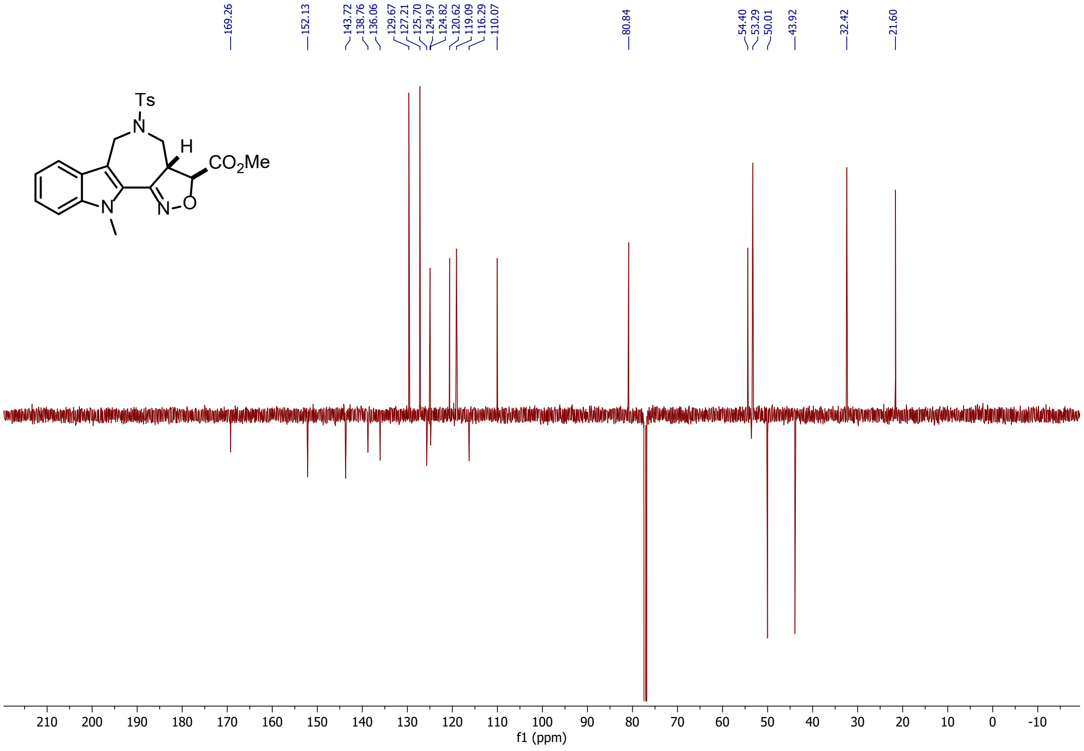

*Figure 151: **13C NMR (151 MHz, CDCl3) of 10b.***

VI. X-ray Analysis

General Information

Single-crystal X-ray diffraction data were collected with a Stadivari Diffractometer (STOE & Cie GmbH, Germany) equipped with an EIGER2 R500 detector (Dectris Ltd, Switzerland). Data were processed and scaled with the STOE software suite X-Area (STOE & Cie GmbH). Structures were solved with SHELXT,[9] and refined with SHELXL[10] or Olex2.[11] Model building was done with Olex2 or ShelXle. The structure was validated with CHECKCIF (https://checkcif.iucr.org/). See the respective CIF file for exact versions and more details.

2-benzyl-6-tosyl-1,4,5,6,7,12-hexahydro-2*H*-1,4-methano[1,2,7]oxadiazonino[4,5-*b*]indole (8b)

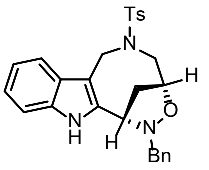

8b

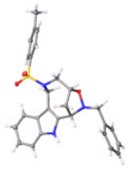

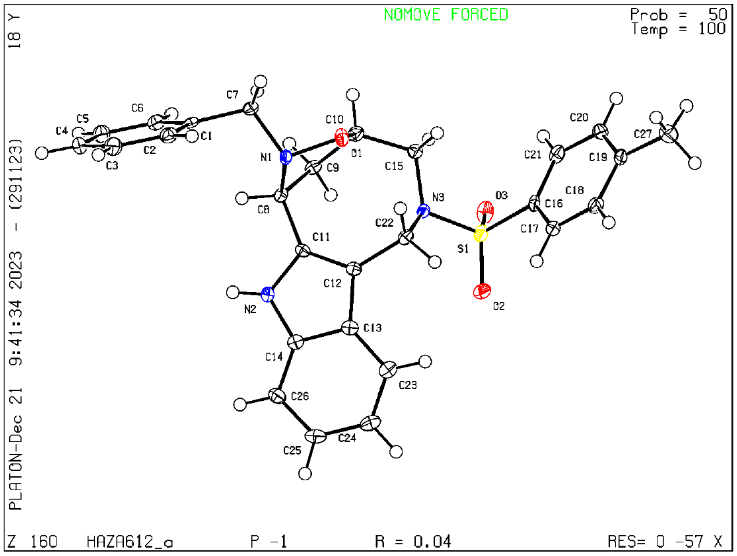

The sample for single crystal X-ray analysis was obtained by layered diffusion: **8b** (10 mg) was dissolved in 0.5 mL DCM and transferred to a NMR-tube. 0.5 mL pentane was slowly added through a syringe. The NMR tube was sealed with a cap and left alone for three days.

Table 1. Crystal data and structure refinement for ***8b****.*

Identification code	**8b**
Empirical formula	C27 H27 N3 O3 S
Formula weight	473.57
Temperature	100 K
Wavelength	0.71073 Å
Crystal system	Triclinic
Space group	P-1

Unit cell dimensions	a = 10.2816(7) Å	α= 66.958(5)°.
	b = 10.8922(7) Å	β= 80.897(5)°.
	c = 11.2934(8) Å	γ = 86.383(5)°.
Volume	1149.18(14) Å^3	
Z	2	
Density (calculated)	1.369 Mg/m^3	
Absorption coefficient	0.177 mm^{-1}	
F(000)	500	
Crystal size	0.400 x 0.273 x 0.160 mm^3	
Theta range for data collection	2.87 to 33.31°.	
Index ranges	-15<=h<=15, -16<=k<=16, -16<=l<=17	
Reflections collected	62296	
Independent reflections	8534 [R(int) = 0.0682]	
Completeness to theta = 25.242°	99.7 %	
Absorption correction	Semi-empirical from equivalents	
Max. and min. transmission	0.5154 and 0.2487	
Refinement method	Full-matrix least-squares on F^2	
Data / restraints / parameters	8534 / 7 / 308	
Goodness-of-fit on F^2	0.893	
Final R indices [I>2sigma(I)]	R1 = 0.0394, wR2 = 0.0746	
R indices (all data)	R1 = 0.0933, wR2 = 0.0782	
Extinction coefficient	n/a	
Largest diff. peak and hole	0.443 and -0.720 e.Å^{-3}	

VII. References

[1] G. R. Fulmer, A. J. M. Miller, N. H. Sherden, H. E. Gottlieb, A. Nudelman, B. M. Stoltz, J. E. Bercaw, K. I. Goldberg, *Organometallics* **2010**, *29*, 2176–2179.
[2] S. W. Youn, S. J. Pastine, D. Sames, *Org. Lett.* **2004**, *6*, 581–584.
[3] R. Murakami, D. Sekine, Y. Aoki, S. Kawamura, M. Sodeoka, *Tetrahedron* **2019**, *75*, 1327–1335.
[4] F. A. M. Mohamed, H. A. M. Gomaa, O. M. Hendawy, A. T. Ali, H. S. Farghaly, A. M. Gouda, A. H. Abdelazeem, M. H. Abdelrahman, L. Trembleau, B. G. M. Youssif, *Bioorg. Chem.* **2021**, *112*, 104960.
[5] W.-R. C. Ling Jong, *Analogs of Indole-3-Carbinol Metabolites as Chemotherapeutic and Chemopreventive Agents*, **2004**, 6800655B2.
[6] C. Swaby, A. Taylor, M. F. Greaney, *J. Org. Chem.* **2023**, *88*, 12821–12825.
[7] D. Janssen-Müller, M. Schedler, M. Fleige, C. G. Daniliuc, F. Glorius, *Angew. Chem. Int. Ed.* **2015**, *54*, 12492–12496.
[8] F. Scheidt, J. Neufeld, M. Schäfer, C. Thiehoff, R. Gilmour, *Org. Lett.* **2018**, *20*, 8073–8076.
[9] G. M. Sheldrick, *Acta Crystallogr A Found Adv* **2015**, *71*, 3–8.
[10] G. M. Sheldrick, *Acta Crystallogr. B* **2015**, *71*, 3–8.
[11] C. B. Hübschle, G. M. Sheldrick, B. Dittrich, *J. Appl. Crystallogr.* **2011**, *44*, 1281–1284.
